# Modelling estimates of the burden of Respiratory Syncytial virus infection in adults and the elderly in the United Kingdom

**DOI:** 10.1186/s12879-015-1218-z

**Published:** 2015-10-23

**Authors:** Douglas M. Fleming, Robert J. Taylor, Roger L. Lustig, Cynthia Schuck-Paim, François Haguinet, David J Webb, John Logie, Gonçalo Matias, Sylvia Taylor

**Affiliations:** Independent Consultant, 9 Dowles Close, Birmingham, B29 4LE United Kingdom; Sage Analytica, 4550 Montgomery Ave., Suite 4915 St. Elmo Ave, Ste. 205, Bethesda, MD 20814 USA; GSK Vaccines, Av Fleming 20, Parc de la Noire Epine, 1300 Wavre, Belgium; GSK Pharmaceuticals, Stockley Park West, 1-3 Ironbridge Road, Heathrow, Uxbridge, Middlesex, UB11 1B S United Kingdom

**Keywords:** Respiratory syncytial virus, Epidemiology, Adults, Elderly, Mortality, Hospitalisation

## Abstract

**Background:**

Growing evidence suggests respiratory syncytial virus (RSV) is an important cause of respiratory disease in adults. However, the adult burden remains largely uncharacterized as most RSV studies focus on children, and population-based studies with laboratory-confirmation of infection are difficult to implement. Indirect modelling methods, long used for influenza, can further our understanding of RSV burden by circumventing some limitations of traditional surveillance studies that rely on direct linkage of individual-level exposure and outcome data.

**Methods:**

Multiple linear time-series regression was used to estimate RSV burden in the United Kingdom (UK) between 1995 and 2009 among the total population and adults in terms of general practice (GP) episodes (counted as first consultation ≥28 days following any previous consultation for same diagnosis/diagnostic group), hospitalisations, and deaths for respiratory disease, using data from Public Health England weekly influenza/RSV surveillance, Clinical Practice Research Datalink, Hospital Episode Statistics, and Office of National Statistics. The main outcome considered all ICD-listed respiratory diseases and, for GP episodes, related symptoms. Estimates were adjusted for non-specific seasonal drivers of disease using secular cyclical terms and stratified by age and risk group (according to chronic conditions indicating severe influenza risk as per UK recommendations for influenza vaccination). Trial registration NCT01706302. Registered 11 October 2012.

**Results:**

Among adults aged 18+ years an estimated 487,247 GP episodes, 17,799 hospitalisations, and 8,482 deaths were attributable to RSV per average season. Of these, 175,070 GP episodes (36 %), 14,039 hospitalisations (79 %) and 7,915 deaths (93 %) were in persons aged 65+ years. High- versus low-risk elderly were two-fold more likely to have a RSV-related GP episode or death and four-fold more likely be hospitalised for RSV. In most seasons since 2001, more GP episodes, hospitalisations and deaths were attributable to RSV in adults than to influenza.

**Conclusion:**

RSV is associated with a substantial disease burden in adults comparable to influenza, with most of the hospitalisation and mortality burden in the elderly. Treatment options and measures to prevent RSV could have a major impact on the burden of RSV respiratory disease in adults, especially the elderly.

**Electronic supplementary material:**

The online version of this article (doi:10.1186/s12879-015-1218-z) contains supplementary material, which is available to authorized users.

## Background

The human respiratory syncytial virus (RSV) is well recognised as a major cause of respiratory infection in children [1], with annual epidemics in temperate climates. Around 40 million cases of RSV are estimated to occur annually in children under 5 years of age, of which 10 % are estimated to result in hospitalization [1]. In the United Kingdom (UK), epidemics typically start in late November and peak in mid-December. RSV seasonality appears to correlate with the onset of general practice (GP) consultations for acute bronchitis in children, followed 2–3 weeks later by the onset of acute bronchitis and hospital admissions due to respiratory disease in elderly adults [2, 3].

A growing body of evidence suggests an important role for RSV in elderly adults [4–7]. In a study in the United States (US), symptomatic RSV infection was detected annually in 3-7 % of 608 healthy community-living persons aged 65+ years followed for four years, and 8-13 % of 1388 patients hospitalised for acute respiratory infections during the same period [5]. In another prospective study conducted in 14 countries, RSV was detected among 7.4 % of 556 episodes of moderate-to-severe influenza-like-illness in elderly adults [8]. Other research suggests that age-related defects in cell-mediated immunity to RSV may lead to increased susceptibility and severe disease in older individuals [9, 10].

Despite the potentially important role of RSV in adults, nearly all RSV studies have focused on children and the burden among adults remains largely uncharacterised. Population-based studies relying on individual level data are severely limited by: the lack of standard testing for RSV among adults presenting to hospitals or GPs with acute respiratory disease; the expense of implementing such testing on a large-scale using sensitive PCR-based assays; the acute phase of RSV infections often resolving by the time patients seek medical care.

An alternative approach to furthering our understanding of the burden of RSV is to use indirect statistical modelling strategies. These strategies have long been used to assess the burden of influenza, and in more recent years have been applied to RSV. Modelling strategies circumvent some of the limitations of standard surveillance studies because they do not rely on direct linkage of exposure and outcome data at an individual level. Instead, they indirectly assess the effect of pathogens on the rates of various health outcomes.

Multiple regression time-series modelling is one such indirect strategy often used in disease burden assessment [11–13], using one or more time-series of independent variables to fit a function to a time-series of a dependent variable. The parameters associated with each term of the mathematical function that best fits the dependent time series estimate how much of the dependent variable being modelled (e.g. hospitalization for respiratory disease) can be attributed to the independent variables in the models (e.g. RSV and influenza).

In this study, multiple linear time series regression modelling was used to estimate the burden of RSV in the UK for the total population and adults, in terms of GP visits, hospitalizations, and deaths due to respiratory disease, using data from the Public Health England (PHE) weekly pathogen surveillance for influenza and RSV, the Clinical Practice Research Datalink (CPRD), the Hospital Episode Statistics (HES), and the Office of National Statistics (ONS) databases.

## Methods

Our study (NCT01706302) registered at www.clinicaltrials.gov was an extension of an earlier study (NCT01520935) conducted to evaluate the burden of influenza disease in the UK for the period 1995 through 2009, controlling for RSV incidence. The study protocol was approved by the Independent Scientific Advisory Committee from the CPRD. All data were anonymised and informed consent was not required.

### Data sources

#### General practitioner data

GP consultation data derived from the CPRD, which is the largest anonymised primary care database in the UK [14], drawn from clinical and prescribing records recorded general practitioners within their practices. At the midpoint of the study (Jan 2001) the monitored population was around 3.7 million. Patients included in the study were registered at approximately 500 distinct practices. All diagnoses and patient interventions are summarised and stored as READ codes. READ codes generally map to International Classification of Diseases (ICD) codes, with one or more READ codes per ICD code and additional non-ICD diagnostic codes for presenting symptoms which are widely used in primary care to describe minor illness. Under the guidance of two experts (DF, DW), we defined sets of READ codes corresponding to each ICD code of interest (provided in Additional file 1). In addition, preliminary searches of the CPRD dictionary were made to assess the frequency of symptom and other diagnostic codes used during three influenza peak periods, and the frequency in periods in which influenza did not circulate. This procedure revealed codes used by general practitioners to code patients who present with symptoms of acute respiratory infection during periods of influenza (and by extension RSV) activity.

A GP episode was counted as the first in a series of consultations for a particular diagnosis/diagnostic group which took place after a minimum of 28 days following any previous consultation for that same diagnosis/diagnostic group. Antibiotic prescription data (broad spectrum penicillins, macrolides, tetracyclines) were identified from prescriptions generated within the GP recording systems. Outcomes of interest were extracted from 1995 to 2009.

#### Hospitalization data

The HES database captures discharge data from all patients admitted to National Health Service non-psychiatric hospitals in England. We used ICD-10 codes corresponding to the outcomes of interest and listed as the primary diagnosis to extract records for each emergency admission from 1997 until 2009 (Table 1).

**Table 1:** Outcomes: GP episodes (CPRD), hospitalisations (HES), deaths (ONS)

Outcome	ICD10 codes	GP (CPRD)*	Hospitalisation (HES)***	Deaths (ONS)
Respiratory outcomes				
Respiratory disease	J00-99	X**	X	X
Cardiorespiratory disease	I00-99,J00-99		X	X
Acute upper resp disease	J00, J02-06	X		
Pneumonia & influenza	J09-18	X	X	X
Bronchitis/Bronchiolitis	J20-22, J40	X	X	X
Chronic resp disease	J41-47	X	X	X
Drug prescriptions				
Antibiotics (broad spectrum penicillins, macrolides, tetracyclines)^‡^		X	-	-
Non-Respiratory control outcomes				
Accidents	V00-99,X00-99,Y00-99		X	
Urinary Tract Infection	N39	X	X	X
Risk group conditions				
Chronic resp disease	J40-J47	X	X	X
Cardiovascular disorders	I00-I52 (except I01-04, I10, I30, I32-33, I40, I46, I49.1, I49.4)	X	X	X
Kidney disorders	N00-N29 (except N00, N10, N17, N20-23)	X	X	X
Diabetes	E10-E14	X	X	X
Immunosuppression	B20-24, O98.7, C00-C99, D37-D48, Z21	X	X	X
Liver disorders	K70-K77	X	X	X
Stroke	I60-I69	X	X	X
Central nervous system disorders	Q00-Q07, G10-G39, G45-46, G70-G99	X	X	X

CPRD does not provide any ranking of diagnostic codes. HES database outcomes listed as the primary discharge diagnosis were studied. ONS database outcomes listed as any mention of the outcome as the cause of death were studied

Risk group defined according to the chronic conditions indicative of risk for severe influenza as per UK recommendation for influenza vaccination

*Any CPRD GP episode, including office visits, home visits, telephone consults and other types, for subjects with subjects registered with research quality data in CPRD. CPRD diagnostic data are coded using READ codes. The list of appropriate Read codes for each definition was generated in consultation with a UK expert (Douglas Fleming) and GSK CPRD expert (David Webb). CPRD diagnostic, antibiotic and risk codes are given in Additional file 1

**Respiratory disease was broadly defined to consider CPRD READ codes corresponding to all ICD-listed codes related to respiratory disease as well as READ codes corresponding to selected symptoms and diagnoses in the CPRD (see description of selection procedure in Methods): these included cough, breathing abnormalities, viral infections, sepsis and septicemia

***Only unscheduled, “emergency” hospitalisations were included

^‡^antibiotics relevant to respiratory disease

#### Mortality data

The ONS records all deaths in England and Wales using ICD classification of cause (ICD-9 prior to 2001 and ICD-10 thereafter). As recommended by ONS [15], for respiratory outcomes that departed from trend estimates between 2000 and 2001 (‘all respiratory diagnoses’, ‘pneumonia and influenza’ and ‘bronchitis and bronchiolitis’), the average baseline incidence rate was adjusted by multiplying the 1996–2000 outcome counts by a constant (1.22 for ‘all respiratory diagnoses’, 1.69 for ‘pneumonia and influenza’ and 2.09 for ‘bronchitis and bronchiolitis’) to produce time-series that were not substantially different from 2000 to 2001 (transition period from ICD-9 to ICD-10). Patients who died in hospital were identified using HES data (England only). Outcomes of interest were extracted from 1996 to 2009.

#### Virology data

Weekly influenza and RSV counts were obtained from the surveillance system at PHE (England and Wales). Reports of RSV come primarily from laboratory-confirmed infections in young children admitted to hospital with respiratory disease, whereas reports of influenza are community-derived from individuals with influenza-like illness. Onset of RSV epidemics in children in England and Wales is determined by an obvious surge of RSV reports to PHE, usually, but not exclusively, in late November.

### Study inclusion criteria

The inclusion criterion was registration during the study period within a GP meeting the “research standard” checks of data quality and consistency in the CPRD, or a registration with a respiratory disease outcome in either the HES with an admission date between 1997 and 2009, or the ONS mortality database between 1996 and 2009 (Table 1).

#### Definitions of study outcomes and strata

The main study outcomes were GP episodes, hospitalizations and deaths due to respiratory disease. Respiratory disease was broadly defined to consider all ICD-listed codes (or corresponding CPRD READ codes) related to respiratory disease as well as READ codes corresponding to selected symptoms and diagnoses in the CPRD (see previously described selection procedure): these included cough, breathing abnormalities, viral infections, sepsis and septicaemia. In addition to cardiorespiratory disease (all ICD respiratory disease codes + cardiovascular codes) and other subcategories of respiratory disease, we examined the RSV-attributable burden associated with the selected antibiotic prescriptions (GP episodes only). Accidents and urinary tract infections, which have no seasonal pattern and no association to RSV or influenza, were used as negative control outcomes to assess the possibility of bias in model attribution over the entire study period. Outcomes related to GP episodes were defined according to any mention of a diagnostic READ code of interest. For hospitalisations (HES database), outcomes listed as the primary discharge diagnosis were studied. For deaths (ONS database) we studied any mention of the outcome as the cause of death.

In addition to estimating the RSV-attributable burden of respiratory disease for the total UK population, estimates were stratified by age and risk group. Data are presented in this manuscript for the following adult age groups: 18–49 years, 50–64 years, 65–74 years, and 75+ years (paediatric-specific data will be presented in a separate publication). Risk group was defined according to the presence of conditions indicative of severe influenza risk as per UK recommendations for influenza vaccination (including chronic cardiac and respiratory diseases, diabetes, immune suppression, chronic liver disease, stroke and multiple sclerosis) [16]. For GP episodes, because CPRD includes unique patient identifiers and records all GP consultations and diagnoses, we were able to follow patients longitudinally over the study years to determine patient risk group. We considered most conditions that give patients “high risk” status to be lifelong, with two exceptions: asthma (a consultation record for this diagnosis was required less than 3 years before the week under study, or, for earlier diagnoses, a drug prescription in the preceding 3 years) and immunosuppression (a prescription was required for an immunosuppressive drug in the previous year) (see Additional file 1 for lists of asthma and immunosuppressive drug codes used). For hospitalization and deaths, the presence of co-morbidities in each hospitalization or death record was used to determine the risk group.

### Statistical methods

Statistical analyses were performed using SAS 9.2. We used data restricted to the subset of patients that were covered by each source. UK population by age (2001 data: ONS [17]) were used to weight the CPRD population to reflect the UK profile.

Weekly time-series for influenza and RSV were calculated using PHE surveillance data. The number of the week was calculated as ISO 8601 V-weeks, which begin with ‘1’ near the beginning of the calendar year. Due to a change from using viral culture to PCR methods at PHE during the seasons studied, pathogen data were split into pre- and post-July 2001 season periods. Weekly time-series for each health outcome were generated and stratified by age and risk group. A multiple linear regression model was applied to each stratum of interest (i.e., age and risk group) to statistically associate outcomes to RSV, while controlling for influenza:

Y = β_0_+ β_s1_t + β_s2_t^2^+ β_s3_t^3^+ β_s4_sin(2πt/52) + β_s5_cos(2πt/52) + β_p1a_Influenza A(pre-July 2001) + β_p1b_Influenza A(post-July 2001) + β_p2a_Influenza B(pre-July 2001) + β_p2b_Influenza B(post-July 2001) + β_p3a_LagN(RSV)(pre-July 2001) + β_p3b_LagN(RSV)(post-July 2001)

where,

- Y = weekly rates of outcomes.
- t = time since 1 July 1995, in weeks.
- β_0_ = intercept
- β_s1_t + β_s2_t^2^+ β_s3_t^3^ = secular polynomial
- β_s4_sin(2πt/52) + β_s5_cos(2πt/52) = secular cyclical terms included to account for other seasonal events that are not related to the pathogens in the model.
- β_p1a_Influenza A(pre-July 2001) + β_p1b_Influenza A(post-July 2001) = pathogen influenza A
- β_p2a_Influenza B(pre-July 2001) + β_p2b_Influenza B(post-July 2001) = pathogen influenza B
- β_p3a_(RSV)(pre-July 2001) + β_p3b_(RSV)(post-July 2001) = pathogen RSV
- Influenza A, Influenza B and RSV are observed counts of positive tests from the PHE dataset.
- LagN = number of weeks by which the RSV data is lagged (varying by age group and database analysed).

Because more than 90 % of tests for RSV are performed on samples from infants and young children, various lagged forms of the influenza and RSV virology predictor variables were tested for each age group to determine the lagged model form that maximised the coefficient of multiple correlation R^2^.

The attribution to each virus was computed for each week as the product of the model parameter and corresponding explanatory variable. The weekly estimates were summed to provide a seasonal estimate and the confidence interval (CI) was based on the standard error of the pathogen parameter estimate. The RSV burden was derived from parameters βp3a and βp3b in the equation above. Results for influenza-attributable respiratory outcomes derived from the model were used to calculate the ratio of RSV-attributable outcomes as a proportion of all disease attributable to either influenza or RSV. The time-series of antibiotic prescriptions was modelled in the same way as the time-series for health outcomes to estimate antibiotic prescribing attributable to RSV.

## Results

### Databases and populations surveyed

Large numbers of cases across all age groups were available for most outcomes of interest. The number of events with a diagnosis of respiratory disease ranged between 629,000 and 925,000 for GP episodes, 392,000 and 568,000 for hospitalisations (primary diagnosis) and 124,000 and 140,000 for deaths (any mention) in each season.

### Model fit

With few exceptions RSV infection is reported in the UK solely in winter months and thus our study was confined to the winter period between September and mid-May. The outcome and pathogen time-series were smoothed using a moving average of order 3 (i.e., the smoothed value of X at week t is (X_t-1_ + X_t_ + X_t+1_)/3) in order to adjust for irregular utilisation of health services over the critical Christmas and New Year holiday periods. The best model fit was obtained by lagging the RSV viral series by two weeks for GP episodes for 50+ year olds (R^2^ = 81.2 %), and by three weeks for hospitalisation and mortality in individuals aged 18+ years (R^2^ = 78.6 % and 86.1 %, respectively). The model did not attribute any positive RSV burden to control outcomes.

### Burden of RSV among total UK population (including children)

Over the seasons studied we estimated that a seasonal average of 927,325 GP episodes (or roughly 1.6 % of the UK population) for RSV-attributable respiratory disease (Table 2). Among all GP episodes with a diagnostic code for respiratory disease, 5.9 % were attributable to RSV. With the exception of pneumonia and influenza, there were more GP episodes attributable to RSV than to influenza for all of the outcomes reported (Table 2).

**Table 2:** Mean RSV-attributable burden of respiratory disease among the total population in the United Kingdom

Respiratory Outcome*	Episodes /100,000 population (range)	N	% of events attributable to RSV**	RSV:influenza ratio †
GP episodes				
Respiratory disease	1579 (1055–1961)	927325	5.7	1.1:1
Acute upper respiratory disease	737 (509–884)	432716	6.0	1.2:1
Pneumonia and influenza	75 (34–123)	43825	8.5	0.2:1
Bronchitis/bronchiolitis	677 (472–801)	397665	11.5	1.6:1
COPD	70 (41–98)	41214	2.4	4.1:1
Antibiotic prescriptions	1553 (1111–1820)	912036	5.4	1.5:1
Hospitalisation (Primary diagnosis)				
Respiratory disease	83 (61–101)	48600	9.0	1.7:1
Pneumonia and influenza	15 (11–19)	8599	9.3	1.2:1
COPD	10 (8–13)	5912	3.9	0.8:1
Cardiorespiratory	69 (49–81)	40255	4.5	1.6:1
Bronchitis/bronchiolitis	47 (34–57)	27806	28.4	3.4:1
Mortality (Any mention)				
Respiratory disease	15 (11–21)	9034	5.8	0.8:1
Pneumonia and influenza	11 (8–15)	6603	7.0	0.7:1
COPD	4 (3–5)	2561	6.9	0.9:1
Cardiorespiratory	20 (16–26)	11600	3.9	0.8:1
Bronchitis/bronchiolitis	1 (0–1)	354	11.6	0.9:1

N = Average seasonal number of specified RSV-attributable events for each outcome

GP = general practice, COPD = chronic obstructive pulmonary disease

Range = range of estimates per season

*Outcomes refer to the ‘Primary’ cause for hospitalisation for HES, or as the ‘Any mention’ cause of death for ONS. For the GPRD data there is no ordered listing or ranking of diagnostic codes

**% of RSV-attributable events among all events due to outcome, † ratio of proportions/100,000 RSV/[influenza A + B]

There were an average of 48,600 hospitalisations and 9,034 deaths due to respiratory disease attributable to RSV in the UK population in each season, across all ages; hospitalisations for respiratory disease, pneumonia and influenza, bronchitis/bronchiolitis and cardiorespiratory disease were more often attributable to RSV than to influenza.

In a subsidiary analysis of deaths in hospital, we estimated that 58 % (5195/9034) of respiratory deaths occurred outside hospitals. The average number of RSV-attributable cardiorespiratory deaths per season was 28 % higher than the respiratory disease estimate (11,600 versus 9034)

The model estimated that 912,036 antibiotic prescriptions were attributed to RSV-associated respiratory disease per season across all age groups.

### RSV-attributable disease burden in adults

There were an average 487,247 GP episodes, 17,799 hospitalisations, and 8,482 deaths attributable to RSV respiratory disease per season in adults aged 18+ years. Of all RSV-attributable GP episodes, 52.5 % were in adults aged 18 years and older (Table 3). Of these, 175,070 GP episodes (36 %), 14,039 hospitalisations (79 %) and 7,915 deaths (93 %) were in persons aged 65+ years (Table 4).

**Table 3:** Mean seasonal RSV-attributable burden of GP episodes, hospital admissions and deaths due to respiratory disease among adults by age group; United Kingdom 1995-2009

	GP episodes				Hospitalisations				Deaths			
Age (years)	Episodes/100,000 population (range)	*N*	% of events attributable to RSV**	RSV:influenza ratio†	Hospitalisations/100,000 population (range)	*N*	% of events attributable to RSV**	RSV:influenza ratio†	Deaths/100,000 population (range)	*N*	% of events attributable to RSV**	RSV:influenza ratio†
Respiratory disease												
18-49	677 (443–850)	176534	3.3	0.6:1	4 (3–5)	1033	1.4	0.3:1	1 (0–1)	144	4.2	0.8:1
50-64	1325 (928–1542)	136469	6.1	1.1:1	30 (22–36)	3067	5.2	0.8:1	6 (5–7)	656	5.9	0.9:1
65-74	1742 (1259–2038)	86201	6.6	1.6:1	86 (62–101)	4236	5.8	0.8:1	29 (21–33)	1416	5.7	0.9:1
75+	2175 (1554–2516)	88043	7.5	1.6:1	234 (180–291)	9463	6.8	0.9:1	155 (108–213)	6266	5.9	0.7:1
Acute upper respiratory disease												
18-49	394 (261–489)	102728	3.9	0.8:1	-	-	-	-	-	-	-	-
50-64	590 (430–696)	60757	7.8	1.7:1	-	-	-	-	-	-	-	-
65-74	557 (433–701)	27580	8.4	2.5:1	-	-	-	-	-	-	-	-
75+	528 (407–659)	21372	10.1	2.4:1	-	-	-	-	-	-	-	-
Pneumonia and Influenza												
18-49	51 (17–97)	13217	5.8	0.1:1	2 (2–3)	615	5.8	0.6:1	0 (0–1)	103	5.9	0.8:1
50-64	88 (40–145)	9031	10.4	0.2:1	7 (5–9)	736	7.2	1.0:1	4 (3–5)	391	7.3	0.9:1
65-74	116 (66–167)	5755	13.6	0.4:1	20 (15–26)	987	7.3	1.0:1	17 (12–20)	821	6.7	0.8:1
75+	196 (112–279)	7944	11.2	0.6:1	80 (51–109)	3238	7.7	1.0:1	121 (86–164)	4884	7.1	0.7:1
Bronchitis/ Bronchiolitis												
18-49	229 (163–264)	59799	6.0	0.7:1	2 (1–2)	444	4.3	0.5:1	0 (0–0)	4	6.2	0.6:1
50-64	653 (458–759)	67269	9.7	1.2:1	7 (5–9)	764	8.3	0.7:1	0 (0–1)	43	17.2	1.0:1
65-74	1088 (776–1257)	53839	11.0	1.8:1	19 (14–22)	952	9.1	0.7:1	1 (1–2)	66	12.6	1.0:1
75+	1594 (1147–1858)	64540	12.3	1.8:1	75 (56–91)	3037	10.6	0.8:1	5 (3–7)	204	10.2	0.8:1
COPD												
18-49	23 (14–31)	6034	1.2	1.7:1	0 (0–0)	0	0.0	0:1	0 (0–0)	15	3.8	0.7:1
50-64	77 (49–100)	7943	2.7	2.2:1	14 (10–17)	1484	5.8	0.8:1	3 (2–4)	296	8.9	1.1:1
65-74	136 (72–206)	6734	2.7	2.9:1	45 (32–52)	2229	6.5	0.9:1	14 (9–17)	685	7.5	0.9:1
75+	82 (44–121)	3300	1.7	1.4:1	75 (55–88)	3052	7.4	1.2:1	36 (27–48)	1456	6.5	0.8:1
Cardiorespiratory												
18-49	-	-		-	1 (1–3)	376	0.4	0.1:1	1 (1–1)	213	2.8	1:1
50-64	-	-		-	18 (12–22)	1837	1.3	0.5:1	8 (6–10)	859	3.4	0.9:1
65-74	-	-		-	56 (39–67)	2780	1.6	0.6:1	37 (25–44)	1826	3.5	0.9:1
75+	-	-		-	174 (126–204)	7042	2.3	0.7:1	198 (155–259)	8028	4.1	0.8:1
Antibiotic prescriptions												
18-49	610 (442–715)	159223	2.6	0.7:1	-	-	-	-	-	-	-	-
50-64	1449 (1081–1750)	149229	5.3	1.4:1	-	-	-	-	-	-	-	-
65-74	1999 (1556–2520)	98921	6.0	2:1	-	-	-	-	-	-	-	-
75+	2261 (1809–2938)	91535	6.3	2.1:1	-	-	-	-	-	-	-	-

*N* = Seasonal number of specified RSV-attributable events in the UK

Proportion = per 100,000 population

Range = range of estimates per season

GP = general practice, COPD = chronic obstructive pulmonary disease

*Outcomes refer to the ‘Primary’ cause for hospitalisation for HES, or as the ‘Any mention’ cause of death for ONS. For the GPRD data there is no ordered listing or ranking of diagnostic codes

**% of RSV-attributable events among all events due to outcome

† ratio of proportions/100,000 RSV/[influenza A + B]

**Table 4:** Mean seasonal RSV-attributable burden of GP episodes, hospitalizations and deaths by risk group*, in individuals 65+ years of age; United Kingdom 1995-2009

	GP episodes		Hospitalisations		Deaths	
	Episodes/100,000 population (range)	*N*	Hospitalisations/100,000 population (range)	*N*	Deaths/100,000 population (range)	*N*
Respiratory disease	1946 (1400–2267)	175,070	156 (119–192)	14,039	88 (64–119)	7,915
Low risk	1335 (963–1560)	120,123	60 (46–74)	5,380	60 (41–84)	5,425
High risk	2697 (1864–3189)	242,616	265 (194–314)	23,872	121 (97–156)	10,915
Acute upper respiratory disease	544 (420–681)	48,900	-	-	-	-
Low risk	457 (349–566)	41,150	-	-	-	-
High risk	644 (492–798)	57,961	-	-	-	-
Pneumonia and Influenza	154 (88–219)	13,814	49 (32–66)	4,367	66 (47–89)	5,894
Low risk	109 (63–153)	9,807	28 (21–37)	2,559	53 (37–72)	4,729
High risk	211 (115–312)	19,025	70 (47–95)	6,322	81 (62–107)	7,307
Bronchitis/ Bronchiolitis	1328 (954–1545)	119,449	46 (34–55)	4,114	3 (2–4)	277
Low risk	849 (597–984)	76,385	30 (22–36)	2,683	1 (0–1)	75
High risk	1908 (1336–2221)	171,650	64 (46–75)	5,749	6 (4–7)	520
COPD	110 (59–166)	9,921	59 (43–70)	5,352	24 (19–30)	2,187
Low risk	0 (0–0)	2	0 (0–0)	0	0 (0–0)	0
High risk	259 (123–420)	23,306	127 (88–153)	11,464	53 (39–63)	4,807
Cardiorespiratory	-	-	112 (81–131)	10,086	113 (90–145)	10,147
Low risk	-	-	55 (43–69)	4,990	60 (40–83)	5,365
High risk	-	-	177 (117–229)	15,964	177 (129–209)	15,909

*N* = Seasonal number of specified RSV-attributable events in the UK. Note that the attributions for the full 65+ population are not equal to the sum of the attributions in 65–74 and 75+ year age groups in Table 3 due to separately run models

Rate = per 100,000 population

Range = range of estimates per season

GP = general practice, COPD = chronic obstructive pulmonary disease

*Risk group defined according to the chronic conditions indicative of risk for severe influenza as per UK recommendation for influenza vaccination. These include COPD; cardiovascular, central nervous system, renal and liver disorders; diabetes; immunosuppressive conditions or stroke

**Fig. 1:**
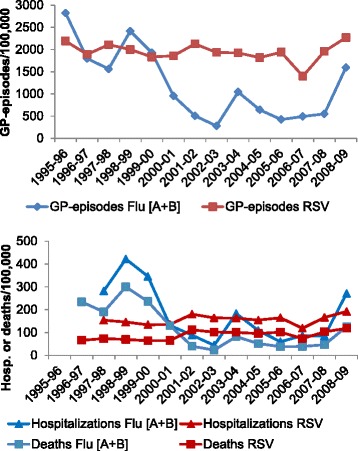
Incidence (per 100,000) of respiratory GP episodes, hospitalizations and deaths among 65+ year olds attributed to RSV or Influenza [A + B] in the seasons studied

There was marked variability between seasons in terms of the proportion of outcomes attributed to RSV versus those attributed to influenza. While the RSV burden remained rather constant from season to season, there was a general decrease in influenza-attributable outcomes after 2001 (illustrated for adults 65+ years in Fig. 1). Among 18–49 year olds, RSV caused fewer GP episodes and hospitalisations than influenza for most outcomes studied. After 2001, more deaths were attributed to RSV than influenza in this age group: average RSV/influenza ratio between 1996–1997 and 1999–2000 was 0.2 versus 1.5 thereafter. In age-group categories >50 years, more GP episodes were attributed to RSV than to influenza for all respiratory outcomes, except pneumonia and Influenza (Table 3). Seasonal proportions of hospitalisations and deaths due to RSV were close to, or equal to rates due to influenza, but exceeded influenza in most years after 2001 (Fig. 1).

**Fig. 2:**
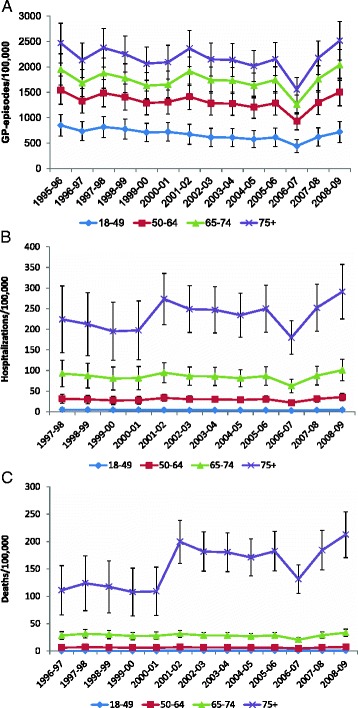
Seasonal incidence (per 100,000) of GP episodes (**a**) hospitalisations (**b**) and deaths (**c**) due to RSV-attributable respiratory disease. Vertical lines = 95 confidence intervals

Estimates of the seasonal incidence rates for GP episodes and hospitalisations for RSV-attributable respiratory disease were relatively consistent, except for a dip during the 2006 to 2007 season observed across all three databases (Fig. 2). Estimated proportions of deaths over the seasons studied were also consistent in adults, with the exception of persons aged 75+ years in whom mortality due to respiratory disease varied (albeit ≤2-fold) over the seasons studied, ranging between 108/100,000 (1999 to 2000) and 213/100,000 (2008 to 2009). Individuals at high risk of severe influenza (based on UK influenza vaccine recommendations) were more likely to have a GP episode, hospitalisation, or death due to RSV-attributable respiratory disease than individuals at low risk (Table 5). Among those aged 65+ years, the presence of a diagnosis indicative of a severe influenza risk was associated with a doubling in the proportion of RSV-attributable GP episodes and deaths for respiratory disease and a 4-fold increase in the proportion of RSV-attributable hospitalisations for respiratory disease (Table 4). Similar increases associated with risk factors were apparent for all of the studied outcomes.

**Table 5:** Mean seasonal RSV-attributable burden of respiratory disease in the United Kingdom by age and risk group*

		RSV-attributable outcome/100,000 (range)	
	Age	Low Risk Population*	High Risk Population*
GP episodes	18-49	595 (377–771)	1344 (924–1598)
	50-64	1067 (721–1295)	2221 (1581–2561)
	65-74	1239 (884–1432)	2508 (1764–2905)
	75+	1466 (1070–1732)	2860 (1948–3441)
Hospitalisations	18-49	3 (2–5)	10 (8–13)
	50-64	9 (6–10)	102 (72–117)
	65-74	20 (14–23)	180 (127–208)
	75+	116 (91–148)	338 (250–405)
Deaths	18-49	0 (0–0)	4 (3–5)
	50-64	2 (1–2)	22 (15–27)
	65-74	9 (7–11)	58 (40–69)
	75+	133 (86–189)	176 (134–233)

GP = general practice

Range = range of estimates per season

*Risk group defined according the chronic conditions indicative of risk for severe influenza as per UK recommendations for influenza vaccination. These include chronic obstructive respiratory disease; cardiovascular, central nervous system, renal and liver disorders; diabetes; immunosuppressive conditions or stroke

## Discussion

Our model estimated that among UK adults, RSV results in more GP episodes for respiratory disease than influenza, and nearly the same numbers of hospitalisations and deaths as influenza. Among persons aged 65+ years, a group in which influenza has long been recognised as a major public health threat, the burden of GP episodes for respiratory disease and the burden of respiratory hospitalisations and deaths in most seasons since 2001 was higher for RSV than for influenza. These data confirm earlier speculations that viruses other than influenza contribute substantially to winter deaths in older adults [3].

Identified risk factors for severe influenza disease also appeared to reflect the risk of severe RSV disease in the elderly. The clinical presentations associated with RSV were diverse, manifesting as bronchitis/bronchiolitis, as well as exacerbations of chronic obstructive pulmonary disease and cardiorespiratory diseases.

A dip in RSV-associated respiratory outcomes was observed in the 2006 to 2007 season that was apparent in GP episodes, hospital admissions and deaths. In this season there was a particularly low number of RSV reports to PHE – roughly half those reported in the previous winter. It was also a season of relatively low and late incidence of influenza-like illness [18]. This decrease was also evident in another study of mortality in the UK that also used ONS data [19]. No explanation is easily forthcoming to explain this isolated, but consistently observed decrease.

Direct measurement of the morbidity and mortality burden of respiratory pathogens in adults is particularly challenging because hospitalisations and deaths associated with respiratory viruses usually occur some days or weeks after the initial infection, by which time the virus may no longer be detectable. Older patients may present with secondary infections or with exacerbations of underlying chronic conditions, and these patients may not be tested for the causal viral infection. We used a multiple linear regression model as previously described [20–22], because it assumes that incidence rates increase linearly with increases in viral circulation and that the model terms have an additive effect on burden. Other models, such as Poisson regression, have been used to assess the burden of seasonal respiratory viruses [12, 23]. However, this model implies that the effect of two (or more) simultaneously circulating respiratory viruses is different from, and often larger than, the sum of their individual effects. In the case of RSV, it is hard to envisage a synergistic effect with co-circulating influenza strains. If anything, co-circulation could be associated with a reduction in burden due to competitive exclusion [24–26].

Previous evidence suggests that winter increases in the incidence of RSV-associated outcomes (e.g., acute bronchitis) among adults follows those in children by 2–4 weeks [27]. Most laboratory tests for RSV are performed on samples from children. Therefore, in the absence of hard data regarding RSV infection in older persons, we hypothesised that the efficacy of the lagged variable in the regression models reflects an actual sequential pattern of infection across age groups, as previously suggested [27]. A longer lag time was used for hospitalisations and deaths (three-week lag) than for office visits (two-week lag), to allow for the delay between illness onset and the development of complications which might prompt admission.

We used a seasonal (sine/cosine pair) adjustment in the model to control for a number of seasonal confounders for which data are not available. Because RSV infections in the UK and other temperate climates are also characterised by a regular seasonal cycle, the rise in RSV-associated illnesses overlaps to some extent with the cyclic terms. The attribution of burden to RSV was determined after adjusting for the cyclic terms. These terms thus reduce both the portion of all outcomes attributed to RSV and the risk that the RSV burden is overestimated. Given the very nature of observational/ecological studies, there is not currently a technique that can satisfactorily ensure temporal and spatial independence in the structure of the time-series data, and simultaneously yield biologically meaningful estimates of disease burden. Therefore, this is a limitation of most studies on disease burden that make use of epidemiological time-series.

Our study is thus potentially limited by the assumption of a constant seasonal baseline and a sinusoidal pattern of seasonal outcomes. Furthermore, patients who did not seek care and changes in coding practices or in the quality of diagnoses recorded in the databases over time could not be accounted for in our model. However, the expected absence of attributions for the negative outcome control (urinary tract infections and accidents) provides support for the model. Finally, the secular component of the model was highly collinear with RSV seasonality, ‘stealing’ the burden of RSV more than the burden of influenza. By including cyclic terms in the model we used a conservative approach that reduced the possibility that the study overestimated the burden of RSV.

The strengths of our study include the availability of individual patient data for defined outcomes including co-morbid risk status, the use of large, nationally representative databases which reduced the risk of sampling error, the extended study period which included 14 seasons, and the use of a relatively broad definition of respiratory disease, which aimed to improve the sensitivity in capturing the full RSV-attributable disease burden by including respiratory symptom codes used by general practitioners, while retaining specificity. Finally, we report novel data on antibiotic prescribing attributable to RSV. Our model estimated that 912,036 antibiotic prescriptions were attributable to RSV-respiratory disease in an average season, suggesting substantial inappropriate antibiotic prescribing. Until the availability of an affordable and accessible diagnostic test capable to differentiating between viral and bacterial infections, this situation is unlikely to change.

Our estimates of the burden of RSV-attributable hospitalisations among persons aged 65+ years in the UK (156/100,000) falls within the range of two modelled burden studies reported for the US by Zhou et al. (86/100,000) [28] and Widmer et al. (254/100,000) [29]. Our finding of a similar RSV and influenza burden among the elderly is in agreement with US studies [5, 29], but not with a UK study of influenza and RSV mortality (1999 to 2010) by Hardelid et al. in which the burden of RSV deaths (using ONS data) was estimated to be around one-half that of influenza [19]. Hardelid et al’s study was based on modelling which included lagged virology counts as variables. It was concerned exclusively with mortality and the final selected model was based on the best fit in persons 75+ years where the 4305 estimated attributable deaths (all causes) compares with our estimated 6266 respiratory deaths. In our study the impact of RSV on mortality was closer to that of influenza than that reported by Hardelid et al. for respiratory deaths [19]. We estimated the RSV-attributable burden of respiratory outcomes which were known to be associated with RSV infection, rather than all-cause mortality: our estimate of cardiorespiratory mortality was 28 % higher than that for respiratory disease. Our findings are in good agreement with those published by Pitman and colleagues, who used a similar multivariate linear regression model to analyse pre-2005 UK data, with the GP data limited to the 2002–2003 season and estimates of RSV attributable burden limited to overall respiratory disease [11]. A modelling study in the Netherlands that assessed nine common pathogens among elderly for which robust surveillance data were available [30], found that influenza A and RSV were associated with similar prevalence among all-cause deaths (RSV 1.4 % and influenza A 1.5 % of all deaths).

## Conclusion

Our study indicates that the burden of RSV in the primary care setting is higher than that of influenza. It also suggests that hospitalisations and deaths attributable to RSV in older adults approach, or in most seasons since 2001, exceed those from influenza; unlike influenza, however, there is little variation in the burden from season to season. Seasonal increases in hospitalisation and deaths increased in a predictable manner, two to three weeks after the onset of epidemic RSV in children. This information could be used to guide public health activities and policy related to prevention and treatment. To date, specific prevention and treatment of RSV for adults is not available. The development of treatment options and preventative measures warrant further investigation, particularly for those at high risk of influenza.

## Additional file

Additional file 1:
**CPRD Coding Definitions.** (DOCX 512 kb)

## Abbreviations

CPRD: Clinical Practice Research Datalink
HES: Hospital Episode Statistics
ICD: International Classification of Diseases
GP: General practice
ONS: Office of National Statistics
PHE: Public Health England
RSV: Respiratory syncytial virus
UK: United Kingdom
US: United States
