# Supplementary material for: Modelling estimates of the burden of Respiratory Syncytial virus infection in adults and the elderly in the United Kingdom
Source: BMC Infect Dis. 2015 Oct 23;15:443. doi: 10.1186/s12879-015-1218-z (PMC4618996; doi:10.1186/s12879-015-1218-z)
Supplement: Additional file 1: — CPRD Coding Definitions. (DOCX 512 kb) [file 12879_2015_1218_MOESM1_ESM.docx]

**Additional file 1: CPRD Coding Definitions**

Oxford Medical Information System (OXMIS) and Read codes are clinical terms used by the UK National Health Service including GPs to describe patient care and treatment. Coding is predominantly made using the READ coding system. The custodians of the CPRD maintain a one to one mapping from these codes to a numeric code, of smaller byte size, called the medcode. The data released to researchers has OXMIS and Read codes recorded by GPs translated to their equivalent medcodes.

## Coding Definitions Diagnosis

[Coding Definitions Diagnosis 1](#_Toc412108204)

[1.1. risk_factor definition 1: Chronic heart disease 2](#_Toc412108205)

[1.2. risk_factor definition 2: Renal disease 34](#_Toc412108206)

[1.3. risk_factor definition 3: Diabetes_diagnosis 40](#_Toc412108207)

[1.4. risk_factor definition 4: Diabetes_monitoring 53](#_Toc412108208)

[1.5. risk_factor definition 5: Liver disease 57](#_Toc412108209)

[1.6. risk_factor definition 6: Stroke_TIA 61](#_Toc412108210)

[1.7. risk_factor definition 7: Central nervous sytem disease 65](#_Toc412108211)

[1.8. risk_factor definition 8: COPD (not asthma) 77](#_Toc412108212)

[1.9. risk_factor definition 9: Immunosuppressive conditions 85](#_Toc412108213)

[1.10. outcome definition 1: All respiratory diagnoses_broad 97](#_Toc412108214)

[1.11. outcome definition 2: Pneumonia and Influenza 138](#_Toc412108215)

[1.12. outcome definition 3: Acute upper respiratory 142](#_Toc412108216)

[1.13. outcome definition 4: Bronchitis Bronchiolitis 146](#_Toc412108217)

[1.14. outcome definition 5: Chronic respiratory including COPD_asthma 148](#_Toc412108218)

[1.15. outcome definition 6: Urinary_Tract_Infection 152](#_Toc412108219)

[Coding Definitions Treatment 153](#_Toc412108220)

[1.16. risk_factor definition 1: Immuno_suppressant Rx 153](#_Toc412108221)

[1.17. outcome definition 94: Any broad spectrum penicillin macrolide tetracycline 189](#_Toc412108222)

| risk_factor definition 1: Chronic heart disease |
| --- |
| n= 835 medcodes used |

| **Read_code** | **readoxmisflag** | **medcode** | **desc** |  |
| --- | --- | --- | --- | --- |
| G1...00 | READ | 9312 | Chronic rheumatic heart disease |  |
| G10..00 | READ | 44376 | Chronic rheumatic pericarditis |  |
| G100.00 | READ | 40957 | Adherent rheumatic pericardium |  |
| G102.00 | READ | 72628 | Chronic rheumatic myopericarditis |  |
| G11..00 | READ | 1267 | Mitral valve diseases |  |
| G11..11 | READ | 16545 | Rheumatic mitral valve disease |  |
| G110.00 | READ | 1885 | Mitral stenosis |  |
| G110.11 | READ | 32435 | Rheumatic mitral stenosis |  |
| G111.00 | READ | 51879 | Rheumatic mitral insufficiency |  |
| G111.11 | READ | 21807 | Mitral incompetence - rheumatic |  |
| G111.12 | READ | 22837 | Mitral regurgitation - rheumatic |  |
| G112.00 | READ | 44488 | Mitral stenosis with insufficiency |  |
| G112.12 | READ | 50983 | Mitral stenosis with incompetence |  |
| G112.13 | READ | 44328 | Mitral stenosis with regurgitation |  |
| G113.00 | READ | 28662 | Nonrheumatic mitral valve stenosis |  |
| G114.00 | READ | 57633 | Ruptured mitral valve cusp |  |
| G11z.00 | READ | 30443 | Mitral valve disease NOS |  |
| G12..00 | READ | 18100 | Rheumatic aortic valve disease |  |
| G120.00 | READ | 9391 | Rheumatic aortic stenosis |  |
| G121.00 | READ | 32211 | Rheumatic aortic insufficiency |  |
| G121.11 | READ | 43347 | Aortic incompetence - rheumatic |  |
| G121.12 | READ | 7963 | Aortic regurgitation - rheumatic |  |
| G122.00 | READ | 63960 | Rheumatic aortic stenosis with insufficiency |  |
| G12z.00 | READ | 50809 | Rheumatic aortic valve disease NOS |  |
| G13..00 | READ | 10078 | Diseases of mitral and aortic valves |  |
| G130.00 | READ | 8274 | Mitral and aortic stenosis |  |
| G131.00 | READ | 49355 | Mitral stenosis and aortic insufficiency |  |
| G131.13 | READ | 61250 | Mitral stenosis and aortic incompetence |  |
| G131.14 | READ | 17596 | Mitral stenosis and aortic regurgitation |  |
| G132.00 | READ | 33262 | Mitral insufficiency and aortic stenosis |  |
| G132.12 | READ | 31759 | Mitral incompetence and aortic stenosis |  |
| G132.13 | READ | 33907 | Mitral regurgitation and aortic stenosis |  |
| G133.00 | READ | 31727 | Mitral and aortic incompetence |  |
| G133.11 | READ | 94872 | Mitral and aortic insufficiency |  |
| G133.12 | READ | 11878 | Mitral and aortic regurgitation |  |
| G13y.00 | READ | 70698 | Multiple mitral and aortic valve involvement |  |
| G13z.00 | READ | 29158 | Mitral and aortic valve disease NOS |  |
| G14..00 | READ | 68126 | Other chronic rheumatic endocardial disease |  |
| G140.00 | READ | 16373 | Tricuspid valve disease NEC |  |
| G140000 | READ | 31505 | Rheumatic tricuspid stenosis |  |
| G140100 | READ | 60266 | Rheumatic tricuspid insufficiency |  |
| G140111 | READ | 21980 | Tricuspid regurgitation - rheumatic |  |
| G140112 | READ | 42239 | Tricuspid incompetence - rheumatic |  |
| G140200 | READ | 93114 | Rheumatic tricuspid stenosis and insufficiency |  |
| G14021X | READ | 93113 | Rheumatic tricuspid stenosis and regurgitation |  |
| G14021Y | READ | 62186 | Rheumatic tricuspid stenosis and incompetence |  |
| G140300 | READ | 56029 | Tricuspid stenosis, cause unspecified |  |
| G140400 | READ | 42128 | Tricuspid insufficiency, cause unspecified |  |
| G140412 | READ | 34869 | Tricuspid incompetence, cause unspecified |  |
| G140413 | READ | 9286 | Tricuspid regurgitation, cause unspecified |  |
| G140500 | READ | 72306 | Tricuspid stenosis and insufficiency, cause unspecified |  |
| G140514 | READ | 49551 | Tricuspid stenosis and regurgitation, cause unspecified |  |
| G140z00 | READ | 72613 | Rheumatic tricuspid valve disease NOS |  |
| G141.00 | READ | 44167 | Rheumatic pulmonary valve disease |  |
| G141000 | READ | 62207 | Rheumatic pulmonary stenosis |  |
| G141100 | READ | 54088 | Rheumatic pulmonary insufficiency |  |
| G141z00 | READ | 36768 | Rheumatic pulmonary valve disease NOS |  |
| G14z.00 | READ | 15132 | Rheumatic endocarditis NOS |  |
| G14z.11 | READ | 59275 | Rheumatic valvulitis, chronic NOS |  |
| G1y..00 | READ | 15643 | Other specified chronic rheumatic heart disease |  |
| G1y0.00 | READ | 62404 | Rheumatic myocarditis |  |
| G1yz.00 | READ | 57980 | Other and unspecified rheumatic heart disease |  |
| G1yz000 | READ | 53878 | Rheumatic heart disease unspecified |  |
| G1yz100 | READ | 22262 | Rheumatic left ventricular failure |  |
| G1yzz00 | READ | 59854 | Other rheumatic heart disease NOS |  |
| G1z..00 | READ | 20001 | Chronic rheumatic heart disease NOS |  |
| G21..00 | READ | 16292 | Hypertensive heart disease |  |
| G210.00 | READ | 50157 | Malignant hypertensive heart disease |  |
| G210000 | READ | 95334 | Malignant hypertensive heart disease without CCF |  |
| G210100 | READ | 72668 | Malignant hypertensive heart disease with CCF |  |
| G211.00 | READ | 52427 | Benign hypertensive heart disease |  |
| G211000 | READ | 61660 | Benign hypertensive heart disease without CCF |  |
| G211100 | READ | 52127 | Benign hypertensive heart disease with CCF |  |
| G21z.00 | READ | 31464 | Hypertensive heart disease NOS |  |
| G21z000 | READ | 61166 | Hypertensive heart disease NOS without CCF |  |
| G21z011 | READ | 8857 | Cardiomegaly - hypertensive |  |
| G21z100 | READ | 62718 | Hypertensive heart disease NOS with CCF |  |
| G21zz00 | READ | 16173 | Hypertensive heart disease NOS |  |
| G22..00 | READ | 4668 | Hypertensive renal disease |  |
| G22..11 | READ | 17434 | Nephrosclerosis |  |
| G220.00 | READ | 39649 | Malignant hypertensive renal disease |  |
| G221.00 | READ | 43935 | Benign hypertensive renal disease |  |
| G222.00 | READ | 32423 | Hypertensive renal disease with renal failure |  |
| G22z.00 | READ | 15106 | Hypertensive renal disease NOS |  |
| G22z.11 | READ | 29310 | Renal hypertension |  |
| G23..00 | READ | 63466 | Hypertensive heart and renal disease |  |
| G230.00 | READ | 67232 | Malignant hypertensive heart and renal disease |  |
| G231.00 | READ | 63000 | Benign hypertensive heart and renal disease |  |
| G232.00 | READ | 21837 | Hypertensive heart&renal dis wth (congestive) heart failure |  |
| G233.00 | READ | 28684 | Hypertensive heart and renal disease with renal failure |  |
| G234.00 | READ | 57987 | Hyperten heart&renal dis+both(congestv)heart and renal fail |  |
| G23z.00 | READ | 68659 | Hypertensive heart and renal disease NOS |  |
| G24..00 | READ | 7329 | Secondary hypertension |  |
| G240.00 | READ | 31755 | Secondary malignant hypertension |  |
| G240000 | READ | 59383 | Secondary malignant renovascular hypertension |  |
| G240z00 | READ | 73293 | Secondary malignant hypertension NOS |  |
| G241.00 | READ | 57288 | Secondary benign hypertension |  |
| G241000 | READ | 25371 | Secondary benign renovascular hypertension |  |
| G241z00 | READ | 51635 | Secondary benign hypertension NOS |  |
| G244.00 | READ | 34744 | Hypertension secondary to endocrine disorders |  |
| G24z.00 | READ | 16059 | Secondary hypertension NOS |  |
| G24z000 | READ | 31387 | Secondary renovascular hypertension NOS |  |
| G24z100 | READ | 31341 | Hypertension secondary to drug |  |
| G24zz00 | READ | 42229 | Secondary hypertension NOS |  |
| G2y..00 | READ | 18765 | Other specified hypertensive disease |  |
| G3...00 | READ | 240 | Ischaemic heart disease |  |
| G3...11 | READ | 24783 | Arteriosclerotic heart disease |  |
| G3...12 | READ | 20416 | Atherosclerotic heart disease |  |
| G3...13 | READ | 1792 | IHD - Ischaemic heart disease |  |
| G30..00 | READ | 241 | Acute myocardial infarction |  |
| G30..11 | READ | 13566 | Attack - heart |  |
| G30..12 | READ | 2491 | Coronary thrombosis |  |
| G30..13 | READ | 30421 | Cardiac rupture following myocardial infarction (MI) |  |
| G30..14 | READ | 1204 | Heart attack |  |
| G30..15 | READ | 1677 | MI - acute myocardial infarction |  |
| G30..16 | READ | 13571 | Thrombosis - coronary |  |
| G30..17 | READ | 17689 | Silent myocardial infarction |  |
| G300.00 | READ | 12139 | Acute anterolateral infarction |  |
| G301.00 | READ | 5387 | Other specified anterior myocardial infarction |  |
| G301000 | READ | 40429 | Acute anteroapical infarction |  |
| G301100 | READ | 17872 | Acute anteroseptal infarction |  |
| G301z00 | READ | 14897 | Anterior myocardial infarction NOS |  |
| G302.00 | READ | 8935 | Acute inferolateral infarction |  |
| G303.00 | READ | 29643 | Acute inferoposterior infarction |  |
| G304.00 | READ | 23892 | Posterior myocardial infarction NOS |  |
| G305.00 | READ | 14898 | Lateral myocardial infarction NOS |  |
| G306.00 | READ | 63467 | True posterior myocardial infarction |  |
| G307.00 | READ | 3704 | Acute subendocardial infarction |  |
| G307000 | READ | 9507 | Acute non-Q wave infarction |  |
| G307100 | READ | 10562 | Acute non-ST segment elevation myocardial infarction |  |
| G308.00 | READ | 1678 | Inferior myocardial infarction NOS |  |
| G309.00 | READ | 30330 | Acute Q-wave infarct |  |
| G30A.00 | READ | 17133 | Mural thrombosis |  |
| G30B.00 | READ | 32854 | Acute posterolateral myocardial infarction |  |
| G30X.00 | READ | 29758 | Acute transmural myocardial infarction of unspecif site |  |
| G30X000 | READ | 12229 | Acute ST segment elevation myocardial infarction |  |
| G30y.00 | READ | 34803 | Other acute myocardial infarction |  |
| G30y000 | READ | 28736 | Acute atrial infarction |  |
| G30y100 | READ | 62626 | Acute papillary muscle infarction |  |
| G30y200 | READ | 41221 | Acute septal infarction |  |
| G30yz00 | READ | 46017 | Other acute myocardial infarction NOS |  |
| G30z.00 | READ | 14658 | Acute myocardial infarction NOS |  |
| G31..00 | READ | 27951 | Other acute and subacute ischaemic heart disease |  |
| G310.00 | READ | 23579 | Postmyocardial infarction syndrome |  |
| G310.11 | READ | 15661 | Dressler's syndrome |  |
| G311.00 | READ | 36523 | Preinfarction syndrome |  |
| G311.11 | READ | 4656 | Crescendo angina |  |
| G311.12 | READ | 39655 | Impending infarction |  |
| G311.13 | READ | 1431 | Unstable angina |  |
| G311.14 | READ | 19655 | Angina at rest |  |
| G311000 | READ | 61072 | Myocardial infarction aborted |  |
| G311011 | READ | 55137 | MI - myocardial infarction aborted |  |
| G311100 | READ | 7347 | Unstable angina |  |
| G311200 | READ | 17307 | Angina at rest |  |
| G311300 | READ | 34328 | Refractory angina |  |
| G311400 | READ | 18118 | Worsening angina |  |
| G311500 | READ | 11983 | Acute coronary syndrome |  |
| G311z00 | READ | 54251 | Preinfarction syndrome NOS |  |
| G312.00 | READ | 39449 | Coronary thrombosis not resulting in myocardial infarction |  |
| G31y.00 | READ | 9413 | Other acute and subacute ischaemic heart disease |  |
| G31y000 | READ | 9276 | Acute coronary insufficiency |  |
| G31y100 | READ | 68357 | Microinfarction of heart |  |
| G31y200 | READ | 39693 | Subendocardial ischaemia |  |
| G31y300 | READ | 21844 | Transient myocardial ischaemia |  |
| G31yz00 | READ | 27977 | Other acute and subacute ischaemic heart disease NOS |  |
| G32..00 | READ | 4017 | Old myocardial infarction |  |
| G32..11 | READ | 16408 | Healed myocardial infarction |  |
| G32..12 | READ | 17464 | Personal history of myocardial infarction |  |
| G33..00 | READ | 1430 | Angina pectoris |  |
| G330.00 | READ | 20095 | Angina decubitus |  |
| G330000 | READ | 18125 | Nocturnal angina |  |
| G330z00 | READ | 29902 | Angina decubitus NOS |  |
| G331.00 | READ | 12986 | Prinzmetal's angina |  |
| G331.11 | READ | 11048 | Variant angina pectoris |  |
| G332.00 | READ | 36854 | Coronary artery spasm |  |
| G33z.00 | READ | 25842 | Angina pectoris NOS |  |
| G33z000 | READ | 66388 | Status anginosus |  |
| G33z100 | READ | 54535 | Stenocardia |  |
| G33z200 | READ | 7696 | Syncope anginosa |  |
| G33z300 | READ | 1414 | Angina on effort |  |
| G33z400 | READ | 32450 | Ischaemic chest pain |  |
| G33z500 | READ | 9555 | Post infarct angina |  |
| G33z600 | READ | 26863 | New onset angina |  |
| G33z700 | READ | 12804 | Stable angina |  |
| G33zz00 | READ | 28554 | Angina pectoris NOS |  |
| G34..00 | READ | 28138 | Other chronic ischaemic heart disease |  |
| G340.00 | READ | 5413 | Coronary atherosclerosis |  |
| G340.11 | READ | 1655 | Triple vessel disease of the heart |  |
| G340.12 | READ | 1344 | Coronary artery disease |  |
| G340000 | READ | 3999 | Single coronary vessel disease |  |
| G340100 | READ | 5254 | Double coronary vessel disease |  |
| G341.00 | READ | 6331 | Aneurysm of heart |  |
| G341.11 | READ | 27484 | Cardiac aneurysm |  |
| G341000 | READ | 2155 | Ventricular cardiac aneurysm |  |
| G341100 | READ | 67087 | Other cardiac wall aneurysm |  |
| G341200 | READ | 59193 | Aneurysm of coronary vessels |  |
| G341300 | READ | 91774 | Acquired atrioventricular fistula of heart |  |
| G341z00 | READ | 41677 | Aneurysm of heart NOS |  |
| G342.00 | READ | 36609 | Atherosclerotic cardiovascular disease |  |
| G343.00 | READ | 7320 | Ischaemic cardiomyopathy |  |
| G344.00 | READ | 29421 | Silent myocardial ischaemia |  |
| G34y.00 | READ | 34633 | Other specified chronic ischaemic heart disease |  |
| G34y000 | READ | 24540 | Chronic coronary insufficiency |  |
| G34y100 | READ | 23078 | Chronic myocardial ischaemia |  |
| G34yz00 | READ | 35713 | Other specified chronic ischaemic heart disease NOS |  |
| G34z.00 | READ | 15754 | Other chronic ischaemic heart disease NOS |  |
| G34z000 | READ | 18889 | Asymptomatic coronary heart disease |  |
| G35..00 | READ | 18842 | Subsequent myocardial infarction |  |
| G350.00 | READ | 45809 | Subsequent myocardial infarction of anterior wall |  |
| G351.00 | READ | 38609 | Subsequent myocardial infarction of inferior wall |  |
| G353.00 | READ | 72562 | Subsequent myocardial infarction of other sites |  |
| G35X.00 | READ | 46166 | Subsequent myocardial infarction of unspecified site |  |
| G36..00 | READ | 36423 | Certain current complication follow acute myocardial infarct |  |
| G360.00 | READ | 24126 | Haemopericardium/current comp folow acut myocard infarct |  |
| G361.00 | READ | 23708 | Atrial septal defect/curr comp folow acut myocardal infarct |  |
| G362.00 | READ | 37657 | Ventric septal defect/curr comp fol acut myocardal infarctn |  |
| G363.00 | READ | 59189 | Ruptur cardiac wall w'out haemopericard/cur comp fol ac MI |  |
| G364.00 | READ | 59940 | Ruptur chordae tendinae/curr comp fol acute myocard infarct |  |
| G365.00 | READ | 69474 | Rupture papillary muscle/curr comp fol acute myocard infarct |  |
| G366.00 | READ | 29553 | Thrombosis atrium,auric append&vent/curr comp foll acute MI |  |
| G37..00 | READ | 8568 | Cardiac syndrome X |  |
| G38..00 | READ | 32272 | Postoperative myocardial infarction |  |
| G380.00 | READ | 46112 | Postoperative transmural myocardial infarction anterior wall |  |
| G381.00 | READ | 46276 | Postoperative transmural myocardial infarction inferior wall |  |
| G384.00 | READ | 41835 | Postoperative subendocardial myocardial infarction |  |
| G38z.00 | READ | 68748 | Postoperative myocardial infarction, unspecified |  |
| G3y..00 | READ | 22383 | Other specified ischaemic heart disease |  |
| G3z..00 | READ | 1676 | Ischaemic heart disease NOS |  |
| G4...00 | READ | 7180 | Pulmonary circulation diseases |  |
| G4...11 | READ | 22412 | Heart disease - pulmonary |  |
| G40..00 | READ | 63217 | Acute pulmonary heart disease |  |
| G400.00 | READ | 8464 | Acute cor pulmonale |  |
| G401.00 | READ | 1266 | Pulmonary embolism |  |
| G401.11 | READ | 24444 | Infarction - pulmonary |  |
| G401.12 | READ | 9701 | Pulmonary embolus |  |
| G401000 | READ | 18121 | Post operative pulmonary embolus |  |
| G402.00 | READ | 4717 | Pulmonary infarct |  |
| G40z.00 | READ | 65533 | Acute pulmonary heart disease NOS |  |
| G41..00 | READ | 46294 | Chronic pulmonary heart disease |  |
| G410.00 | READ | 245 | Primary pulmonary hypertension |  |
| G411.00 | READ | 42901 | Kyphoscoliotic heart disease |  |
| G41y.00 | READ | 54113 | Other chronic pulmonary heart disease |  |
| G41y000 | READ | 34065 | Secondary pulmonary hypertension |  |
| G41yz00 | READ | 71046 | Other chronic pulmonary heart disease NOS |  |
| G41z.00 | READ | 15782 | Chronic pulmonary heart disease NOS |  |
| G41z.11 | READ | 5695 | Chronic cor pulmonale |  |
| G42..00 | READ | 24549 | Other pulmonary circulation disease |  |
| G42..11 | READ | 31883 | Pulmonary vessel disease |  |
| G420.00 | READ | 53377 | Arteriovenous fistula of pulmonary vessels |  |
| G421.00 | READ | 22781 | Aneurysm of pulmonary artery |  |
| G42y.00 | READ | 65954 | Other specified pulmonary circulation disease |  |
| G42y000 | READ | 37807 | Pulmonary arteritis |  |
| G42y100 | READ | 51540 | Pulmonary vessel rupture |  |
| G42yz00 | READ | 41728 | Other specified pulmonary circulation disease NOS |  |
| G42z.00 | READ | 73599 | Other pulmonary circulation disease NOS |  |
| G4y..00 | READ | 61138 | Other specified pulmonary circulation disease |  |
| G4z..00 | READ | 16084 | Pulmonary circulation disease NOS |  |
| G5...00 | READ | 30171 | Other forms of heart disease |  |
| G50..00 | READ | 3399 | Acute pericarditis |  |
| G50..11 | READ | 45311 | Pericardial effusion - acute |  |
| G500.00 | READ | 29551 | Acute pericarditis in diseases EC |  |
| G500000 | READ | 58769 | Acute pericarditis - coxsackie |  |
| G500100 | READ | 61379 | Acute pericarditis - meningococcal |  |
| G500300 | READ | 57126 | Acute pericarditis - tuberculous |  |
| G500311 | READ | 16996 | TB - acute pericarditis |  |
| G500400 | READ | 40956 | Acute pericarditis - uraemic |  |
| G500z00 | READ | 15089 | Acute pericarditis in diseases EC NOS |  |
| G501.00 | READ | 35119 | Post infarction pericarditis |  |
| G50z.00 | READ | 14646 | Other and unspecified acute pericarditis |  |
| G50z000 | READ | 27606 | Acute pericarditis - unspecified |  |
| G50z100 | READ | 59102 | Acute idiopathic pericarditis |  |
| G50z111 | READ | 8411 | Viral pericarditis NOS |  |
| G50z200 | READ | 36496 | Acute pericarditis - pneumococcal |  |
| G50z400 | READ | 59677 | Acute pericarditis - streptococcal |  |
| G50z500 | READ | 64481 | Acute purulent pericarditis unspecified |  |
| G50z511 | READ | 60411 | Pyopericardium |  |
| G50zz00 | READ | 36755 | Acute pericarditis NOS |  |
| G51..00 | READ | 12775 | Acute and subacute endocarditis |  |
| G510.00 | READ | 25617 | Acute and subacute bacterial endocarditis |  |
| G510.11 | READ | 4939 | Bacterial endocarditis |  |
| G510000 | READ | 31308 | Acute bacterial endocarditis |  |
| G510100 | READ | 5449 | Subacute bacterial endocarditis - SBE |  |
| G510200 | READ | 40569 | Chronic bacterial endocarditis |  |
| G510z00 | READ | 45174 | Acute and subacute bacterial endocarditis NOS |  |
| G511.00 | READ | 48340 | Acute and subacute infective endocarditis in diseases EC |  |
| G511100 | READ | 69593 | Endocarditis - coxsackie |  |
| G511200 | READ | 100572 | Endocarditis - gonococcal |  |
| G511400 | READ | 62494 | Endocarditis - typhoid |  |
| G511600 | READ | 67780 | Endocarditis - Q fever |  |
| G511z00 | READ | 27843 | Infective endocarditis in diseases EC, NOS |  |
| G51z.00 | READ | 38876 | Acute and subacute endocarditis unspecified |  |
| G51z000 | READ | 34290 | Acute endocarditis NOS |  |
| G51z100 | READ | 100924 | Acute myoendocarditis NOS |  |
| G51z300 | READ | 48024 | Subacute endocarditis NOS |  |
| G51zz00 | READ | 66121 | Acute and subacute endocarditis unspecified, NOS |  |
| G52..00 | READ | 22639 | Acute myocarditis |  |
| G520100 | READ | 53518 | Acute myocarditis - coxsackie |  |
| G520300 | READ | 55646 | Acute myocarditis - influenzal |  |
| G520500 | READ | 67291 | Acute myocarditis - toxoplasmosis |  |
| G520600 | READ | 91847 | Acute myocarditis - tuberculous |  |
| G520700 | READ | 68411 | Acute myocarditis - meningococcal |  |
| G520z00 | READ | 62736 | Acute myocarditis in diseases EC, NOS |  |
| G52y.00 | READ | 72110 | Other acute myocarditis |  |
| G52y000 | READ | 61492 | Acute myocarditis, unspecified |  |
| G52y111 | READ | 98167 | Giant cell myocarditis |  |
| G52y200 | READ | 72409 | Idiopathic myocarditis NOS |  |
| G52y600 | READ | 71848 | Septic myocarditis NOS |  |
| G52y700 | READ | 55416 | Toxic myocarditis |  |
| G52yz00 | READ | 63078 | Other acute myocarditis NOS |  |
| G52z.00 | READ | 41527 | Acute myocarditis NOS |  |
| G53..00 | READ | 40427 | Other diseases of pericardium |  |
| G530.00 | READ | 15534 | Haemopericardium |  |
| G531.00 | READ | 50720 | Adhesive pericarditis |  |
| G531000 | READ | 41163 |  |  |
| G531100 | READ | 63675 | Fibrosis of pericardium |  |
| G531z00 | READ | 96101 | Adhesive pericarditis NOS |  |
| G532.00 | READ | 20157 | Constrictive pericarditis |  |
| G532000 | READ | 56180 | Concato's disease |  |
| G532100 | READ | 59140 | Pick's disease of heart |  |
| G532z00 | READ | 65807 | Constrictive pericarditis NOS |  |
| G533.00 | READ | 18293 | Pericardial effusion - noninflammatory |  |
| G534.00 | READ | 2520 | Pericardial effusion - acute |  |
| G53y.00 | READ | 57916 | Other diseases of pericardium OS |  |
| G53y000 | READ | 37628 | Calcification of pericardium |  |
| G53y100 | READ | 61929 | Fistula of pericardium |  |
| G53yz00 | READ | 42024 | Other specified pericardial disease NOS |  |
| G53yz11 | READ | 18877 | Chronic pericarditis |  |
| G53z.00 | READ | 33370 | Other pericardial disease NOS |  |
| G53z.11 | READ | 15792 | Cardiac tamponade |  |
| G53z000 | READ | 38817 | Non-traumatic pneumopericardium |  |
| G54..00 | READ | 17146 | Other diseases of endocardium |  |
| G54..11 | READ | 28850 | Heart valve disorders - non rheumatic |  |
| G540.00 | READ | 2977 | Mitral valve incompetence |  |
| G540.12 | READ | 40949 | Mitral valve insufficiency |  |
| G540.14 | READ | 9450 | Mitral valve regurgitation |  |
| G540.15 | READ | 1294 | Mitral valve prolapse |  |
| G540.16 | READ | 561 | Mitral regurgitation |  |
| G540000 | READ | 5058 | Mitral incompetence, non-rheumatic |  |
| G540100 | READ | 34240 | Mitral incompetence, cause unspecified |  |
| G540200 | READ | 31839 | Mitral valve prolapse |  |
| G540300 | READ | 39916 | Mitral valve leaf prolapse |  |
| G540z00 | READ | 24557 | Mitral valve disorders NOS |  |
| G541.00 | READ | 4548 | Aortic valve disorders |  |
| G541000 | READ | 14998 | Aortic incompetence, non-rheumatic |  |
| G541011 | READ | 47887 | Aortic insufficiency, non-rheumatic |  |
| G541012 | READ | 10187 | Aortic regurgitation, non-rheumatic |  |
| G541100 | READ | 999 | Aortic stenosis, non-rheumatic |  |
| G541200 | READ | 1007 | Aortic incompetence alone, cause unspecified |  |
| G541211 | READ | 58810 | Aortic insufficiency alone, cause unspecified |  |
| G541212 | READ | 1005 | Aortic regurgitation alone, cause unspecified |  |
| G541300 | READ | 2343 | Aortic stenosis alone, cause unspecified |  |
| G541400 | READ | 10964 | Aortic valve stenosis with insufficiency |  |
| G541500 | READ | 9591 | Aortic stenosis |  |
| G541600 | READ | 30610 | Aortic valve sclerosis |  |
| G541700 | READ | 49185 | Aortic valve calcification |  |
| G541z00 | READ | 19019 | Aortic valve disorders NOS |  |
| G542.00 | READ | 2817 | Tricuspid valve disorders, non-rheumatic |  |
| G542000 | READ | 1779 | Tricuspid incompetence, non-rheumatic |  |
| G542011 | READ | 97738 | Tricuspid insufficiency, non-rheumatic |  |
| G542012 | READ | 35372 | Tricuspid regurgitation, non-rheumatic |  |
| G542100 | READ | 35724 | Tricuspid stenosis, non-rheumatic |  |
| G542200 | READ | 52271 | Nonrheumatic tricuspid valve stenosis with insufficiency |  |
| G542X00 | READ | 98538 | Nonrheumatic tricuspid valve disorder, unspecified |  |
| G542z00 | READ | 43855 | Tricuspid valve disorders NOS |  |
| G543.00 | READ | 12312 | Pulmonary valve disorders |  |
| G543000 | READ | 23608 | Pulmonary incompetence, non-rheumatic |  |
| G543011 | READ | 15640 | Pulmonary insufficiency, non-rheumatic |  |
| G543012 | READ | 15496 | Pulmonary regurgitation, non-rheumatic |  |
| G543100 | READ | 14723 | Pulmonary stenosis, non-rheumatic |  |
| G543200 | READ | 46736 | Pulmonary incompetence, cause unspecified |  |
| G543213 | READ | 38299 | Pulmonary insufficiency, cause unspecified |  |
| G543215 | READ | 6077 | Pulmonary regurgitation, cause unspecified |  |
| G543300 | READ | 2669 | Pulmonary stenosis, cause unspecified |  |
| G543311 | READ | 61878 | Pulmonary stenosis, cause unspecified |  |
| G543400 | READ | 34932 | Pulmonary valve stenosis with insufficiency |  |
| G543z00 | READ | 19957 | Pulmonary valve disorders NOS |  |
| G544.00 | READ | 40239 | Multiple valve diseases |  |
| G544000 | READ | 40582 | Disorders of both aortic and tricuspid valves |  |
| G544100 | READ | 19699 | Disorders of both mitral and tricuspid valves |  |
| G544200 | READ | 18475 | Combined disorders of mitral, aortic and tricuspid valves |  |
| G544X00 | READ | 57338 | Multiple valve disease, unspecified |  |
| G54z.00 | READ | 31979 | Endocarditis, valve unspecified |  |
| G54z000 | READ | 39671 | Incompetence of unspecified heart valve |  |
| G54z013 | READ | 22003 | Regurgitation of unspecified heart valve |  |
| G54z014 | READ | 89579 | Insufficiency of unspecified heart valve |  |
| G54z100 | READ | 10111 | Stenosis of unspecified heart valve |  |
| G54z300 | READ | 51472 | Endocarditis, valve unspecified, OS |  |
| G54z400 | READ | 46237 | Endocarditis in disease EC |  |
| G54z500 | READ | 5743 | Valvular heart disease |  |
| G54zz00 | READ | 939 | Endocarditis, valve unspecified, NOS |  |
| G55..00 | READ | 3204 | Cardiomyopathy |  |
| G550.00 | READ | 15990 | Endomyocardial fibrosis |  |
| G551.00 | READ | 8010 | Hypertrophic obstructive cardiomyopathy |  |
| G552.00 | READ | 68685 | Obscure African cardiomyopathy |  |
| G552.11 | READ | 73283 | Becker's disease |  |
| G553.00 | READ | 54478 | Endocardial fibroelastosis |  |
| G554.00 | READ | 57306 | Other primary cardiomyopathies |  |
| G554000 | READ | 5141 | Congestive cardiomyopathy |  |
| G554011 | READ | 68766 | Congestive obstructive cardiomyopathy |  |
| G554100 | READ | 41488 | Constrictive cardiomyopathy |  |
| G554200 | READ | 21852 | Familial cardiomyopathy |  |
| G554300 | READ | 3499 | Hypertrophic non-obstructive cardiomyopathy |  |
| G554400 | READ | 7535 | Primary dilated cardiomyopathy |  |
| G554z00 | READ | 40834 | Other primary cardiomyopathy NOS |  |
| G555.00 | READ | 4915 | Alcoholic cardiomyopathy |  |
| G557.00 | READ | 100966 | Nutritional and metabolic cardiomyopathies |  |
| G557000 | READ | 30667 | Amyloid heart disease |  |
| G557100 | READ | 49844 | Beriberi heart disease |  |
| G557300 | READ | 57334 | Gouty tophi of heart |  |
| G557500 | READ | 20035 | Thyrotoxic heart disease |  |
| G557z00 | READ | 64673 | Nutritional and metabolic cardiomyopathy NOS |  |
| G557z13 | READ | 59213 | Fatty infiltration heart |  |
| G558.00 | READ | 55850 | Cardiomyopathy in disease EC |  |
| G558000 | READ | 70855 | Cardiomyopathy in Friedreich's ataxia |  |
| G558100 | READ | 27683 | Cardiomyopathy in myotonic dystrophy |  |
| G558200 | READ | 64837 | Dystrophic cardiomyopathy |  |
| G558300 | READ | 47037 | Sarcoid heart disease |  |
| G558z00 | READ | 98020 | Cardiomyopathy in diseases EC, NOS |  |
| G55y.00 | READ | 42043 | Secondary cardiomyopathy NOS |  |
| G55y.11 | READ | 9402 | Secondary dilated cardiomyopathy |  |
| G55y000 | READ | 58938 | Cardiomyopathy due to drugs and other external agents |  |
| G55z.00 | READ | 22993 | Cardiomyopathy NOS |  |
| G56..00 | READ | 33673 | Conduction disorders |  |
| G56..11 | READ | 19191 | Conduction disorders of heart |  |
| G56..12 | READ | 4549 | Heart block |  |
| G560.00 | READ | 3810 | Complete atrioventricular block |  |
| G560.11 | READ | 24377 | Third degree atrioventricular block |  |
| G561.00 | READ | 3603 | Partial atrioventricular block |  |
| G561000 | READ | 58032 | Atrioventricular block unspecified |  |
| G561100 | READ | 12149 | First degree atrioventricular block |  |
| G561111 | READ | 46992 | Prolonged P-R interval |  |
| G561200 | READ | 10922 | Mobitz type II atrioventricular block |  |
| G561300 | READ | 27928 | Mobitz type I (Wenckebach) atrioventricular block |  |
| G561400 | READ | 36629 | Second degree atrioventricular block |  |
| G561z00 | READ | 27375 | Atrioventricular block NOS |  |
| G562.00 | READ | 7482 | Left bundle branch hemiblock |  |
| G562.11 | READ | 17840 | Left bundle branch block |  |
| G562000 | READ | 62349 | Left anterior fascicular block |  |
| G562100 | READ | 69809 | Left posterior fascicular block |  |
| G562z00 | READ | 53826 | Left bundle branch hemiblock NOS |  |
| G563.00 | READ | 26318 | Left main stem bundle branch block |  |
| G564.00 | READ | 9906 | Right bundle branch block |  |
| G565.00 | READ | 18117 | Other bundle branch block |  |
| G565000 | READ | 3032 | Bundle branch block unspecified |  |
| G565100 | READ | 98675 | Right BBB with left posterior fascicular block |  |
| G565200 | READ | 57069 | Right BBB with left anterior fascicular block |  |
| G565300 | READ | 72653 | Other bilateral bundle branch block |  |
| G565400 | READ | 10712 | Trifascicular block |  |
| G565500 | READ | 17206 | Bifascicular block |  |
| G565z00 | READ | 39003 | Other bundle branch block NOS |  |
| G566.00 | READ | 39843 | Other heart block |  |
| G566000 | READ | 18437 | Sinoatrial block |  |
| G566100 | READ | 54554 | Interventricular block NOS |  |
| G566200 | READ | 35947 | Right fascicular block |  |
| G566z00 | READ | 46178 | Other heart block NOS |  |
| G567.00 | READ | 25147 | Anomalous atrioventricular excitation |  |
| G567000 | READ | 50788 | Accelerated atrioventricular conduction |  |
| G567100 | READ | 69216 | Accessory atrioventricular conduction |  |
| G567200 | READ | 72888 | Pre-excitation atrioventricular conduction |  |
| G567300 | READ | 32059 | Ventricular pre-excitation |  |
| G567400 | READ | 8230 | Wolff-Parkinson-White syndrome |  |
| G567z00 | READ | 42803 | Anomalous atrioventricular excitation NOS |  |
| G56y.00 | READ | 27874 | Other conduction disorders |  |
| G56y000 | READ | 34326 | Lown-Ganong-Levine syndrome |  |
| G56y100 | READ | 5714 | Atrioventricular dissociation |  |
| G56y200 | READ | 22691 | Romano - Ward syndrome |  |
| G56y300 | READ | 39956 | Jervell and Lange-Nielsen syndrome |  |
| G56y400 | READ | 65653 | Right fascicular block |  |
| G56y500 | READ | 19337 | Long Q-T syndrome |  |
| G56yz00 | READ | 65073 | Other conduction disorders NOS |  |
| G56z.00 | READ | 44096 | Conduction disorders unspecified |  |
| G56z000 | READ | 3769 | Stokes-Adams syndrome |  |
| G56zz00 | READ | 36227 | Conduction disorders NOS |  |
| G57..00 | READ | 4044 | Cardiac dysrhythmias |  |
| G57..11 | READ | 6503 | Cardiac arrhythmias |  |
| G570.00 | READ | 4940 | Paroxysmal supraventricular tachycardia |  |
| G570000 | READ | 1297 | Paroxysmal atrial tachycardia |  |
| G570100 | READ | 23647 | Paroxysmal atrioventricular tachycardia |  |
| G570200 | READ | 51845 | Paroxysmal junctional tachycardia |  |
| G570300 | READ | 29491 | Paroxysmal nodal tachycardia |  |
| G570z00 | READ | 35124 | Paroxysmal supraventricular tachycardia NOS |  |
| G571.00 | READ | 3418 | Paroxysmal ventricular tachycardia |  |
| G571.11 | READ | 7794 | Ventricular tachycardia |  |
| G572.00 | READ | 25266 | Paroxysmal tachycardia unspecified |  |
| G572000 | READ | 60047 | Essential paroxysmal tachycardia |  |
| G572100 | READ | 70366 | Bouveret-Hoffmann syndrome |  |
| G572z00 | READ | 1381 | Paroxysmal tachycardia NOS |  |
| G573.00 | READ | 2212 | Atrial fibrillation and flutter |  |
| G573000 | READ | 1664 | Atrial fibrillation |  |
| G573100 | READ | 1757 | Atrial flutter |  |
| G573200 | READ | 1268 | Paroxysmal atrial fibrillation |  |
| G573300 | READ | 35127 | Non-rheumatic atrial fibrillation |  |
| G573z00 | READ | 23437 | Atrial fibrillation and flutter NOS |  |
| G574.00 | READ | 4374 | Ventricular fibrillation and flutter |  |
| G574000 | READ | 4827 | Ventricular fibrillation |  |
| G574011 | READ | 25583 | Cardiac arrest-ventricular fibrillation |  |
| G574100 | READ | 5484 | Ventricular flutter |  |
| G574z00 | READ | 41916 | Ventricular fibrillation and flutter NOS |  |
| G575.00 | READ | 2099 | Cardiac arrest |  |
| G575.11 | READ | 25407 | Cardio-respiratory arrest |  |
| G575.12 | READ | 33402 | Asystole |  |
| G575000 | READ | 33899 | Cardiac arrest with successful resuscitation |  |
| G575100 | READ | 21195 | Sudden cardiac death, so described |  |
| G575200 | READ | 51140 | Electromechanical dissociation with successful resuscitation |  |
| G575300 | READ | 7630 | Electromechanical dissociation |  |
| G575z00 | READ | 49882 | Cardiac arrest, unspecified |  |
| G576.00 | READ | 7457 | Ectopic beats |  |
| G576.11 | READ | 3909 | Premature beats |  |
| G577.00 | READ | 426 | Sinus arrhythmia |  |
| G57y.00 | READ | 7827 | Other cardiac dysrhythmias |  |
| G57y.11 | READ | 27463 | Pulsus alternans |  |
| G57y.14 | READ | 4421 | Heart beats irregular |  |
| G57y000 | READ | 3849 | Persistent sinus bradycardia |  |
| G57y100 | READ | 18268 | Severe sinus bradycardia |  |
| G57y300 | READ | 5576 | Sick sinus syndrome |  |
| G57y400 | READ | 7410 | Sinoatrial node dysfunction NOS |  |
| G57y500 | READ | 23494 | Wandering atrial pacemaker |  |
| G57y600 | READ | 8651 | Nodal rhythm disorder |  |
| G57y700 | READ | 7005 | Sinus tachycardia |  |
| G57y800 | READ | 9515 | Bigeminal pulse |  |
| G57y900 | READ | 1536 | Supraventricular tachycardia NOS |  |
| G57yA00 | READ | 31690 | Re-entry ventricular arrhythmia |  |
| G57yz00 | READ | 31133 | Other cardiac dysrhythmia NOS |  |
| G57z.00 | READ | 1535 | Cardiac dysrhythmia NOS |  |
| G58..00 | READ | 2062 | Heart failure |  |
| G58..11 | READ | 1223 | Cardiac failure |  |
| G580.00 | READ | 398 | Congestive heart failure |  |
| G580.11 | READ | 2906 | Congestive cardiac failure |  |
| G580.12 | READ | 10079 | Right heart failure |  |
| G580.13 | READ | 10154 | Right ventricular failure |  |
| G580.14 | READ | 9524 | Biventricular failure |  |
| G580000 | READ | 23707 | Acute congestive heart failure |  |
| G580100 | READ | 32671 | Chronic congestive heart failure |  |
| G580200 | READ | 27884 | Decompensated cardiac failure |  |
| G580300 | READ | 11424 | Compensated cardiac failure |  |
| G580400 | READ | 94870 | Congestive heart failure due to valvular disease |  |
| G581.00 | READ | 884 | Left ventricular failure |  |
| G581.11 | READ | 23481 | Asthma - cardiac |  |
| G581.12 | READ | 43618 | Pulmonary oedema - acute |  |
| G581.13 | READ | 5942 | Impaired left ventricular function |  |
| G581000 | READ | 5255 | Acute left ventricular failure |  |
| G582.00 | READ | 27964 | Acute heart failure |  |
| G58z.00 | READ | 4024 | Heart failure NOS |  |
| G58z.11 | READ | 12590 | Weak heart |  |
| G58z.12 | READ | 17278 | Cardiac failure NOS |  |
| G5y..00 | READ | 36193 | Other specified heart disease |  |
| G5y0.00 | READ | 10415 | Myocarditis NOS |  |
| G5y1.00 | READ | 24683 | Myocardial degeneration |  |
| G5y2.00 | READ | 56621 | Cardiovascular arteriosclerosis unspecified |  |
| G5y3.00 | READ | 509 | Cardiomegaly |  |
| G5y3.11 | READ | 13578 | Dilatation - cardiac |  |
| G5y3000 | READ | 15889 | Atrial dilatation |  |
| G5y3100 | READ | 3729 | Ventricular dilatation |  |
| G5y3200 | READ | 42014 | Cardiac dilatation NOS |  |
| G5y3300 | READ | 33348 | Atrial hypertrophy |  |
| G5y3400 | READ | 2724 | Ventricular hypertrophy |  |
| G5y3411 | READ | 562 | Left ventricular hypertrophy |  |
| G5y3500 | READ | 61124 | Cardiac hypertrophy NOS |  |
| G5y3z00 | READ | 14904 | Cardiomegaly NOS |  |
| G5y4.00 | READ | 99959 | Post cardiac operation functional disturbance |  |
| G5y4000 | READ | 16240 | Postcardiotomy syndrome |  |
| G5y4z00 | READ | 96799 | Post cardiac operation heart failure NOS |  |
| G5y5.00 | READ | 31784 | Rupture of chordae tendinae |  |
| G5y6.00 | READ | 49735 | Rupture of papillary muscle |  |
| G5y7.00 | READ | 34437 | Sarcoid myocarditis |  |
| G5y8.00 | READ | 49787 | Rheumatoid myocarditis |  |
| G5y9.00 | READ | 29180 | Cardiac septal defect, acquired |  |
| G5yA.00 | READ | 43816 | Rheumatoid carditis |  |
| G5yX.00 | READ | 22672 | Cardiovascular disease, unspecified |  |
| G5yy.00 | READ | 59687 | Other ill-defined heart disease |  |
| G5yy.11 | READ | 67528 | Papillary muscle disease |  |
| G5yy000 | READ | 43937 | Papillary muscle atrophy |  |
| G5yy100 | READ | 40793 | Papillary muscle degeneration |  |
| G5yy200 | READ | 92267 | Papillary muscle dysfunction |  |
| G5yy300 | READ | 61774 | Papillary muscle scarring |  |
| G5yy500 | READ | 20011 | Hyperkinetic heart disease |  |
| G5yy600 | READ | 30454 | Atrial thrombosis |  |
| G5yy700 | READ | 21854 | Left ventricular thrombosis |  |
| G5yy800 | READ | 7839 | Right ventricular thrombosis |  |
| G5yy900 | READ | 8966 | Left ventricular systolic dysfunction |  |
| G5yyA00 | READ | 12550 | Left ventricular diastolic dysfunction |  |
| G5yyz00 | READ | 41179 | Other ill-defined heart disease NOS |  |
| G5yz.00 | READ | 1811 | Other heart disease NOS |  |
| G5z..00 | READ | 1490 | Heart disease NOS |  |
| P5...00 | READ | 31518 | Bulbus cordis and cardiac septal closure anomalies |  |
| P5...11 | READ | 21943 | Cardiac septal defects |  |
| P5...12 | READ | 23754 | Congenital heart disease, septal and bulbar anomalies |  |
| P5...13 | READ | 18785 | Heart septal defects |  |
| P50..00 | READ | 4964 | Common aorto-pulmonary trunk |  |
| P50..11 | READ | 37405 | Aortic septal defect |  |
| P50..12 | READ | 98893 | Common truncus |  |
| P500.00 | READ | 72604 | Absent septum between aorta and pulmonary artery |  |
| P500.11 | READ | 61914 | Persistent truncus arteriosus |  |
| P500.12 | READ | 45187 | Truncus arteriosus |  |
| P501.00 | READ | 45505 | Aortic septal defect |  |
| P501.11 | READ | 51418 | Aortopulmonary window |  |
| P501.12 | READ | 65330 | Aorticopulmonary septal defect |  |
| P502.00 | READ | 66245 | Persistent truncus arteriosus |  |
| P502.11 | READ | 41371 | Truncus arteriosus |  |
| P50z.00 | READ | 94344 | Common aorto-pulmonary trunk NOS |  |
| P51..00 | READ | 2816 | Transposition of great vessels |  |
| P510.00 | READ | 69858 | Total great vessel transposition |  |
| P511.00 | READ | 1778 | Double outlet right ventricle |  |
| P511100 | READ | 23988 | Dextratransposition of aorta |  |
| P511300 | READ | 40025 | Taussig-Bing syndrome |  |
| P511z00 | READ | 65318 | Double outlet right ventricle NOS |  |
| P512.00 | READ | 63390 | Corrected great vessel transposition |  |
| P51y.00 | READ | 62169 | Other specified transposition of great vessels |  |
| P51y.11 | READ | 61100 | Transposition of aorta |  |
| P51z.00 | READ | 73539 | Great vessel transposition NOS |  |
| P51z.11 | READ | 100622 | Transposition of arterial trunk NEC |  |
| P52..00 | READ | 4864 | Tetralogy of Fallot |  |
| P520.00 | READ | 38967 | Tetralogy of Fallot, unspecified |  |
| P520.11 | READ | 23692 | Ventricular septal defect in Fallot's tetralogy |  |
| P520.12 | READ | 71252 | Dextraposition of aorta in Fallot's tetralogy |  |
| P521.00 | READ | 51649 | Pentalogy of Fallot |  |
| P52z.00 | READ | 63046 | Tetralogy of Fallot NOS |  |
| P53..00 | READ | 19969 | Common ventricle |  |
| P54..00 | READ | 246 | Ventricular septal defect |  |
| P540.00 | READ | 42132 | Ventricular septal defect, unspecified |  |
| P541.00 | READ | 56575 | Interventricular septal defect |  |
| P542.00 | READ | 67657 | Left ventricle to right atrial communication |  |
| P543.00 | READ | 9011 | Eisenmenger's complex |  |
| P544.00 | READ | 51897 | Gerbode's defect |  |
| P545.00 | READ | 20153 | Roger's disease |  |
| P54y.00 | READ | 54772 | Other specified ventricular septal defect |  |
| P54z.00 | READ | 34067 | Ventricular septal defect NOS |  |
| P55..00 | READ | 7474 | Ostium secundum atrial septal defect |  |
| P550.00 | READ | 3255 | Atrial septal defect NOS |  |
| P550.11 | READ | 40673 | Auricular septal defect NOS |  |
| P550.12 | READ | 28174 | Interatrial septal defect NEC |  |
| P550.13 | READ | 72577 | Interauricular septal defect |  |
| P551.00 | READ | 3625 | Patent foramen ovale |  |
| P552.00 | READ | 37816 | Persistent ostium secundum |  |
| P553.00 | READ | 59144 | Lutembacher's syndrome |  |
| P55y.00 | READ | 53088 | Other specified ostium secundum atrial septal defect |  |
| P55y.11 | READ | 18395 | Other specified atrial septal defect |  |
| P55z.00 | READ | 54243 | Ostium secundum atrial septal defect NOS |  |
| P56..00 | READ | 63340 | Endocardial cushion defects |  |
| P561.00 | READ | 51053 | Ostium primum defect |  |
| P561.11 | READ | 93161 | Persistent ostium primum |  |
| P56y.00 | READ | 101393 | Other specified endocardial cushion defects |  |
| P56z.00 | READ | 49702 | Endocardial cushion defects NOS |  |
| P56z000 | READ | 55535 | Common atrium |  |
| P56z011 | READ | 61715 | Cor triloculare biventriculare |  |
| P56z100 | READ | 43049 | Common atrioventricular canal |  |
| P56z200 | READ | 44896 | Common atrioventricular-type ventricular septal defect |  |
| P56zz00 | READ | 73816 | Endocardial cushion defects NOS |  |
| P58..00 | READ | 46117 | Double outlet left ventricle |  |
| P59..00 | READ | 9361 | Isomerism of atrial appendages |  |
| P5X..00 | READ | 48205 | Congenital malforms of cardiac chambers+connections unsp |  |
| P5y..00 | READ | 66401 | Other heart bulb and septal closure defect |  |
| P5z..00 | READ | 54196 | Heart bulb or septal closure defects NOS |  |
| P6...00 | READ | 5621 | Other congenital heart anomalies |  |
| P60..00 | READ | 39992 | Pulmonary valve anomalies |  |
| P600.00 | READ | 69940 | Pulmonary valve anomaly, unspecified |  |
| P601.00 | READ | 3862 | Congenital atresia of the pulmonary valve |  |
| P601000 | READ | 54488 | Hypoplasia of pulmonary valve |  |
| P601z00 | READ | 93561 | Congenital atresia of pulmonary valve NOS |  |
| P602.00 | READ | 22778 | Congenital pulmonary stenosis |  |
| P602100 | READ | 45452 | Congenital fusion of pulmonary valve segment |  |
| P602z00 | READ | 33919 | Congenital pulmonary stenosis NOS |  |
| P603.00 | READ | 21851 | Right hypoplastic heart syndrome |  |
| P603.11 | READ | 68982 | Pseudotruncus arteriosus |  |
| P60z.00 | READ | 44478 | Other pulmonary valve anomalies |  |
| P60z100 | READ | 52607 | Fallot's trilogy |  |
| P60zz00 | READ | 70880 | Other pulmonary valve anomaly NOS |  |
| P61..00 | READ | 12752 | Congenital tricuspid atresia and stenosis |  |
| P610.00 | READ | 21272 | Congenital tricuspid atresia |  |
| P611.00 | READ | 69169 | Congenital tricuspid stenosis |  |
| P61z.00 | READ | 100065 | Congenital tricuspid atresia or stenosis NOS |  |
| P62..00 | READ | 23709 | Ebstein's anomaly |  |
| P63..00 | READ | 6886 | Congenital aortic valve stenosis |  |
| P64..00 | READ | 8636 | Congenital aortic valve insufficiency |  |
| P640.00 | READ | 58734 | Congenital aortic valve insufficiency, unspecified |  |
| P641.00 | READ | 3300 | Bicuspid aortic valve |  |
| P64z.00 | READ | 6843 | Congenital aortic valve insufficiency NOS |  |
| P65..00 | READ | 57091 | Congenital mitral stenosis |  |
| P65..11 | READ | 68888 | Duroziez's disease |  |
| P650.00 | READ | 90551 | Congenital mitral stenosis, unspecified |  |
| P651.00 | READ | 73592 | Fused commissure of the mitral valve |  |
| P652.00 | READ | 63069 | Parachute deformity of the mitral valve |  |
| P65z.00 | READ | 100291 | Congenital mitral stenosis NOS |  |
| P66..00 | READ | 61651 | Congenital mitral insufficiency |  |
| P67..00 | READ | 20772 | Hypoplastic left heart syndrome |  |
| P68..00 | READ | 89256 | Congenital heart disease |  |
| P6W..00 | READ | 46825 | Congenital malformation of aortic and mitral valves unsp |  |
| P6X..00 | READ | 50529 | Congenital malformation of tricuspid valve, unspecified |  |
| P6y..00 | READ | 24789 | Other specified heart anomalies |  |
| P6y0.00 | READ | 16539 | Subaortic stenosis |  |
| P6y1.00 | READ | 34668 | Cor triatriatum |  |
| P6y2.00 | READ | 9401 | Pulmonary infundibular stenosis |  |
| P6y3.00 | READ | 99128 | Obstructive heart anomaly NEC |  |
| P6y3000 | READ | 32748 | Uhl's disease |  |
| P6y3z00 | READ | 97818 | Obstructive heart anomaly NEC NOS |  |
| P6y4.00 | READ | 25481 | Coronary artery anomaly |  |
| P6y4000 | READ | 71956 | Congenital absence of coronary artery |  |
| P6y4100 | READ | 62163 | Single coronary artery |  |
| P6y4400 | READ | 31373 | Anomalous coronary artery communication |  |
| P6y4411 | READ | 28705 | Congenital coronary arterio-venous fistula |  |
| P6y4500 | READ | 49901 | Congenital coronary aneurysm |  |
| P6y4z00 | READ | 53546 | Coronary artery anomaly NOS |  |
| P6y5.00 | READ | 24533 | Congenital heart block |  |
| P6y5000 | READ | 68097 | Congenital heart block, unspecified |  |
| P6y5100 | READ | 52310 | Congenital complete atrio-ventricular heart block |  |
| P6y5200 | READ | 102167 | Congenital incomplete atrio-ventricular heart block |  |
| P6y5z00 | READ | 70992 | Congenital heart block NOS |  |
| P6y6.00 | READ | 71678 | Heart and cardiac apex malposition |  |
| P6y6.11 | READ | 20466 | Ectopic heart |  |
| P6y6000 | READ | 3774 | Dextrocardia |  |
| P6y6100 | READ | 72259 | Levocardia |  |
| P6y6111 | READ | 69556 | Laevocardia |  |
| P6y6200 | READ | 72405 | Mesocardia |  |
| P6y6300 | READ | 50284 | Ectopia cordis |  |
| P6y6z00 | READ | 92426 | Heart or cardiac apex malposition NOS |  |
| P6y7.00 | READ | 52615 | Myocardial bridge of coronary artery |  |
| P6yy.00 | READ | 24854 | Other specified heart anomalies |  |
| P6yy.11 | READ | 44767 | Hypoplastic aortic orifice or valve |  |
| P6yy.12 | READ | 9232 | Hypoplasia of heart NOS |  |
| P6yy000 | READ | 93089 | Atresia of cardiac vein |  |
| P6yy100 | READ | 68063 | Hypoplasia of cardiac vein |  |
| P6yy200 | READ | 34007 | Congenital cardiomegaly |  |
| P6yy300 | READ | 49133 | Congenital left ventricular diverticulum |  |
| P6yy400 | READ | 50362 | Congenital pericardial defect |  |
| P6yy411 | READ | 101896 | Congenital absence of pericardium |  |
| P6yy500 | READ | 68894 | Congenital anomaly of myocardium |  |
| P6yy700 | READ | 37451 | Atresia of heart valve NEC |  |
| P6yy900 | READ | 23752 | Congenital epicardial cyst |  |
| P6yyA00 | READ | 95785 | Hemicardia |  |
| P6yyC00 | READ | 98317 | Fusion of mitral valve cusps |  |
| P6yyz00 | READ | 24714 | Other specified heart anomalies NOS |  |
| P6z..00 | READ | 247 | Congenital heart anomaly NOS |  |
| P6z..11 | READ | 8279 | Chiari's malformation |  |
| P6z0.00 | READ | 26626 | Unspecified anomaly of heart valve |  |
| P6z1100 | READ | 64778 | Anomalous ventricular bands |  |
| P6z2.00 | READ | 38968 | Acyanotic congenital heart disease NOS |  |
| P6z3.00 | READ | 3863 | Cyanotic congenital heart disease NOS |  |
| P6z3.11 | READ | 7262 | Blue baby |  |
| P6zz.00 | READ | 11982 | Congenital heart anomaly NOS |  |
| P8...00 | READ | 46398 | Respiratory system congenital anomalies |  |
| P80..00 | READ | 31027 | Choanal atresia |  |
| P800.00 | READ | 66643 | Choanal atresia, unspecified |  |
| P802.00 | READ | 68606 | Atresia of the posterior nares |  |
| P804.00 | READ | 96389 | Congenital stenosis of the posterior nares |  |
| P80z.00 | READ | 60514 | Choanal atresia NOS |  |
| P81..00 | READ | 45438 | Other anomalies of nose |  |
| P810.00 | READ | 15715 | Congenital nose deformity, unspecified |  |
| P811.00 | READ | 52579 | Absent nose |  |
| P812.00 | READ | 60987 | Accessory nose |  |
| P813.00 | READ | 66698 | Congenital cleft nose |  |
| P814.00 | READ | 37110 | Deformity of nasal sinus wall |  |
| P815.00 | READ | 58422 | Congenital notching of tip of nose |  |
| P816.00 | READ | 73595 | Congenital perforation of the nasal sinus wall |  |
| P817.00 | READ | 10793 | Perforated nasal septum |  |
| P818.00 | READ | 65192 | Congenital fissure of nose |  |
| P819.00 | READ | 28199 | Congenital hypoplastic nose |  |
| P81z.00 | READ | 45376 | Other anomalies of nose NOS |  |
| P81z.11 | READ | 16933 | Single nostril |  |
| P82..00 | READ | 31771 | Congenital web of larynx |  |
| P821.00 | READ | 42855 | Congenital glottic web of larynx |  |
| P822.00 | READ | 97956 | Congenital subglottic web of larynx |  |
| P82z.00 | READ | 70985 | Congenital web of larynx NOS |  |
| P83..00 | READ | 29373 | Other anomalies of larynx, trachea and bronchus |  |
| P830200 | READ | 101497 | Agenesis of trachea |  |
| P831.00 | READ | 63530 | Anomaly of laryngeal and tracheal cartilage |  |
| P831000 | READ | 44151 | Anomaly of cricoid cartilage |  |
| P831100 | READ | 43205 | Anomaly of epiglottis |  |
| P831200 | READ | 47467 | Anomaly of thyroid cartilage |  |
| P831300 | READ | 31020 | Anomaly of tracheal cartilage |  |
| P831400 | READ | 2910 | Tracheomalacia |  |
| P831500 | READ | 51933 | Laryngeal hypoplasia |  |
| P831600 | READ | 7592 | Laryngomalacia |  |
| P831z00 | READ | 55062 | Anomaly of laryngeal or tracheal cartilage NOS |  |
| P832.00 | READ | 72001 | Atresia of larynx and trachea |  |
| P832000 | READ | 61727 | Atresia of epiglottis |  |
| P832100 | READ | 92834 | Atresia of glottis |  |
| P832300 | READ | 63288 | Atresia of trachea |  |
| P833.00 | READ | 59273 | Congenital stenosis of larynx, trachea and bronchus |  |
| P833000 | READ | 61450 | Congenital stenosis of larynx |  |
| P833100 | READ | 48818 | Congenital stenosis of trachea |  |
| P833200 | READ | 71893 | Congenital stenosis of bronchus |  |
| P833300 | READ | 49142 | Congenital subglottic stenosis |  |
| P833400 | READ | 55144 | Congenital supraglottic stenosis |  |
| P83y.00 | READ | 63637 | Other anomaly of larynx, trachea and bronchus |  |
| P83y100 | READ | 37835 | Congenital dilatation of trachea |  |
| P83y300 | READ | 45351 | Congenital laryngocele |  |
| P83y500 | READ | 99683 | Congenital diverticulum of trachea |  |
| P83y600 | READ | 58868 | Congenital fissure of epiglottis |  |
| P83y800 | READ | 55656 | Rudimentary tracheal bronchus |  |
| P83y900 | READ | 5405 | Congenital laryngeal stridor |  |
| P83yB00 | READ | 11845 | Congenital bronchomalacia |  |
| P83yX00 | READ | 36474 | Congenital malformation of larynx, unspecified |  |
| P83yw00 | READ | 63507 | Other anomaly of larynx |  |
| P83yx00 | READ | 70193 | Other anomaly of trachea |  |
| P83z.00 | READ | 36779 | Other anomalies of larynx, trachea or bronchus NOS |  |
| P84..00 | READ | 10699 | Congenital cystic lung |  |
| P840.00 | READ | 66223 | Congenital cystic lung disease, unspecified |  |
| P841.00 | READ | 31820 | Congenital polycystic lung |  |
| P841.11 | READ | 57330 | Multiple lung cysts |  |
| P841.12 | READ | 50980 | Multiple congenital bronchogenic cysts |  |
| P842.00 | READ | 66951 | Congenital honeycomb lung |  |
| P843.00 | READ | 38769 | Single lung cyst |  |
| P843.11 | READ | 15639 | Lung cyst |  |
| P843.12 | READ | 32600 | Congenital bronchogenic cyst |  |
| P84y.00 | READ | 64802 | Other specified congenital cystic lung |  |
| P84z.00 | READ | 33804 | Congenital cystic lung NOS |  |
| P85..00 | READ | 26838 | Lung agenesis, hypoplasia and dysplasia |  |
| P850.00 | READ | 52664 | Aplasia of lung |  |
| P851.00 | READ | 23874 | Hypoplasia of lung |  |
| P852.00 | READ | 35661 | Sequestration of lung |  |
| P853.00 | READ | 59444 | Agenesis of lung |  |
| P853.11 | READ | 54384 | Congenital absence of lung |  |
| P853000 | READ | 68268 | Congenital absence of lung fissures |  |
| P853100 | READ | 97185 | Congenital absence of lobe of lung |  |
| P853z00 | READ | 89691 | Agenesis of lung NOS |  |
| P85y.00 | READ | 99799 | Other specified lung agenesis, hypoplasia or dysplasia |  |
| P85y000 | READ | 50991 | Fusion of lobes of lung |  |
| P85yz00 | READ | 100037 | Other lung agenesis, hypoplasia or dysplasia NOS |  |
| P85z.00 | READ | 93008 | Lung agenesis, hypoplasia or dysplasia NOS |  |
| P86..00 | READ | 18487 | Other lung anomalies |  |
| P860.00 | READ | 63453 | Anomaly of lung, unspecified |  |
| P861.00 | READ | 56427 | Congenital bronchiectasis |  |
| P86y.00 | READ | 60290 | Other lung anomaly |  |
| P86y100 | READ | 48229 | Azygos lobe of lung |  |
| P86y200 | READ | 83570 | Accessory lobe of lung |  |
| P86yz00 | READ | 64758 | Other lung anomaly NOS |  |
| P86z.00 | READ | 8311 | Lung anomaly NOS |  |
| P8y..00 | READ | 89730 | Other specified respiratory system anomalies |  |
| P8y0.00 | READ | 70956 | Abnormal pericardio-pleural communication |  |
| P8y1.00 | READ | 39900 | Anomaly, pleural folds |  |
| P8y2.00 | READ | 52894 | Atresia of nasopharynx |  |
| P8y3.00 | READ | 32883 | Congenital cyst of mediastinum |  |
| P8y4.00 | READ | 44347 | Congenital pulmonary lymphangiectasis |  |
| P8yz.00 | READ | 32416 | Other specified respiratory system anomaly NOS |  |
| P8z..00 | READ | 46397 | Respiratory system anomaly NOS |  |

| risk_factor definition 2: Renal disease |
| --- |
| n= 110 medcodes used |

| **Read_code** | **readoxmisflag** | **medcode** | **desc** |  |
| --- | --- | --- | --- | --- |
| 1Z1..00 | READ | 12720 | Chronic renal impairment |  |
| 1Z10.00 | READ | 29013 | Chronic kidney disease stage 1 |  |
| 1Z11.00 | READ | 12586 | Chronic kidney disease stage 2 |  |
| 1Z12.00 | READ | 12566 | Chronic kidney disease stage 3 |  |
| 1Z13.00 | READ | 12479 | Chronic kidney disease stage 4 |  |
| 1Z14.00 | READ | 12585 | Chronic kidney disease stage 5 |  |
| 1Z15.00 | READ | 94965 | Chronic kidney disease stage 3A |  |
| 1Z16.00 | READ | 95179 | Chronic kidney disease stage 3B |  |
| 1Z17.00 | READ | 94789 | Chronic kidney disease stage 1 with proteinuria |  |
| 1Z18.00 | READ | 95572 | Chronic kidney disease stage 1 without proteinuria |  |
| 1Z19.00 | READ | 95146 | Chronic kidney disease stage 2 with proteinuria |  |
| 1Z1A.00 | READ | 95121 | Chronic kidney disease stage 2 without proteinuria |  |
| 1Z1B.00 | READ | 94793 | Chronic kidney disease stage 3 with proteinuria |  |
| 1Z1B.11 | READ | 95145 | CKD stage 3 with proteinuria |  |
| 1Z1C.00 | READ | 95123 | Chronic kidney disease stage 3 without proteinuria |  |
| 1Z1C.11 | READ | 95188 | CKD stage 3 without proteinuria |  |
| 1Z1D.00 | READ | 95408 | Chronic kidney disease stage 3A with proteinuria |  |
| 1Z1D.11 | READ | 95571 | CKD stage 3A with proteinuria |  |
| 1Z1E.00 | READ | 95175 | Chronic kidney disease stage 3A without proteinuria |  |
| 1Z1E.11 | READ | 95176 | CKD stage 3A without proteinuria |  |
| 1Z1F.00 | READ | 95178 | Chronic kidney disease stage 3B with proteinuria |  |
| 1Z1F.11 | READ | 95180 | CKD stage 3B with proteinuria |  |
| 1Z1G.00 | READ | 95177 | Chronic kidney disease stage 3B without proteinuria |  |
| 1Z1G.11 | READ | 100633 | CKD stage 3B without proteinuria |  |
| 1Z1H.00 | READ | 95122 | Chronic kidney disease stage 4 with proteinuria |  |
| 1Z1J.00 | READ | 95406 | Chronic kidney disease stage 4 without proteinuria |  |
| 1Z1K.00 | READ | 95508 | Chronic kidney disease stage 5 with proteinuria |  |
| 1Z1L.00 | READ | 95405 | Chronic kidney disease stage 5 without proteinuria |  |
| 7B00.00 | READ | 2997 | Transplantation of kidney |  |
| 7B00000 | READ | 55151 | Autotransplant of kidney |  |
| 7B00100 | READ | 11745 | Transplantation of kidney from live donor |  |
| 7B00111 | READ | 66705 | Allotransplantation of kidney from live donor |  |
| 7B00200 | READ | 24361 | Transplantation of kidney from cadaver |  |
| 7B00211 | READ | 98364 | Allotransplantation of kidney from cadaver |  |
| 7B00300 | READ | 89924 | Allotransplantation of kidney from cadaver, heart-beating |  |
| 7B00y00 | READ | 70874 | Other specified transplantation of kidney |  |
| 7B00z00 | READ | 5504 | Transplantation of kidney NOS |  |
| 7B01.00 | READ | 867 | Total nephrectomy |  |
| 7B01.11 | READ | 7568 | Total excision of kidney |  |
| 7B01000 | READ | 6136 | Radical nephrectomy |  |
| 7B01011 | READ | 68574 | Nephrectomy and excision of perirenal tissue |  |
| 7B01100 | READ | 1600 | Nephroureterectomy-unspecified |  |
| 7B01200 | READ | 51039 | Bilateral nephrectomy |  |
| 7B01300 | READ | 34834 | Heminephrectomy for horseshoe kidney |  |
| 7B01311 | READ | 60919 | Excision of half of horseshoe kidney |  |
| 7B01400 | READ | 29120 | Simple nephrectomy - other |  |
| 7B01500 | READ | 48121 | Transplant nephrectomy |  |
| 7B01511 | READ | 72004 | Excision of rejected transplanted kidney |  |
| 7B01600 | READ | 37007 | Simple nephrectomy -live donor |  |
| 7B01700 | READ | 35225 | Nephroureterectomy with open lower ureterectomy |  |
| 7B01800 | READ | 49535 | Nephroureterectomy with pluck lower ureterectomy |  |
| 7B01y00 | READ | 56892 | Other specified total nephrectomy |  |
| 7B01z00 | READ | 34366 | Total nephrectomy NOS |  |
| 7B06300 | READ | 26862 | Exploration of renal transplant |  |
| 8L50.00 | READ | 17253 | Renal transplant planned |  |
| K01..00 | READ | 2999 | Nephrotic syndrome |  |
| K010.00 | READ | 9840 | Nephrotic syndrome with proliferative glomerulonephritis |  |
| K011.00 | READ | 1803 | Nephrotic syndrome with membranous glomerulonephritis |  |
| K012.00 | READ | 99644 | Nephrotic syndrome+membranoproliferative glomerulonephritis |  |
| K013.00 | READ | 29634 | Nephrotic syndrome with minimal change glomerulonephritis |  |
| K013.11 | READ | 40349 | Lipoid nephrosis |  |
| K013.12 | READ | 57926 | Steroid sensitive nephrotic syndrome |  |
| K014.00 | READ | 23913 | Nephrotic syndrome, minor glomerular abnormality |  |
| K015.00 | READ | 22852 | Nephrotic syndrome, focal and segmental glomerular lesions |  |
| K016.00 | READ | 19316 | Nephrotic syndrome, diffuse membranous glomerulonephritis |  |
| K017.00 | READ | 21947 | Nephrotic syn difus mesangial prolifertiv glomerulonephritis |  |
| K018.00 | READ | 50472 | Nephrotic syn,difus endocapilary proliftv glomerulonephritis |  |
| K019.00 | READ | 21989 | Nephrotic syn,diffuse mesangiocapillary glomerulonephritis |  |
| K01A.00 | READ | 56987 | Nephrotic syndrome, dense deposit disease |  |
| K01B.00 | READ | 17365 | Nephrotic syndrome, diffuse crescentic glomerulonephritis |  |
| K01w.00 | READ | 63786 | Congenital nephrotic syndrome |  |
| K01w000 | READ | 72303 | Finnish nephrosis syndrome |  |
| K01x000 | READ | 47922 | Nephrotic syndrome in amyloidosis |  |
| K01x100 | READ | 2471 | Nephrotic syndrome in diabetes mellitus |  |
| K01x111 | READ | 45499 | Kimmelstiel - Wilson disease |  |
| K01x200 | READ | 99201 | Nephrotic syndrome in malaria |  |
| K01x300 | READ | 58750 | Nephrotic syndrome in polyarteritis nodosa |  |
| K01x400 | READ | 47672 | Nephrotic syndrome in systemic lupus erythematosus |  |
| K01x411 | READ | 22205 | Lupus nephritis |  |
| K01y.00 | READ | 94373 | Nephrotic syndrome with other pathological kidney lesions |  |
| K01z.00 | READ | 27427 | Nephrotic syndrome NOS |  |
| K02..00 | READ | 7804 | Chronic glomerulonephritis |  |
| K02..11 | READ | 10647 | Nephritis - chronic |  |
| K02..12 | READ | 11875 | Nephropathy - chronic |  |
| K020.00 | READ | 34998 | Chronic proliferative glomerulonephritis |  |
| K021.00 | READ | 10809 | Chronic membranous glomerulonephritis |  |
| K022.00 | READ | 61494 | Chronic membranoproliferative glomerulonephritis |  |
| K023.00 | READ | 65064 | Chronic rapidly progressive glomerulonephritis |  |
| K02y.00 | READ | 60960 | Other chronic glomerulonephritis |  |
| K02y000 | READ | 97758 | Chronic glomerulonephritis + diseases EC |  |
| K02y200 | READ | 4669 | Chronic focal glomerulonephritis |  |
| K02y300 | READ | 65400 | Chronic diffuse glomerulonephritis |  |
| K02yz00 | READ | 63615 | Other chronic glomerulonephritis NOS |  |
| K02z.00 | READ | 15097 | Chronic glomerulonephritis NOS |  |
| K05..00 | READ | 512 | Chronic renal failure |  |
| K05..11 | READ | 10081 | Chronic uraemia |  |
| K05..12 | READ | 53852 | End stage renal failure |  |
| K050.00 | READ | 6712 | End stage renal failure |  |
| K0A3.00 | READ | 21297 | Chronic nephritic syndrome |  |
| K0A3000 | READ | 66505 | Chronic nephritic syndrome, minor glomerular abnormality |  |
| K0A3100 | READ | 40413 | Chronic nephritic syndrm focal+segmental glomerular lesions |  |
| K0A3200 | READ | 57168 | Chron nephritic syndrom difuse membranous glomerulonephritis |  |
| K0A3300 | READ | 56893 | Chron neph syn difus mesangial prolifrtiv glomerulonephritis |  |
| K0A3500 | READ | 73026 | Chronic neph syn difus mesangiocapillary glomerulonephritis |  |
| K0A3600 | READ | 60198 | Chronic nephritic syndrome, dense deposit disease |  |
| K0A3700 | READ | 60857 | Chronic nephritic syn diffuse crescentic glomerulonephritis |  |
| K0D..00 | READ | 8330 | End-stage renal disease |  |
| TB00100 | READ | 54990 | Kidney transplant with complication, without blame |  |
| TB00111 | READ | 18774 | Renal transplant with complication, without blame |  |
| ZV42000 | READ | 5911 | [V]Kidney transplanted |  |

| risk_factor definition 3: Diabetes_diagnosis |
| --- |
| n= 259 medcodes used |

| **Read_code** | **readoxmisflag** | **medcode** | **desc** |  |
| --- | --- | --- | --- | --- |
| C10..00 | READ | 711 | Diabetes mellitus |  |
| C100.00 | READ | 38986 | Diabetes mellitus with no mention of complication |  |
| C100000 | READ | 24490 | Diabetes mellitus, juvenile type, no mention of complication |  |
| C100011 | READ | 1038 | Insulin dependent diabetes mellitus |  |
| C100100 | READ | 14803 | Diabetes mellitus, adult onset, no mention of complication |  |
| C100111 | READ | 14889 | Maturity onset diabetes |  |
| C100112 | READ | 506 | Non-insulin dependent diabetes mellitus |  |
| C100z00 | READ | 50972 | Diabetes mellitus NOS with no mention of complication |  |
| C101.00 | READ | 1682 | Diabetes mellitus with ketoacidosis |  |
| C101000 | READ | 53200 | Diabetes mellitus, juvenile type, with ketoacidosis |  |
| C101100 | READ | 54856 | Diabetes mellitus, adult onset, with ketoacidosis |  |
| C101y00 | READ | 38617 | Other specified diabetes mellitus with ketoacidosis |  |
| C101z00 | READ | 42505 | Diabetes mellitus NOS with ketoacidosis |  |
| C102.00 | READ | 21482 | Diabetes mellitus with hyperosmolar coma |  |
| C102000 | READ | 40023 | Diabetes mellitus, juvenile type, with hyperosmolar coma |  |
| C102100 | READ | 43139 | Diabetes mellitus, adult onset, with hyperosmolar coma |  |
| C102z00 | READ | 72345 | Diabetes mellitus NOS with hyperosmolar coma |  |
| C103.00 | READ | 15690 | Diabetes mellitus with ketoacidotic coma |  |
| C103000 | READ | 42567 | Diabetes mellitus, juvenile type, with ketoacidotic coma |  |
| C103100 | READ | 68843 | Diabetes mellitus, adult onset, with ketoacidotic coma |  |
| C103y00 | READ | 59288 | Other specified diabetes mellitus with coma |  |
| C103z00 | READ | 65062 | Diabetes mellitus NOS with ketoacidotic coma |  |
| C104.00 | READ | 16502 | Diabetes mellitus with renal manifestation |  |
| C104.11 | READ | 2475 | Diabetic nephropathy |  |
| C104000 | READ | 93922 | Diabetes mellitus, juvenile type, with renal manifestation |  |
| C104100 | READ | 35105 | Diabetes mellitus, adult onset, with renal manifestation |  |
| C104y00 | READ | 13279 | Other specified diabetes mellitus with renal complications |  |
| C104z00 | READ | 35107 | Diabetes mellitus with nephropathy NOS |  |
| C105.00 | READ | 33254 | Diabetes mellitus with ophthalmic manifestation |  |
| C105000 | READ | 69748 | Diabetes mellitus, juvenile type, + ophthalmic manifestation |  |
| C105100 | READ | 41389 | Diabetes mellitus, adult onset, + ophthalmic manifestation |  |
| C105y00 | READ | 47377 | Other specified diabetes mellitus with ophthalmic complicatn |  |
| C105z00 | READ | 34283 | Diabetes mellitus NOS with ophthalmic manifestation |  |
| C106.00 | READ | 16230 | Diabetes mellitus with neurological manifestation |  |
| C106.11 | READ | 59903 | Diabetic amyotrophy |  |
| C106.12 | READ | 7795 | Diabetes mellitus with neuropathy |  |
| C106.13 | READ | 16491 | Diabetes mellitus with polyneuropathy |  |
| C106000 | READ | 67853 | Diabetes mellitus, juvenile, + neurological manifestation |  |
| C106100 | READ | 39317 | Diabetes mellitus, adult onset, + neurological manifestation |  |
| C106y00 | READ | 61523 | Other specified diabetes mellitus with neurological comps |  |
| C106z00 | READ | 22573 | Diabetes mellitus NOS with neurological manifestation |  |
| C107.00 | READ | 35399 | Diabetes mellitus with peripheral circulatory disorder |  |
| C107.11 | READ | 32403 | Diabetes mellitus with gangrene |  |
| C107.12 | READ | 32556 | Diabetes with gangrene |  |
| C107000 | READ | 70448 | Diabetes mellitus, juvenile +peripheral circulatory disorder |  |
| C107100 | READ | 63357 | Diabetes mellitus, adult, + peripheral circulatory disorder |  |
| C107200 | READ | 33807 | Diabetes mellitus, adult with gangrene |  |
| C107300 | READ | 69124 | IDDM with peripheral circulatory disorder |  |
| C107400 | READ | 56803 | NIDDM with peripheral circulatory disorder |  |
| C107z00 | READ | 65025 | Diabetes mellitus NOS with peripheral circulatory disorder |  |
| C108.00 | READ | 1647 | Insulin dependent diabetes mellitus |  |
| C108.11 | READ | 18505 | IDDM-Insulin dependent diabetes mellitus |  |
| C108.12 | READ | 17858 | Type 1 diabetes mellitus |  |
| C108.13 | READ | 24423 | Type I diabetes mellitus |  |
| C108000 | READ | 46963 | Insulin-dependent diabetes mellitus with renal complications |  |
| C108011 | READ | 61344 | Type I diabetes mellitus with renal complications |  |
| C108012 | READ | 21983 | Type 1 diabetes mellitus with renal complications |  |
| C108100 | READ | 49276 | Insulin-dependent diabetes mellitus with ophthalmic comps |  |
| C108200 | READ | 52283 | Insulin-dependent diabetes mellitus with neurological comps |  |
| C108211 | READ | 49146 | Type I diabetes mellitus with neurological complications |  |
| C108212 | READ | 61829 | Type 1 diabetes mellitus with neurological complications |  |
| C108300 | READ | 52104 | Insulin dependent diabetes mellitus with multiple complicatn |  |
| C108400 | READ | 26855 | Unstable insulin dependent diabetes mellitus |  |
| C108411 | READ | 60107 | Unstable type I diabetes mellitus |  |
| C108500 | READ | 44443 | Insulin dependent diabetes mellitus with ulcer |  |
| C108511 | READ | 51957 | Type I diabetes mellitus with ulcer |  |
| C108600 | READ | 60499 | Insulin dependent diabetes mellitus with gangrene |  |
| C108700 | READ | 6509 | Insulin dependent diabetes mellitus with retinopathy |  |
| C108711 | READ | 38161 | Type I diabetes mellitus with retinopathy |  |
| C108712 | READ | 41049 | Type 1 diabetes mellitus with retinopathy |  |
| C108800 | READ | 6791 | Insulin dependent diabetes mellitus - poor control |  |
| C108811 | READ | 46850 | Type I diabetes mellitus - poor control |  |
| C108812 | READ | 45914 | Type 1 diabetes mellitus - poor control |  |
| C108900 | READ | 31310 | Insulin dependent diabetes maturity onset |  |
| C108911 | READ | 63017 | Type I diabetes mellitus maturity onset |  |
| C108A00 | READ | 56448 | Insulin-dependent diabetes without complication |  |
| C108B00 | READ | 24694 | Insulin dependent diabetes mellitus with mononeuropathy |  |
| C108B11 | READ | 99231 | Type I diabetes mellitus with mononeuropathy |  |
| C108C00 | READ | 41716 | Insulin dependent diabetes mellitus with polyneuropathy |  |
| C108D00 | READ | 57621 | Insulin dependent diabetes mellitus with nephropathy |  |
| C108D11 | READ | 66872 | Type I diabetes mellitus with nephropathy |  |
| C108E00 | READ | 44440 | Insulin dependent diabetes mellitus with hypoglycaemic coma |  |
| C108E11 | READ | 42729 | Type I diabetes mellitus with hypoglycaemic coma |  |
| C108E12 | READ | 70766 | Type 1 diabetes mellitus with hypoglycaemic coma |  |
| C108F00 | READ | 44260 | Insulin dependent diabetes mellitus with diabetic cataract |  |
| C108F11 | READ | 17545 | Type I diabetes mellitus with diabetic cataract |  |
| C108G00 | READ | 64446 | Insulin dependent diab mell with peripheral angiopathy |  |
| C108H00 | READ | 65616 | Insulin dependent diabetes mellitus with arthropathy |  |
| C108H11 | READ | 62352 | Type I diabetes mellitus with arthropathy |  |
| C108J00 | READ | 39809 | Insulin dependent diab mell with neuropathic arthropathy |  |
| C108J12 | READ | 18230 | Type 1 diabetes mellitus with neuropathic arthropathy |  |
| C108y00 | READ | 46290 | Other specified diabetes mellitus with multiple comps |  |
| C108z00 | READ | 64449 | Unspecified diabetes mellitus with multiple complications |  |
| C109.00 | READ | 4513 | Non-insulin dependent diabetes mellitus |  |
| C109.11 | READ | 5884 | NIDDM - Non-insulin dependent diabetes mellitus |  |
| C109.12 | READ | 17859 | Type 2 diabetes mellitus |  |
| C109.13 | READ | 18219 | Type II diabetes mellitus |  |
| C109000 | READ | 52303 | Non-insulin-dependent diabetes mellitus with renal comps |  |
| C109011 | READ | 50225 | Type II diabetes mellitus with renal complications |  |
| C109012 | READ | 18209 | Type 2 diabetes mellitus with renal complications |  |
| C109100 | READ | 50429 | Non-insulin-dependent diabetes mellitus with ophthalm comps |  |
| C109111 | READ | 59725 | Type II diabetes mellitus with ophthalmic complications |  |
| C109112 | READ | 70316 | Type 2 diabetes mellitus with ophthalmic complications |  |
| C109200 | READ | 55842 | Non-insulin-dependent diabetes mellitus with neuro comps |  |
| C109211 | READ | 67905 | Type II diabetes mellitus with neurological complications |  |
| C109212 | READ | 45919 | Type 2 diabetes mellitus with neurological complications |  |
| C109300 | READ | 62146 | Non-insulin-dependent diabetes mellitus with multiple comps |  |
| C109400 | READ | 34912 | Non-insulin dependent diabetes mellitus with ulcer |  |
| C109411 | READ | 55075 | Type II diabetes mellitus with ulcer |  |
| C109412 | READ | 65704 | Type 2 diabetes mellitus with ulcer |  |
| C109500 | READ | 40401 | Non-insulin dependent diabetes mellitus with gangrene |  |
| C109511 | READ | 62107 | Type II diabetes mellitus with gangrene |  |
| C109600 | READ | 17262 | Non-insulin-dependent diabetes mellitus with retinopathy |  |
| C109611 | READ | 58604 | Type II diabetes mellitus with retinopathy |  |
| C109612 | READ | 42762 | Type 2 diabetes mellitus with retinopathy |  |
| C109700 | READ | 8403 | Non-insulin dependent diabetes mellitus - poor control |  |
| C109711 | READ | 24458 | Type II diabetes mellitus - poor control |  |
| C109712 | READ | 45913 | Type 2 diabetes mellitus - poor control |  |
| C109800 | READ | 39406 | Reaven's syndrome |  |
| C109900 | READ | 29979 | Non-insulin-dependent diabetes mellitus without complication |  |
| C109A00 | READ | 72320 | Non-insulin dependent diabetes mellitus with mononeuropathy |  |
| C109A11 | READ | 50813 | Type II diabetes mellitus with mononeuropathy |  |
| C109B00 | READ | 45467 | Non-insulin dependent diabetes mellitus with polyneuropathy |  |
| C109B11 | READ | 47409 | Type II diabetes mellitus with polyneuropathy |  |
| C109C00 | READ | 59365 | Non-insulin dependent diabetes mellitus with nephropathy |  |
| C109C11 | READ | 64571 | Type II diabetes mellitus with nephropathy |  |
| C109C12 | READ | 24836 | Type 2 diabetes mellitus with nephropathy |  |
| C109D00 | READ | 43785 | Non-insulin dependent diabetes mellitus with hypoglyca coma |  |
| C109D11 | READ | 56268 | Type II diabetes mellitus with hypoglycaemic coma |  |
| C109D12 | READ | 61071 | Type 2 diabetes mellitus with hypoglycaemic coma |  |
| C109E00 | READ | 69278 | Non-insulin depend diabetes mellitus with diabetic cataract |  |
| C109E11 | READ | 48192 | Type II diabetes mellitus with diabetic cataract |  |
| C109E12 | READ | 44779 | Type 2 diabetes mellitus with diabetic cataract |  |
| C109F00 | READ | 54212 | Non-insulin-dependent d m with peripheral angiopath |  |
| C109F11 | READ | 54899 | Type II diabetes mellitus with peripheral angiopathy |  |
| C109F12 | READ | 60699 | Type 2 diabetes mellitus with peripheral angiopathy |  |
| C109G00 | READ | 24693 | Non-insulin dependent diabetes mellitus with arthropathy |  |
| C109G11 | READ | 18143 | Type II diabetes mellitus with arthropathy |  |
| C109G12 | READ | 49869 | Type 2 diabetes mellitus with arthropathy |  |
| C109H00 | READ | 40962 | Non-insulin dependent d m with neuropathic arthropathy |  |
| C109H11 | READ | 47816 | Type II diabetes mellitus with neuropathic arthropathy |  |
| C109H12 | READ | 66965 | Type 2 diabetes mellitus with neuropathic arthropathy |  |
| C109J00 | READ | 18278 | Insulin treated Type 2 diabetes mellitus |  |
| C109J11 | READ | 37648 | Insulin treated non-insulin dependent diabetes mellitus |  |
| C109J12 | READ | 18264 | Insulin treated Type II diabetes mellitus |  |
| C109K00 | READ | 36633 | Hyperosmolar non-ketotic state in type 2 diabetes mellitus |  |
| C10A.00 | READ | 52236 | Malnutrition-related diabetes mellitus |  |
| C10A000 | READ | 66675 | Malnutrition-related diabetes mellitus with coma |  |
| C10A100 | READ | 33969 | Malnutrition-related diabetes mellitus with ketoacidosis |  |
| C10A500 | READ | 100347 | Malnutritn-relat diabetes melitus wth periph circul complctn |  |
| C10B.00 | READ | 11551 | Diabetes mellitus induced by steroids |  |
| C10B000 | READ | 26108 | Steroid induced diabetes mellitus without complication |  |
| C10C.00 | READ | 43453 | Diabetes mellitus autosomal dominant |  |
| C10C.11 | READ | 46624 | Maturity onset diabetes in youth |  |
| C10D.00 | READ | 36695 | Diabetes mellitus autosomal dominant type 2 |  |
| C10D.11 | READ | 59991 | Maturity onset diabetes in youth type 2 |  |
| C10E.00 | READ | 1549 | Type 1 diabetes mellitus |  |
| C10E.11 | READ | 12455 | Type I diabetes mellitus |  |
| C10E.12 | READ | 51261 | Insulin dependent diabetes mellitus |  |
| C10E000 | READ | 47582 | Type 1 diabetes mellitus with renal complications |  |
| C10E100 | READ | 47649 | Type 1 diabetes mellitus with ophthalmic complications |  |
| C10E200 | READ | 42831 | Type 1 diabetes mellitus with neurological complications |  |
| C10E300 | READ | 47650 | Type 1 diabetes mellitus with multiple complications |  |
| C10E311 | READ | 91942 | Type I diabetes mellitus with multiple complications |  |
| C10E312 | READ | 45276 | Insulin dependent diabetes mellitus with multiple complicat |  |
| C10E400 | READ | 43921 | Unstable type 1 diabetes mellitus |  |
| C10E411 | READ | 49949 | Unstable type I diabetes mellitus |  |
| C10E412 | READ | 54600 | Unstable insulin dependent diabetes mellitus |  |
| C10E500 | READ | 18683 | Type 1 diabetes mellitus with ulcer |  |
| C10E511 | READ | 93878 | Type I diabetes mellitus with ulcer |  |
| C10E600 | READ | 69993 | Type 1 diabetes mellitus with gangrene |  |
| C10E700 | READ | 18387 | Type 1 diabetes mellitus with retinopathy |  |
| C10E711 | READ | 95343 | Type I diabetes mellitus with retinopathy |  |
| C10E712 | READ | 93875 | Insulin dependent diabetes mellitus with retinopathy |  |
| C10E800 | READ | 35288 | Type 1 diabetes mellitus - poor control |  |
| C10E812 | READ | 72702 | Insulin dependent diabetes mellitus - poor control |  |
| C10E900 | READ | 40682 | Type 1 diabetes mellitus maturity onset |  |
| C10EA00 | READ | 69676 | Type 1 diabetes mellitus without complication |  |
| C10EA11 | READ | 62613 | Type I diabetes mellitus without complication |  |
| C10EB00 | READ | 68105 | Type 1 diabetes mellitus with mononeuropathy |  |
| C10EC00 | READ | 46301 | Type 1 diabetes mellitus with polyneuropathy |  |
| C10EC11 | READ | 91943 | Type I diabetes mellitus with polyneuropathy |  |
| C10ED00 | READ | 10418 | Type 1 diabetes mellitus with nephropathy |  |
| C10EE00 | READ | 39070 | Type 1 diabetes mellitus with hypoglycaemic coma |  |
| C10EF00 | READ | 49554 | Type 1 diabetes mellitus with diabetic cataract |  |
| C10EG00 | READ | 93468 | Type 1 diabetes mellitus with peripheral angiopathy |  |
| C10EH00 | READ | 18642 | Type 1 diabetes mellitus with arthropathy |  |
| C10EJ00 | READ | 54008 | Type 1 diabetes mellitus with neuropathic arthropathy |  |
| C10EK00 | READ | 30323 | Type 1 diabetes mellitus with persistent proteinuria |  |
| C10EL00 | READ | 30294 | Type 1 diabetes mellitus with persistent microalbuminuria |  |
| C10EM00 | READ | 10692 | Type 1 diabetes mellitus with ketoacidosis |  |
| C10EM11 | READ | 62209 | Type I diabetes mellitus with ketoacidosis |  |
| C10EN00 | READ | 40837 | Type 1 diabetes mellitus with ketoacidotic coma |  |
| C10EN11 | READ | 66145 | Type I diabetes mellitus with ketoacidotic coma |  |
| C10EP00 | READ | 22871 | Type 1 diabetes mellitus with exudative maculopathy |  |
| C10EQ00 | READ | 55239 | Type 1 diabetes mellitus with gastroparesis |  |
| C10ER00 | READ | 95636 | Latent autoimmune diabetes mellitus in adult |  |
| C10F.00 | READ | 758 | Type 2 diabetes mellitus |  |
| C10F.11 | READ | 22884 | Type II diabetes mellitus |  |
| C10F000 | READ | 18777 | Type 2 diabetes mellitus with renal complications |  |
| C10F011 | READ | 57278 | Type II diabetes mellitus with renal complications |  |
| C10F100 | READ | 47321 | Type 2 diabetes mellitus with ophthalmic complications |  |
| C10F200 | READ | 34268 | Type 2 diabetes mellitus with neurological complications |  |
| C10F300 | READ | 65267 | Type 2 diabetes mellitus with multiple complications |  |
| C10F311 | READ | 43227 | Type II diabetes mellitus with multiple complications |  |
| C10F400 | READ | 49074 | Type 2 diabetes mellitus with ulcer |  |
| C10F411 | READ | 91646 | Type II diabetes mellitus with ulcer |  |
| C10F500 | READ | 12736 | Type 2 diabetes mellitus with gangrene |  |
| C10F600 | READ | 18496 | Type 2 diabetes mellitus with retinopathy |  |
| C10F611 | READ | 49655 | Type II diabetes mellitus with retinopathy |  |
| C10F700 | READ | 25627 | Type 2 diabetes mellitus - poor control |  |
| C10F711 | READ | 47315 | Type II diabetes mellitus - poor control |  |
| C10F800 | READ | 54773 | Reaven's syndrome |  |
| C10F811 | READ | 39481 | Metabolic syndrome X |  |
| C10F900 | READ | 47954 | Type 2 diabetes mellitus without complication |  |
| C10F911 | READ | 53392 | Type II diabetes mellitus without complication |  |
| C10FA00 | READ | 62674 | Type 2 diabetes mellitus with mononeuropathy |  |
| C10FA11 | READ | 95351 | Type II diabetes mellitus with mononeuropathy |  |
| C10FB00 | READ | 18425 | Type 2 diabetes mellitus with polyneuropathy |  |
| C10FB11 | READ | 50527 | Type II diabetes mellitus with polyneuropathy |  |
| C10FC00 | READ | 12640 | Type 2 diabetes mellitus with nephropathy |  |
| C10FC11 | READ | 102201 | Type II diabetes mellitus with nephropathy |  |
| C10FD00 | READ | 46917 | Type 2 diabetes mellitus with hypoglycaemic coma |  |
| C10FE00 | READ | 44982 | Type 2 diabetes mellitus with diabetic cataract |  |
| C10FE11 | READ | 93727 | Type II diabetes mellitus with diabetic cataract |  |
| C10FF00 | READ | 37806 | Type 2 diabetes mellitus with peripheral angiopathy |  |
| C10FG00 | READ | 59253 | Type 2 diabetes mellitus with arthropathy |  |
| C10FH00 | READ | 35385 | Type 2 diabetes mellitus with neuropathic arthropathy |  |
| C10FJ00 | READ | 1407 | Insulin treated Type 2 diabetes mellitus |  |
| C10FJ11 | READ | 64668 | Insulin treated Type II diabetes mellitus |  |
| C10FK00 | READ | 34450 | Hyperosmolar non-ketotic state in type 2 diabetes mellitus |  |
| C10FL00 | READ | 26054 | Type 2 diabetes mellitus with persistent proteinuria |  |
| C10FL11 | READ | 60796 | Type II diabetes mellitus with persistent proteinuria |  |
| C10FM00 | READ | 18390 | Type 2 diabetes mellitus with persistent microalbuminuria |  |
| C10FM11 | READ | 85991 | Type II diabetes mellitus with persistent microalbuminuria |  |
| C10FN00 | READ | 32627 | Type 2 diabetes mellitus with ketoacidosis |  |
| C10FP00 | READ | 51756 | Type 2 diabetes mellitus with ketoacidotic coma |  |
| C10FQ00 | READ | 25591 | Type 2 diabetes mellitus with exudative maculopathy |  |
| C10FR00 | READ | 63690 | Type 2 diabetes mellitus with gastroparesis |  |
| C10FS00 | READ | 95539 | Maternally inherited diabetes mellitus |  |
| C10G.00 | READ | 51697 | Secondary pancreatic diabetes mellitus |  |
| C10G000 | READ | 96506 | Secondary pancreatic diabetes mellitus without complication |  |
| C10H.00 | READ | 61122 | Diabetes mellitus induced by non-steroid drugs |  |
| C10H000 | READ | 67212 | DM induced by non-steroid drugs without complication |  |
| C10J.00 | READ | 68517 | Insulin autoimmune syndrome |  |
| C10K.00 | READ | 37957 | Type A insulin resistance |  |
| C10K000 | READ | 56885 | Type A insulin resistance without complication |  |
| C10M.00 | READ | 43857 | Lipoatrophic diabetes mellitus |  |
| C10N.00 | READ | 22487 | Secondary diabetes mellitus |  |
| C10N100 | READ | 93380 | Cystic fibrosis related diabetes mellitus |  |
| C10y.00 | READ | 33343 | Diabetes mellitus with other specified manifestation |  |
| C10y100 | READ | 63371 | Diabetes mellitus, adult, + other specified manifestation |  |
| C10yy00 | READ | 10098 | Other specified diabetes mellitus with other spec comps |  |
| C10yz00 | READ | 70821 | Diabetes mellitus NOS with other specified manifestation |  |
| C10z.00 | READ | 45491 | Diabetes mellitus with unspecified complication |  |
| C10z000 | READ | 68792 | Diabetes mellitus, juvenile type, + unspecified complication |  |
| C10z100 | READ | 63762 | Diabetes mellitus, adult onset, + unspecified complication |  |
| C10zy00 | READ | 64283 | Other specified diabetes mellitus with unspecified comps |  |
| C10zz00 | READ | 64357 | Diabetes mellitus NOS with unspecified complication |  |

| risk_factor definition 4: Diabetes_monitoring |
| --- |
| n= 86 medcodes used |

| **Read_code** | **readoxmisflag** | **medcode** | **desc** |  |
| --- | --- | --- | --- | --- |
| 66A..00 | READ | 3550 | Diabetic monitoring |  |
| 66A1.00 | READ | 13070 | Initial diabetic assessment |  |
| 66A2.00 | READ | 608 | Follow-up diabetic assessment |  |
| 66A3.00 | READ | 7563 | Diabetic on diet only |  |
| 66A4.00 | READ | 1684 | Diabetic on oral treatment |  |
| 66A5.00 | READ | 8842 | Diabetic on insulin |  |
| 66A6.00 | READ | 13068 | Last hypo. attack |  |
| 66A7.00 | READ | 13281 | Frequency of hypo. attacks |  |
| 66A7000 | READ | 31752 | Frequency of hospital treated hypoglycaemia |  |
| 66A7100 | READ | 40363 | Frequency of GP or paramedic treated hypoglycaemia |  |
| 66A8.00 | READ | 13069 | Has seen dietician - diabetes |  |
| 66A9.00 | READ | 38078 | Understands diet - diabetes |  |
| 66AA.00 | READ | 2319 | Injection sites |  |
| 66AA.11 | READ | 20696 | Injection sites - diabetic |  |
| 66AB.00 | READ | 26510 | Urine sugar charts |  |
| 66AC.00 | READ | 13193 | Blood sugar charts |  |
| 66AD.00 | READ | 13196 | Fundoscopy - diabetic check |  |
| 66AE.00 | READ | 13291 | Feet examination |  |
| 66AF.00 | READ | 17861 | Attends out-patients |  |
| 66AG.00 | READ | 53238 | Diabetic drug side effects |  |
| 66AH.00 | READ | 16490 | Diabetic treatment changed |  |
| 66AH000 | READ | 11047 | Conversion to insulin |  |
| 66AI.00 | READ | 13071 | Diabetic - good control |  |
| 66AJ.00 | READ | 2378 | Diabetic - poor control |  |
| 66AJ.11 | READ | 9013 | Unstable diabetes |  |
| 66AJ000 | READ | 22959 | Chronic hyperglycaemia |  |
| 66AJ100 | READ | 2478 | Brittle diabetes |  |
| 66AJ200 | READ | 21420 | Loss of hypoglycaemic warning |  |
| 66AJ300 | READ | 37625 | Recurrent severe hypos |  |
| 66AJz00 | READ | 22023 | Diabetic - poor control NOS |  |
| 66AK.00 | READ | 43951 | Diabetic - cooperative patient |  |
| 66AL.00 | READ | 17869 | Diabetic-uncooperative patient |  |
| 66AM.00 | READ | 17886 | Diabetic - follow-up default |  |
| 66AN.00 | READ | 29041 | Date diabetic treatment start |  |
| 66AO.00 | READ | 55123 | Date diabetic treatment stopp. |  |
| 66AP.00 | READ | 12506 | Diabetes: practice programme |  |
| 66AQ.00 | READ | 12675 | Diabetes: shared care programme |  |
| 66AR.00 | READ | 8836 | Diabetes management plan given |  |
| 66AS.00 | READ | 6125 | Diabetic annual review |  |
| 66AT.00 | READ | 18167 | Annual diabetic blood test |  |
| 66AU.00 | READ | 12307 | Diabetes care by hospital only |  |
| 66AV.00 | READ | 28769 | Diabetic on insulin and oral treatment |  |
| 66AW.00 | READ | 50175 | Diabetic foot risk assessment |  |
| 66AX.00 | READ | 46577 | Diabetes: shared care in pregnancy - diabetol and obstet |  |
| 66AY.00 | READ | 26604 | Diabetic diet - good compliance |  |
| 66AZ.00 | READ | 13067 | Diabetic monitoring NOS |  |
| 66Aa.00 | READ | 25636 | Diabetic diet - poor compliance |  |
| 66Ab.00 | READ | 22823 | Diabetic foot examination |  |
| 66Ac.00 | READ | 10977 | Diabetic peripheral neuropathy screening |  |
| 66Ad.00 | READ | 18583 | Hypoglycaemic attack requiring 3rd party assistance |  |
| 66Ae.00 | READ | 37035 | HbA1c target |  |
| 66Af.00 | READ | 32619 | Patient diabetes education review |  |
| 66Ag.00 | READ | 90301 | Insulin needles changed daily |  |
| 66Ah.00 | READ | 66274 | Insulin needles changed for each injection |  |
| 66Ai.00 | READ | 28873 | Diabetic 6 month review |  |
| 66Aj.00 | READ | 69152 | Insulin needles changed less than once a day |  |
| 66Ak.00 | READ | 66475 | Diabetic monitoring - lower risk albumin excretion |  |
| 66Al.00 | READ | 61470 | Diabetic monitoring - higher risk albumin excretion |  |
| 66Am.00 | READ | 83485 | Insulin dose changed |  |
| 66An.00 | READ | 85660 | Diabetes type 1 review |  |
| 66Ao.00 | READ | 83532 | Diabetes type 2 review |  |
| 9OL..00 | READ | 9897 | Diabetes monitoring admin. |  |
| 9OL..11 | READ | 13191 | Diabetes clinic administration |  |
| 9OL1.00 | READ | 13197 | Attends diabetes monitoring |  |
| 9OL2.00 | READ | 26603 | Refuses diabetes monitoring |  |
| 9OL3.00 | READ | 22130 | Diabetes monitoring default |  |
| 9OL4.00 | READ | 13194 | Diabetes monitoring 1st letter |  |
| 9OL5.00 | READ | 13195 | Diabetes monitoring 2nd letter |  |
| 9OL6.00 | READ | 12030 | Diabetes monitoring 3rd letter |  |
| 9OL7.00 | READ | 31240 | Diabetes monitor.verbal invite |  |
| 9OL8.00 | READ | 31141 | Diabetes monitor.phone invite |  |
| 9OL9.00 | READ | 54846 | Diabetes monitoring deleted |  |
| 9OLA.00 | READ | 13192 | Diabetes monitor. check done |  |
| 9OLA.11 | READ | 20900 | Diabetes monitored |  |
| 9OLB.00 | READ | 26605 | Attended diabetes structured education programme |  |
| 9OLC.00 | READ | 51066 | Family/carer attended diabetes structured education prog |  |
| 9OLD.00 | READ | 35383 | Diabetic patient unsuitable for digital retinal photography |  |
| 9OLE.00 | READ | 93530 | Attended DESMOND structured programme |  |
| 9OLF.00 | READ | 94186 | Diabetes structured education programme completed |  |
| 9OLG.00 | READ | 94011 | Attended XPERT diabetes structured education programme |  |
| 9OLH.00 | READ | 93390 | Attended DAFNE diabetes structured education programme |  |
| 9OLJ.00 | READ | 93491 | DAFNE diabetes structured education programme completed |  |
| 9OLK.00 | READ | 93529 | DESMOND diabetes structured education programme completed |  |
| 9OLL.00 | READ | 93631 | XPERT diabetes structured education programme completed |  |
| 9OLM.00 | READ | 93854 | Diabetes structured education programme declined |  |
| 9OLZ.00 | READ | 31241 | Diabetes monitoring admin.NOS |  |

| risk_factor definition 5: Liver disease |
| --- |
| n= 102 medcodes used |

| **Read_code** | **readoxmisflag** | **medcode** | **desc** |  |
| --- | --- | --- | --- | --- |
| J60..00 | READ | 48488 | Acute and subacute liver necrosis |  |
| J600.00 | READ | 41480 | Acute necrosis of liver |  |
| J600000 | READ | 6690 | Acute hepatic failure |  |
| J600011 | READ | 39945 | Acute liver failure |  |
| J600100 | READ | 15855 | Acute hepatitis - noninfective |  |
| J600200 | READ | 53704 | Acute yellow atrophy |  |
| J600z00 | READ | 55637 | Acute necrosis of liver NOS |  |
| J601.00 | READ | 57324 | Subacute necrosis of liver |  |
| J601000 | READ | 26490 | Subacute hepatic failure |  |
| J601100 | READ | 22168 | Subacute hepatitis - noninfective |  |
| J601200 | READ | 69313 | Subacute yellow atrophy |  |
| J601z00 | READ | 69367 | Subacute necrosis of liver NOS |  |
| J60z.00 | READ | 65067 | Acute and subacute liver necrosis NOS |  |
| J61..00 | READ | 6863 | Cirrhosis and chronic liver disease |  |
| J610.00 | READ | 10691 | Alcoholic fatty liver |  |
| J611.00 | READ | 3216 | Acute alcoholic hepatitis |  |
| J612.00 | READ | 4743 | Alcoholic cirrhosis of liver |  |
| J612.11 | READ | 68376 | Florid cirrhosis |  |
| J612.12 | READ | 100474 | Laennec's cirrhosis |  |
| J612000 | READ | 21713 | Alcoholic fibrosis and sclerosis of liver |  |
| J613.00 | READ | 7885 | Alcoholic liver damage unspecified |  |
| J613000 | READ | 17330 | Alcoholic hepatic failure |  |
| J614.00 | READ | 1754 | Chronic hepatitis |  |
| J614000 | READ | 23578 | Chronic persistent hepatitis |  |
| J614100 | READ | 9029 | Chronic active hepatitis |  |
| J614111 | READ | 7957 | Autoimmune chronic active hepatitis |  |
| J614200 | READ | 1755 | Chronic aggressive hepatitis |  |
| J614300 | READ | 53480 | Recurrent hepatitis |  |
| J614400 | READ | 66534 | Chronic lobular hepatitis |  |
| J614y00 | READ | 53877 | Chronic hepatitis unspecified |  |
| J614z00 | READ | 15489 | Chronic hepatitis NOS |  |
| J615.00 | READ | 16725 | Cirrhosis - non alcoholic |  |
| J615.11 | READ | 47257 | Portal cirrhosis |  |
| J615100 | READ | 69204 | Multilobular portal cirrhosis |  |
| J615300 | READ | 3450 | Diffuse nodular cirrhosis |  |
| J615400 | READ | 44676 | Fatty portal cirrhosis |  |
| J615500 | READ | 92909 | Hypertrophic portal cirrhosis |  |
| J615600 | READ | 40567 | Capsular portal cirrhosis |  |
| J615700 | READ | 27438 | Cardiac portal cirrhosis |  |
| J615800 | READ | 96664 | Juvenile portal cirrhosis |  |
| J615812 | READ | 58184 | Indian childhood cirrhosis |  |
| J615C00 | READ | 100253 | Xanthomatous portal cirrhosis |  |
| J615D00 | READ | 73482 | Bacterial portal cirrhosis |  |
| J615H00 | READ | 48928 | Infectious cirrhosis NOS |  |
| J615y00 | READ | 55454 | Portal cirrhosis unspecified |  |
| J615z00 | READ | 16455 | Non-alcoholic cirrhosis NOS |  |
| J615z11 | READ | 22841 | Macronodular cirrhosis of liver |  |
| J615z12 | READ | 18739 | Cryptogenic cirrhosis of liver |  |
| J615z13 | READ | 1638 | Cirrhosis of liver NOS |  |
| J615z15 | READ | 71453 | Hepatic fibrosis |  |
| J616.00 | READ | 9494 | Biliary cirrhosis |  |
| J616000 | READ | 5638 | Primary biliary cirrhosis |  |
| J616100 | READ | 15424 | Secondary biliary cirrhosis |  |
| J616200 | READ | 91591 | Biliary cirrhosis of children |  |
| J616z00 | READ | 58630 | Biliary cirrhosis NOS |  |
| J617.00 | READ | 7943 | Alcoholic hepatitis |  |
| J617000 | READ | 7602 | Chronic alcoholic hepatitis |  |
| J61y.00 | READ | 42843 | Other non-alcoholic chronic liver disease |  |
| J61y100 | READ | 10234 | Non-alcoholic fatty liver |  |
| J61y200 | READ | 1780 | Hepatosplenomegaly |  |
| J61y300 | READ | 40963 | Portal fibrosis without cirrhosis |  |
| J61y400 | READ | 25383 | Hepatic fibrosis |  |
| J61y500 | READ | 60104 | Hepatic sclerosis |  |
| J61y600 | READ | 100592 | Hepatic fibrosis with hepatic sclerosis |  |
| J61y700 | READ | 10572 | Steatosis of liver |  |
| J61yz00 | READ | 33597 | Other non-alcoholic chronic liver disease NOS |  |
| J61z.00 | READ | 10539 | Chronic liver disease NOS |  |
| J62..00 | READ | 31897 | Liver abscess and sequelae of chronic liver disease |  |
| J620.00 | READ | 46023 | Liver abscess - excluding amoebic liver abscess |  |
| J620000 | READ | 68692 | Liver abscess due to portal pyaemia |  |
| J620100 | READ | 25341 | Liver abscess due to cholangitis |  |
| J620300 | READ | 70524 | Liver abscess via umbilicus |  |
| J620z00 | READ | 4454 | Liver abscess NOS |  |
| J621.00 | READ | 63404 | Portal pyaemia |  |
| J621.11 | READ | 46278 | Phlebitis of portal vein |  |
| J622.00 | READ | 23511 | Hepatic coma |  |
| J622.11 | READ | 22411 | Encephalopathy - hepatic |  |
| J623.00 | READ | 5129 | Portal hypertension |  |
| J624.00 | READ | 10636 | Hepatorenal syndrome |  |
| J625.00 | READ | 24901 | [X] Hepatic failure |  |
| J625.11 | READ | 21769 | [X] Liver failure |  |
| J62y.00 | READ | 48102 | Other sequelae of chronic liver disease |  |
| J62y.11 | READ | 56070 | Hepatic failure NOS |  |
| J62y.12 | READ | 23775 | Liver failure NOS |  |
| J62y.13 | READ | 16062 | Hepatic failure |  |
| PB61.00 | READ | 25597 | Biliary atresia |  |
| PB61000 | READ | 16417 | Congenital absence of bile duct |  |
| PB61200 | READ | 49479 | Congenital obstruction of bile duct |  |
| PB61300 | READ | 95810 | Congenital stricture of bile duct |  |
| PB61311 | READ | 32803 | Congenital stricture of common bile duct |  |
| PB61400 | READ | 38389 | Atresia of bile duct |  |
| PB61411 | READ | 65889 | Intrahepatic atresia of bile duct |  |
| PB61412 | READ | 50458 | Extrahepatic atresia of bile duct |  |
| PB61500 | READ | 48269 | Congenital absence of hepatic ducts |  |
| PB61600 | READ | 50400 | Atresia of hepatic ducts |  |
| PB61z00 | READ | 50444 | Biliary atresia NOS |  |
| PB63000 | READ | 42256 | Congenital absence of gallbladder |  |
| PB63011 | READ | 47576 | Agenesis of gallbladder |  |
| PB63100 | READ | 98402 | Congenital absence of liver lobe |  |
| PB63300 | READ | 20610 | Riedel's lobe liver |  |
| PB63500 | READ | 40029 | Alagille syndrome |  |
| PB6y100 | READ | 48738 | Congenital hepatomegaly |  |

| risk_factor definition 6: Stroke_TIA |
| --- |
| n= 81 medcodes used |

| **Read_code** | **readoxmisflag** | **medcode** | **desc** |  |
| --- | --- | --- | --- | --- |
| G61..00 | READ | 5051 | Intracerebral haemorrhage |  |
| G61..11 | READ | 6960 | CVA - cerebrovascular accid due to intracerebral haemorrhage |  |
| G61..12 | READ | 18604 | Stroke due to intracerebral haemorrhage |  |
| G610.00 | READ | 31595 | Cortical haemorrhage |  |
| G611.00 | READ | 40338 | Internal capsule haemorrhage |  |
| G612.00 | READ | 46316 | Basal nucleus haemorrhage |  |
| G613.00 | READ | 13564 | Cerebellar haemorrhage |  |
| G614.00 | READ | 7912 | Pontine haemorrhage |  |
| G615.00 | READ | 62342 | Bulbar haemorrhage |  |
| G616.00 | READ | 30045 | External capsule haemorrhage |  |
| G617.00 | READ | 30202 | Intracerebral haemorrhage, intraventricular |  |
| G618.00 | READ | 57315 | Intracerebral haemorrhage, multiple localized |  |
| G61X.00 | READ | 31060 | Intracerebral haemorrhage in hemisphere, unspecified |  |
| G61X000 | READ | 28314 | Left sided intracerebral haemorrhage, unspecified |  |
| G61X100 | READ | 19201 | Right sided intracerebral haemorrhage, unspecified |  |
| G61z.00 | READ | 3535 | Intracerebral haemorrhage NOS |  |
| G63y000 | READ | 23671 | Cerebral infarct due to thrombosis of precerebral arteries |  |
| G63y100 | READ | 24446 | Cerebral infarction due to embolism of precerebral arteries |  |
| G64..00 | READ | 8837 | Cerebral arterial occlusion |  |
| G64..11 | READ | 5363 | CVA - cerebral artery occlusion |  |
| G64..12 | READ | 569 | Infarction - cerebral |  |
| G64..13 | READ | 6155 | Stroke due to cerebral arterial occlusion |  |
| G640.00 | READ | 16517 | Cerebral thrombosis |  |
| G640000 | READ | 36717 | Cerebral infarction due to thrombosis of cerebral arteries |  |
| G641.00 | READ | 15019 | Cerebral embolism |  |
| G641.11 | READ | 34758 | Cerebral embolus |  |
| G641000 | READ | 27975 | Cerebral infarction due to embolism of cerebral arteries |  |
| G64z.00 | READ | 3149 | Cerebral infarction NOS |  |
| G64z.11 | READ | 15252 | Brainstem infarction NOS |  |
| G64z.12 | READ | 5602 | Cerebellar infarction |  |
| G64z000 | READ | 25615 | Brainstem infarction |  |
| G64z100 | READ | 47642 | Wallenberg syndrome |  |
| G64z111 | READ | 5185 | Lateral medullary syndrome |  |
| G64z200 | READ | 9985 | Left sided cerebral infarction |  |
| G64z300 | READ | 10504 | Right sided cerebral infarction |  |
| G64z400 | READ | 26424 | Infarction of basal ganglia |  |
| G65..00 | READ | 504 | Transient cerebral ischaemia |  |
| G65..11 | READ | 3132 | Drop attack |  |
| G65..12 | READ | 1433 | Transient ischaemic attack |  |
| G65..13 | READ | 2417 | Vertebro-basilar insufficiency |  |
| G650.00 | READ | 23942 | Basilar artery syndrome |  |
| G650.11 | READ | 5268 | Insufficiency - basilar artery |  |
| G651.00 | READ | 33377 | Vertebral artery syndrome |  |
| G651000 | READ | 21118 | Vertebro-basilar artery syndrome |  |
| G652.00 | READ | 23465 | Subclavian steal syndrome |  |
| G653.00 | READ | 44765 | Carotid artery syndrome hemispheric |  |
| G654.00 | READ | 50594 | Multiple and bilateral precerebral artery syndromes |  |
| G655.00 | READ | 6489 | Transient global amnesia |  |
| G656.00 | READ | 10794 | Vertebrobasilar insufficiency |  |
| G65y.00 | READ | 19354 | Other transient cerebral ischaemia |  |
| G65z.00 | READ | 1895 | Transient cerebral ischaemia NOS |  |
| G65z000 | READ | 55247 | Impending cerebral ischaemia |  |
| G65z100 | READ | 16507 | Intermittent cerebral ischaemia |  |
| G65zz00 | READ | 15788 | Transient cerebral ischaemia NOS |  |
| G66..00 | READ | 1469 | Stroke and cerebrovascular accident unspecified |  |
| G66..11 | READ | 1298 | CVA unspecified |  |
| G66..12 | READ | 6253 | Stroke unspecified |  |
| G66..13 | READ | 6116 | CVA - Cerebrovascular accident unspecified |  |
| G660.00 | READ | 18689 | Middle cerebral artery syndrome |  |
| G661.00 | READ | 19280 | Anterior cerebral artery syndrome |  |
| G662.00 | READ | 19260 | Posterior cerebral artery syndrome |  |
| G663.00 | READ | 8443 | Brain stem stroke syndrome |  |
| G664.00 | READ | 17322 | Cerebellar stroke syndrome |  |
| G665.00 | READ | 33499 | Pure motor lacunar syndrome |  |
| G666.00 | READ | 51767 | Pure sensory lacunar syndrome |  |
| G667.00 | READ | 7780 | Left sided CVA |  |
| G668.00 | READ | 12833 | Right sided CVA |  |
| G669.00 | READ | 16956 | Cerebral palsy, not congenital or infantile, acute |  |
| G676000 | READ | 39344 | Cereb infarct due cerebral venous thrombosis, nonpyogenic |  |
| G6W..00 | READ | 40758 | Cereb infarct due unsp occlus/stenos precerebr arteries |  |
| G6X..00 | READ | 33543 | Cerebrl infarctn due/unspcf occlusn or sten/cerebrl artrs |  |
| Gyu6.00 | READ | 73901 | [X]Cerebrovascular diseases |  |
| Gyu6100 | READ | 65745 | [X]Other subarachnoid haemorrhage |  |
| Gyu6200 | READ | 53810 | [X]Other intracerebral haemorrhage |  |
| Gyu6300 | READ | 91627 | [X]Cerebrl infarctn due/unspcf occlusn or sten/cerebrl artrs |  |
| Gyu6400 | READ | 53745 | [X]Other cerebral infarction |  |
| Gyu6500 | READ | 90572 | [X]Occlusion and stenosis of other precerebral arteries |  |
| Gyu6600 | READ | 92036 | [X]Occlusion and stenosis of other cerebral arteries |  |
| Gyu6A00 | READ | 99367 | [X]Other cerebrovascular disorders in diseases CE |  |
| Gyu6F00 | READ | 96630 | [X]Intracerebral haemorrhage in hemisphere, unspecified |  |
| Gyu6G00 | READ | 94482 | [X]Cereb infarct due unsp occlus/stenos precerebr arteries |  |

| risk_factor definition 7: Central nervous sytem disease |
| --- |
| n= 291 medcodes used |

| **Read_code** | **readoxmisflag** | **medcode** | **desc** |  |
| --- | --- | --- | --- | --- |
| F1...00 | READ | 34092 | Hereditary and degenerative diseases of the CNS |  |
| F10..00 | READ | 56288 | Cerebral degenerations usually manifest in childhood |  |
| F100.00 | READ | 43203 | Leucodystrophy |  |
| F100000 | READ | 45903 | Krabbe's disease |  |
| F100100 | READ | 97092 | Schulz's disease |  |
| F100200 | READ | 53382 | Pelizaeus-Merzbacher disease |  |
| F100300 | READ | 59855 | Metachromatic leucodystrophy |  |
| F100z00 | READ | 59035 | Leucodystrophy NOS |  |
| F101.00 | READ | 53365 | Cerebral lipidoses |  |
| F101.11 | READ | 73990 | Amaurotic familial idiocy |  |
| F101200 | READ | 56951 | Spielmeyer-Vogt (Batten) disease |  |
| F101211 | READ | 25268 | Batten's disease of retina |  |
| F101300 | READ | 21169 | Tay-Sach's disease |  |
| F101400 | READ | 49181 | Gangliosidosis |  |
| F101500 | READ | 41000 | Retinal dystrophy in cerebroretinal lipidosis |  |
| F101600 | READ | 93963 | Sandhoff disease |  |
| F102100 | READ | 65343 | Cerebral degeneration in Niemann-Pick disease |  |
| F103.00 | READ | 93372 | Cerebral degeneration in diseases EC |  |
| F103000 | READ | 63652 | Cerebral degeneration in Hunter's disease |  |
| F103100 | READ | 67762 | Cerebral degeneration in mucopolysaccharidoses |  |
| F10y000 | READ | 48211 | Alper's disease |  |
| F10y100 | READ | 48300 | Leigh's disease |  |
| F10z.00 | READ | 59956 | Childhood cerebral degenerations NOS |  |
| F11..00 | READ | 31892 | Other cerebral degenerations |  |
| F110.00 | READ | 1917 | Alzheimer's disease |  |
| F110000 | READ | 16797 | Alzheimer's disease with early onset |  |
| F110100 | READ | 32057 | Alzheimer's disease with late onset |  |
| F111.00 | READ | 11136 | Pick's disease |  |
| F112.00 | READ | 29512 | Senile degeneration of brain |  |
| F113.00 | READ | 4675 | Acquired communicating hydrocephalus |  |
| F113000 | READ | 10288 | Normal pressure hydrocephalus |  |
| F113011 | READ | 50976 | Low pressure hydrocephalus |  |
| F113z00 | READ | 70569 | Communicating hydrocephalus - acquired NOS |  |
| F114.00 | READ | 15388 | Acquired obstructive hydrocephalus |  |
| F115.00 | READ | 3584 | Hydrocephalus |  |
| F116.00 | READ | 7572 | Lewy body disease |  |
| F117.00 | READ | 45734 | Infantile posthaemorrhagic hydrocephalus |  |
| F11X.00 | READ | 54085 | Post-traumatic hydrocephalus, unspecified |  |
| F11x.00 | READ | 97422 | Cerebral degeneration in other disease EC |  |
| F11x000 | READ | 47555 | Cerebral degeneration due to alcoholism |  |
| F11x011 | READ | 36748 | Alcoholic encephalopathy |  |
| F11x200 | READ | 54744 | Cerebral degeneration due to cerebrovascular disease |  |
| F11x400 | READ | 70957 | Cerebral degeneration due to neoplastic disease |  |
| F11x500 | READ | 47658 | Cerebral degeneration due to myxoedema |  |
| F11x600 | READ | 24581 | Cerebral degeneration due to vitamin B12 deficiency |  |
| F11x700 | READ | 48531 | Cerebral degeneration due to Jakob - Creutzfeldt disease |  |
| F11x800 | READ | 99684 | Cerebral degeneration due to multifocal leucoencephalopathy |  |
| F11xz00 | READ | 44592 | Cerebral degeneration other disease NOS |  |
| F11y.00 | READ | 34976 | Other cerebral degeneration |  |
| F11y000 | READ | 33353 | Reye's syndrome |  |
| F11y100 | READ | 15261 | Cerebral ataxia |  |
| F11yz00 | READ | 31524 | Other cerebral degeneration NOS |  |
| F11z.00 | READ | 5651 | Cerebral degeneration NOS |  |
| F11z.11 | READ | 2731 | Cerebral atrophy |  |
| F12..00 | READ | 4321 | Parkinson's disease |  |
| F120.00 | READ | 1691 | Paralysis agitans |  |
| F121.00 | READ | 33544 | Parkinsonism secondary to drugs |  |
| F121.11 | READ | 19478 | Drug induced parkinsonism |  |
| F122.00 | READ | 5443 | Malignant neuroleptic syndrome |  |
| F123.00 | READ | 51105 | Postencephalitic parkinsonism |  |
| F12W.00 | READ | 24001 | Secondary parkinsonism due to other external agents |  |
| F12X.00 | READ | 26181 | Secondary parkinsonism, unspecified |  |
| F12z.00 | READ | 14912 | Parkinson's disease NOS |  |
| F13..00 | READ | 35006 | Other extrapyramidal disease and abnormal movement disorders |  |
| F13..11 | READ | 28170 | Extrapyramidal disease excluding Parkinson's disease |  |
| F130.00 | READ | 21863 | Other basal ganglia degenerative diseases |  |
| F130000 | READ | 51502 | Dejerine-Thomas syndrome |  |
| F130100 | READ | 55636 | Hallervorden-Spatz disease |  |
| F130200 | READ | 49203 | Striatonigral degeneration |  |
| F130300 | READ | 8956 | Parkinsonism with orthostatic hypotension |  |
| F130400 | READ | 40553 | Progressive supranuclear ophthalmoplegia |  |
| F130500 | READ | 35839 | Shy-Drager syndrome |  |
| F130600 | READ | 47974 | Aicardi Goutieres syndrome |  |
| F130z00 | READ | 50762 | Other basal ganglia degenerative disease NOS |  |
| F131.00 | READ | 6948 | Essential and other specified forms of tremor |  |
| F131000 | READ | 3051 | Benign essential tremor |  |
| F131100 | READ | 2512 | Familial tremor |  |
| F131200 | READ | 52748 | Drug-induced tremor |  |
| F131z00 | READ | 34349 | Essential and other specified forms of tremor NOS |  |
| F132.00 | READ | 5545 | Myoclonus |  |
| F132000 | READ | 52420 | Familial essential myoclonus |  |
| F132100 | READ | 37644 | Progressive myoclonic epilepsy |  |
| F132111 | READ | 63826 | Unverricht - Lundborg disease |  |
| F132200 | READ | 45602 | Myoclonic encephalopathy |  |
| F132300 | READ | 19015 | Myoclonic jerks |  |
| F132y00 | READ | 28281 | Other specified myoclonus |  |
| F132y11 | READ | 97376 | Paramyoclonus multiplex |  |
| F132z00 | READ | 37897 | Myoclonus NOS |  |
| F132z12 | READ | 8487 | Myoclonic seizure |  |
| F133.00 | READ | 56972 | Tics of organic origin |  |
| F134.00 | READ | 3591 | Huntington's chorea |  |
| F135.00 | READ | 6889 | Other choreas |  |
| F135000 | READ | 4108 | Hemiballismus |  |
| F135100 | READ | 2685 | Paroxysmal choreo-athetosis |  |
| F135200 | READ | 34327 | Drug-induced chorea |  |
| F135z00 | READ | 27450 | Other choreas NOS |  |
| F136.00 | READ | 11881 | Idiopathic torsion dystonia |  |
| F136000 | READ | 62243 | Idiopathic familial dystonia |  |
| F137.00 | READ | 27967 | Symptomatic torsion dystonia |  |
| F137.11 | READ | 73943 | Athetoid cerebral palsy |  |
| F137.12 | READ | 42406 | Athetosis - congenital |  |
| F137000 | READ | 16977 | Athetoid cerebral palsy |  |
| F137011 | READ | 64561 | Vogt's disease |  |
| F137100 | READ | 92598 | Double athetosis |  |
| F137111 | READ | 66314 | Congenital athetosis |  |
| F137200 | READ | 27655 | Drug-induced dystonia |  |
| F137y00 | READ | 25777 | Other specified symptomatic torsion dystonia |  |
| F137z00 | READ | 62081 | Symptomatic torsion dystonia NOS |  |
| F138.00 | READ | 50078 | Fragments of torsion dystonia |  |
| F138000 | READ | 2829 | Blepharospasm |  |
| F138100 | READ | 23356 | Orofacial dyskinesia |  |
| F138111 | READ | 5094 | Tardive dyskinesia |  |
| F138200 | READ | 2005 | Spasmodic torticollis |  |
| F138300 | READ | 21268 | Organic writers' cramp |  |
| F138z00 | READ | 71249 | Fragments of torsion dystonia NOS |  |
| F139.00 | READ | 63182 | Paroxysmal dyskinesia |  |
| F139100 | READ | 67242 | Paroxysmal kinesigenic dyskinesia |  |
| F13A.00 | READ | 51777 | Paroxysmal dystonia |  |
| F13B.00 | READ | 94690 | Myoclonic dystonia |  |
| F13X.00 | READ | 2006 | Dystonia, unspecified |  |
| F13z.00 | READ | 6787 | Other/unspecified extrapyramidal/abnormal movement disorders |  |
| F13z000 | READ | 33868 | Unspecified extrapyramidal disease |  |
| F13z100 | READ | 25880 | Stiff-man syndrome |  |
| F13z200 | READ | 6275 | Restless legs syndrome |  |
| F13z300 | READ | 50476 | Akinetic rigid syndrome |  |
| F13z400 | READ | 62421 | Hyperekplexia |  |
| F13z500 | READ | 30742 | Benign neonatal sleep myoclonus |  |
| F13zz00 | READ | 36319 | Extrapyramidal disease and abnormal movement disorder NOS |  |
| F14..00 | READ | 21216 | Spinocerebellar disease |  |
| F14..11 | READ | 8692 | Cerebellar disease |  |
| F140.00 | READ | 4165 | Friedreich's ataxia |  |
| F141.00 | READ | 3514 | Hereditary spastic paraplegia |  |
| F142.00 | READ | 5128 | Primary cerebellar degeneration |  |
| F142000 | READ | 99763 | Marie's cerebellar ataxia |  |
| F142200 | READ | 49737 | Dyssynergia cerebellaris myoclonica |  |
| F142z00 | READ | 58772 | Primary cerebellar degeneration NOS |  |
| F143.00 | READ | 2336 | Cerebellar ataxia NOS |  |
| F144.00 | READ | 93137 | Cerebellar ataxia in diseases EC |  |
| F144000 | READ | 33839 | Cerebellar ataxia due to alcoholism |  |
| F144200 | READ | 41622 | Cerebellar ataxia due to neoplasia |  |
| F144z00 | READ | 20206 | Cerebellar ataxia in disease NOS |  |
| F145.00 | READ | 50096 | Congenital nonprogressive ataxia |  |
| F14y.00 | READ | 52795 | Other spinocerebellar diseases |  |
| F14y000 | READ | 73583 | Ataxia-telangiectasia |  |
| F14y011 | READ | 16903 | Louis - Bar syndrome |  |
| F14y100 | READ | 73584 | Corticostriatal-spinal degeneration |  |
| F14yz00 | READ | 57847 | Other spinocerebellar disease NOS |  |
| F14z.00 | READ | 27331 | Spinocerebellar disease NOS |  |
| F15..00 | READ | 21889 | Anterior horn cell disease |  |
| F150.00 | READ | 33334 | Werdnig - Hoffmann disease |  |
| F150.11 | READ | 95615 | Infantile spinal muscular atrophy |  |
| F151.00 | READ | 9179 | Spinal muscular atrophy |  |
| F151000 | READ | 70572 | Unspecified spinal muscular atrophy |  |
| F151100 | READ | 43394 | Kugelberg - Welander disease |  |
| F151111 | READ | 101222 | Juvenile spinal muscular atrophy |  |
| F151200 | READ | 66575 | Adult spinal muscular atrophy |  |
| F151300 | READ | 70109 | X-linked bulbo-spinal atrophy |  |
| F151z00 | READ | 57632 | Spinal muscular atrophy NOS |  |
| F152.00 | READ | 4796 | Motor neurone disease |  |
| F152000 | READ | 36433 | Amyotrophic lateral sclerosis |  |
| F152100 | READ | 30987 | Progressive muscular atrophy |  |
| F152111 | READ | 7470 | Duchenne Aran muscular atrophy |  |
| F152200 | READ | 27377 | Progressive bulbar palsy |  |
| F152300 | READ | 18084 | Pseudobulbar palsy |  |
| F152400 | READ | 20845 | Primary lateral sclerosis |  |
| F152z00 | READ | 20120 | Motor neurone disease NOS |  |
| F15y.00 | READ | 71400 | Other anterior horn cell disease |  |
| F15z.00 | READ | 58729 | Anterior horn cell disease NOS |  |
| F16..00 | READ | 17194 | Other diseases of spinal cord |  |
| F160.00 | READ | 69740 | Syringomyelia and syringobulbia |  |
| F160000 | READ | 5195 | Syringomyelia |  |
| F160100 | READ | 47358 | Syringobulbia |  |
| F160z00 | READ | 96785 | Syringomyelia or syringobulbia NOS |  |
| F161.00 | READ | 56342 | Vascular myelopathies |  |
| F161000 | READ | 17216 | Myelopathy due to acute infarction of spinal cord |  |
| F161100 | READ | 65630 | Myelopathy due to arterial thrombosis of spinal cord |  |
| F161200 | READ | 67980 | Myelopathy due to oedema of spinal cord |  |
| F161300 | READ | 72449 | Myelopathy due to haematomyelia |  |
| F161400 | READ | 45714 | Subacute necrotic myelopathy |  |
| F161500 | READ | 33535 | Anterior spinal artery thrombosis |  |
| F161z00 | READ | 60189 | Vascular myelopathy NOS |  |
| F162.00 | READ | 7736 | Subacute combined degeneration of spinal cord |  |
| F163.00 | READ | 41250 | Myelopathy due to disease EC |  |
| F163000 | READ | 99739 | Myelopathy due to intervertebral disc disease |  |
| F163100 | READ | 67422 | Myelopathy due to neoplastic disease |  |
| F163200 | READ | 8920 | Myelopathy due to spondylosis |  |
| F163z00 | READ | 24346 | Myelopathy due to disease NOS |  |
| F16y.00 | READ | 68598 | Other myelopathy |  |
| F16y000 | READ | 55767 | Drug induced myelopathy |  |
| F16y100 | READ | 62758 | Radiation induced myelopathy |  |
| F16yz00 | READ | 70733 | Other myelopathy NOS |  |
| F16z.00 | READ | 4844 | Myelopathy NOS |  |
| F16z.11 | READ | 8816 | Cord compression NOS |  |
| F16z.12 | READ | 16564 | Spinal cord compression NOS |  |
| F17..00 | READ | 22849 | Autonomic nervous system disorders |  |
| F170.00 | READ | 16368 | Idiopathic peripheral autonomic neuropathy |  |
| F170000 | READ | 16471 | Carotid sinus syndrome |  |
| F170100 | READ | 62733 | Cervical sympathetic paralysis |  |
| F170z00 | READ | 49664 | Idiopathic peripheral autonomic neuropathy NOS |  |
| F171.00 | READ | 33257 | Peripheral autonomic neuropathy disease EC |  |
| F171000 | READ | 30668 | Autonomic neuropathy due to amyloid |  |
| F171100 | READ | 17067 | Autonomic neuropathy due to diabetes |  |
| F171z00 | READ | 50099 | Peripheral autonomic neuropathy due to disease NOS |  |
| F172.00 | READ | 5600 | [X] Horners syndrome |  |
| F173.00 | READ | 10733 | Shoulder-hand syndrome |  |
| F174.00 | READ | 22454 | Multiple system atrophy |  |
| F17z.00 | READ | 2748 | Autonomic nervous system disorder NOS |  |
| F17z.11 | READ | 16167 | Horner's syndrome |  |
| F17z.12 | READ | 40383 | Autonomic failure |  |
| F1y..00 | READ | 61968 | Hereditary and degenerative diseases of the CNS OS |  |
| F1z..00 | READ | 56006 | Hereditary and degenerative diseases of the CNS NOS |  |
| F20..00 | READ | 684 | Multiple sclerosis |  |
| F20..11 | READ | 3440 | Disseminated sclerosis |  |
| F200.00 | READ | 40344 | Multiple sclerosis of the brain stem |  |
| F201.00 | READ | 69886 | Multiple sclerosis of the spinal cord |  |
| F202.00 | READ | 23730 | Generalised multiple sclerosis |  |
| F203.00 | READ | 2298 | Exacerbation of multiple sclerosis |  |
| F20z.00 | READ | 20493 | Multiple sclerosis NOS |  |
| F21..00 | READ | 3464 | Other central nervous system demyelinating diseases |  |
| F210.00 | READ | 44795 | Neuromyelitis optica |  |
| F210.11 | READ | 36312 | Devic's disease |  |
| F211.11 | READ | 62945 | Balo's concentric sclerosis |  |
| F212.00 | READ | 66907 | Acute and subacute haemorrhagic leukoencephalitis [Hurst] |  |
| F21X.00 | READ | 28082 | Acute disseminated demyelination, unspecified |  |
| F21y.00 | READ | 43583 | Other specified central nervous system demyelinating disease |  |
| F21y000 | READ | 56153 | Marchiafava-Bignami disease |  |
| F21y100 | READ | 40501 | Central pontine myelinosis |  |
| F21y200 | READ | 5095 | Binswanger's disease |  |
| F21y211 | READ | 68194 | Binswanger's encephalopathy |  |
| F21y300 | READ | 55426 | Central demyelination of corpus callosum |  |
| F21y400 | READ | 69848 | Subacute necrotizing myelitis |  |
| F21y500 | READ | 40968 | Concentric sclerosis |  |
| F21yz00 | READ | 54300 | Other specified central nervous system demyelination NOS |  |
| F21z.00 | READ | 12054 | Central nervous system demyelination NOS |  |
| F22..00 | READ | 1749 | Hemiplegia |  |
| F22..11 | READ | 807 | Hemiparesis |  |
| F220.00 | READ | 39085 | Flaccid hemiplegia |  |
| F221.00 | READ | 20122 | Spastic hemiplegia |  |
| F221.11 | READ | 35106 | Spastic foot |  |
| F222.00 | READ | 8933 | Left hemiplegia |  |
| F222.11 | READ | 8862 | Left sided weakness |  |
| F223.00 | READ | 3293 | Right hemiplegia |  |
| F223.11 | READ | 2713 | Right sided weakness |  |
| F22z.00 | READ | 8492 | Hemiplegia NOS |  |
| F23..00 | READ | 2069 | Congenital cerebral palsy |  |
| F23..11 | READ | 15530 | Congenital spastic cerebral palsy |  |
| F23..12 | READ | 5560 | Infantile cerebral palsy |  |
| F23..13 | READ | 39971 | Littles disease |  |
| F23..14 | READ | 61219 | Cerebral atonia |  |
| F230.00 | READ | 25324 | Congenital diplegia |  |
| F230.11 | READ | 99040 | Paraplegia - congenital |  |
| F230000 | READ | 37160 | Congenital paraplegia |  |
| F230100 | READ | 5512 | Cerebral palsy with spastic diplegia |  |
| F230z00 | READ | 45551 | Congenital diplegia NOS |  |
| F231.00 | READ | 27966 | Congenital hemiplegia |  |
| F232.00 | READ | 21249 | Congenital quadriplegia |  |
| F232.11 | READ | 48126 | Tetraplegia - congenital |  |
| F233.00 | READ | 33925 | Congenital monoplegia |  |
| F233.11 | READ | 55593 | Congenital spastic foot |  |
| F234.00 | READ | 2019 | Infantile hemiplegia NOS |  |
| F23y.00 | READ | 53178 | Other congenital cerebral palsy |  |
| F23y000 | READ | 21548 | Ataxic infantile cerebral palsy |  |
| F23y200 | READ | 25570 | Spastic cerebral palsy |  |
| F23y300 | READ | 49967 | Dyskinetic cerebral palsy |  |
| F23y400 | READ | 52659 | Ataxic diplegic cerebral palsy |  |
| F23y500 | READ | 95132 | Worster-Drought syndrome |  |
| F23yz00 | READ | 12666 | Other infantile cerebral palsy NOS |  |
| F23z.00 | READ | 28306 | Congenital cerebral palsy NOS |  |
| F24..00 | READ | 39630 | Other paralytic syndromes |  |
| F240.00 | READ | 9271 | Quadriplegia |  |
| F240.11 | READ | 16117 | Tetraplegia |  |
| F240000 | READ | 46128 | Flaccid tetraplegia |  |
| F240100 | READ | 35540 | Spastic tetraplegia |  |
| F241.00 | READ | 3063 | Paraplegia |  |
| F241000 | READ | 46175 | Flaccid paraplegia |  |
| F241100 | READ | 9375 | Spastic paraplegia |  |
| F242.00 | READ | 22907 | Diplegia of upper limbs |  |
| F24y.00 | READ | 2848 | Other specified paralytic syndromes |  |
| F24y000 | READ | 9385 | Progressive supranuclear palsy |  |
| F24y011 | READ | 49034 | Steele Richardson Olszewsk syn |  |
| F24y012 | READ | 93910 | Steele - Richardson Oszewski syndrome |  |
| F24y100 | READ | 18688 | Todd's paralysis |  |
| F24y200 | READ | 7037 | Steele-Richardson-Olszewski syndrome |  |
| F24yz00 | READ | 39082 | Other paralytic syndromes NOS |  |
| F24yz11 | READ | 7167 | Specified palsy NEC |  |
| F24z.00 | READ | 2640 | Paralysis NOS |  |
| F2A..00 | READ | 12793 | Hemiparesis |  |
| F2Az.00 | READ | 65275 | Hemiparesis NOS |  |
| Fyu9.00 | READ | 53755 | [X]Cerebral palsy and other paralytic syndromes |  |
| Fyu9000 | READ | 90520 | [X]Other infantile cerebral palsy |  |
| Fyu9100 | READ | 98465 | [X]Other specified paralytic syndromes |  |

| risk_factor definition 8: COPD (not asthma) |
| --- |
| n= 185 medcodes used |

| **Read_code** | **readoxmisflag** | **medcode** | **desc** |  |
| --- | --- | --- | --- | --- |
| H3...00 | READ | 1001 | Chronic obstructive pulmonary disease |  |
| H3...11 | READ | 998 | Chronic obstructive airways disease |  |
| H31..00 | READ | 3243 | Chronic bronchitis |  |
| H310.00 | READ | 25603 | Simple chronic bronchitis |  |
| H310000 | READ | 15626 | Chronic catarrhal bronchitis |  |
| H310100 | READ | 16717 | Smokers' cough |  |
| H310z00 | READ | 61118 | Simple chronic bronchitis NOS |  |
| H311.00 | READ | 11150 | Mucopurulent chronic bronchitis |  |
| H311000 | READ | 40159 | Purulent chronic bronchitis |  |
| H311100 | READ | 37959 | Fetid chronic bronchitis |  |
| H311z00 | READ | 61513 | Mucopurulent chronic bronchitis NOS |  |
| H312.00 | READ | 27819 | Obstructive chronic bronchitis |  |
| H312000 | READ | 5798 | Chronic asthmatic bronchitis |  |
| H312011 | READ | 5909 | Chronic wheezy bronchitis |  |
| H312100 | READ | 14798 | Emphysematous bronchitis |  |
| H312200 | READ | 1446 | Acute exacerbation of chronic obstructive airways disease |  |
| H312300 | READ | 26125 | Bronchiolitis obliterans |  |
| H312z00 | READ | 44525 | Obstructive chronic bronchitis NOS |  |
| H313.00 | READ | 24248 | Mixed simple and mucopurulent chronic bronchitis |  |
| H31y.00 | READ | 66043 | Other chronic bronchitis |  |
| H31y000 | READ | 23618 | Chronic tracheitis |  |
| H31y100 | READ | 45089 | Chronic tracheobronchitis |  |
| H31yz00 | READ | 68066 | Other chronic bronchitis NOS |  |
| H31z.00 | READ | 15157 | Chronic bronchitis NOS |  |
| H32..00 | READ | 794 | Emphysema |  |
| H320.00 | READ | 26306 | Chronic bullous emphysema |  |
| H320000 | READ | 56860 | Segmental bullous emphysema |  |
| H320100 | READ | 68662 | Zonal bullous emphysema |  |
| H320200 | READ | 60188 | Giant bullous emphysema |  |
| H320300 | READ | 99536 | Bullous emphysema with collapse |  |
| H320z00 | READ | 23492 | Chronic bullous emphysema NOS |  |
| H321.00 | READ | 46578 | Panlobular emphysema |  |
| H322.00 | READ | 10980 | Centrilobular emphysema |  |
| H32y.00 | READ | 40788 | Other emphysema |  |
| H32y000 | READ | 92955 | Acute vesicular emphysema |  |
| H32y100 | READ | 70787 | Atrophic (senile) emphysema |  |
| H32y111 | READ | 59263 | Acute interstitial emphysema |  |
| H32y200 | READ | 63479 | MacLeod's unilateral emphysema |  |
| H32yz00 | READ | 16410 | Other emphysema NOS |  |
| H32z.00 | READ | 33450 | Emphysema NOS |  |
| H34..00 | READ | 2195 | Bronchiectasis |  |
| H340.00 | READ | 20364 | Recurrent bronchiectasis |  |
| H341.00 | READ | 41491 | Post-infective bronchiectasis |  |
| H34z.00 | READ | 32679 | Bronchiectasis NOS |  |
| H35..00 | READ | 11312 | Extrinsic allergic alveolitis |  |
| H350.00 | READ | 15588 | Farmers' lung |  |
| H351.00 | READ | 62200 | Bagassosis |  |
| H352.00 | READ | 27345 | Bird-fancier's lung |  |
| H352000 | READ | 54822 | Budgerigar-fanciers' lung |  |
| H352100 | READ | 31447 | Pigeon-fanciers' lung |  |
| H352z00 | READ | 36240 | Bird-fancier's lung NOS |  |
| H353.00 | READ | 93206 | Suberosis ( cork-handlers' lung ) |  |
| H354.00 | READ | 67709 | Malt workers' lung |  |
| H355.00 | READ | 41694 | Mushroom workers' lung |  |
| H356.00 | READ | 56652 | Maple bark strippers' lung |  |
| H357.00 | READ | 26278 | pneumonitis |  |
| H35y.00 | READ | 51858 | Other allergic alveolitis |  |
| H35y000 | READ | 91989 | Cheese-washers' lung |  |
| H35y100 | READ | 53943 | Coffee-workers' lung |  |
| H35y200 | READ | 70286 | Fish-meal workers' lung |  |
| H35y500 | READ | 45427 | Pituitary snuff-takers' disease |  |
| H35y600 | READ | 93353 | Sequoiosis (red-cedar asthma) |  |
| H35y700 | READ | 39478 | Wood asthma |  |
| H35y800 | READ | 69452 | Air-conditioner and humidifier lung |  |
| H35yz00 | READ | 55552 | Other allergic alveolitis NOS |  |
| H35z.00 | READ | 46977 | Allergic alveolitis and pneumonitis NOS |  |
| H35z000 | READ | 62442 | Allergic extrinsic alveolitis NOS |  |
| H35z100 | READ | 11833 | Hypersensitivity pneumonitis NOS |  |
| H35zz00 | READ | 53095 | Allergic alveolitis and pneumonitis NOS |  |
| H36..00 | READ | 10863 | Mild chronic obstructive pulmonary disease |  |
| H37..00 | READ | 10802 | Moderate chronic obstructive pulmonary disease |  |
| H38..00 | READ | 9876 | Severe chronic obstructive pulmonary disease |  |
| H39..00 | READ | 93568 | Very severe chronic obstructive pulmonary disease |  |
| H3y..00 | READ | 12166 | Other specified chronic obstructive airways disease |  |
| H3y..11 | READ | 67040 | Other specified chronic obstructive pulmonary disease |  |
| H3y0.00 | READ | 21061 | Chronic obstruct pulmonary dis with acute lower resp infectn |  |
| H3y1.00 | READ | 7884 | Chron obstruct pulmonary dis wth acute exacerbation, unspec |  |
| H3z..00 | READ | 5710 | Chronic obstructive airways disease NOS |  |
| H3z..11 | READ | 37247 | Chronic obstructive pulmonary disease NOS |  |
| H4...00 | READ | 21973 | Lung disease due to external agents |  |
| H4...11 | READ | 25013 | Pneumoconioses |  |
| H4...12 | READ | 21257 | Occupational lung disease |  |
| H40..00 | READ | 19492 | Coal workers' pneumoconiosis |  |
| H41..00 | READ | 8303 | Asbestosis |  |
| H410.00 | READ | 5005 | Pleural plaque disease due to asbestosis |  |
| H41z.00 | READ | 51410 | Asbestosis NOS |  |
| H42..00 | READ | 46460 | Silica and silicate pneumoconiosis |  |
| H420.00 | READ | 60805 | Talc pneumoconiosis |  |
| H421.00 | READ | 62233 | Simple silicosis |  |
| H422.00 | READ | 71853 | Complicated silicosis |  |
| H423.00 | READ | 89206 | Massive silicotic fibrosis |  |
| H42z.00 | READ | 23446 | Silica pneumoconiosis NOS |  |
| H43..00 | READ | 65376 | Pneumoconiosis due to other inorganic dust |  |
| H431.00 | READ | 94894 | Bauxite fibrosis of lung |  |
| H432.00 | READ | 49194 | Berylliosis |  |
| H433.00 | READ | 94575 | Graphite fibrosis of lung |  |
| H434.00 | READ | 30235 | Siderosis |  |
| H435.00 | READ | 93577 | Stannosis |  |
| H43z.00 | READ | 23461 | Pneumoconiosis due to inorganic dust NOS |  |
| H44..00 | READ | 60313 | Pneumopathy due to inhalation of other dust |  |
| H440.00 | READ | 37365 | Byssinosis |  |
| H441.00 | READ | 26442 | Cannabinosis |  |
| H44z.00 | READ | 73414 | Pneumopathy due to inhalation of other dust NOS |  |
| H45..00 | READ | 31423 | Pneumoconiosis NOS |  |
| H450.00 | READ | 63172 | Pneumoconiosis associated with tuberculosis |  |
| H46..00 | READ | 31722 | Respiratory disease due to chemical fumes and vapours |  |
| H460.00 | READ | 38639 | Bronchitis and pneumonitis due to chemical fumes |  |
| H460000 | READ | 54830 | Acute bronchitis due to chemical fumes |  |
| H460100 | READ | 49025 | Acute pneumonitis due to chemical fumes |  |
| H460z00 | READ | 55758 | Bronchitis and pneumonitis due to chemical fumes NOS |  |
| H461.00 | READ | 62227 | Acute pulmonary oedema due to chemical fumes |  |
| H462.00 | READ | 20448 | Upper respiratory inflammation due to chemical fumes |  |
| H463.00 | READ | 52937 | Other acute respiratory diseases due to chemical fumes |  |
| H464.00 | READ | 47142 | Chronic respiratory conditions due to chemical fumes |  |
| H464000 | READ | 64721 | Chronic emphysema due to chemical fumes |  |
| H464100 | READ | 63216 | Obliterative bronchiolitis due to chemical fumes |  |
| H464200 | READ | 47782 | Chronic pulmonary fibrosis due to chemical fumes |  |
| H464z00 | READ | 70815 | Chronic respiratory conditions due to chemical fumes NOS |  |
| H46z.00 | READ | 48647 | Respiratory conditions due to chemical fumes NOS |  |
| H46zz00 | READ | 33663 | Respiratory conditions due to chemical fumes NOS |  |
| H47..00 | READ | 9711 | Pneumonitis due to inhalation of solids or liquids |  |
| H47..11 | READ | 10992 | Aspiration pneumonitis |  |
| H47y.00 | READ | 47504 | Pneumonitis due to inhalation of other solid or liquid |  |
| H47y000 | READ | 47684 | Detergent asthma |  |
| H47yz00 | READ | 54252 | Pneumonitis due to inhalation of solid or liquid NOS |  |
| H47z.00 | READ | 46066 | Pneumonitis due to inhalation of solid or liquid NOS |  |
| H48..00 | READ | 43285 | Progressive massive fibrosis |  |
| H4y..00 | READ | 38985 | Other specified lung diseases due to external agent |  |
| H4y0.00 | READ | 51713 | Acute pulmonary radiation disease |  |
| H4y0000 | READ | 18130 | Acute radiation pneumonitis |  |
| H4y1.00 | READ | 69914 | Chronic pulmonary radiation disease |  |
| H4y1000 | READ | 22536 | Chronic pulmonary fibrosis following radiation |  |
| H4y1z00 | READ | 50374 | Chronic pulmonary radiation disease NOS |  |
| H4y2.00 | READ | 44015 | Drug-induced interstitial lung disorders |  |
| H4y2000 | READ | 53205 | Acute drug-induced interstitial lung disorders |  |
| H4y2100 | READ | 43417 | Chronic drug-induced interstitial lung disorders |  |
| H4yy.00 | READ | 55557 | Other external agent causing respiratory condition |  |
| H4yz.00 | READ | 62707 | External agent causing respiratory conditions NOS |  |
| H4z..00 | READ | 34001 | Lung disease due to external agents NOS |  |
| H541000 | READ | 26082 | Chronic pulmonary oedema |  |
| H55..00 | READ | 7791 | Postinflammatory pulmonary fibrosis |  |
| H55..11 | READ | 40953 | Cirrhosis of lung |  |
| H562.00 | READ | 68814 | Pulmonary alveolar microlithiasis |  |
| H563.00 | READ | 6837 | Idiopathic fibrosing alveolitis |  |
| H563.11 | READ | 63174 | Hamman - Rich syndrome |  |
| H563.12 | READ | 5519 | Cryptogenic fibrosing alveolitis |  |
| H563000 | READ | 94136 | Alveolar capillary block |  |
| H563100 | READ | 6051 | Diffuse pulmonary fibrosis |  |
| H563z00 | READ | 28229 | Idiopathic fibrosing alveolitis NOS |  |
| H57..00 | READ | 49044 | Lung involvement in diseases EC |  |
| H570.00 | READ | 9954 | Rheumatoid lung |  |
| H571.00 | READ | 64799 | Rheumatic pneumonia |  |
| H572.00 | READ | 94996 | Lung disease with systemic sclerosis |  |
| H57y.00 | READ | 58791 | Lung disease with diseases EC |  |
| H57y000 | READ | 54010 | Pulmonary amyloidosis |  |
| H57y100 | READ | 42940 | Lung disease with polymyositis |  |
| H57y200 | READ | 3859 | Pulmonary sarcoidosis |  |
| H57y300 | READ | 47364 | Lung disease with Sjogren's disease |  |
| H57y400 | READ | 31564 | Lung disease with systemic lupus erythematosus |  |
| H57y500 | READ | 96655 | Lung disease with syphilis |  |
| H57yz00 | READ | 63912 | Lung disease with diseases EC NOS |  |
| H581.00 | READ | 22905 | Interstitial emphysema |  |
| H581.11 | READ | 35432 | Pneumomediastinum |  |
| H582.00 | READ | 54893 | Compensatory emphysema |  |
| H583.00 | READ | 22915 | Pulmonary eosinophilia |  |
| H583000 | READ | 31319 | Loeffler's syndrome |  |
| H583100 | READ | 16439 | Tropical eosinophilia |  |
| H583z00 | READ | 20269 | Pulmonary eosinophilia NOS |  |
| H59..00 | READ | 25249 | Respiratory failure |  |
| H591.00 | READ | 24814 | Chronic respiratory failure |  |
| H592.00 | READ | 94946 | Chronic type 1 respiratory failure |  |
| H593.00 | READ | 94486 | Chronic type 2 respiratory failure |  |
| Hyu3.00 | READ | 67278 | [X]Chronic lower respiratory diseases |  |
| Hyu3000 | READ | 66058 | [X]Other emphysema |  |
| Hyu3100 | READ | 65733 | [X]Other specified chronic obstructive pulmonary disease |  |
| Hyu5.00 | READ | 54706 | [X]Other resp diseases principally affecting interstitium |  |
| Hyu5000 | READ | 65060 | [X]Other interstitial pulmonary diseases with fibrosis |  |
| Hyu5100 | READ | 91912 | [X]Other specified interstitial pulmonary diseases |  |
| Hyu5200 | READ | 71906 | [X]Hepatopulmonary syndrome |  |
| Q317.00 | READ | 40715 | Perinatal chronic respiratory disease |  |
| Q317000 | READ | 6883 | Perinatal bronchopulmonary dysplasia |  |
| Q317100 | READ | 46795 | Prematurity with interstitial pulmonary fibrosis |  |
| Q317200 | READ | 48476 | Wilson-Mikity syndrome |  |
| Q317y00 | READ | 93710 | Other specified perinatal chronic respiratory disease |  |
| Q317z00 | READ | 59696 | Perinatal chronic respiratory disease NOS |  |

| risk_factor definition 9: Immunosuppressive conditions |
| --- |
| n= 263 medcodes used |

| **Read_code** | **readoxmisflag** | **medcode** | **desc** |  |
| --- | --- | --- | --- | --- |
| 14N7.00 | READ | 16827 | H/O: splenectomy |  |
| 2J3..00 | READ | 40484 | General immune status |  |
| 2J30.00 | READ | 47106 | Patient immunocompromised |  |
| 2J31.00 | READ | 22307 | Patient immunosuppressed |  |
| 43C3.00 | READ | 540 | HTLV-3 antibody positive |  |
| 43C3.11 | READ | 2835 | HIV positive |  |
| 7840.00 | READ | 34310 | Total excision of spleen |  |
| 7840.11 | READ | 2759 | Total splenectomy |  |
| 7840100 | READ | 27514 | Total splenectomy |  |
| 7840200 | READ | 65148 | Excision of accessory spleen |  |
| 7840300 | READ | 1393 | Splenectomy NEC |  |
| 7840400 | READ | 31041 | Laparoscopic total splenectomy |  |
| 7840z00 | READ | 59784 | Total excision of spleen NOS |  |
| A788.00 | READ | 23770 | Acquired immune deficiency syndrome |  |
| A788.11 | READ | 9130 | Human immunodeficiency virus infection |  |
| A788000 | READ | 58857 | Acute human immunodeficiency virus infection |  |
| A788100 | READ | 58859 | Asymptomatic human immunodeficiency virus infection |  |
| A788200 | READ | 69766 | HIV infection with persistent generalised lymphadenopathy |  |
| A788300 | READ | 70869 | Human immunodeficiency virus with constitutional disease |  |
| A788400 | READ | 53636 | Human immunodeficiency virus with neurological disease |  |
| A788500 | READ | 70528 | Human immunodeficiency virus with secondary infection |  |
| A788600 | READ | 101836 | Human immunodeficiency virus with secondary cancers |  |
| A788U00 | READ | 47632 | HIV disease result/haematological+immunologic abnorms,NEC |  |
| A788W00 | READ | 67575 | HIV disease resulting in unspecified malignant neoplasm |  |
| A788X00 | READ | 71450 | HIV disease resulting/unspcf infectious+parasitic disease |  |
| A788y00 | READ | 62891 | Human immunodeficiency virus with other clinical findings |  |
| A788z00 | READ | 36294 | Acquired human immunodeficiency virus infection syndrome NOS |  |
| A789.00 | READ | 44303 | Human immunodef virus resulting in other disease |  |
| A789000 | READ | 37006 | HIV disease resulting in mycobacterial infection |  |
| A789100 | READ | 66368 | HIV disease resulting in cytomegaloviral disease |  |
| A789200 | READ | 23951 | HIV disease resulting in candidiasis |  |
| A789300 | READ | 27641 | HIV disease resulting in Pneumocystis carinii pneumonia |  |
| A789400 | READ | 50076 | HIV disease resulting in multiple infections |  |
| A789500 | READ | 27853 | HIV disease resulting in Kaposi's sarcoma |  |
| A789600 | READ | 44617 | HIV disease resulting in Burkitt's lymphoma |  |
| A789700 | READ | 66367 | HIV dis resulting oth types of non-Hodgkin's lymphoma |  |
| A789900 | READ | 65117 | HIV disease resulting in lymphoid interstitial pneumonitis |  |
| A789A00 | READ | 8281 | HIV disease resulting in wasting syndrome |  |
| A789X00 | READ | 51708 | HIV dis reslt/oth mal neopl/lymph,h'matopoetc+reltd tissu |  |
| B6...00 | READ | 12323 | Malignant neoplasm of lymphatic and haemopoietic tissue |  |
| B6...11 | READ | 37112 | Malignant neoplasm of histiocytic tissue |  |
| B60..00 | READ | 41369 | Lymphosarcoma and reticulosarcoma |  |
| B600.00 | READ | 1481 | Reticulosarcoma |  |
| B600000 | READ | 60242 | Reticulosarcoma of unspecified site |  |
| B600100 | READ | 71031 | Reticulosarcoma of lymph nodes of head, face and neck |  |
| B600300 | READ | 70374 | Reticulosarcoma of intra-abdominal lymph nodes |  |
| B600700 | READ | 95058 | Reticulosarcoma of spleen |  |
| B600z00 | READ | 99240 | Reticulosarcoma NOS |  |
| B601.00 | READ | 27416 | Lymphosarcoma |  |
| B601000 | READ | 71625 | Lymphosarcoma of unspecified site |  |
| B601100 | READ | 71238 | Lymphosarcoma of lymph nodes of head, face and neck |  |
| B601200 | READ | 62380 | Lymphosarcoma of intrathoracic lymph nodes |  |
| B601300 | READ | 64670 | Lymphosarcoma of intra-abdominal lymph nodes |  |
| B601500 | READ | 100352 | Lymphosarcoma of lymph nodes of inguinal region and leg |  |
| B601z00 | READ | 63723 | Lymphosarcoma NOS |  |
| B602.00 | READ | 21402 | Burkitt's lymphoma |  |
| B602100 | READ | 59115 | Burkitt's lymphoma of lymph nodes of head, face and neck |  |
| B602200 | READ | 100006 | Burkitt's lymphoma of intrathoracic lymph nodes |  |
| B602300 | READ | 97577 | Burkitt's lymphoma of intra-abdominal lymph nodes |  |
| B602500 | READ | 92380 | Burkitt's lymphoma of lymph nodes of inguinal region and leg |  |
| B602z00 | READ | 71304 | Burkitt's lymphoma NOS |  |
| B60y.00 | READ | 99887 | Other specified reticulosarcoma or lymphosarcoma |  |
| B60z.00 | READ | 99951 | Reticulosarcoma or lymphosarcoma NOS |  |
| B61..00 | READ | 2462 | Hodgkin's disease |  |
| B610.00 | READ | 65489 | Hodgkin's paragranuloma |  |
| B610100 | READ | 100423 | Hodgkin's paragranuloma of lymph nodes of head, face, neck |  |
| B610300 | READ | 98840 | Hodgkin's paragranuloma of intra-abdominal lymph nodes |  |
| B611.00 | READ | 44196 | Hodgkin's granuloma |  |
| B611100 | READ | 98909 | Hodgkin's granuloma of lymph nodes of head, face and neck |  |
| B612.00 | READ | 64036 | Hodgkin's sarcoma |  |
| B612400 | READ | 68039 | Hodgkin's sarcoma of lymph nodes of axilla and upper limb |  |
| B613.00 | READ | 38939 | Hodgkin's disease, lymphocytic-histiocytic predominance |  |
| B613000 | READ | 71142 | Hodgkin's, lymphocytic-histiocytic predominance unspec site |  |
| B613100 | READ | 68330 | Hodgkin's, lymphocytic-histiocytic pred of head, face, neck |  |
| B613200 | READ | 92245 | Hodgkin's, lymphocytic-histiocytic pred intrathoracic nodes |  |
| B613300 | READ | 73532 | Hodgkin's, lymphocytic-histiocytic pred intra-abdominal node |  |
| B613500 | READ | 93951 | Hodgkin's, lymphocytic-histiocytic pred inguinal and leg |  |
| B613600 | READ | 95338 | Hodgkin's, lymphocytic-histiocytic pred intrapelvic nodes |  |
| B613z00 | READ | 29876 | Hodgkin's, lymphocytic-histiocytic predominance NOS |  |
| B614.00 | READ | 29178 | Hodgkin's disease, nodular sclerosis |  |
| B614000 | READ | 57225 | Hodgkin's disease, nodular sclerosis of unspecified site |  |
| B614100 | READ | 55303 | Hodgkin's nodular sclerosis of head, face and neck |  |
| B614200 | READ | 67506 | Hodgkin's nodular sclerosis of intrathoracic lymph nodes |  |
| B614300 | READ | 61149 | Hodgkin's nodular sclerosis of intra-abdominal lymph nodes |  |
| B614400 | READ | 65483 | Hodgkin's nodular sclerosis of lymph nodes of axilla and arm |  |
| B614800 | READ | 19140 | Hodgkin's nodular sclerosis of lymph nodes of multiple sites |  |
| B614z00 | READ | 63054 | Hodgkin's disease, nodular sclerosis NOS |  |
| B615.00 | READ | 49605 | Hodgkin's disease, mixed cellularity |  |
| B615000 | READ | 97863 | Hodgkin's disease, mixed cellularity of unspecified site |  |
| B615100 | READ | 94407 | Hodgkin's mixed cellularity of lymph nodes head, face, neck |  |
| B615200 | READ | 58684 | Hodgkin's mixed cellularity of intrathoracic lymph nodes |  |
| B615z00 | READ | 94005 | Hodgkin's disease, mixed cellularity NOS |  |
| B616.00 | READ | 67703 | Hodgkin's disease, lymphocytic depletion |  |
| B616000 | READ | 95049 | Hodgkin's lymphocytic depletion of unspecified site |  |
| B616400 | READ | 63625 | Hodgkin's lymphocytic depletion lymph nodes axilla and arm |  |
| B616700 | READ | 101715 | Hodgkin's disease, lymphocytic depletion of spleen |  |
| B616z00 | READ | 101530 | Hodgkin's disease, lymphocytic depletion NOS |  |
| B61z.00 | READ | 53397 | Hodgkin's disease NOS |  |
| B61z000 | READ | 61662 | Hodgkin's disease NOS, unspecified site |  |
| B61z100 | READ | 59778 | Hodgkin's disease NOS of lymph nodes of head, face and neck |  |
| B61z200 | READ | 59755 | Hodgkin's disease NOS of intrathoracic lymph nodes |  |
| B61z400 | READ | 91900 | Hodgkin's disease NOS of lymph nodes of axilla and arm |  |
| B61z500 | READ | 99012 | Hodgkin's disease NOS of lymph nodes inguinal region and leg |  |
| B61z700 | READ | 94279 | Hodgkin's disease NOS of spleen |  |
| B61z800 | READ | 97746 | Hodgkin's disease NOS of lymph nodes of multiple sites |  |
| B61zz00 | READ | 42461 | Hodgkin's disease NOS |  |
| B62..00 | READ | 33333 | Other malignant neoplasm of lymphoid and histiocytic tissue |  |
| B620.00 | READ | 5179 | Nodular lymphoma (Brill - Symmers disease) |  |
| B620000 | READ | 66327 | Nodular lymphoma of unspecified site |  |
| B620100 | READ | 45264 | Nodular lymphoma of lymph nodes of head, face and neck |  |
| B620300 | READ | 92068 | Nodular lymphoma of intra-abdominal lymph nodes |  |
| B620500 | READ | 94995 | Nodular lymphoma of lymph nodes of inguinal region and leg |  |
| B620800 | READ | 58082 | Nodular lymphoma of lymph nodes of multiple sites |  |
| B620z00 | READ | 65701 | Nodular lymphoma NOS |  |
| B621.00 | READ | 12006 | Mycosis fungoides |  |
| B621000 | READ | 95949 | Mycosis fungoides of unspecified site |  |
| B621300 | READ | 91674 | Mycosis fungoides of intra-abdominal lymph nodes |  |
| B621400 | READ | 96379 | Mycosis fungoides of lymph nodes of axilla and upper limb |  |
| B621500 | READ | 72714 | Mycosis fungoides of lymph nodes of inguinal region and leg |  |
| B621800 | READ | 95012 | Mycosis fungoides of lymph nodes of multiple sites |  |
| B621z00 | READ | 38005 | Mycosis fungoides NOS |  |
| B622.00 | READ | 35014 | Sezary's disease |  |
| B622z00 | READ | 100532 | Sezary's disease NOS |  |
| B623.00 | READ | 44267 | Malignant histiocytosis |  |
| B623000 | READ | 69497 | Malignant histiocytosis of unspecified site |  |
| B623100 | READ | 94415 | Malignant histiocytosis of lymph nodes head, face and neck |  |
| B623300 | READ | 65642 | Malignant histiocytosis of intra-abdominal lymph nodes |  |
| B623z00 | READ | 58871 | Malignant histiocytosis NOS |  |
| B624.00 | READ | 27330 | Leukaemic reticuloendotheliosis |  |
| B624.11 | READ | 5137 | Leukaemic reticuloendotheliosis |  |
| B624.12 | READ | 87335 | Hairy cell leukaemia |  |
| B624000 | READ | 65122 | Leukaemic reticuloendotheliosis of unspecified sites |  |
| B624300 | READ | 65123 | Leukaemic reticuloend of intra-abdominal lymph nodes |  |
| B624z00 | READ | 73777 | Leukaemic reticuloendotheliosis NOS |  |
| B625.00 | READ | 34926 | Letterer-Siwe disease |  |
| B625.11 | READ | 4870 | Histiocytosis X (acute, progressive) |  |
| B625200 | READ | 102158 | Letterer-Siwe disease of intrathoracic lymph nodes |  |
| B625800 | READ | 54083 | Letterer-Siwe disease of lymph nodes of multiple sites |  |
| B625z00 | READ | 47204 | Letterer-Siwe disease NOS |  |
| B626.00 | READ | 15036 | Malignant mast cell tumours |  |
| B626500 | READ | 100615 | Mast cell malignancy of lymph nodes inguinal region and leg |  |
| B626800 | READ | 31324 | Mast cell malignancy of lymph nodes of multiple sites |  |
| B626z00 | READ | 89657 | Malignant mast cell tumour NOS |  |
| B627.00 | READ | 3604 | Non - Hodgkin's lymphoma |  |
| B627000 | READ | 28639 | Follicular non-Hodgkin's small cleaved cell lymphoma |  |
| B627100 | READ | 70842 | Follicular non-Hodg mixed sml cleavd & lge cell lymphoma |  |
| B627200 | READ | 49262 | Follicular non-Hodgkin's large cell lymphoma |  |
| B627300 | READ | 50668 | Diffuse non-Hodgkin's small cell (diffuse) lymphoma |  |
| B627500 | READ | 50695 | Diffuse non-Hodgkin mixed sml & lge cell (diffuse) lymphoma |  |
| B627600 | READ | 53551 | Diffuse non-Hodgkin's immunoblastic (diffuse) lymphoma |  |
| B627700 | READ | 17460 | Diffuse non-Hodgkin's lymphoblastic (diffuse) lymphoma |  |
| B627800 | READ | 65180 | Diffuse non-Hodgkin's lymphoma undifferentiated (diffuse) |  |
| B627900 | READ | 95715 | Mucosa-associated lymphoma |  |
| B627911 | READ | 95545 | Maltoma |  |
| B627B00 | READ | 31576 | Other types of follicular non-Hodgkin's lymphoma |  |
| B627C00 | READ | 21549 | Follicular non-Hodgkin's lymphoma |  |
| B627C11 | READ | 17182 | Follicular lymphoma NOS |  |
| B627D00 | READ | 70509 | Diffuse non-Hodgkin's centroblastic lymphoma |  |
| B627W00 | READ | 31794 | Unspecified B-cell non-Hodgkin's lymphoma |  |
| B627X00 | READ | 39798 | Diffuse non-Hodgkin's lymphoma, unspecified |  |
| B62x.00 | READ | 17887 | Malignant lymphoma otherwise specified |  |
| B62x000 | READ | 90201 | T-zone lymphoma |  |
| B62x100 | READ | 57737 | Lymphoepithelioid lymphoma |  |
| B62x200 | READ | 12464 | Peripheral T-cell lymphoma |  |
| B62x400 | READ | 62437 | Malignant reticulosis |  |
| B62x500 | READ | 58962 | Malignant immunoproliferative small intestinal disease |  |
| B62x600 | READ | 95630 | True histiocytic lymphoma |  |
| B62xX00 | READ | 44318 | Oth and unspecif peripheral & cutaneous T-cell lymphomas |  |
| B62y.00 | READ | 12335 | Malignant lymphoma NOS |  |
| B62y000 | READ | 57427 | Malignant lymphoma NOS of unspecified site |  |
| B62y100 | READ | 50696 | Malignant lymphoma NOS of lymph nodes of head, face and neck |  |
| B62y200 | READ | 72725 | Malignant lymphoma NOS of intrathoracic lymph nodes |  |
| B62y300 | READ | 42579 | Malignant lymphoma NOS of intra-abdominal lymph nodes |  |
| B62y400 | READ | 34089 | Malignant lymphoma NOS of lymph nodes of axilla and arm |  |
| B62y500 | READ | 63105 | Malignant lymphoma NOS of lymph node inguinal region and leg |  |
| B62y600 | READ | 71262 | Malignant lymphoma NOS of intrapelvic lymph nodes |  |
| B62y700 | READ | 60092 | Malignant lymphoma NOS of spleen |  |
| B62y800 | READ | 15504 | Malignant lymphoma NOS of lymph nodes of multiple sites |  |
| B62yz00 | READ | 15027 | Malignant lymphoma NOS |  |
| B62z.00 | READ | 65434 | Malignant neoplasms of lymphoid and histiocytic tissue NOS |  |
| B62z100 | READ | 64427 | Unspec malig neop lymphoid/histiocytic lymph node head/neck |  |
| B62z200 | READ | 93384 | Unspec malig neop lymphoid/histiocytic of intrathoracic node |  |
| B62z500 | READ | 71609 | Unspec malig neop lymphoid/histiocytic nodes inguinal/leg |  |
| B62z800 | READ | 101465 | Unspec malig neop lymphoid/histiocytic of multiple sites |  |
| B62zz00 | READ | 95792 | Lymphoid and histiocytic malignancy NOS |  |
| B62zz11 | READ | 70716 | Immunoproliferative neoplasm |  |
| B63..00 | READ | 37182 | Multiple myeloma and immunoproliferative neoplasms |  |
| B630.00 | READ | 4944 | Multiple myeloma |  |
| B630.11 | READ | 43552 | Kahler's disease |  |
| B630.12 | READ | 15211 | Myelomatosis |  |
| B630000 | READ | 22158 | Malignant plasma cell neoplasm, extramedullary plasmacytoma |  |
| B630100 | READ | 19028 | Solitary myeloma |  |
| B630200 | READ | 21329 | Plasmacytoma NOS |  |
| B630300 | READ | 46042 | Lambda light chain myeloma |  |
| B631.00 | READ | 39187 | Plasma cell leukaemia |  |
| B63y.00 | READ | 64567 | Other immunoproliferative neoplasms |  |
| B63z.00 | READ | 43450 | Immunoproliferative neoplasm or myeloma NOS |  |
| B64..00 | READ | 19372 | Lymphoid leukaemia |  |
| B64..11 | READ | 4222 | Lymphatic leukaemia |  |
| B640.00 | READ | 4251 | Acute lymphoid leukaemia |  |
| B641.00 | READ | 8625 | Chronic lymphoid leukaemia |  |
| B641.11 | READ | 27790 | Chronic lymphatic leukaemia |  |
| B642.00 | READ | 72774 | Subacute lymphoid leukaemia |  |
| B64y.00 | READ | 49725 | Other lymphoid leukaemia |  |
| B64y100 | READ | 31586 | Prolymphocytic leukaemia |  |
| B64y200 | READ | 37461 | Adult T-cell leukaemia |  |
| B64yz00 | READ | 38331 | Other lymphoid leukaemia NOS |  |
| B64z.00 | READ | 38914 | Lymphoid leukaemia NOS |  |
| B65..00 | READ | 7176 | Myeloid leukaemia |  |
| B650.00 | READ | 4413 | Acute myeloid leukaemia |  |
| B651.00 | READ | 10726 | Chronic myeloid leukaemia |  |
| B651.11 | READ | 31701 | Chronic granulocytic leukaemia |  |
| B651000 | READ | 100786 | Chronic eosinophilic leukaemia |  |
| B651z00 | READ | 27520 | Chronic myeloid leukaemia NOS |  |
| B652.00 | READ | 63475 | Subacute myeloid leukaemia |  |
| B653.00 | READ | 70724 | Myeloid sarcoma |  |
| B653000 | READ | 52327 | Chloroma |  |
| B653100 | READ | 39629 | Granulocytic sarcoma |  |
| B65y100 | READ | 27664 | Acute promyelocytic leukaemia |  |
| B65yz00 | READ | 66089 | Other myeloid leukaemia NOS |  |
| B65z.00 | READ | 33344 | Myeloid leukaemia NOS |  |
| B66..00 | READ | 35875 | Monocytic leukaemia |  |
| B66..12 | READ | 67700 | Monoblastic leukaemia |  |
| B660.00 | READ | 19974 | Acute monocytic leukaemia |  |
| B661.00 | READ | 27458 | Chronic monocytic leukaemia |  |
| B662.00 | READ | 101606 | Subacute monocytic leukaemia |  |
| B66y.00 | READ | 99015 | Other monocytic leukaemia |  |
| B66z.00 | READ | 93342 | Monocytic leukaemia NOS |  |
| B67..00 | READ | 37272 | Other specified leukaemia |  |
| B670.00 | READ | 42539 | Acute erythraemia and erythroleukaemia |  |
| B670.11 | READ | 27340 | Di Guglielmo's disease |  |
| B671.00 | READ | 37468 | Chronic erythraemia |  |
| B671.11 | READ | 63653 | Heilmeyer - Schoner disease |  |
| B672.00 | READ | 57671 | Megakaryocytic leukaemia |  |
| B672.11 | READ | 65777 | Thrombocytic leukaemia |  |
| B673.00 | READ | 65721 | Mast cell leukaemia |  |
| B674.00 | READ | 50858 | Acute panmyelosis |  |
| B675.00 | READ | 28276 | Acute myelofibrosis |  |
| B67y.00 | READ | 94174 | Other and unspecified leukaemia |  |
| B67y000 | READ | 72197 | Lymphosarcoma cell leukaemia |  |
| B67yz00 | READ | 99413 | Other and unspecified leukaemia NOS |  |
| B67z.00 | READ | 30632 | Other specified leukaemia NOS |  |
| B68..00 | READ | 25191 | Leukaemia of unspecified cell type |  |
| B680.00 | READ | 4072 | Acute leukaemia NOS |  |
| B681.00 | READ | 16416 | Chronic leukaemia NOS |  |
| B682.00 | READ | 54793 | Subacute leukaemia NOS |  |
| B68y.00 | READ | 34692 | Other leukaemia of unspecified cell type |  |
| B68z.00 | READ | 4250 | Leukaemia NOS |  |
| B69..00 | READ | 20440 | Myelomonocytic leukaemia |  |
| B690.00 | READ | 61500 | Acute myelomonocytic leukaemia |  |
| B691.00 | READ | 22050 | Chronic myelomonocytic leukaemia |  |
| B6y..00 | READ | 30646 | Malignant neoplasm lymphatic or haematopoietic tissue OS |  |
| B6y0.00 | READ | 6115 | Myeloproliferative disorder |  |
| B6y0.11 | READ | 17056 | Myeloproliferative disease |  |
| B6y1.00 | READ | 39336 | Myelosclerosis with myeloid metaplasia |  |
| B6z..00 | READ | 49301 | Malignant neoplasm lymphatic or haematopoietic tissue NOS |  |
| B6z0.00 | READ | 50290 | Kaposi's sarcoma of lymph nodes |  |
| D415100 | READ | 96439 | Chronic congestive splenamegaly |  |
| D415400 | READ | 66973 | Splenic atrophy |  |
| D415600 | READ | 66980 | Splenic fibrosis |  |
| G74y600 | READ | 66981 | Embolism and thrombosis of the splenic artery |  |
| PK01.00 | READ | 22319 | Absent spleen |  |
| PK01.11 | READ | 10505 | Asplenia |  |

| outcome definition 1: All respiratory diagnoses_broad |
| --- |
| n= 1040 medcodes used |

| **Read_code** | **readoxmisflag** | **medcode** | **desc** |  |
| --- | --- | --- | --- | --- |
| A793.00 | READ | 32037 | Rhinovirus |  |
| A79A.00 | READ | 12573 | Respiratory syncytial virus infection |  |
| A79z.00 | READ | 1041 | Viral infection NOS |  |
| A79z.11 | READ | 3112 | Viral illness |  |
| F51..00 | READ | 5577 | Nonsuppurative otitis media + eustachian tube disorders |  |
| F510.00 | READ | 5887 | Acute non suppurative otitis media |  |
| F510000 | READ | 18371 | Acute otitis media with effusion |  |
| F510011 | READ | 5148 | Acute secretory otitis media |  |
| F510100 | READ | 7730 | Acute serous otitis media |  |
| F510200 | READ | 21012 | Acute mucoid otitis media |  |
| F510300 | READ | 15973 | Acute sanguinous otitis media |  |
| F510400 | READ | 70788 | Acute allergic serous otitis media |  |
| F510600 | READ | 63780 | Acute allergic sanguinous otitis media |  |
| F510z00 | READ | 20374 | Acute nonsuppurative otitis media NOS |  |
| F511.00 | READ | 6559 | Chronic otitis media with effusion, serous |  |
| F511.11 | READ | 24742 | Chronic secretory otitis media, serous |  |
| F511000 | READ | 17308 | Chronic tubotympanic catarrh |  |
| F511100 | READ | 35910 | Serosanguinous chronic otitis media |  |
| F511200 | READ | 3817 | Bilateral chronic serous otitis |  |
| F511300 | READ | 31353 | Unilateral chronic serous otitis |  |
| F511z00 | READ | 33661 | Chronic serous otitis media NOS |  |
| F512.00 | READ | 2686 | Chronic otitis media with effusion, mucoid |  |
| F512.11 | READ | 354 | Glue ear |  |
| F512.12 | READ | 1184 | Chronic secretory otitis media, mucoid |  |
| F512000 | READ | 5539 | Glue ear, unspecified |  |
| F512100 | READ | 62905 | Mucosanguinous chronic otitis media |  |
| F512z00 | READ | 20578 | Chronic mucoid otitis media NOS |  |
| F513.00 | READ | 34348 | Chronic otitis media with effusion, other |  |
| F513000 | READ | 37597 | Chronic allergic otitis media |  |
| F513100 | READ | 25188 | Chronic otitis media with effusion, purulent |  |
| F513111 | READ | 9993 | Chronic secretory otitis media, purulent |  |
| F513z00 | READ | 26085 | Other chronic nonsuppurative otitis media NOS |  |
| F514.00 | READ | 17772 | Unspecified nonsuppurative otitis media |  |
| F514000 | READ | 29845 | Allergic otitis media NOS |  |
| F514100 | READ | 5102 | Serous otitis media NOS |  |
| F514200 | READ | 5390 | Catarrhal otitis media NOS |  |
| F514300 | READ | 21749 | Mucoid otitis media NOS |  |
| F514z00 | READ | 21725 | Nonsuppurative otitis media NOS |  |
| F515.00 | READ | 16121 | Eustachian tube salpingitis |  |
| F515.11 | READ | 7479 | Catarrh - eustachian |  |
| F515000 | READ | 67441 | Unspecified eustachian tube salpingitis |  |
| F515100 | READ | 62907 | Acute eustachian tube salpingitis |  |
| F515200 | READ | 69893 | Chronic eustachian tube salpingitis |  |
| F515z00 | READ | 65039 | Eustachian tube salpingitis NOS |  |
| F516.00 | READ | 17160 | Eustachian tube obstruction |  |
| F516.11 | READ | 2130 | Block - eustachian tube |  |
| F516000 | READ | 21513 | Unspecified eustachian tube obstruction |  |
| F516200 | READ | 71089 | Cartilaginous eustachian tube obstruction |  |
| F516z00 | READ | 29867 | Eustachian tube obstruction NOS |  |
| F517.00 | READ | 38116 | Patulous eustachian tube |  |
| F518.00 | READ | 10517 | Chronic otitis media with effusion, unspecified |  |
| F51y.00 | READ | 35765 | Other eustachian tube disorder |  |
| F51y000 | READ | 535 | Eustachian tube dysfunction |  |
| F51yz00 | READ | 50044 | Other eustachian tube disorder NOS |  |
| F51z.00 | READ | 21010 | Eustachian tube disorder NOS |  |
| F52..00 | READ | 1474 | Suppurative and unspecified otitis media |  |
| F520.00 | READ | 2137 | Acute suppurative otitis media |  |
| F520000 | READ | 10781 | Acute suppurative otitis media tympanic membrane intact |  |
| F520100 | READ | 20669 | Acute suppurative otitis media tympanic membrane ruptured |  |
| F520300 | READ | 61497 | Acute suppurative otitis media due to disease EC |  |
| F520z00 | READ | 20372 | Acute suppurative otitis media NOS |  |
| F521.00 | READ | 17866 | Chronic suppurative otitis media, tubotympanic |  |
| F522.00 | READ | 24590 | Chronic suppurative otitis media, atticoantral |  |
| F523.00 | READ | 1376 | Chronic suppurative otitis media NOS |  |
| F524.00 | READ | 15568 | Purulent otitis media NOS |  |
| F524000 | READ | 20871 | Bilateral suppurative otitis media |  |
| F525.00 | READ | 9973 | Recurrent acute otitis media |  |
| F526.00 | READ | 3694 | Acute left otitis media |  |
| F527.00 | READ | 4348 | Acute right otitis media |  |
| F528.00 | READ | 1134 | Acute bilateral otitis media |  |
| F52z.00 | READ | 267 | Otitis media NOS |  |
| F52z.11 | READ | 1513 | Infection ear |  |
| F53..00 | READ | 11399 | Mastoiditis and related conditions |  |
| F530.00 | READ | 37443 | Acute mastoiditis |  |
| F530.11 | READ | 30216 | Abscess of mastoid |  |
| F530.12 | READ | 28228 | Empyema of mastoid |  |
| F530000 | READ | 48721 | Acute mastoiditis without complications |  |
| F530100 | READ | 50034 | Subperiosteal mastoid abscess |  |
| F530200 | READ | 49277 | Gradenigo's syndrome |  |
| F530300 | READ | 73570 | Acute mastoiditis with other complication |  |
| F530z00 | READ | 11221 | Acute mastoiditis NOS |  |
| F531.00 | READ | 5251 | Chronic mastoiditis |  |
| F531000 | READ | 57689 | Caries of mastoid |  |
| F531100 | READ | 39834 | Post aural mastoid fistula |  |
| F531z00 | READ | 15003 | Chronic mastoiditis NOS |  |
| F532.00 | READ | 60591 | Petrositis |  |
| F533.00 | READ | 40167 | Postmastoidectomy complication |  |
| F533000 | READ | 67380 | Unspecified postmastoidectomy complication |  |
| F533200 | READ | 33277 | Recurrent cholesteatoma postmastoidectomy |  |
| F533300 | READ | 51376 | Postmastoidectomy granulation cavity |  |
| F533z00 | READ | 62843 | Postmastoidectomy complication NOS |  |
| F53y.00 | READ | 41648 | Other mastoid disorders |  |
| F53y000 | READ | 40356 | Postauricular fistula |  |
| F53y100 | READ | 48314 | Other mastoid disorder NOS |  |
| F53z.00 | READ | 2567 | Mastoiditis NOS |  |
| F542.00 | READ | 1707 | Tympanic membrane perforation |  |
| F542.11 | READ | 624 | Ear drum perforation |  |
| F542000 | READ | 38807 | Unspecified tympanic membrane perforation |  |
| F542100 | READ | 33780 | Tympanic membrane central perforation |  |
| F542111 | READ | 40337 | Pars tensa central perforation |  |
| F542200 | READ | 28128 | Tympanic membrane attic perforation |  |
| F542300 | READ | 20577 | Other marginal tympanic membrane perforation |  |
| F542311 | READ | 67809 | Pars tensa marginal perforation |  |
| F542400 | READ | 62962 | Tympanic membrane with multiple perforations |  |
| F542500 | READ | 54708 | Tympanic membrane perforation, more than 50 % |  |
| F542511 | READ | 68681 | Tympanic membrane - total perforation |  |
| F542600 | READ | 32763 | Tympanic membrane perforation, less than 50 % |  |
| F542z00 | READ | 29755 | Tympanic membrane perforation NOS |  |
| F543.00 | READ | 16780 | Tympanic atelectasis |  |
| F54y.00 | READ | 47486 | Other tympanic membrane disorder |  |
| F54y000 | READ | 20731 | Healed tympanic membrane perforation |  |
| F54y100 | READ | 15108 | Atrophic flaccid tympanic membrane NOS |  |
| F54y200 | READ | 89229 | Atrophic nonflaccid tympanic membrane |  |
| F54y300 | READ | 21898 | Retraction of tympanic membrane |  |
| F54yz00 | READ | 31523 | Other tympanic membrane disorder NOS |  |
| F54z.00 | READ | 35779 | Tympanic membrane disorder NOS |  |
| F586.00 | READ | 638 | Otorrhoea |  |
| F587.00 | READ | 731 | Otalgia |  |
| F587.11 | READ | 1135 | Ear pain |  |
| H....00 | READ | 5733 | Respiratory system diseases |  |
| H0...00 | READ | 8025 | Acute respiratory infections |  |
| H00..00 | READ | 3260 | Acute nasopharyngitis |  |
| H00..11 | READ | 368 | Common cold |  |
| H00..12 | READ | 1246 | Coryza - acute |  |
| H00..13 | READ | 6620 | Febrile cold |  |
| H00..14 | READ | 896 | Nasal catarrh - acute |  |
| H00..15 | READ | 9093 | Pyrexial cold |  |
| H00..16 | READ | 3821 | Rhinitis - acute |  |
| H01..00 | READ | 980 | Acute sinusitis |  |
| H01..11 | READ | 243 | Sinusitis |  |
| H010.00 | READ | 7021 | Acute maxillary sinusitis |  |
| H010.11 | READ | 18555 | Antritis - acute |  |
| H011.00 | READ | 8213 | Acute frontal sinusitis |  |
| H012.00 | READ | 15724 | Acute ethmoidal sinusitis |  |
| H013.00 | READ | 38816 | Acute sphenoidal sinusitis |  |
| H014.00 | READ | 94218 | Acute rhinosinusitis |  |
| H01y.00 | READ | 29696 | Other acute sinusitis |  |
| H01y000 | READ | 19284 | Acute pansinusitis |  |
| H01yz00 | READ | 60733 | Other acute sinusitis NOS |  |
| H01z.00 | READ | 33664 | Acute sinusitis NOS |  |
| H02..00 | READ | 893 | Acute pharyngitis |  |
| H02..11 | READ | 6014 | Sore throat NOS |  |
| H02..12 | READ | 6466 | Viral sore throat NOS |  |
| H02..13 | READ | 310 | Throat infection - pharyngitis |  |
| H020.00 | READ | 36219 | Acute gangrenous pharyngitis |  |
| H021.00 | READ | 24708 | Acute phlegmonous pharyngitis |  |
| H022.00 | READ | 21486 | Acute ulcerative pharyngitis |  |
| H023.00 | READ | 17899 | Acute bacterial pharyngitis |  |
| H023000 | READ | 92428 | Acute pneumococcal pharyngitis |  |
| H023100 | READ | 29589 | Acute staphylococcal pharyngitis |  |
| H023z00 | READ | 53395 | Acute bacterial pharyngitis NOS |  |
| H024.00 | READ | 4868 | Acute viral pharyngitis |  |
| H02z.00 | READ | 407 | Acute pharyngitis NOS |  |
| H03..00 | READ | 138 | Acute tonsillitis |  |
| H03..11 | READ | 11499 | Throat infection - tonsillitis |  |
| H03..12 | READ | 2125 | Tonsillitis |  |
| H030.00 | READ | 12010 | Acute erythematous tonsillitis |  |
| H031.00 | READ | 4061 | Acute follicular tonsillitis |  |
| H032.00 | READ | 8452 | Acute ulcerative tonsillitis |  |
| H033.00 | READ | 37409 | Acute catarrhal tonsillitis |  |
| H034.00 | READ | 59986 | Acute gangrenous tonsillitis |  |
| H035.00 | READ | 10156 | Acute bacterial tonsillitis |  |
| H035000 | READ | 58188 | Acute pneumococcal tonsillitis |  |
| H035100 | READ | 64973 | Acute staphylococcal tonsillitis |  |
| H035z00 | READ | 15970 | Acute bacterial tonsillitis NOS |  |
| H036.00 | READ | 9357 | Acute viral tonsillitis |  |
| H037.00 | READ | 1747 | Recurrent acute tonsillitis |  |
| H03z.00 | READ | 20104 | Acute tonsillitis NOS |  |
| H04..00 | READ | 41324 | Acute laryngitis and tracheitis |  |
| H040.00 | READ | 142 | Acute laryngitis |  |
| H040000 | READ | 26038 | Acute oedematous laryngitis |  |
| H040100 | READ | 62885 | Acute ulcerative laryngitis |  |
| H040200 | READ | 10765 | Acute catarrhal laryngitis |  |
| H040300 | READ | 31501 | Acute phlegmonous laryngitis |  |
| H040400 | READ | 43317 | Acute haemophilus influenzae laryngitis |  |
| H040600 | READ | 51562 | Acute suppurative laryngitis |  |
| H040w00 | READ | 5115 | Acute viral laryngitis unspecified |  |
| H040x00 | READ | 52756 | Acute bacterial laryngitis unspecified |  |
| H040z00 | READ | 22720 | Acute laryngitis NOS |  |
| H041.00 | READ | 1257 | Acute tracheitis |  |
| H041000 | READ | 12476 | Acute tracheitis without obstruction |  |
| H041100 | READ | 68867 | Acute tracheitis with obstruction |  |
| H041z00 | READ | 16313 | Acute tracheitis NOS |  |
| H042.00 | READ | 10087 | Acute laryngotracheitis |  |
| H042.11 | READ | 1285 | Laryngotracheitis |  |
| H042000 | READ | 25259 | Acute laryngotracheitis without obstruction |  |
| H042100 | READ | 69898 | Acute laryngotracheitis with obstruction |  |
| H042z00 | READ | 24471 | Acute laryngotracheitis NOS |  |
| H043.00 | READ | 10641 | Acute epiglottitis (non strep) |  |
| H043.11 | READ | 69489 | Viral epiglottitis |  |
| H043000 | READ | 65650 | Acute epiglottitis without obstruction |  |
| H043100 | READ | 48669 | Acute epiglottitis with obstruction |  |
| H043200 | READ | 38128 | Acute obstructive laryngitis |  |
| H043211 | READ | 19431 | Croup |  |
| H043z00 | READ | 892 | Acute epiglottitis NOS |  |
| H044.00 | READ | 1142 | Croup |  |
| H04z.00 | READ | 16120 | Acute laryngitis and tracheitis NOS |  |
| H05..00 | READ | 26010 | Other acute upper respiratory infections |  |
| H050.00 | READ | 18908 | Acute laryngopharyngitis |  |
| H051.00 | READ | 6294 | Acute upper respiratory tract infection |  |
| H052.00 | READ | 21415 | Pharyngotracheitis |  |
| H053.00 | READ | 10093 | Tracheopharyngitis |  |
| H054.00 | READ | 4221 | Recurrent upper respiratory tract infection |  |
| H055.00 | READ | 4718 | Pharyngolaryngitis |  |
| H05y.00 | READ | 15628 | Other upper respiratory infections of multiple sites |  |
| H05z.00 | READ | 76 | Upper respiratory infection NOS |  |
| H05z.11 | READ | 2637 | Upper respiratory tract infection NOS |  |
| H05z.12 | READ | 6421 | Viral upper respiratory tract infection NOS |  |
| H06..00 | READ | 29669 | Acute bronchitis and bronchiolitis |  |
| H060.00 | READ | 312 | Acute bronchitis |  |
| H060.11 | READ | 5978 | Acute wheezy bronchitis |  |
| H060000 | READ | 50396 | Acute fibrinous bronchitis |  |
| H060100 | READ | 101775 | Acute membranous bronchitis |  |
| H060200 | READ | 71370 | Acute pseudomembranous bronchitis |  |
| H060300 | READ | 11072 | Acute purulent bronchitis |  |
| H060400 | READ | 21145 | Acute croupous bronchitis |  |
| H060500 | READ | 11101 | Acute tracheobronchitis |  |
| H060600 | READ | 9043 | Acute pneumococcal bronchitis |  |
| H060700 | READ | 43362 | Acute streptococcal bronchitis |  |
| H060800 | READ | 21492 | Acute haemophilus influenzae bronchitis |  |
| H060900 | READ | 49794 | Acute neisseria catarrhalis bronchitis |  |
| H060A00 | READ | 31886 | Acute bronchitis due to mycoplasma pneumoniae |  |
| H060B00 | READ | 93153 | Acute bronchitis due to coxsackievirus |  |
| H060C00 | READ | 29273 | Acute bronchitis due to parainfluenza virus |  |
| H060D00 | READ | 48593 | Acute bronchitis due to respiratory syncytial virus |  |
| H060E00 | READ | 64890 | Acute bronchitis due to rhinovirus |  |
| H060F00 | READ | 65916 | Acute bronchitis due to echovirus |  |
| H060v00 | READ | 55391 | Subacute bronchitis unspecified |  |
| H060w00 | READ | 1382 | Acute viral bronchitis unspecified |  |
| H060x00 | READ | 24800 | Acute bacterial bronchitis unspecified |  |
| H060z00 | READ | 20198 | Acute bronchitis NOS |  |
| H061.00 | READ | 1019 | Acute bronchiolitis |  |
| H061000 | READ | 54533 | Acute capillary bronchiolitis |  |
| H061100 | READ | 41589 | Acute obliterating bronchiolitis |  |
| H061200 | READ | 17185 | Acute bronchiolitis with bronchospasm |  |
| H061300 | READ | 69192 | Acute exudative bronchiolitis |  |
| H061400 | READ | 6181 | Obliterating fibrous bronchiolitis |  |
| H061500 | READ | 18451 | Acute bronchiolitis due to respiratory syncytial virus |  |
| H061600 | READ | 66228 | Acute bronchiolitis due to other specified organisms |  |
| H061z00 | READ | 17917 | Acute bronchiolitis NOS |  |
| H062.00 | READ | 6124 | Acute lower respiratory tract infection |  |
| H06z.00 | READ | 41137 | Acute bronchitis or bronchiolitis NOS |  |
| H06z000 | READ | 2581 | Chest infection NOS |  |
| H06z011 | READ | 68 | Chest infection |  |
| H06z100 | READ | 3358 | Lower resp tract infection |  |
| H06z111 | READ | 293 | Respiratory tract infection |  |
| H06z112 | READ | 37447 | Acute lower respiratory tract infection |  |
| H06z200 | READ | 4899 | Recurrent chest infection |  |
| H07..00 | READ | 2476 | Chest cold |  |
| H0y..00 | READ | 23640 | Other specified acute respiratory infections |  |
| H0z..00 | READ | 21113 | Acute respiratory infection NOS |  |
| H1...00 | READ | 32802 | Other upper respiratory tract diseases |  |
| H10..00 | READ | 1141 | Deviated nasal septum - acquired |  |
| H10..11 | READ | 32884 | Deflected nasal septum |  |
| H10..12 | READ | 4734 | DNS - deviated nasal septum |  |
| H11..00 | READ | 4686 | Nasal polyps |  |
| H110.00 | READ | 977 | Polyp of nasal cavity |  |
| H110000 | READ | 31837 | Choanal polyp |  |
| H110100 | READ | 47466 | Nasopharyngeal polyp |  |
| H110z00 | READ | 14749 | Polyp of nasal cavity NOS |  |
| H111.00 | READ | 44934 | Polypoid sinus degeneration |  |
| H111000 | READ | 36229 | Woakes' ethmoiditis |  |
| H111z00 | READ | 50528 | Polypoid sinus degeneration NOS |  |
| H11y.00 | READ | 56372 | Other polyp of sinus |  |
| H11y.11 | READ | 19742 | Nasal sinus polyps |  |
| H11y000 | READ | 91482 | Polyp of frontal sinus |  |
| H11y100 | READ | 11744 | Polyp of ethmoidal sinus |  |
| H11y200 | READ | 35897 | Polyp of maxillary sinus |  |
| H11y211 | READ | 4341 | Antral (maxillary) polyp |  |
| H11y300 | READ | 61281 | Polyp of sphenoidal sinus |  |
| H11yz00 | READ | 44518 | Other polyp of sinus NOS |  |
| H11z.00 | READ | 14888 | Nasal polyp NOS |  |
| H12..00 | READ | 10083 | Chronic pharyngitis and nasopharyngitis |  |
| H120.00 | READ | 774 | Chronic rhinitis |  |
| H120.11 | READ | 8975 | Catarrh unspecified |  |
| H120000 | READ | 28589 | Chronic simple rhinitis |  |
| H120100 | READ | 805 | Chronic catarrhal rhinitis |  |
| H120111 | READ | 2769 | Catarrhal child |  |
| H120200 | READ | 15553 | Chronic hypertrophic rhinitis |  |
| H120300 | READ | 16441 | Chronic atrophic rhinitis |  |
| H120400 | READ | 16158 | Chronic infective rhinitis |  |
| H120500 | READ | 42556 | Chronic ulcerative rhinitis |  |
| H120600 | READ | 42065 | Chronic membranous rhinitis |  |
| H120z00 | READ | 14645 | Chronic rhinitis NOS |  |
| H121.00 | READ | 4324 | Chronic pharyngitis |  |
| H121.11 | READ | 16814 | Sore throat - chronic |  |
| H121000 | READ | 47426 | Simple chronic pharyngitis |  |
| H121100 | READ | 38879 | Atrophic pharyngitis |  |
| H121200 | READ | 21562 | Granular pharyngitis |  |
| H121300 | READ | 15794 | Hypertrophic pharyngitis |  |
| H121400 | READ | 56361 | Pharyngitis keratosa |  |
| H121500 | READ | 30569 | Pharyngitis sicca |  |
| H121600 | READ | 47269 | Chronic follicular pharyngitis |  |
| H121z00 | READ | 14926 | Chronic pharyngitis NOS |  |
| H122.00 | READ | 12667 | Chronic nasopharyngitis |  |
| H12z.00 | READ | 54657 | Chronic pharyngitis and nasopharyngitis NOS |  |
| H13..00 | READ | 2257 | Chronic sinusitis |  |
| H13..11 | READ | 10546 | Chronic rhinosinusitis |  |
| H130.00 | READ | 4433 | Chronic maxillary sinusitis |  |
| H130.11 | READ | 33437 | Antritis - chronic |  |
| H130.12 | READ | 3624 | Maxillary sinusitis |  |
| H131.00 | READ | 15163 | Chronic frontal sinusitis |  |
| H131.11 | READ | 2984 | Frontal sinusitis |  |
| H132.00 | READ | 1674 | Chronic ethmoidal sinusitis |  |
| H133.00 | READ | 48703 | Chronic sphenoidal sinusitis |  |
| H134.00 | READ | 29429 | Fistula of nasal sinus |  |
| H135.00 | READ | 17173 | Recurrent sinusitis |  |
| H13y.00 | READ | 49548 | Other chronic sinusitis |  |
| H13y000 | READ | 39501 | Chronic pansinusitis |  |
| H13y100 | READ | 2233 | Pansinusitis |  |
| H13yz00 | READ | 54375 | Other chronic sinusitis NOS |  |
| H13z.00 | READ | 5437 | Chronic sinusitis NOS |  |
| H14..00 | READ | 21000 | Chronic tonsil and adenoid disease |  |
| H14..11 | READ | 7601 | Adenoid disease - chronic |  |
| H14..12 | READ | 16864 | Tonsil disease - chronic |  |
| H140.00 | READ | 1667 | Chronic tonsillitis |  |
| H140.11 | READ | 21051 | Chronic adenoiditis |  |
| H141.00 | READ | 3549 | Tonsil and/or adenoid hypertrophy |  |
| H141.11 | READ | 17408 | Adenoid hypertrophy |  |
| H141.12 | READ | 18238 | Enlargement of tonsil or adenoid |  |
| H141000 | READ | 24164 | Hypertrophy of tonsils and adenoids |  |
| H141100 | READ | 2158 | Hypertrophy of tonsils alone |  |
| H141200 | READ | 2723 | Hypertrophy of adenoids alone |  |
| H141z00 | READ | 29502 | Hypertrophy of tonsils and adenoids NOS |  |
| H142.00 | READ | 68301 | Adenoid vegetations |  |
| H143.00 | READ | 9328 | Chronic adenotonsillitis |  |
| H14y.00 | READ | 54475 | Other chronic diseases of tonsils and adenoids |  |
| H14y000 | READ | 33464 | Calculus of tonsil |  |
| H14y011 | READ | 96642 | Amygdalolith |  |
| H14y100 | READ | 41793 | Cicatrix of tonsil |  |
| H14y300 | READ | 41574 | Tonsillar tag |  |
| H14y400 | READ | 35641 | Tonsil ulcer |  |
| H14y500 | READ | 36462 | Caseous tonsillitis |  |
| H14y600 | READ | 35249 | Lingular tonsillitis |  |
| H14y700 | READ | 17656 | Cyst of tonsil |  |
| H14y711 | READ | 9259 | Tonsillar cyst |  |
| H14yz00 | READ | 3430 | Other chronic diseases of tonsils and adenoids NOS |  |
| H14z.00 | READ | 43146 | Chronic tonsil and adenoid disease NOS |  |
| H14z000 | READ | 5047 | Chronic tonsil disease NOS |  |
| H14z100 | READ | 43633 | Chronic adenoid disease NOS |  |
| H15..00 | READ | 3605 | Peritonsillar abscess - quinsy |  |
| H15..11 | READ | 911 | Quinsy |  |
| H16..00 | READ | 11942 | Chronic laryngitis and laryngotracheitis |  |
| H160.00 | READ | 15231 | Chronic laryngitis |  |
| H160000 | READ | 25436 | Chronic simple laryngitis |  |
| H160100 | READ | 17513 | Chronic catarrhal laryngitis |  |
| H160200 | READ | 49839 | Chronic hypertrophic laryngitis |  |
| H160300 | READ | 73546 | Chronic atrophic laryngitis |  |
| H160400 | READ | 4276 | Laryngitis sicca |  |
| H160500 | READ | 32834 | Congested larynx |  |
| H160z00 | READ | 41268 | Chronic laryngitis NOS |  |
| H161.00 | READ | 7318 | Chronic laryngotracheitis |  |
| H16z.00 | READ | 6173 | Chronic laryngitis NOS |  |
| H18..00 | READ | 1108 | Vasomotor rhinitis |  |
| H1y..00 | READ | 39041 | Other specified diseases of upper respiratory tract |  |
| H1y0.00 | READ | 3557 | Nasal turbinate hypertrophy |  |
| H1y1.00 | READ | 14788 | Other nasal cavity and sinus disease |  |
| H1y1.11 | READ | 31699 | Nasal cavity disease NOS |  |
| H1y1.12 | READ | 4195 | Nasal vestibulitis |  |
| H1y1.13 | READ | 4722 | Sinus disease NOS |  |
| H1y1.14 | READ | 5690 | Perforation of nasal septum NOS |  |
| H1y1000 | READ | 15228 | Nasal septum abscess |  |
| H1y1011 | READ | 17224 | Boil in nose |  |
| H1y1100 | READ | 14814 | Nasal septum necrosis |  |
| H1y1200 | READ | 15851 | Nasal septum ulcer |  |
| H1y1300 | READ | 7896 | Nasal sinus cyst |  |
| H1y1400 | READ | 5224 | Nasal sinus mucocele |  |
| H1y1500 | READ | 40820 | Rhinolith |  |
| H1y1600 | READ | 6257 | Nasal obstruction |  |
| H1y1700 | READ | 32205 | Nasal septal granuloma |  |
| H1y1800 | READ | 19303 | Adhesions of nasal cavity |  |
| H1y1900 | READ | 38819 | Nasal septal adhesions |  |
| H1y1A00 | READ | 22862 | Nasal furuncle |  |
| H1y1z00 | READ | 2097 | Nasal cavity and sinus disease NOS |  |
| H1y1z11 | READ | 1401 | Nasal obstruction |  |
| H1y1z12 | READ | 3110 | Nasal congestion |  |
| H1y1z13 | READ | 9483 | Sinus congestion |  |
| H1y1z14 | READ | 1309 | Nasal infection |  |
| H1y1z15 | READ | 2255 | Sore nostril |  |
| H1y2.00 | READ | 10355 | Other pharyngeal disease NEC |  |
| H1y2.11 | READ | 37288 | Other nasopharyngeal disease NEC |  |
| H1y2000 | READ | 41878 | Pharyngeal disease unspecified |  |
| H1y2100 | READ | 25156 | Pharynx or nasopharynx cellulitis |  |
| H1y2200 | READ | 12231 | Parapharyngeal abscess |  |
| H1y2300 | READ | 27279 | Retropharyngeal abscess |  |
| H1y2400 | READ | 19948 | Pharynx or nasopharynx oedema |  |
| H1y2500 | READ | 1965 | Pharynx or nasopharynx cyst |  |
| H1y2600 | READ | 14710 | Pharynx or nasopharynx abscess |  |
| H1y2700 | READ | 22782 | Vallecular cyst |  |
| H1y2800 | READ | 63411 | Pharyngocutaneous fistula |  |
| H1y2z00 | READ | 15324 | Other pharyngeal disease NOS |  |
| H1y3.00 | READ | 10373 | Paralysis of vocal cords or larynx |  |
| H1y3.11 | READ | 42382 | Larynx paralysis |  |
| H1y3000 | READ | 15303 | Paralysis of larynx unspecified |  |
| H1y3011 | READ | 71108 | Glottis paralysis |  |
| H1y3012 | READ | 60715 | Laryngoplegia |  |
| H1y3100 | READ | 35070 | Unilateral partial vocal cord paralysis |  |
| H1y3200 | READ | 44959 | Unilateral total vocal cord paralysis |  |
| H1y3300 | READ | 33816 | Bilateral partial vocal cord paralysis |  |
| H1y3400 | READ | 67128 | Bilateral total vocal cord paralysis |  |
| H1y3500 |  | 99791 | Vocal cord palsy |  |
| H1y3z00 | READ | 60114 | Laryngoplegia NOS |  |
| H1y4.00 | READ | 15776 | Polyp of vocal cord or larynx |  |
| H1y4000 | READ | 2434 | Polyp of vocal cord |  |
| H1y4100 | READ | 34936 | Polyp of larynx |  |
| H1y4z00 | READ | 70927 | Polyp of vocal cord or larynx NOS |  |
| H1y5.00 | READ | 21869 | Other vocal cord disease |  |
| H1y5000 | READ | 93040 | Abscess of vocal cords |  |
| H1y5100 | READ | 64484 | Cellulitis of vocal cords |  |
| H1y5200 | READ | 41779 | Granuloma of vocal cords |  |
| H1y5300 | READ | 18422 | Leukoplakia of vocal cords |  |
| H1y5400 | READ | 15398 | Singers' chorditis |  |
| H1y5500 | READ | 89649 | Fibrinous chorditis |  |
| H1y5600 | READ | 10342 | Vocal cord nodule |  |
| H1y5612 | READ | 88276 | Nodular chorditis-singers'node |  |
| H1y5613 | READ | 28145 | Singer's node |  |
| H1y5700 | READ | 72601 | Chorditis vocalis inferior |  |
| H1y5800 | READ | 91780 | Hyperaemic vocal cords |  |
| H1y5900 | READ | 32551 | Dysplasia of vocal cord |  |
| H1y5x00 | READ | 44400 | Chorditis NOS |  |
| H1y5z00 | READ | 22769 | Other vocal cord disease NOS |  |
| H1y6.00 | READ | 15408 | Oedema of larynx |  |
| H1y6000 | READ | 28585 | Oedema of glottis |  |
| H1y6100 | READ | 48704 | Subglottic oedema |  |
| H1y6200 | READ | 12236 | Supraglottic oedema |  |
| H1y6300 | READ | 1596 | Reinke's oedema of the vocal cords |  |
| H1y6z00 | READ | 54492 | Oedema of larynx NOS |  |
| H1y7.00 | READ | 19173 | Other diseases of larynx NEC |  |
| H1y7000 | READ | 46679 | Disease of larynx unspecified |  |
| H1y7100 | READ | 61518 | Cellulitis of larynx |  |
| H1y7200 | READ | 16155 | Perichondritis of larynx |  |
| H1y7300 | READ | 3571 | Stenosis of larynx |  |
| H1y7311 | READ | 94535 | Laryngismus |  |
| H1y7312 | READ | 101858 | Stridulus - laryngismus |  |
| H1y7313 | READ | 22214 | Subglottic stenosis |  |
| H1y7400 | READ | 4585 | Laryngeal spasm |  |
| H1y7500 | READ | 34656 | Abscess of larynx |  |
| H1y7600 | READ | 56563 | Necrosis of larynx |  |
| H1y7700 | READ | 20051 | Obstruction of larynx NOS |  |
| H1y7800 | READ | 19955 | Pachyderma of larynx |  |
| H1y7900 | READ | 36010 | Ulcer of larynx |  |
| H1y7A00 | READ | 18242 | Cyst of larynx |  |
| H1y7B00 | READ | 3949 | Laryngomalacia |  |
| H1y7C00 | READ | 16246 | Laryngismus |  |
| H1y7C11 | READ | 40603 | Laryngismus stridulus |  |
| H1y7D00 | READ | 31868 | Dysplasia of larynx |  |
| H1y7E00 | READ | 9493 | Acquired laryngocele |  |
| H1y7z00 | READ | 15055 | Other disease of larynx NOS |  |
| H1y8.00 | READ | 30513 | Upper respiratory tract hypersensitivity reaction NOS |  |
| H1y9.00 | READ | 17624 | Nasal septal spur |  |
| H1yz.00 | READ | 57308 | Other upper respiratory tract diseases NOS |  |
| H1yz000 | READ | 51893 | Abscess of trachea |  |
| H1yz100 | READ | 97505 | Cicatrix of trachea |  |
| H1yz200 | READ | 43432 | Upper airway resistance syndrome |  |
| H1yzz00 | READ | 16116 | Other upper respiratory tract disease NOS |  |
| H1z..00 | READ | 29466 | Upper respiratory tract disease NOS |  |
| H2...00 | READ | 10086 | Pneumonia and influenza |  |
| H20..00 | READ | 5202 | Viral pneumonia |  |
| H20..11 | READ | 9389 | Chest infection - viral pneumonia |  |
| H200.00 | READ | 67836 | Pneumonia due to adenovirus |  |
| H201.00 | READ | 31269 | Pneumonia due to respiratory syncytial virus |  |
| H202.00 | READ | 36675 | Pneumonia due to parainfluenza virus |  |
| H20y.00 | READ | 33478 | Viral pneumonia NEC |  |
| H20y000 | READ | 46052 | Severe acute respiratory syndrome |  |
| H20z.00 | READ | 14976 | Viral pneumonia NOS |  |
| H21..00 | READ | 1849 | Lobar (pneumococcal) pneumonia |  |
| H21..11 | READ | 29166 | Chest infection - pneumococcal pneumonia |  |
| H22..00 | READ | 28634 | Other bacterial pneumonia |  |
| H22..11 | READ | 22795 | Chest infection - other bacterial pneumonia |  |
| H220.00 | READ | 23546 | Pneumonia due to klebsiella pneumoniae |  |
| H221.00 | READ | 30591 | Pneumonia due to pseudomonas |  |
| H222.00 | READ | 37881 | Pneumonia due to haemophilus influenzae |  |
| H222.11 | READ | 48804 | Pneumonia due to haemophilus influenzae |  |
| H223.00 | READ | 12423 | Pneumonia due to streptococcus |  |
| H223000 | READ | 63858 | Pneumonia due to streptococcus, group B |  |
| H224.00 | READ | 5612 | Pneumonia due to staphylococcus |  |
| H22y.00 | READ | 50867 | Pneumonia due to other specified bacteria |  |
| H22y000 | READ | 65419 | Pneumonia due to escherichia coli |  |
| H22y011 | READ | 60299 | E.coli pneumonia |  |
| H22y100 | READ | 45425 | Pneumonia due to proteus |  |
| H22y200 | READ | 12061 | Pneumonia - Legionella |  |
| H22yX00 | READ | 52384 | Pneumonia due to other aerobic gram-negative bacteria |  |
| H22yz00 | READ | 43884 | Pneumonia due to bacteria NOS |  |
| H22z.00 | READ | 23095 | Bacterial pneumonia NOS |  |
| H23..00 | READ | 25694 | Pneumonia due to other specified organisms |  |
| H23..11 | READ | 30653 | Chest infection - pneumonia organism OS |  |
| H230.00 | READ | 60119 | Pneumonia due to Eaton's agent |  |
| H231.00 | READ | 1576 | Pneumonia due to mycoplasma pneumoniae |  |
| H232.00 | READ | 73735 | Pneumonia due to pleuropneumonia like organisms |  |
| H233.00 | READ | 17025 | Chlamydial pneumonia |  |
| H23z.00 | READ | 34251 | Pneumonia due to specified organism NOS |  |
| H24..00 | READ | 40498 | Pneumonia with infectious diseases EC |  |
| H24..11 | READ | 24316 | Chest infection with infectious disease EC |  |
| H240.00 | READ | 41034 | Pneumonia with measles |  |
| H241.00 | READ | 43286 | Pneumonia with cytomegalic inclusion disease |  |
| H242.00 | READ | 62623 | Pneumonia with ornithosis |  |
| H243.00 | READ | 30437 | Pneumonia with whooping cough |  |
| H243.11 | READ | 35082 | Pneumonia with pertussis |  |
| H246.00 | READ | 34274 | Pneumonia with aspergillosis |  |
| H247000 | READ | 52071 | Pneumonia with candidiasis |  |
| H247z00 | READ | 53969 | Pneumonia with systemic mycosis NOS |  |
| H24y.00 | READ | 69782 | Pneumonia with other infectious diseases EC |  |
| H24y000 | READ | 61623 | Pneumonia with actinomycosis |  |
| H24y100 | READ | 67901 | Pneumonia with nocardiasis |  |
| H24y200 | READ | 27519 | Pneumonia with pneumocystis carinii |  |
| H24y300 | READ | 60482 | Pneumonia with Q-fever |  |
| H24y400 | READ | 72182 | Pneumonia with salmonellosis |  |
| H24y500 | READ | 98782 | Pneumonia with toxoplasmosis |  |
| H24y600 | READ | 49398 | Pneumonia with typhoid fever |  |
| H24y700 | READ | 23726 | Pneumonia with varicella |  |
| H24yz00 | READ | 70559 | Pneumonia with other infectious diseases EC NOS |  |
| H24z.00 | READ | 66362 | Pneumonia with infectious diseases EC NOS |  |
| H25..00 | READ | 886 | Bronchopneumonia due to unspecified organism |  |
| H25..11 | READ | 16287 | Chest infection - unspecified bronchopneumonia |  |
| H26..00 | READ | 572 | Pneumonia due to unspecified organism |  |
| H26..11 | READ | 19400 | Chest infection - pnemonia due to unspecified organism |  |
| H260.00 | READ | 9639 | Lobar pneumonia due to unspecified organism |  |
| H260000 | READ | 8318 | Lung consolidation |  |
| H261.00 | READ | 3683 | Basal pneumonia due to unspecified organism |  |
| H262.00 | READ | 34300 | Postoperative pneumonia |  |
| H263.00 | READ | 38065 | Pneumonitis, unspecified |  |
| H27..00 | READ | 556 | Influenza |  |
| H270.00 | READ | 15912 | Influenza with pneumonia |  |
| H270.11 | READ | 29457 | Chest infection - influenza with pneumonia |  |
| H270000 | READ | 13573 | Influenza with bronchopneumonia |  |
| H270100 | READ | 62632 | Influenza with pneumonia, influenza virus identified |  |
| H270z00 | READ | 35745 | Influenza with pneumonia NOS |  |
| H271.00 | READ | 43625 | Influenza with other respiratory manifestation |  |
| H271000 | READ | 15774 | Influenza with laryngitis |  |
| H271100 | READ | 29617 | Influenza with pharyngitis |  |
| H271z00 | READ | 23488 | Influenza with respiratory manifestations NOS |  |
| H27y.00 | READ | 47472 | Influenza with other manifestations |  |
| H27y000 | READ | 46157 | Influenza with encephalopathy |  |
| H27y100 | READ | 14791 | Influenza with gastrointestinal tract involvement |  |
| H27yz00 | READ | 31363 | Influenza with other manifestations NOS |  |
| H27z.00 | READ | 16388 | Influenza NOS |  |
| H27z.11 | READ | 2157 | Flu like illness |  |
| H27z.12 | READ | 5947 | Influenza like illness |  |
| H28..00 | READ | 5324 | Atypical pneumonia |  |
| H29..00 | READ | 94930 | Avian influenza |  |
| H2A..00 |  | 98129 | Influenza due to Influenza A virus subtype H1N1 |  |
| H2A..11 |  | 98102 | Influenza A (H1N1) swine flu |  |
| H2y..00 | READ | 11849 | Other specified pneumonia or influenza |  |
| H2z..00 | READ | 6094 | Pneumonia or influenza NOS |  |
| H3...00 | READ | 1001 | Chronic obstructive pulmonary disease |  |
| H3...11 | READ | 998 | Chronic obstructive airways disease |  |
| H30..00 | READ | 148 | Bronchitis unspecified |  |
| H30..11 | READ | 17359 | Chest infection - unspecified bronchitis |  |
| H30..12 | READ | 7092 | Recurrent wheezy bronchitis |  |
| H300.00 | READ | 3163 | Tracheobronchitis NOS |  |
| H301.00 | READ | 1934 | Laryngotracheobronchitis |  |
| H302.00 | READ | 152 | Wheezy bronchitis |  |
| H30z.00 | READ | 3480 | Bronchitis NOS |  |
| H31..00 | READ | 3243 | Chronic bronchitis |  |
| H310.00 | READ | 25603 | Simple chronic bronchitis |  |
| H310000 | READ | 15626 | Chronic catarrhal bronchitis |  |
| H310100 | READ | 16717 | Smokers' cough |  |
| H310z00 | READ | 61118 | Simple chronic bronchitis NOS |  |
| H311.00 | READ | 11150 | Mucopurulent chronic bronchitis |  |
| H311000 | READ | 40159 | Purulent chronic bronchitis |  |
| H311100 | READ | 37959 | Fetid chronic bronchitis |  |
| H311z00 | READ | 61513 | Mucopurulent chronic bronchitis NOS |  |
| H312.00 | READ | 27819 | Obstructive chronic bronchitis |  |
| H312000 | READ | 5798 | Chronic asthmatic bronchitis |  |
| H312011 | READ | 5909 | Chronic wheezy bronchitis |  |
| H312100 | READ | 14798 | Emphysematous bronchitis |  |
| H312200 | READ | 1446 | Acute exacerbation of chronic obstructive airways disease |  |
| H312300 | READ | 26125 | Bronchiolitis obliterans |  |
| H312z00 | READ | 44525 | Obstructive chronic bronchitis NOS |  |
| H313.00 | READ | 24248 | Mixed simple and mucopurulent chronic bronchitis |  |
| H31y.00 | READ | 66043 | Other chronic bronchitis |  |
| H31y000 | READ | 23618 | Chronic tracheitis |  |
| H31y100 | READ | 45089 | Chronic tracheobronchitis |  |
| H31yz00 | READ | 68066 | Other chronic bronchitis NOS |  |
| H31z.00 | READ | 15157 | Chronic bronchitis NOS |  |
| H32..00 | READ | 794 | Emphysema |  |
| H320.00 | READ | 26306 | Chronic bullous emphysema |  |
| H320000 | READ | 56860 | Segmental bullous emphysema |  |
| H320100 | READ | 68662 | Zonal bullous emphysema |  |
| H320200 | READ | 60188 | Giant bullous emphysema |  |
| H320300 | READ | 99536 | Bullous emphysema with collapse |  |
| H320z00 | READ | 23492 | Chronic bullous emphysema NOS |  |
| H321.00 | READ | 46578 | Panlobular emphysema |  |
| H322.00 | READ | 10980 | Centrilobular emphysema |  |
| H32y.00 | READ | 40788 | Other emphysema |  |
| H32y000 | READ | 92955 | Acute vesicular emphysema |  |
| H32y100 | READ | 70787 | Atrophic (senile) emphysema |  |
| H32y111 | READ | 59263 | Acute interstitial emphysema |  |
| H32y200 | READ | 63479 | MacLeod's unilateral emphysema |  |
| H32yz00 | READ | 16410 | Other emphysema NOS |  |
| H32z.00 | READ | 33450 | Emphysema NOS |  |
| H33..00 | READ | 78 | Asthma |  |
| H33..11 | READ | 1555 | Bronchial asthma |  |
| H330.00 | READ | 7146 | Extrinsic (atopic) asthma |  |
| H330.11 | READ | 2290 | Allergic asthma |  |
| H330.12 | READ | 1208 | Childhood asthma |  |
| H330.13 | READ | 15248 | Hay fever with asthma |  |
| H330.14 | READ | 7731 | Pollen asthma |  |
| H330000 | READ | 14777 | Extrinsic asthma without status asthmaticus |  |
| H330011 | READ | 5627 | Hay fever with asthma |  |
| H330100 | READ | 27926 | Extrinsic asthma with status asthmaticus |  |
| H330111 | READ | 6707 | Extrinsic asthma with asthma attack |  |
| H330z00 | READ | 45782 | Extrinsic asthma NOS |  |
| H331.00 | READ | 5267 | Intrinsic asthma |  |
| H331.11 | READ | 3665 | Late onset asthma |  |
| H331000 | READ | 29325 | Intrinsic asthma without status asthmaticus |  |
| H331100 | READ | 58196 | Intrinsic asthma with status asthmaticus |  |
| H331111 | READ | 18323 | Intrinsic asthma with asthma attack |  |
| H331z00 | READ | 45073 | Intrinsic asthma NOS |  |
| H332.00 | READ | 25796 | Mixed asthma |  |
| H333.00 | READ | 185 | Acute exacerbation of asthma |  |
| H334.00 | READ | 40823 | Brittle asthma |  |
| H33z.00 | READ | 4442 | Asthma unspecified |  |
| H33z.11 | READ | 32727 | Hyperreactive airways disease |  |
| H33z000 | READ | 4892 | Status asthmaticus NOS |  |
| H33z011 | READ | 233 | Severe asthma attack |  |
| H33z100 | READ | 232 | Asthma attack |  |
| H33z111 | READ | 8335 | Asthma attack NOS |  |
| H33z200 | READ | 12987 | Late-onset asthma |  |
| H33zz00 | READ | 16070 | Asthma NOS |  |
| H33zz11 | READ | 4606 | Exercise induced asthma |  |
| H33zz12 | READ | 21232 | Allergic asthma NEC |  |
| H34..00 | READ | 2195 | Bronchiectasis |  |
| H340.00 | READ | 20364 | Recurrent bronchiectasis |  |
| H341.00 | READ | 41491 | Post-infective bronchiectasis |  |
| H34z.00 | READ | 32679 | Bronchiectasis NOS |  |
| H350.00 | READ | 15588 | Farmers' lung |  |
| H351.00 | READ | 62200 | Bagassosis |  |
| H352.00 | READ | 27345 | Bird-fancier's lung |  |
| H352000 | READ | 54822 | Budgerigar-fanciers' lung |  |
| H352100 | READ | 31447 | Pigeon-fanciers' lung |  |
| H352z00 | READ | 36240 | Bird-fancier's lung NOS |  |
| H353.00 | READ | 93206 | Suberosis ( cork-handlers' lung ) |  |
| H354.00 | READ | 67709 | Malt workers' lung |  |
| H355.00 | READ | 41694 | Mushroom workers' lung |  |
| H356.00 | READ | 56652 | Maple bark strippers' lung |  |
| H357.00 | READ | 26278 | pneumonitis |  |
| H35y000 | READ | 91989 | Cheese-washers' lung |  |
| H35y100 | READ | 53943 | Coffee-workers' lung |  |
| H35y200 | READ | 70286 | Fish-meal workers' lung |  |
| H35y500 | READ | 45427 | Pituitary snuff-takers' disease |  |
| H35y600 | READ | 93353 | Sequoiosis (red-cedar asthma) |  |
| H35y700 | READ | 39478 | Wood asthma |  |
| H35y800 | READ | 69452 | Air-conditioner and humidifier lung |  |
| H35z100 | READ | 11833 | Hypersensitivity pneumonitis NOS |  |
| H36..00 | READ | 10863 | Mild chronic obstructive pulmonary disease |  |
| H37..00 | READ | 10802 | Moderate chronic obstructive pulmonary disease |  |
| H38..00 | READ | 9876 | Severe chronic obstructive pulmonary disease |  |
| H39..00 | READ | 93568 | Very severe chronic obstructive pulmonary disease |  |
| H3y..00 | READ | 12166 | Other specified chronic obstructive airways disease |  |
| H3y..11 | READ | 67040 | Other specified chronic obstructive pulmonary disease |  |
| H3y0.00 | READ | 21061 | Chronic obstruct pulmonary dis with acute lower resp infectn |  |
| H3y1.00 | READ | 7884 | Chron obstruct pulmonary dis wth acute exacerbation, unspec |  |
| H3z..00 | READ | 5710 | Chronic obstructive airways disease NOS |  |
| H3z..11 | READ | 37247 | Chronic obstructive pulmonary disease NOS |  |
| H4...00 | READ | 21973 | Lung disease due to external agents |  |
| H4...11 | READ | 25013 | Pneumoconioses |  |
| H4...12 | READ | 21257 | Occupational lung disease |  |
| H40..00 | READ | 19492 | Coal workers' pneumoconiosis |  |
| H41..00 | READ | 8303 | Asbestosis |  |
| H410.00 | READ | 5005 | Pleural plaque disease due to asbestosis |  |
| H410.11 |  | 100994 | Asbestos-induced pleural plaque |  |
| H41z.00 | READ | 51410 | Asbestosis NOS |  |
| H42..00 | READ | 46460 | Silica and silicate pneumoconiosis |  |
| H420.00 | READ | 60805 | Talc pneumoconiosis |  |
| H421.00 | READ | 62233 | Simple silicosis |  |
| H422.00 | READ | 71853 | Complicated silicosis |  |
| H423.00 | READ | 89206 | Massive silicotic fibrosis |  |
| H42z.00 | READ | 23446 | Silica pneumoconiosis NOS |  |
| H43..00 | READ | 65376 | Pneumoconiosis due to other inorganic dust |  |
| H431.00 | READ | 94894 | Bauxite fibrosis of lung |  |
| H432.00 | READ | 49194 | Berylliosis |  |
| H433.00 | READ | 94575 | Graphite fibrosis of lung |  |
| H434.00 | READ | 30235 | Siderosis |  |
| H435.00 | READ | 93577 | Stannosis |  |
| H43z.00 | READ | 23461 | Pneumoconiosis due to inorganic dust NOS |  |
| H44..00 | READ | 60313 | Pneumopathy due to inhalation of other dust |  |
| H440.00 | READ | 37365 | Byssinosis |  |
| H441.00 | READ | 26442 | Cannabinosis |  |
| H44z.00 | READ | 73414 | Pneumopathy due to inhalation of other dust NOS |  |
| H45..00 | READ | 31423 | Pneumoconiosis NOS |  |
| H450.00 | READ | 63172 | Pneumoconiosis associated with tuberculosis |  |
| H46..00 | READ | 31722 | Respiratory disease due to chemical fumes and vapours |  |
| H460.00 | READ | 38639 | Bronchitis and pneumonitis due to chemical fumes |  |
| H460000 | READ | 54830 | Acute bronchitis due to chemical fumes |  |
| H460100 | READ | 49025 | Acute pneumonitis due to chemical fumes |  |
| H460z00 | READ | 55758 | Bronchitis and pneumonitis due to chemical fumes NOS |  |
| H461.00 | READ | 62227 | Acute pulmonary oedema due to chemical fumes |  |
| H462.00 | READ | 20448 | Upper respiratory inflammation due to chemical fumes |  |
| H463.00 | READ | 52937 | Other acute respiratory diseases due to chemical fumes |  |
| H464.00 | READ | 47142 | Chronic respiratory conditions due to chemical fumes |  |
| H464000 | READ | 64721 | Chronic emphysema due to chemical fumes |  |
| H464100 | READ | 63216 | Obliterative bronchiolitis due to chemical fumes |  |
| H464200 | READ | 47782 | Chronic pulmonary fibrosis due to chemical fumes |  |
| H464z00 | READ | 70815 | Chronic respiratory conditions due to chemical fumes NOS |  |
| H46z.00 | READ | 48647 | Respiratory conditions due to chemical fumes NOS |  |
| H46zz00 | READ | 33663 | Respiratory conditions due to chemical fumes NOS |  |
| H47..00 | READ | 9711 | Pneumonitis due to inhalation of solids or liquids |  |
| H47..11 | READ | 10992 | Aspiration pneumonitis |  |
| H470.00 | READ | 3847 | Pneumonitis due to inhalation of food or vomitus |  |
| H470.11 |  | 101204 | Aspiration pneumonia |  |
| H470000 | READ | 41781 | Pneumonitis due to inhalation of regurgitated food |  |
| H470100 | READ | 59083 | Pneumonitis due to inhalation of gastric secretions |  |
| H470200 | READ | 66104 | Pneumonitis due to inhalation of milk |  |
| H470211 | READ | 30996 | Milk inhalation pneumonitis |  |
| H470300 | READ | 45948 | Pneumonitis due to inhalation of vomitus |  |
| H470311 | READ | 56385 | Vomit inhalation pneumonitis |  |
| H470312 | READ | 25054 | Aspiration pneumonia due to vomit |  |
| H470z00 | READ | 33837 | Pneumonitis due to inhalation of food or vomitus NOS |  |
| H471.00 | READ | 56647 | Pneumonitis due to inhalation of oil or essence |  |
| H471000 | READ | 41015 | Lipoid pneumonia (exogenous) |  |
| H471z00 | READ | 66773 | Pneumonitis due to inhalation of oil or essence NOS |  |
| H472.00 | READ | 50876 | Asp pneumonitis due to anaesthesia during labour and deliv |  |
| H47y.00 | READ | 47504 | Pneumonitis due to inhalation of other solid or liquid |  |
| H47y000 | READ | 47684 | Detergent asthma |  |
| H47yz00 | READ | 54252 | Pneumonitis due to inhalation of solid or liquid NOS |  |
| H47z.00 | READ | 46066 | Pneumonitis due to inhalation of solid or liquid NOS |  |
| H48..00 | READ | 43285 | Progressive massive fibrosis |  |
| H4y..00 | READ | 38985 | Other specified lung diseases due to external agent |  |
| H4y0.00 | READ | 51713 | Acute pulmonary radiation disease |  |
| H4y0000 | READ | 18130 | Acute radiation pneumonitis |  |
| H4y1.00 | READ | 69914 | Chronic pulmonary radiation disease |  |
| H4y1000 | READ | 22536 | Chronic pulmonary fibrosis following radiation |  |
| H4y1z00 | READ | 50374 | Chronic pulmonary radiation disease NOS |  |
| H4y2.00 | READ | 44015 | Drug-induced interstitial lung disorders |  |
| H4y2000 | READ | 53205 | Acute drug-induced interstitial lung disorders |  |
| H4y2100 | READ | 43417 | Chronic drug-induced interstitial lung disorders |  |
| H4yy.00 | READ | 55557 | Other external agent causing respiratory condition |  |
| H4yz.00 | READ | 62707 | External agent causing respiratory conditions NOS |  |
| H4z..00 | READ | 34001 | Lung disease due to external agents NOS |  |
| H5...00 | READ | 11494 | Other respiratory system diseases |  |
| H50..00 | READ | 2375 | Empyema |  |
| H500.00 | READ | 59340 | Empyema with fistula |  |
| H500000 | READ | 66856 | Empyema with bronchocutaneous fistula |  |
| H500100 | READ | 34651 | Empyema with bronchopleural fistula |  |
| H500400 | READ | 99547 | Empyema with pleural fistula NOS |  |
| H501.00 | READ | 53494 | Empyema with no fistula |  |
| H501000 | READ | 6624 | Pleural abscess |  |
| H501100 | READ | 15932 | Thorax abscess NOS |  |
| H501200 | READ | 44425 | Pleural empyema |  |
| H501300 | READ | 38052 | Lung empyema NOS |  |
| H501400 | READ | 49452 | Purulent pleurisy |  |
| H501500 | READ | 46628 | Pyopneumothorax |  |
| H501600 | READ | 59587 | Pyothorax |  |
| H50z.00 | READ | 34282 | Empyema NOS |  |
| H51..00 | READ | 978 | Pleurisy |  |
| H510.00 | READ | 23482 | Pleurisy without effusion or active tuberculosis |  |
| H510000 | READ | 15024 | Adhesion of pleura or lung |  |
| H510100 | READ | 3409 | Thickening of pleura |  |
| H510200 | READ | 4428 | Calcification of pleura |  |
| H510300 | READ | 19207 | Acute dry pleurisy |  |
| H510400 | READ | 40819 | Diaphragmatic pleurisy |  |
| H510500 | READ | 37599 | Basal pleurisy |  |
| H510600 | READ | 62895 | Chronic dry pleurisy |  |
| H510700 | READ | 47354 | Fibrinous pleurisy |  |
| H510800 | READ | 67452 | Sterile pleurisy |  |
| H510900 | READ | 32818 | Pneumococcal pleurisy |  |
| H510A00 | READ | 31603 | Staphylococcal pleurisy |  |
| H510B00 | READ | 69352 | Streptococcal pleurisy |  |
| H510C00 |  | 102492 | Pleural plaque |  |
| H510z00 | READ | 31645 | Pleurisy without effusion or active tuberculosis NOS |  |
| H511.00 | READ | 31689 | Bacterial pleurisy with effusion |  |
| H511000 | READ | 43345 | Pneumococcal pleurisy with effusion |  |
| H511100 | READ | 93010 | Staphylococcal pleurisy with effusion |  |
| H511z00 | READ | 44842 | Bacterial pleurisy with effusion NOS |  |
| H51y.00 | READ | 30065 | Other pleural effusion excluding mention of tuberculosis |  |
| H51y000 | READ | 57092 | Encysted pleurisy |  |
| H51y100 | READ | 4493 | Haemopneumothorax |  |
| H51y200 | READ | 6830 | Haemothorax |  |
| H51y300 | READ | 29188 | Hydropneumothorax |  |
| H51y400 | READ | 33577 | Hydrothorax |  |
| H51y500 | READ | 43807 | Chylous effusion |  |
| H51y600 | READ | 45236 | Fibrothorax |  |
| H51y700 | READ | 7593 | Malignant pleural effusion |  |
| H51yz00 | READ | 18081 | Other pleural effusion |  |
| H51z.00 | READ | 947 | Pleural effusion NOS |  |
| H51z000 | READ | 60142 | Exudative pleurisy NOS |  |
| H51z100 | READ | 68470 | Serofibrinous pleurisy NOS |  |
| H51z200 | READ | 55211 | Serous pleurisy NOS |  |
| H51zz00 | READ | 9559 | Pleural effusion NOS |  |
| H52..00 | READ | 1550 | Pneumothorax |  |
| H520.00 | READ | 27821 | Spontaneous tension pneumothorax |  |
| H52y.00 | READ | 23766 | Other spontaneous pneumothorax |  |
| H52y000 | READ | 36017 | Acute pneumothorax NOS |  |
| H52y100 | READ | 70492 | Chronic pneumothorax |  |
| H52yz00 | READ | 2486 | Other spontaneous pneumothorax NOS |  |
| H52yz11 | READ | 9101 | Spontaneous pneumothorax NOS |  |
| H52z.00 | READ | 28695 | Pneumothorax NOS |  |
| H53..00 | READ | 21185 | Abscess of lung and mediastinum |  |
| H530.00 | READ | 29005 | Abscess of lung |  |
| H530000 | READ | 33730 | Single lung abscess |  |
| H530100 | READ | 37711 | Multiple lung abscess |  |
| H530200 | READ | 57667 | Gangrenous pneumonia |  |
| H530300 | READ | 35189 | Abscess of lung with pneumonia |  |
| H530z00 | READ | 11202 | Abscess of lung NOS |  |
| H531.00 | READ | 20127 | Abscess of mediastinum |  |
| H53z.00 | READ | 34659 | Abscess of lung and mediastinum NOS |  |
| H54..00 | READ | 30214 | Pulmonary congestion and hypostasis |  |
| H540.00 | READ | 38297 | Pulmonary hypostasis |  |
| H540000 | READ | 23333 | Hypostatic pneumonia |  |
| H540100 | READ | 24356 | Hypostatic bronchopneumonia |  |
| H540z00 | READ | 46405 | Pulmonary hypostasis NOS |  |
| H541.00 | READ | 1585 | Pulmonary congestion |  |
| H541000 | READ | 26082 | Chronic pulmonary oedema |  |
| H541z00 | READ | 7321 | Pulmonary oedema NOS |  |
| H54z.00 | READ | 61229 | Pulmonary congestion and hypostasis NOS |  |
| H55..00 | READ | 7791 | Postinflammatory pulmonary fibrosis |  |
| H55..11 | READ | 40953 | Cirrhosis of lung |  |
| H56..00 | READ | 31806 | Other alveolar and parietoalveolar disease |  |
| H560.00 | READ | 61119 | Pulmonary alveolar proteinosis |  |
| H561.00 | READ | 27348 | Idiopathic pulmonary haemosiderosis |  |
| H562.00 | READ | 68814 | Pulmonary alveolar microlithiasis |  |
| H563.00 | READ | 6837 | Idiopathic fibrosing alveolitis |  |
| H563.11 | READ | 63174 | Hamman - Rich syndrome |  |
| H563.12 | READ | 5519 | Cryptogenic fibrosing alveolitis |  |
| H563000 | READ | 94136 | Alveolar capillary block |  |
| H563100 | READ | 6051 | Diffuse pulmonary fibrosis |  |
| H563z00 | READ | 28229 | Idiopathic fibrosing alveolitis NOS |  |
| H564.00 | READ | 22835 | Bronchiolitis obliterans organising pneumonia |  |
| H56y.00 | READ | 54308 | Other alveolar and parietoalveolar disease |  |
| H56y000 | READ | 61991 | Endogenous lipoid pneumonia |  |
| H56y100 | READ | 4910 | Interstitial pneumonia |  |
| H56yz00 | READ | 33830 | Other alveolar and parietoalveolar disease NOS |  |
| H56z.00 | READ | 15815 | Alveolar and parietoalveolar disease NOS |  |
| H57..00 | READ | 49044 | Lung involvement in diseases EC |  |
| H570.00 | READ | 9954 | Rheumatoid lung |  |
| H571.00 | READ | 64799 | Rheumatic pneumonia |  |
| H572.00 | READ | 94996 | Lung disease with systemic sclerosis |  |
| H57y.00 | READ | 58791 | Lung disease with diseases EC |  |
| H57y000 | READ | 54010 | Pulmonary amyloidosis |  |
| H57y100 | READ | 42940 | Lung disease with polymyositis |  |
| H57y200 | READ | 3859 | Pulmonary sarcoidosis |  |
| H57y300 | READ | 47364 | Lung disease with Sjogren's disease |  |
| H57y400 | READ | 31564 | Lung disease with systemic lupus erythematosus |  |
| H57y500 | READ | 96655 | Lung disease with syphilis |  |
| H57yz00 | READ | 63912 | Lung disease with diseases EC NOS |  |
| H58..00 | READ | 6563 | Other diseases of lung |  |
| H580.00 | READ | 3094 | Pulmonary collapse with atelectasis |  |
| H580.11 | READ | 7324 | Atelectasis |  |
| H580.12 | READ | 8370 | Collapse of lung |  |
| H580000 | READ | 30406 | Post operative atelectasis |  |
| H581.00 | READ | 22905 | Interstitial emphysema |  |
| H581.11 | READ | 35432 | Pneumomediastinum |  |
| H582.00 | READ | 54893 | Compensatory emphysema |  |
| H583.00 | READ | 22915 | Pulmonary eosinophilia |  |
| H583000 | READ | 31319 | Loeffler's syndrome |  |
| H583100 | READ | 16439 | Tropical eosinophilia |  |
| H583z00 | READ | 20269 | Pulmonary eosinophilia NOS |  |
| H584.00 | READ | 558 | Acute pulmonary oedema unspecified |  |
| H584.11 | READ | 48466 | Acute oedema of lung, unspecified |  |
| H584000 | READ | 39706 | Postoperative pulmonary oedema |  |
| H584z00 | READ | 5293 | Acute pulmonary oedema NOS |  |
| H585.00 | READ | 65000 | Trauma and post-operative pulmonary insufficiency |  |
| H585.11 | READ | 57678 | Adult respiratory distress syndrome |  |
| H585000 | READ | 93880 | Pulmonary insufficiency following shock |  |
| H585100 | READ | 72221 | Pulmonary insufficiency following surgery |  |
| H585200 | READ | 96754 | Pulmonary insufficiency following trauma |  |
| H585300 | READ | 24848 | Adult respiratory distress syndrome |  |
| H58y.00 | READ | 51069 | Other lung disease NEC |  |
| H58y000 | READ | 24466 | Broncholithiasis |  |
| H58y100 | READ | 36706 | Calcification of lung |  |
| H58y200 | READ | 99158 | Pulmolithiasis |  |
| H58y300 | READ | 8317 | Interstitial lung disease NEC |  |
| H58y400 | READ | 6140 | Squamous metaplasia of lung |  |
| H58yz00 | READ | 11573 | Other lung disease NEC NOS |  |
| H58z.00 | READ | 1813 | Lung disease NOS |  |
| H59..00 | READ | 25249 | Respiratory failure |  |
| H590.00 | READ | 37961 | Acute respiratory failure |  |
| H591.00 | READ | 24814 | Chronic respiratory failure |  |
| H592.00 | READ | 94946 | Chronic type 1 respiratory failure |  |
| H593.00 | READ | 94486 | Chronic type 2 respiratory failure |  |
| H5B..00 | READ | 23779 | Sleep apnoea |  |
| H5B0.00 | READ | 20748 | Obstructive sleep apnoea |  |
| H5C..00 | READ | 4229 | Choking due to airways obstruction |  |
| H5X..00 | READ | 11665 | Pleural condition, unspecified |  |
| H5y..00 | READ | 42929 | Other specified diseases of respiratory system |  |
| H5y0.00 | READ | 1477 | Tracheostomy complication |  |
| H5y0000 | READ | 51717 | Tracheostomy haemorrhage |  |
| H5y0100 | READ | 54077 | Tracheostomy sepsis |  |
| H5y0200 | READ | 54147 | Tracheostomy stenosis |  |
| H5y0300 | READ | 39008 | Tracheostomy obstruction |  |
| H5y0400 | READ | 51167 | Tracheo-oesophageal fistula following tracheostomy |  |
| H5y0z00 | READ | 54076 | Tracheostomy complication NOS |  |
| H5y1.00 | READ | 37944 | Other diseases of trachea and bronchus NEC |  |
| H5y1.11 | READ | 35218 | Other bronchus disease |  |
| H5y1.12 | READ | 2911 | Other trachea disease |  |
| H5y1000 | READ | 68690 | Calcification of trachea |  |
| H5y1100 | READ | 38605 | Calcification of bronchus |  |
| H5y1200 | READ | 29172 | Stenosis of trachea |  |
| H5y1300 | READ | 15572 | Stenosis of bronchus |  |
| H5y1400 | READ | 96952 | Ulcer of trachea |  |
| H5y1500 | READ | 59317 | Ulcer of bronchus |  |
| H5y1600 | READ | 9653 | Bronchospasm |  |
| H5y1z00 | READ | 56017 | Diseases of trachea and bronchus NEC NOS |  |
| H5y2.00 | READ | 28862 | Mediastinitis |  |
| H5y3.00 | READ | 46447 | Other diseases of mediastinum, NEC |  |
| H5y3000 | READ | 49709 | Fibrosis of mediastinum |  |
| H5y3100 | READ | 58735 | Hernia of mediastinum |  |
| H5y3200 | READ | 62439 | Retraction of mediastinum |  |
| H5y3z00 | READ | 14914 | Diseases of mediastinum, NEC NOS |  |
| H5y4.00 | READ | 18052 | Disorders of diaphragm |  |
| H5y4000 | READ | 48673 | Diaphragmatitis |  |
| H5y4100 | READ | 10832 | Paralysis of diaphragm |  |
| H5y4z00 | READ | 42188 | Disorders of diaphragm NOS |  |
| H5yy.00 | READ | 16080 | Other diseases of respiratory system NEC |  |
| H5yy.11 | READ | 7074 | Respiratory infection NOS |  |
| H5yz.00 | READ | 4492 | Other diseases of respiratory system NOS |  |
| H5z..00 | READ | 17928 | Respiratory system diseases NOS |  |
| Hy...00 | READ | 38987 | Other specified diseases of respiratory system |  |
| Hy0..00 | READ | 54080 | Postprocedural respiratory disorders |  |
| Hy00.00 | READ | 65340 | Acute pulmonary insufficiency following thoracic surgery |  |
| Hy01.00 | READ | 57425 | Acute pulmonary insufficiency following nonthoracic surgery |  |
| Hy02.00 | READ | 59188 | Chronic pulmonary insufficiency following surgery |  |
| Hy03.00 | READ | 69941 | Postprocedural subglottic stenosis |  |
| Hy04.00 | READ | 55674 | Postprocedural respiratory failure |  |
| Hy0y.00 | READ | 66425 | Other post procedural respiratory disorder |  |
| Hyu..00 | READ | 62085 | [X]Additional respiratory disease classification terms |  |
| Hyu0.00 | READ | 53055 | [X]Acute upper respiratory infections |  |
| Hyu0000 | READ | 97330 | [X]Other acute sinusitis |  |
| Hyu0100 | READ | 93964 | [X]Acute pharyngitis due to other specified organisms |  |
| Hyu0200 | READ | 73118 | [X]Acute tonsillitis due to other specified organisms |  |
| Hyu0400 | READ | 98257 | [X]Flu+oth respiratory manifestations,'flu virus identified |  |
| Hyu0500 | READ | 97936 | [X]Influenza+other manifestations,influenza virus identified |  |
| Hyu0600 | READ | 97605 | [X]Influenza+oth respiratory manifestatns,virus not identifd |  |
| Hyu0700 | READ | 97279 | [X]Influenza+other manifestations, virus not identified |  |
| Hyu0800 | READ | 52520 | [X]Other viral pneumonia |  |
| Hyu0A00 | READ | 63763 | [X]Other bacterial pneumonia |  |
| Hyu0B00 | READ | 98381 | [X]Pneumonia due to other specified infectious organisms |  |
| Hyu0D00 | READ | 53947 | [X]Pneumonia in viral diseases classified elsewhere |  |
| Hyu0H00 | READ | 53753 | [X]Other pneumonia, organism unspecified |  |
| Hyu1.00 | READ | 66397 | [X]Other acute lower respiratory infections |  |
| Hyu1000 | READ | 73100 | [X]Acute bronchitis due to other specified organisms |  |
| Hyu1100 | READ | 99214 | [X]Acute bronchiolitis due to other specified organisms |  |
| Hyu2.00 | READ | 68281 | [X]Other diseases of the upper respiratory tract |  |
| Hyu2200 | READ | 63733 | [X]Other chronic sinusitis |  |
| Hyu2300 | READ | 69765 | [X]Other polyp of sinus |  |
| Hyu2400 | READ | 45181 | [X]Other specified disorders of nose and nasal sinuses |  |
| Hyu2600 | READ | 73255 | [X]Other diseases of vocal cords |  |
| Hyu2A00 | READ | 70941 | [X]Other specified diseases of upper respiratory tract |  |
| Hyu3.00 | READ | 67278 | [X]Chronic lower respiratory diseases |  |
| Hyu3000 | READ | 66058 | [X]Other emphysema |  |
| Hyu3100 | READ | 65733 | [X]Other specified chronic obstructive pulmonary disease |  |
| Hyu4200 | READ | 53851 | [X]Airway disease due to other specific organic dusts |  |
| Hyu4700 | READ | 99232 | [X]Pneumonitis due to inhalation of other solids and liquids |  |
| Hyu4900 | READ | 100577 | [X]Respiratory conditions/other specified external agents |  |
| Hyu5.00 | READ | 54706 | [X]Other resp diseases principally affecting interstitium |  |
| Hyu5000 | READ | 65060 | [X]Other interstitial pulmonary diseases with fibrosis |  |
| Hyu5100 | READ | 91912 | [X]Other specified interstitial pulmonary diseases |  |
| Hyu5200 | READ | 71906 | [X]Hepatopulmonary syndrome |  |
| Hyu6.00 | READ | 54669 | [X]Suppurative & necrotic conditions of lower respir tract |  |
| Hyu7.00 | READ | 73184 | [X]Other diseases of the pleura |  |
| Hyu7000 | READ | 52850 | [X]Pleural effusion in conditions classified elsewhere |  |
| Hyu7100 | READ | 98427 | [X]Other spontaneous pneumothorax |  |
| Hyu7200 | READ | 53808 | [X]Other pneumothorax |  |
| Hyu7300 | READ | 73095 | [X]Other specified pleural conditions |  |
| Hyu7400 | READ | 53885 | [X]Pleural condition, unspecified |  |
| Hyu8.00 | READ | 70630 | [X]Other diseases of the respiratory system |  |
| Hyu8000 | READ | 96697 | [X]Other postprocedural respiratory disorders |  |
| Hyu8100 | READ | 50264 | [X]Other disorders of lung |  |
| Hyu8200 | READ | 66460 | [X]Other specified respiratory disorders |  |
| Hyu8400 | READ | 54739 | [X]Respiratory disorders in other diseases CE |  |
| Hz...00 | READ | 23002 | Respiratory system diseases NOS |  |
| Q31y512 | READ | 1390 | Snuffles |  |
| R003000 | READ | 614 | [D]Convulsions, febrile |  |
| R006.00 | READ | 2389 | [D]Pyrexia of unknown origin |  |
| R006.11 | READ | 17989 | [D]Fever of unknown origin |  |
| R006000 | READ | 23349 | [D]Chills with fever |  |
| R006100 | READ | 20119 | [D]Hyperpyrexia NOS |  |
| R006200 | READ | 1020 | [D]Fever NOS |  |
| R006300 | READ | 22444 | [D]Persistent fever |  |
| R006z00 | READ | 20367 | [D]Pyrexia of unknown origin NOS |  |
| R06..00 | READ | 6475 | [D]Respiratory system and chest symptoms |  |
| R060.00 | READ | 39958 | [D]Respiratory abnormalities |  |
| R060000 | READ | 36347 | [D]Respiratory symptom, unspecified |  |
| R060100 | READ | 2100 | [D]Hyperventilation |  |
| R060200 | READ | 11451 | [D]Orthopnoea |  |
| R060300 | READ | 820 | [D]Tachypnoea |  |
| R060400 | READ | 982 | [D]Apnoea |  |
| R060500 | READ | 27400 | [D]Cheyne-Stokes respiration |  |
| R060600 | READ | 2563 | [D]Respiratory distress |  |
| R060700 | READ | 9297 | [D]Respiratory insufficiency |  |
| R060800 | READ | 741 | [D]Shortness of breath |  |
| R060900 | READ | 2210 | [D]Wheezing |  |
| R060A00 | READ | 3092 | [D]Dyspnoea |  |
| R060B00 | READ | 2578 | [D]Snoring |  |
| R060C00 | READ | 18737 | [D]Yawning |  |
| R060D00 | READ | 735 | [D]Breathlessness |  |
| R060E00 |  | 100954 | [D]Mild wheeze |  |
| R060F00 |  | 101037 | [D]Moderate wheeze |  |
| R060G00 |  | 101073 | [D]Severe wheeze |  |
| R060H00 |  | 101421 | [D]Very severe wheeze |  |
| R060z00 | READ | 2395 | [D]Respiratory abnormalities NOS |  |
| R060z11 | READ | 4342 | [D]Mouth breather |  |
| R061.00 | READ | 3456 | [D]Stridor |  |
| R062.00 | READ | 1160 | [D]Cough |  |
| R062000 | READ | 9799 | [D]Cough syncope |  |
| R063.00 | READ | 2244 | [D]Haemoptysis |  |
| R063000 | READ | 8239 | [D]Cough with haemorrhage |  |
| R063100 | READ | 7285 | [D]Pulmonary haemorrhage NOS |  |
| R063z00 | READ | 33742 | [D]Haemoptysis NOS |  |
| R064.00 | READ | 1251 | [D]Abnormal sputum |  |
| R064000 | READ | 20086 | [D]Sputum abnormal - amount |  |
| R064100 | READ | 15430 | [D]Sputum abnormal - colour |  |
| R064200 | READ | 44214 | [D]Sputum abnormal - odour |  |
| R064300 | READ | 36515 | [D]Abnormal sputum - tenacious |  |
| R064z00 | READ | 23582 | [D]Abnormal sputum NOS |  |
| R065.00 | READ | 2584 | [D]Chest pain |  |
| R065000 | READ | 544 | [D]Chest pain, unspecified |  |
| R065011 | READ | 29490 | [D] Retrosternal chest pain |  |
| R065100 | READ | 20481 | [D]Precordial pain |  |
| R065200 | READ | 14823 | [D]Anterior chest wall pain |  |
| R065300 | READ | 14819 | [D]Painful respiration NOS |  |
| R065400 | READ | 18183 | [D]Pleuritic pain |  |
| R065500 | READ | 24761 | [D]Pleurodynia |  |
| R065600 | READ | 7878 | [D]Chest discomfort |  |
| R065700 | READ | 15528 | [D]Chest pressure |  |
| R065800 | READ | 1270 | [D]Chest tightness |  |
| R065900 | READ | 19199 | [D]Parasternal chest pain |  |
| R065A00 | READ | 3518 | [D]Musculoskeletal chest pain |  |
| R065B00 | READ | 9340 | [D]Non cardiac chest pain |  |
| R065B14 | READ | 7844 | [D]Non-cardiac chest pain |  |
| R065C00 | READ | 21082 | [D]Retrosternal chest pain |  |
| R065D00 | READ | 50477 | [D]Central chest pain |  |
| R065z00 | READ | 3796 | [D]Chest pain NOS |  |
| R066.00 | READ | 16002 | [D]Swelling, mass and lump of chest |  |
| R066000 | READ | 15285 | [D]Chest swelling |  |
| R066100 | READ | 1853 | [D]Chest lump |  |
| R066200 | READ | 8371 | [D]Chest mass |  |
| R066z00 | READ | 57679 | [D]Swelling, mass or lump of chest NOS |  |
| R067.00 | READ | 20107 | [D]Abnormal chest sounds |  |
| R067000 | READ | 12520 | [D]Percussion of chest abnormal |  |
| R067100 | READ | 16375 | [D]Friction sounds, chest |  |
| R067200 | READ | 36426 | [D]Rales |  |
| R067300 | READ | 63034 | [D]Tympany, chest |  |
| R067z00 | READ | 19937 | [D]Abnormal chest sounds NOS |  |
| R068.00 | READ | 3151 | [D]Hiccough |  |
| R06z.00 | READ | 47638 | [D]Other respiratory system and chest symptoms |  |
| R06z000 | READ | 439 | [D]Breath-holding spell |  |
| R06zz00 | READ | 16468 | [D]Respiratory system and chest symptoms NOS |  |
| R107.00 | READ | 1571 | [D]Unspecified viraemia |  |

| outcome definition 2: Pneumonia and Influenza |
| --- |
| n= 88 medcodes used |

| **Read_code** | **readoxmisflag** | **medcode** | **desc** |  |
| --- | --- | --- | --- | --- |
| H2...00 | READ | 10086 | Pneumonia and influenza |  |
| H20..00 | READ | 5202 | Viral pneumonia |  |
| H20..11 | READ | 9389 | Chest infection - viral pneumonia |  |
| H200.00 | READ | 67836 | Pneumonia due to adenovirus |  |
| H201.00 | READ | 31269 | Pneumonia due to respiratory syncytial virus |  |
| H202.00 | READ | 36675 | Pneumonia due to parainfluenza virus |  |
| H20y.00 | READ | 33478 | Viral pneumonia NEC |  |
| H20y000 | READ | 46052 | Severe acute respiratory syndrome |  |
| H20z.00 | READ | 14976 | Viral pneumonia NOS |  |
| H21..00 | READ | 1849 | Lobar (pneumococcal) pneumonia |  |
| H21..11 | READ | 29166 | Chest infection - pneumococcal pneumonia |  |
| H22..00 | READ | 28634 | Other bacterial pneumonia |  |
| H22..11 | READ | 22795 | Chest infection - other bacterial pneumonia |  |
| H220.00 | READ | 23546 | Pneumonia due to klebsiella pneumoniae |  |
| H221.00 | READ | 30591 | Pneumonia due to pseudomonas |  |
| H222.00 | READ | 37881 | Pneumonia due to haemophilus influenzae |  |
| H222.11 | READ | 48804 | Pneumonia due to haemophilus influenzae |  |
| H223.00 | READ | 12423 | Pneumonia due to streptococcus |  |
| H223000 | READ | 63858 | Pneumonia due to streptococcus, group B |  |
| H224.00 | READ | 5612 | Pneumonia due to staphylococcus |  |
| H22y.00 | READ | 50867 | Pneumonia due to other specified bacteria |  |
| H22y000 | READ | 65419 | Pneumonia due to escherichia coli |  |
| H22y011 | READ | 60299 | E.coli pneumonia |  |
| H22y100 | READ | 45425 | Pneumonia due to proteus |  |
| H22y200 | READ | 12061 | Pneumonia - Legionella |  |
| H22yX00 | READ | 52384 | Pneumonia due to other aerobic gram-negative bacteria |  |
| H22yz00 | READ | 43884 | Pneumonia due to bacteria NOS |  |
| H22z.00 | READ | 23095 | Bacterial pneumonia NOS |  |
| H23..00 | READ | 25694 | Pneumonia due to other specified organisms |  |
| H23..11 | READ | 30653 | Chest infection - pneumonia organism OS |  |
| H230.00 | READ | 60119 | Pneumonia due to Eaton's agent |  |
| H231.00 | READ | 1576 | Pneumonia due to mycoplasma pneumoniae |  |
| H232.00 | READ | 73735 | Pneumonia due to pleuropneumonia like organisms |  |
| H233.00 | READ | 17025 | Chlamydial pneumonia |  |
| H23z.00 | READ | 34251 | Pneumonia due to specified organism NOS |  |
| H24..00 | READ | 40498 | Pneumonia with infectious diseases EC |  |
| H24..11 | READ | 24316 | Chest infection with infectious disease EC |  |
| H240.00 | READ | 41034 | Pneumonia with measles |  |
| H241.00 | READ | 43286 | Pneumonia with cytomegalic inclusion disease |  |
| H242.00 | READ | 62623 | Pneumonia with ornithosis |  |
| H243.00 | READ | 30437 | Pneumonia with whooping cough |  |
| H243.11 | READ | 35082 | Pneumonia with pertussis |  |
| H246.00 | READ | 34274 | Pneumonia with aspergillosis |  |
| H247000 | READ | 52071 | Pneumonia with candidiasis |  |
| H247z00 | READ | 53969 | Pneumonia with systemic mycosis NOS |  |
| H24y.00 | READ | 69782 | Pneumonia with other infectious diseases EC |  |
| H24y000 | READ | 61623 | Pneumonia with actinomycosis |  |
| H24y100 | READ | 67901 | Pneumonia with nocardiasis |  |
| H24y200 | READ | 27519 | Pneumonia with pneumocystis carinii |  |
| H24y300 | READ | 60482 | Pneumonia with Q-fever |  |
| H24y400 | READ | 72182 | Pneumonia with salmonellosis |  |
| H24y500 | READ | 98782 | Pneumonia with toxoplasmosis |  |
| H24y600 | READ | 49398 | Pneumonia with typhoid fever |  |
| H24y700 | READ | 23726 | Pneumonia with varicella |  |
| H24yz00 | READ | 70559 | Pneumonia with other infectious diseases EC NOS |  |
| H24z.00 | READ | 66362 | Pneumonia with infectious diseases EC NOS |  |
| H25..00 | READ | 886 | Bronchopneumonia due to unspecified organism |  |
| H25..11 | READ | 16287 | Chest infection - unspecified bronchopneumonia |  |
| H26..00 | READ | 572 | Pneumonia due to unspecified organism |  |
| H26..11 | READ | 19400 | Chest infection - pnemonia due to unspecified organism |  |
| H260.00 | READ | 9639 | Lobar pneumonia due to unspecified organism |  |
| H260000 | READ | 8318 | Lung consolidation |  |
| H261.00 | READ | 3683 | Basal pneumonia due to unspecified organism |  |
| H262.00 | READ | 34300 | Postoperative pneumonia |  |
| H263.00 | READ | 38065 | Pneumonitis, unspecified |  |
| H27..00 | READ | 556 | Influenza |  |
| H270.00 | READ | 15912 | Influenza with pneumonia |  |
| H270.11 | READ | 29457 | Chest infection - influenza with pneumonia |  |
| H270000 | READ | 13573 | Influenza with bronchopneumonia |  |
| H270100 | READ | 62632 | Influenza with pneumonia, influenza virus identified |  |
| H270z00 | READ | 35745 | Influenza with pneumonia NOS |  |
| H271.00 | READ | 43625 | Influenza with other respiratory manifestation |  |
| H271000 | READ | 15774 | Influenza with laryngitis |  |
| H271100 | READ | 29617 | Influenza with pharyngitis |  |
| H271z00 | READ | 23488 | Influenza with respiratory manifestations NOS |  |
| H27y.00 | READ | 47472 | Influenza with other manifestations |  |
| H27y000 | READ | 46157 | Influenza with encephalopathy |  |
| H27y100 | READ | 14791 | Influenza with gastrointestinal tract involvement |  |
| H27yz00 | READ | 31363 | Influenza with other manifestations NOS |  |
| H27z.00 | READ | 16388 | Influenza NOS |  |
| H27z.11 | READ | 2157 | Flu like illness |  |
| H27z.12 | READ | 5947 | Influenza like illness |  |
| H28..00 | READ | 5324 | Atypical pneumonia |  |
| H29..00 | READ | 94930 | Avian influenza |  |
| H2A..00 |  | 98129 | Influenza due to Influenza A virus subtype H1N1 |  |
| H2A..11 |  | 98102 | Influenza A (H1N1) swine flu |  |
| H2y..00 | READ | 11849 | Other specified pneumonia or influenza |  |
| H2z..00 | READ | 6094 | Pneumonia or influenza NOS |  |

| outcome definition 3: Acute upper respiratory |
| --- |
| n= 90 medcodes used |

| **Read_code** | **readoxmisflag** | **medcode** | **desc** |  |
| --- | --- | --- | --- | --- |
| H0...00 | READ | 8025 | Acute respiratory infections |  |
| H00..00 | READ | 3260 | Acute nasopharyngitis |  |
| H00..11 | READ | 368 | Common cold |  |
| H00..12 | READ | 1246 | Coryza - acute |  |
| H00..13 | READ | 6620 | Febrile cold |  |
| H00..14 | READ | 896 | Nasal catarrh - acute |  |
| H00..15 | READ | 9093 | Pyrexial cold |  |
| H00..16 | READ | 3821 | Rhinitis - acute |  |
| H01..00 | READ | 980 | Acute sinusitis |  |
| H01..11 | READ | 243 | Sinusitis |  |
| H010.00 | READ | 7021 | Acute maxillary sinusitis |  |
| H010.11 | READ | 18555 | Antritis - acute |  |
| H011.00 | READ | 8213 | Acute frontal sinusitis |  |
| H012.00 | READ | 15724 | Acute ethmoidal sinusitis |  |
| H013.00 | READ | 38816 | Acute sphenoidal sinusitis |  |
| H014.00 | READ | 94218 | Acute rhinosinusitis |  |
| H01y.00 | READ | 29696 | Other acute sinusitis |  |
| H01y000 | READ | 19284 | Acute pansinusitis |  |
| H01yz00 | READ | 60733 | Other acute sinusitis NOS |  |
| H01z.00 | READ | 33664 | Acute sinusitis NOS |  |
| H02..00 | READ | 893 | Acute pharyngitis |  |
| H02..11 | READ | 6014 | Sore throat NOS |  |
| H02..12 | READ | 6466 | Viral sore throat NOS |  |
| H02..13 | READ | 310 | Throat infection - pharyngitis |  |
| H020.00 | READ | 36219 | Acute gangrenous pharyngitis |  |
| H021.00 | READ | 24708 | Acute phlegmonous pharyngitis |  |
| H022.00 | READ | 21486 | Acute ulcerative pharyngitis |  |
| H023.00 | READ | 17899 | Acute bacterial pharyngitis |  |
| H023000 | READ | 92428 | Acute pneumococcal pharyngitis |  |
| H023100 | READ | 29589 | Acute staphylococcal pharyngitis |  |
| H023z00 | READ | 53395 | Acute bacterial pharyngitis NOS |  |
| H024.00 | READ | 4868 | Acute viral pharyngitis |  |
| H025.00 | READ | 6274 | Allergic pharyngitis |  |
| H02z.00 | READ | 407 | Acute pharyngitis NOS |  |
| H03..00 | READ | 138 | Acute tonsillitis |  |
| H03..11 | READ | 11499 | Throat infection - tonsillitis |  |
| H03..12 | READ | 2125 | Tonsillitis |  |
| H030.00 | READ | 12010 | Acute erythematous tonsillitis |  |
| H031.00 | READ | 4061 | Acute follicular tonsillitis |  |
| H032.00 | READ | 8452 | Acute ulcerative tonsillitis |  |
| H033.00 | READ | 37409 | Acute catarrhal tonsillitis |  |
| H034.00 | READ | 59986 | Acute gangrenous tonsillitis |  |
| H035.00 | READ | 10156 | Acute bacterial tonsillitis |  |
| H035000 | READ | 58188 | Acute pneumococcal tonsillitis |  |
| H035100 | READ | 64973 | Acute staphylococcal tonsillitis |  |
| H035z00 | READ | 15970 | Acute bacterial tonsillitis NOS |  |
| H036.00 | READ | 9357 | Acute viral tonsillitis |  |
| H037.00 | READ | 1747 | Recurrent acute tonsillitis |  |
| H03z.00 | READ | 20104 | Acute tonsillitis NOS |  |
| H04..00 | READ | 41324 | Acute laryngitis and tracheitis |  |
| H040.00 | READ | 142 | Acute laryngitis |  |
| H040000 | READ | 26038 | Acute oedematous laryngitis |  |
| H040100 | READ | 62885 | Acute ulcerative laryngitis |  |
| H040200 | READ | 10765 | Acute catarrhal laryngitis |  |
| H040300 | READ | 31501 | Acute phlegmonous laryngitis |  |
| H040400 | READ | 43317 | Acute haemophilus influenzae laryngitis |  |
| H040600 | READ | 51562 | Acute suppurative laryngitis |  |
| H040w00 | READ | 5115 | Acute viral laryngitis unspecified |  |
| H040x00 | READ | 52756 | Acute bacterial laryngitis unspecified |  |
| H040z00 | READ | 22720 | Acute laryngitis NOS |  |
| H041.00 | READ | 1257 | Acute tracheitis |  |
| H041000 | READ | 12476 | Acute tracheitis without obstruction |  |
| H041100 | READ | 68867 | Acute tracheitis with obstruction |  |
| H041z00 | READ | 16313 | Acute tracheitis NOS |  |
| H042.00 | READ | 10087 | Acute laryngotracheitis |  |
| H042.11 | READ | 1285 | Laryngotracheitis |  |
| H042000 | READ | 25259 | Acute laryngotracheitis without obstruction |  |
| H042100 | READ | 69898 | Acute laryngotracheitis with obstruction |  |
| H042z00 | READ | 24471 | Acute laryngotracheitis NOS |  |
| H043.00 | READ | 10641 | Acute epiglottitis (non strep) |  |
| H043.11 | READ | 69489 | Viral epiglottitis |  |
| H043000 | READ | 65650 | Acute epiglottitis without obstruction |  |
| H043100 | READ | 48669 | Acute epiglottitis with obstruction |  |
| H043200 | READ | 38128 | Acute obstructive laryngitis |  |
| H043211 | READ | 19431 | Croup |  |
| H043z00 | READ | 892 | Acute epiglottitis NOS |  |
| H044.00 | READ | 1142 | Croup |  |
| H04z.00 | READ | 16120 | Acute laryngitis and tracheitis NOS |  |
| H05..00 | READ | 26010 | Other acute upper respiratory infections |  |
| H050.00 | READ | 18908 | Acute laryngopharyngitis |  |
| H051.00 | READ | 6294 | Acute upper respiratory tract infection |  |
| H052.00 | READ | 21415 | Pharyngotracheitis |  |
| H053.00 | READ | 10093 | Tracheopharyngitis |  |
| H054.00 | READ | 4221 | Recurrent upper respiratory tract infection |  |
| H055.00 | READ | 4718 | Pharyngolaryngitis |  |
| H05y.00 | READ | 15628 | Other upper respiratory infections of multiple sites |  |
| H05z.00 | READ | 76 | Upper respiratory infection NOS |  |
| H05z.11 | READ | 2637 | Upper respiratory tract infection NOS |  |
| H05z.12 | READ | 6421 | Viral upper respiratory tract infection NOS |  |
| H130.12 | READ | 3624 | Maxillary sinusitis |  |

| outcome definition 4: Bronchitis Bronchiolitis |
| --- |
| n= 40 medcodes used |

| **Read_code** | **readoxmisflag** | **medcode** | **desc** |  |
| --- | --- | --- | --- | --- |
| H06..00 | READ | 29669 | Acute bronchitis and bronchiolitis |  |
| H060.00 | READ | 312 | Acute bronchitis |  |
| H060.11 | READ | 5978 | Acute wheezy bronchitis |  |
| H060000 | READ | 50396 | Acute fibrinous bronchitis |  |
| H060100 | READ | 101775 | Acute membranous bronchitis |  |
| H060200 | READ | 71370 | Acute pseudomembranous bronchitis |  |
| H060300 | READ | 11072 | Acute purulent bronchitis |  |
| H060400 | READ | 21145 | Acute croupous bronchitis |  |
| H060500 | READ | 11101 | Acute tracheobronchitis |  |
| H060600 | READ | 9043 | Acute pneumococcal bronchitis |  |
| H060700 | READ | 43362 | Acute streptococcal bronchitis |  |
| H060800 | READ | 21492 | Acute haemophilus influenzae bronchitis |  |
| H060900 | READ | 49794 | Acute neisseria catarrhalis bronchitis |  |
| H060A00 | READ | 31886 | Acute bronchitis due to mycoplasma pneumoniae |  |
| H060B00 | READ | 93153 | Acute bronchitis due to coxsackievirus |  |
| H060C00 | READ | 29273 | Acute bronchitis due to parainfluenza virus |  |
| H060D00 | READ | 48593 | Acute bronchitis due to respiratory syncytial virus |  |
| H060E00 | READ | 64890 | Acute bronchitis due to rhinovirus |  |
| H060F00 | READ | 65916 | Acute bronchitis due to echovirus |  |
| H060v00 | READ | 55391 | Subacute bronchitis unspecified |  |
| H060w00 | READ | 1382 | Acute viral bronchitis unspecified |  |
| H060x00 | READ | 24800 | Acute bacterial bronchitis unspecified |  |
| H060z00 | READ | 20198 | Acute bronchitis NOS |  |
| H061.00 | READ | 1019 | Acute bronchiolitis |  |
| H061000 | READ | 54533 | Acute capillary bronchiolitis |  |
| H061100 | READ | 41589 | Acute obliterating bronchiolitis |  |
| H061200 | READ | 17185 | Acute bronchiolitis with bronchospasm |  |
| H061300 | READ | 69192 | Acute exudative bronchiolitis |  |
| H061400 | READ | 6181 | Obliterating fibrous bronchiolitis |  |
| H061500 | READ | 18451 | Acute bronchiolitis due to respiratory syncytial virus |  |
| H061600 | READ | 66228 | Acute bronchiolitis due to other specified organisms |  |
| H061z00 | READ | 17917 | Acute bronchiolitis NOS |  |
| H062.00 | READ | 6124 | Acute lower respiratory tract infection |  |
| H06z.00 | READ | 41137 | Acute bronchitis or bronchiolitis NOS |  |
| H06z000 | READ | 2581 | Chest infection NOS |  |
| H06z011 | READ | 68 | Chest infection |  |
| H06z100 | READ | 3358 | Lower resp tract infection |  |
| H06z111 | READ | 293 | Respiratory tract infection |  |
| H06z112 | READ | 37447 | Acute lower respiratory tract infection |  |
| H06z200 | READ | 4899 | Recurrent chest infection |  |

| outcome definition 5: Chronic respiratory including COPD_asthma |
| --- |
| n= 86 medcodes used |

| **Read_code** | **readoxmisflag** | **medcode** | **desc** |  |
| --- | --- | --- | --- | --- |
| H3...00 | READ | 1001 | Chronic obstructive pulmonary disease |  |
| H3...11 | READ | 998 | Chronic obstructive airways disease |  |
| H31..00 | READ | 3243 | Chronic bronchitis |  |
| H310.00 | READ | 25603 | Simple chronic bronchitis |  |
| H310000 | READ | 15626 | Chronic catarrhal bronchitis |  |
| H310100 | READ | 16717 | Smokers' cough |  |
| H310z00 | READ | 61118 | Simple chronic bronchitis NOS |  |
| H311.00 | READ | 11150 | Mucopurulent chronic bronchitis |  |
| H311000 | READ | 40159 | Purulent chronic bronchitis |  |
| H311100 | READ | 37959 | Fetid chronic bronchitis |  |
| H311z00 | READ | 61513 | Mucopurulent chronic bronchitis NOS |  |
| H312.00 | READ | 27819 | Obstructive chronic bronchitis |  |
| H312000 | READ | 5798 | Chronic asthmatic bronchitis |  |
| H312011 | READ | 5909 | Chronic wheezy bronchitis |  |
| H312100 | READ | 14798 | Emphysematous bronchitis |  |
| H312200 | READ | 1446 | Acute exacerbation of chronic obstructive airways disease |  |
| H312300 | READ | 26125 | Bronchiolitis obliterans |  |
| H312z00 | READ | 44525 | Obstructive chronic bronchitis NOS |  |
| H313.00 | READ | 24248 | Mixed simple and mucopurulent chronic bronchitis |  |
| H31y.00 | READ | 66043 | Other chronic bronchitis |  |
| H31y000 | READ | 23618 | Chronic tracheitis |  |
| H31y100 | READ | 45089 | Chronic tracheobronchitis |  |
| H31yz00 | READ | 68066 | Other chronic bronchitis NOS |  |
| H31z.00 | READ | 15157 | Chronic bronchitis NOS |  |
| H32..00 | READ | 794 | Emphysema |  |
| H320.00 | READ | 26306 | Chronic bullous emphysema |  |
| H320000 | READ | 56860 | Segmental bullous emphysema |  |
| H320100 | READ | 68662 | Zonal bullous emphysema |  |
| H320200 | READ | 60188 | Giant bullous emphysema |  |
| H320300 | READ | 99536 | Bullous emphysema with collapse |  |
| H320z00 | READ | 23492 | Chronic bullous emphysema NOS |  |
| H321.00 | READ | 46578 | Panlobular emphysema |  |
| H322.00 | READ | 10980 | Centrilobular emphysema |  |
| H32y.00 | READ | 40788 | Other emphysema |  |
| H32y000 | READ | 92955 | Acute vesicular emphysema |  |
| H32y100 | READ | 70787 | Atrophic (senile) emphysema |  |
| H32y111 | READ | 59263 | Acute interstitial emphysema |  |
| H32y200 | READ | 63479 | MacLeod's unilateral emphysema |  |
| H32yz00 | READ | 16410 | Other emphysema NOS |  |
| H32z.00 | READ | 33450 | Emphysema NOS |  |
| H33..00 | READ | 78 | Asthma |  |
| H33..11 | READ | 1555 | Bronchial asthma |  |
| H330.00 | READ | 7146 | Extrinsic (atopic) asthma |  |
| H330.11 | READ | 2290 | Allergic asthma |  |
| H330.12 | READ | 1208 | Childhood asthma |  |
| H330.13 | READ | 15248 | Hay fever with asthma |  |
| H330.14 | READ | 7731 | Pollen asthma |  |
| H330000 | READ | 14777 | Extrinsic asthma without status asthmaticus |  |
| H330011 | READ | 5627 | Hay fever with asthma |  |
| H330100 | READ | 27926 | Extrinsic asthma with status asthmaticus |  |
| H330111 | READ | 6707 | Extrinsic asthma with asthma attack |  |
| H330z00 | READ | 45782 | Extrinsic asthma NOS |  |
| H331.00 | READ | 5267 | Intrinsic asthma |  |
| H331.11 | READ | 3665 | Late onset asthma |  |
| H331000 | READ | 29325 | Intrinsic asthma without status asthmaticus |  |
| H331100 | READ | 58196 | Intrinsic asthma with status asthmaticus |  |
| H331111 | READ | 18323 | Intrinsic asthma with asthma attack |  |
| H331z00 | READ | 45073 | Intrinsic asthma NOS |  |
| H332.00 | READ | 25796 | Mixed asthma |  |
| H333.00 | READ | 185 | Acute exacerbation of asthma |  |
| H334.00 | READ | 40823 | Brittle asthma |  |
| H33z.00 | READ | 4442 | Asthma unspecified |  |
| H33z.11 | READ | 32727 | Hyperreactive airways disease |  |
| H33z000 | READ | 4892 | Status asthmaticus NOS |  |
| H33z011 | READ | 233 | Severe asthma attack |  |
| H33z100 | READ | 232 | Asthma attack |  |
| H33z111 | READ | 8335 | Asthma attack NOS |  |
| H33z200 | READ | 12987 | Late-onset asthma |  |
| H33zz00 | READ | 16070 | Asthma NOS |  |
| H33zz11 | READ | 4606 | Exercise induced asthma |  |
| H33zz12 | READ | 21232 | Allergic asthma NEC |  |
| H33zz13 | READ | 18207 | Allergic bronchitis NEC |  |
| H34..00 | READ | 2195 | Bronchiectasis |  |
| H340.00 | READ | 20364 | Recurrent bronchiectasis |  |
| H341.00 | READ | 41491 | Post-infective bronchiectasis |  |
| H34z.00 | READ | 32679 | Bronchiectasis NOS |  |
| H36..00 | READ | 10863 | Mild chronic obstructive pulmonary disease |  |
| H37..00 | READ | 10802 | Moderate chronic obstructive pulmonary disease |  |
| H38..00 | READ | 9876 | Severe chronic obstructive pulmonary disease |  |
| H39..00 | READ | 93568 | Very severe chronic obstructive pulmonary disease |  |
| H3y..00 | READ | 12166 | Other specified chronic obstructive airways disease |  |
| H3y..11 | READ | 67040 | Other specified chronic obstructive pulmonary disease |  |
| H3y0.00 | READ | 21061 | Chronic obstruct pulmonary dis with acute lower resp infectn |  |
| H3y1.00 | READ | 7884 | Chron obstruct pulmonary dis wth acute exacerbation, unspec |  |
| H3z..00 | READ | 5710 | Chronic obstructive airways disease NOS |  |
| H3z..11 | READ | 37247 | Chronic obstructive pulmonary disease NOS |  |

| outcome definition 6: Urinary_Tract_Infection |
| --- |
| n= 2 medcodes used |

| **Read_code** | **readoxmisflag** | **medcode** | **desc** |  |
| --- | --- | --- | --- | --- |
| K190.00 | READ | 1289 | Urinary tract infection, site not specified |  |
| K190z00 | READ | 150 | Urinary tract infection, site not specified NOS |  |

## Coding Definitions Treatment

| risk_factor definition 1: Immuno_suppressant Rx |
| --- |
| n= 333 prodcodes used |

| **prodcode** | **drugsubstance** | **bnfchapter** | **productname** |  |
| --- | --- | --- | --- | --- |
| 25904 | arsenic trioxide | Arsenic trioxide | arsenic trioxide concentrate for solution for infusion 10mg/10ml |  |
| 13493 | auranofin | Gold salts | RIDAURA TILTAB tablets 3mg [ASTELLAS] |  |
| 3934 | auranofin | Gold salts | auranofin tablets 3mg |  |
| 14395 | azathioprine | Antiproliferative immunosuppressants/Immunosuppressants (in chronic bowel disorders)/Drugs affecting the immune response (in rheumatic disease) | IMURAN injection 50mg/vial [ASPEN EURO] |  |
| 270 | azathioprine | Antiproliferative immunosuppressants/Immunosuppressants (in chronic bowel disorders)/Drugs affecting the immune response (in rheumatic disease) | azathioprine injection 50mg/vial |  |
| 12339 | azathioprine | Antiproliferative immunosuppressants/Immunosuppressants (in chronic bowel disorders)/Drugs affecting the immune response (in rheumatic disease)/Drugs affecting the immune response (for skin conditions) | AZAMUNE tablets 50mg [PENN] |  |
| 34816 | azathioprine | Antiproliferative immunosuppressants/Immunosuppressants (in chronic bowel disorders)/Drugs affecting the immune response (in rheumatic disease)/Drugs affecting the immune response (for skin conditions) | AZATHIOPRINE tablets 25mg [GEN (UK)] |  |
| 32101 | azathioprine | Antiproliferative immunosuppressants/Immunosuppressants (in chronic bowel disorders)/Drugs affecting the immune response (in rheumatic disease)/Drugs affecting the immune response (for skin conditions) | AZATHIOPRINE tablets 25mg [HILLCROSS] |  |
| 43562 | azathioprine | Antiproliferative immunosuppressants/Immunosuppressants (in chronic bowel disorders)/Drugs affecting the immune response (in rheumatic disease)/Drugs affecting the immune response (for skin conditions) | AZATHIOPRINE tablets 50mg [ACTAVIS] |  |
| 41670 | azathioprine | Antiproliferative immunosuppressants/Immunosuppressants (in chronic bowel disorders)/Drugs affecting the immune response (in rheumatic disease)/Drugs affecting the immune response (for skin conditions) | AZATHIOPRINE tablets 50mg [CP PHARM] |  |
| 34451 | azathioprine | Antiproliferative immunosuppressants/Immunosuppressants (in chronic bowel disorders)/Drugs affecting the immune response (in rheumatic disease)/Drugs affecting the immune response (for skin conditions) | AZATHIOPRINE tablets 50mg [GEN (UK)] |  |
| 34687 | azathioprine | Antiproliferative immunosuppressants/Immunosuppressants (in chronic bowel disorders)/Drugs affecting the immune response (in rheumatic disease)/Drugs affecting the immune response (for skin conditions) | AZATHIOPRINE tablets 50mg [HILLCROSS] |  |
| 29340 | azathioprine | Antiproliferative immunosuppressants/Immunosuppressants (in chronic bowel disorders)/Drugs affecting the immune response (in rheumatic disease)/Drugs affecting the immune response (for skin conditions) | AZATHIOPRINE tablets 50mg [IVAX] |  |
| 31215 | azathioprine | Antiproliferative immunosuppressants/Immunosuppressants (in chronic bowel disorders)/Drugs affecting the immune response (in rheumatic disease)/Drugs affecting the immune response (for skin conditions) | AZATHIOPRINE tablets 50mg [KENT] |  |
| 41620 | azathioprine | Antiproliferative immunosuppressants/Immunosuppressants (in chronic bowel disorders)/Drugs affecting the immune response (in rheumatic disease)/Drugs affecting the immune response (for skin conditions) | AZATHIOPRINE tablets 50mg [TEVA] |  |
| 26261 | azathioprine | Antiproliferative immunosuppressants/Immunosuppressants (in chronic bowel disorders)/Drugs affecting the immune response (in rheumatic disease)/Drugs affecting the immune response (for skin conditions) | BERKAPRINE tablets 50mg [RORER] |  |
| 21899 | azathioprine | Antiproliferative immunosuppressants/Immunosuppressants (in chronic bowel disorders)/Drugs affecting the immune response (in rheumatic disease)/Drugs affecting the immune response (for skin conditions) | IMMUNOPRIN tablets 50mg [ASHBOURNE] |  |
| 30495 | azathioprine | Antiproliferative immunosuppressants/Immunosuppressants (in chronic bowel disorders)/Drugs affecting the immune response (in rheumatic disease)/Drugs affecting the immune response (for skin conditions) | IMURAN tablets 10mg [WELLCOME] |  |
| 43077 | azathioprine | Antiproliferative immunosuppressants/Immunosuppressants (in chronic bowel disorders)/Drugs affecting the immune response (in rheumatic disease)/Drugs affecting the immune response (for skin conditions) | IMURAN tablets 25mg [ASPEN EURO] |  |
| 671 | azathioprine | Antiproliferative immunosuppressants/Immunosuppressants (in chronic bowel disorders)/Drugs affecting the immune response (in rheumatic disease)/Drugs affecting the immune response (for skin conditions) | IMURAN tablets 25mg [WELLCOME] |  |
| 42988 | azathioprine | Antiproliferative immunosuppressants/Immunosuppressants (in chronic bowel disorders)/Drugs affecting the immune response (in rheumatic disease)/Drugs affecting the immune response (for skin conditions) | IMURAN tablets 50mg [ASPEN EURO] |  |
| 1899 | azathioprine | Antiproliferative immunosuppressants/Immunosuppressants (in chronic bowel disorders)/Drugs affecting the immune response (in rheumatic disease)/Drugs affecting the immune response (for skin conditions) | IMURAN tablets 50mg [WELLCOME] |  |
| 19072 | azathioprine | Antiproliferative immunosuppressants/Immunosuppressants (in chronic bowel disorders)/Drugs affecting the immune response (in rheumatic disease)/Drugs affecting the immune response (for skin conditions) | OPRISINE tablets 50mg [OPUS] |  |
| 770 | azathioprine | Antiproliferative immunosuppressants/Immunosuppressants (in chronic bowel disorders)/Drugs affecting the immune response (in rheumatic disease)/Drugs affecting the immune response (for skin conditions) | azathioprine capsules |  |
| 39115 | azathioprine | Antiproliferative immunosuppressants/Immunosuppressants (in chronic bowel disorders)/Drugs affecting the immune response (in rheumatic disease)/Drugs affecting the immune response (for skin conditions) | azathioprine capsules 10mg |  |
| 22982 | azathioprine | Antiproliferative immunosuppressants/Immunosuppressants (in chronic bowel disorders)/Drugs affecting the immune response (in rheumatic disease)/Drugs affecting the immune response (for skin conditions) | azathioprine oral solution 50mg/5ml |  |
| 36792 | azathioprine | Antiproliferative immunosuppressants/Immunosuppressants (in chronic bowel disorders)/Drugs affecting the immune response (in rheumatic disease)/Drugs affecting the immune response (for skin conditions) | azathioprine oral solution 50mg/ml |  |
| 35518 | azathioprine | Antiproliferative immunosuppressants/Immunosuppressants (in chronic bowel disorders)/Drugs affecting the immune response (in rheumatic disease)/Drugs affecting the immune response (for skin conditions) | azathioprine oral suspension 50mg/5ml |  |
| 13320 | azathioprine | Antiproliferative immunosuppressants/Immunosuppressants (in chronic bowel disorders)/Drugs affecting the immune response (in rheumatic disease)/Drugs affecting the immune response (for skin conditions) | azathioprine tablets 10mg |  |
| 451 | azathioprine | Antiproliferative immunosuppressants/Immunosuppressants (in chronic bowel disorders)/Drugs affecting the immune response (in rheumatic disease)/Drugs affecting the immune response (for skin conditions) | azathioprine tablets 25mg |  |
| 571 | azathioprine | Antiproliferative immunosuppressants/Immunosuppressants (in chronic bowel disorders)/Drugs affecting the immune response (in rheumatic disease)/Drugs affecting the immune response (for skin conditions) | azathioprine tablets 50mg |  |
| 25740 | bevacizumab | Bevacizumab | AVASTIN concentrate for solution for infusion 100mg/4ml [ROCHE] |  |
| 38145 | bevacizumab | Bevacizumab | bevacizumab concentrate for solution for infusion 100mg/4ml |  |
| 40379 | bexarotene | Bexarotene | TARGRETIN capsules 75mg [CEPHALON] |  |
| 36769 | bexarotene | Bexarotene | bexarotene capsules 75mg |  |
| 25851 | bleomycin sulphate | Anthracyclines and other cytotoxic antibiotics | BLEO-KYOWA injection 15000 iu/vial [KYOWA HAK] |  |
| 32261 | bleomycin sulphate | Anthracyclines and other cytotoxic antibiotics | bleomycin sulphate injection 15000 iu/vial |  |
| 40732 | bortezomib | Bortezomib | VELCADE powder for solution for injection 3.5mg [ORTHO BIO] |  |
| 44740 | bortezomib | Bortezomib | bortezomib powder for solution for injection 3.5mg |  |
| 26301 | busulfan | Alkylating agents | MYLERAN tablets 2mg [WELLCOME] |  |
| 32412 | busulfan | Alkylating agents | MYLERAN tablets 500micrograms [WELLCOME] |  |
| 3874 | busulfan | Alkylating agents | busulfan tablets 2mg |  |
| 22204 | busulfan | Alkylating agents | busulfan tablets 500micrograms |  |
| 26320 | calcium folinate | Chemotherapy-induced mucositis and myelosuppression | CALCIUM LEUCOVORIN injection 15mg/vial [WYETH PHAR] |  |
| 33261 | calcium folinate | Chemotherapy-induced mucositis and myelosuppression | REFOLINON injection 3mg/ml [PHARMACIA] |  |
| 22811 | calcium folinate | Chemotherapy-induced mucositis and myelosuppression | calcium folinate injection (powder) 15mg/vial |  |
| 26006 | calcium folinate | Chemotherapy-induced mucositis and myelosuppression | calcium folinate injection (solution) 10mg/ml |  |
| 38371 | calcium folinate | Chemotherapy-induced mucositis and myelosuppression | calcium folinate injection (solution) 15mg/2ml |  |
| 39468 | calcium folinate | Chemotherapy-induced mucositis and myelosuppression | calcium folinate injection (solution) 30mg/10ml |  |
| 39201 | calcium folinate | Chemotherapy-induced mucositis and myelosuppression | calcium folinate injection (solution) 3mg/1ml |  |
| 39574 | calcium folinate | Chemotherapy-induced mucositis and myelosuppression | calcium leucovorin injection (solution) 15mg/2ml |  |
| 26702 | calcium folinate | Chemotherapy-induced mucositis and myelosuppression | folinic acid injection 350mg/35ml |  |
| 34599 | calcium folinate | Chemotherapy-induced mucositis and myelosuppression/Folic acid and derivatives | CALCIUM FOLINATE tablets 15mg [BMS] |  |
| 23949 | calcium folinate | Chemotherapy-induced mucositis and myelosuppression/Folic acid and derivatives | CALCIUM LEUCOVORIN tablets 15mg [WYETH PHAR] |  |
| 28208 | calcium folinate | Chemotherapy-induced mucositis and myelosuppression/Folic acid and derivatives | REFOLINON tablets 15mg [PHARMACIA] |  |
| 2169 | calcium folinate | Chemotherapy-induced mucositis and myelosuppression/Folic acid and derivatives | calcium folinate tablets 15mg |  |
| 31524 | calcium folinate | Chemotherapy-induced mucositis and myelosuppression/Folic acid and derivatives | calcium leucovorin tablets 15mg |  |
| 33127 | capecitabine | Antimetabolites | XELODA tablets 150mg [ROCHE] |  |
| 18063 | capecitabine | Antimetabolites | XELODA tablets 500mg [ROCHE] |  |
| 7340 | capecitabine | Antimetabolites | capecitabine tablets 150mg |  |
| 7341 | capecitabine | Antimetabolites | capecitabine tablets 500mg |  |
| 41963 | carboplatin | Platinum Compounds | PARAPLATIN concentrate for solution for infusion 10mg/ml [BRISTOL] |  |
| 10328 | carboplatin | Platinum Compounds | carboplatin concentrate for solution for infusion 10mg/ml |  |
| 40781 | carboplatin | Platinum Compounds | carboplatin concentrate for solution for infusion 150mg/15ml |  |
| 35855 | carboplatin | Platinum Compounds | carboplatin concentrate for solution for infusion 50mg/5ml |  |
| 32712 | cetuximab | Cetuximab | ERBITUX solution for infusion 2mg/ml [MERCK PHAR] |  |
| 16838 | chlorambucil | Alkylating agents | LEUKERAN tablets 2mg [WELLCOME] |  |
| 26315 | chlorambucil | Alkylating agents | LEUKERAN tablets 5mg [WELLCOME] |  |
| 5600 | chlorambucil | Alkylating agents | chlorambucil tablets 2mg |  |
| 8665 | chlorambucil | Alkylating agents | chlorambucil tablets 5mg |  |
| 26119 | chlormethine hydrochloride | Alkylating agents | CHLORMETHINE injection 10mg/ml [SOVEREIGN] |  |
| 28709 | chlormethine hydrochloride | Alkylating agents | chlormethine injection 10mg |  |
| 42449 | ciclosporin | Corticosteroids and other immunosuppressants/Immunosuppressants (in chronic bowel disorders)/Drugs affecting the immune response (in rheumatic disease)/Drugs affecting the immune response (for skin conditions) | DEXIMUNE capsules 100mg [DEXCEL] |  |
| 42637 | ciclosporin | Corticosteroids and other immunosuppressants/Immunosuppressants (in chronic bowel disorders)/Drugs affecting the immune response (in rheumatic disease)/Drugs affecting the immune response (for skin conditions) | DEXIMUNE capsules 25mg [DEXCEL] |  |
| 42448 | ciclosporin | Corticosteroids and other immunosuppressants/Immunosuppressants (in chronic bowel disorders)/Drugs affecting the immune response (in rheumatic disease)/Drugs affecting the immune response (for skin conditions) | DEXIMUNE capsules 50mg [DEXCEL] |  |
| 973 | ciclosporin | Corticosteroids and other immunosuppressants/Immunosuppressants (in chronic bowel disorders)/Drugs affecting the immune response (in rheumatic disease)/Drugs affecting the immune response (for skin conditions) | NEORAL capsules 100mg [NOVARTIS] |  |
| 16137 | ciclosporin | Corticosteroids and other immunosuppressants/Immunosuppressants (in chronic bowel disorders)/Drugs affecting the immune response (in rheumatic disease)/Drugs affecting the immune response (for skin conditions) | NEORAL capsules 10mg [NOVARTIS] |  |
| 972 | ciclosporin | Corticosteroids and other immunosuppressants/Immunosuppressants (in chronic bowel disorders)/Drugs affecting the immune response (in rheumatic disease)/Drugs affecting the immune response (for skin conditions) | NEORAL capsules 25mg [NOVARTIS] |  |
| 4231 | ciclosporin | Corticosteroids and other immunosuppressants/Immunosuppressants (in chronic bowel disorders)/Drugs affecting the immune response (in rheumatic disease)/Drugs affecting the immune response (for skin conditions) | NEORAL capsules 50mg [NOVARTIS] |  |
| 1905 | ciclosporin | Corticosteroids and other immunosuppressants/Immunosuppressants (in chronic bowel disorders)/Drugs affecting the immune response (in rheumatic disease)/Drugs affecting the immune response (for skin conditions) | NEORAL oral solution 100mg/ml [NOVARTIS] |  |
| 13556 | ciclosporin | Corticosteroids and other immunosuppressants/Immunosuppressants (in chronic bowel disorders)/Drugs affecting the immune response (in rheumatic disease)/Drugs affecting the immune response (for skin conditions) | SANDIMMUN capsules 100mg [NOVARTIS] |  |
| 3920 | ciclosporin | Corticosteroids and other immunosuppressants/Immunosuppressants (in chronic bowel disorders)/Drugs affecting the immune response (in rheumatic disease)/Drugs affecting the immune response (for skin conditions) | SANDIMMUN capsules 25mg [NOVARTIS] |  |
| 15596 | ciclosporin | Corticosteroids and other immunosuppressants/Immunosuppressants (in chronic bowel disorders)/Drugs affecting the immune response (in rheumatic disease)/Drugs affecting the immune response (for skin conditions) | SANDIMMUN capsules 50mg [NOVARTIS] |  |
| 26790 | ciclosporin | Corticosteroids and other immunosuppressants/Immunosuppressants (in chronic bowel disorders)/Drugs affecting the immune response (in rheumatic disease)/Drugs affecting the immune response (for skin conditions) | SANDIMMUN concentrate for solution for infusion 50mg/ml [NOVARTIS] |  |
| 13494 | ciclosporin | Corticosteroids and other immunosuppressants/Immunosuppressants (in chronic bowel disorders)/Drugs affecting the immune response (in rheumatic disease)/Drugs affecting the immune response (for skin conditions) | SANDIMMUN sugar free solution 100mg/ml [NOVARTIS] |  |
| 3896 | ciclosporin | Corticosteroids and other immunosuppressants/Immunosuppressants (in chronic bowel disorders)/Drugs affecting the immune response (in rheumatic disease)/Drugs affecting the immune response (for skin conditions) | ciclosporin capsules 100mg |  |
| 16035 | ciclosporin | Corticosteroids and other immunosuppressants/Immunosuppressants (in chronic bowel disorders)/Drugs affecting the immune response (in rheumatic disease)/Drugs affecting the immune response (for skin conditions) | ciclosporin capsules 10mg |  |
| 2838 | ciclosporin | Corticosteroids and other immunosuppressants/Immunosuppressants (in chronic bowel disorders)/Drugs affecting the immune response (in rheumatic disease)/Drugs affecting the immune response (for skin conditions) | ciclosporin capsules 25mg |  |
| 2837 | ciclosporin | Corticosteroids and other immunosuppressants/Immunosuppressants (in chronic bowel disorders)/Drugs affecting the immune response (in rheumatic disease)/Drugs affecting the immune response (for skin conditions) | ciclosporin capsules 50mg |  |
| 42924 | ciclosporin | Corticosteroids and other immunosuppressants/Immunosuppressants (in chronic bowel disorders)/Drugs affecting the immune response (in rheumatic disease)/Drugs affecting the immune response (for skin conditions) | ciclosporin concentrate for solution for infusion 250mg/5ml |  |
| 38056 | ciclosporin | Corticosteroids and other immunosuppressants/Immunosuppressants (in chronic bowel disorders)/Drugs affecting the immune response (in rheumatic disease)/Drugs affecting the immune response (for skin conditions) | ciclosporin concentrate for solution for infusion 50mg/1ml |  |
| 19370 | ciclosporin | Corticosteroids and other immunosuppressants/Immunosuppressants (in chronic bowel disorders)/Drugs affecting the immune response (in rheumatic disease)/Drugs affecting the immune response (for skin conditions) | ciclosporin concentrate for solution for infusion 50mg/ml |  |
| 1626 | ciclosporin | Corticosteroids and other immunosuppressants/Immunosuppressants (in chronic bowel disorders)/Drugs affecting the immune response (in rheumatic disease)/Drugs affecting the immune response (for skin conditions) | ciclosporin oral solution 100mg/ml |  |
| 38453 | cisplatin | Platinum Compounds | cisplatin concentrate for solution for infusion 10mg/10ml |  |
| 44388 | cisplatin | Platinum Compounds | cisplatin concentrate for solution for infusion 50mg/50ml |  |
| 28324 | cisplatin | Platinum Compounds | cisplatin powder 25mg/vial |  |
| 40454 | cladribine | Antimetabolites | cladribine injection 10mg/5ml |  |
| 34728 | cyclophosphamide | Alkylating agents | CYCLOPHOSPHAMIDE tablets 50mg [PHARMACIA] |  |
| 44309 | cyclophosphamide | Alkylating agents | ENDOXANA injection 1000mg [BAXTER ONC] |  |
| 44273 | cyclophosphamide | Alkylating agents | ENDOXANA injection 200mg [BAXTER ONC] |  |
| 31193 | cyclophosphamide | Alkylating agents | ENDOXANA tablets 10mg [BAXTER ONC] |  |
| 10729 | cyclophosphamide | Alkylating agents | ENDOXANA tablets 50mg [BAXTER ONC] |  |
| 29840 | cyclophosphamide | Alkylating agents | cyclophosphamide injection 1000mg |  |
| 26322 | cyclophosphamide | Alkylating agents | cyclophosphamide injection 100mg |  |
| 26066 | cyclophosphamide | Alkylating agents | cyclophosphamide injection 200mg |  |
| 16105 | cyclophosphamide | Alkylating agents | cyclophosphamide injection 500mg |  |
| 3984 | cyclophosphamide | Alkylating agents | cyclophosphamide tablets 10mg |  |
| 3985 | cyclophosphamide | Alkylating agents | cyclophosphamide tablets 50mg |  |
| 19335 | cytarabine | Antimetabolites | CYTOSAR injection 500mg [PHARMACIA] |  |
| 41266 | cytarabine | Antimetabolites | cytarabine injection solution 1g/10ml |  |
| 18238 | dacarbazine citrate | Dacarbazine and temozolomide | dacarbazine powder for solution for injection 100mg |  |
| 27071 | dactinomycin | Anthracyclines and other cytotoxic antibiotics | dactinomycin powder for solution for injection 500micrograms |  |
| 42390 | dasatinib | Dasatinib | dasatinib tablets 100mg |  |
| 37238 | dasatinib | Dasatinib | dasatinib tablets 20mg |  |
| 36062 | dasatinib | Dasatinib | dasatinib tablets 50mg |  |
| 36957 | dasatinib | Dasatinib | dasatinib tablets 70mg |  |
| 11003 | daunorubicin | Anthracyclines and other cytotoxic antibiotics | CERUBIDIN powder for concentrate for solution for injection 20mg/vial [RHONE] |  |
| 43805 | daunorubicin | Anthracyclines and other cytotoxic antibiotics | daunorubicin powder for concentrate for solution for injection 20mg/vial |  |
| 36052 | disodium folinate | Chemotherapy-induced mucositis and myelosuppression | SODIOFOLIN injection 100mg/2ml [MEDAC UK] |  |
| 23849 | docetaxel | Taxanes | TAXOTERE concentrate for intravenous infusion 40mg/ml [AVENTIS] |  |
| 36552 | docetaxel | Taxanes | TAXOTERE concentrate for solution for infusion 20mg/0.5ml [AVENTIS] |  |
| 44087 | docetaxel | Taxanes | TAXOTERE concentrate for solution for infusion 20mg/1ml [AVENTIS] |  |
| 36831 | docetaxel | Taxanes | docetaxel concentrate for dilution for infusion solution 20mg/0.5ml |  |
| 38999 | docetaxel | Taxanes | docetaxel concentrate for dilution for infusion solution 80mg/2ml |  |
| 33560 | docetaxel | Taxanes | docetaxel concentrate for intravenous infusion 40mg/ml |  |
| 37784 | doxorubicin hydrochloride | Anthracyclines and other cytotoxic antibiotics | CAELYX concentrate for solution for infusion 20mg/10ml [JANSSEN] |  |
| 40250 | doxorubicin hydrochloride | Anthracyclines and other cytotoxic antibiotics | CAELYX concentrate for solution for infusion 50mg/25ml [JANSSEN] |  |
| 33878 | doxorubicin hydrochloride | Anthracyclines and other cytotoxic antibiotics | MYOCET powder for concentrate for solution for infusion 50mg [CEPHALON] |  |
| 25262 | erlotinib hydrochloride | Erlotinib | TARCEVA film coated tablets 150mg [ROCHE] |  |
| 37441 | erlotinib hydrochloride | Erlotinib | TARCEVA film coated tablets 25mg [ROCHE] |  |
| 40094 | erlotinib hydrochloride | Erlotinib | erlotinib tablets 100mg |  |
| 33827 | erlotinib hydrochloride | Erlotinib | erlotinib tablets 150mg |  |
| 13604 | estramustine sodium phosphate | Alkylating agents | ESTRACYT capsules 140mg [PHARMACIA] |  |
| 13735 | estramustine sodium phosphate | Alkylating agents | estramustine phosphate capsules 140mg |  |
| 36263 | etoposide | Vinca alkaloids and etoposide | EPOSIN concentrate for solution for infusion 20mg/ml [MEDAC UK] |  |
| 37375 | etoposide | Vinca alkaloids and etoposide | VEPESID capsules 100mg [BRISTOL] |  |
| 29761 | etoposide | Vinca alkaloids and etoposide | VEPESID capsules 50mg [BRISTOL] |  |
| 18751 | etoposide | Vinca alkaloids and etoposide | etoposide capsules 100mg |  |
| 8756 | etoposide | Vinca alkaloids and etoposide | etoposide capsules 50mg |  |
| 31115 | etoposide | Vinca alkaloids and etoposide | etoposide concentrate for solution for infusion 20mg/ml |  |
| 44387 | etoposide phosphate | Vinca alkaloids and etoposide | etoposide phosphate lyophilised powder for injection 100mg |  |
| 43781 | everolimus | Everolimus | AFINITOR tablets 10mg [NOVARTIS] |  |
| 29743 | fludarabine phosphate | Antimetabolites | FLUDARA ORAL tablets 10mg [GENZYME] |  |
| 24681 | fludarabine phosphate | Antimetabolites | fludarabine powder for solution for injection 50mg |  |
| 18476 | fludarabine phosphate | Antimetabolites | fludarabine tablets 10mg |  |
| 19556 | fluorouracil | Antimetabolites | FLUORO-URACIL capsules 250mg [CAMBRIDGE] |  |
| 20229 | fluorouracil | Antimetabolites | FLUORO-URACIL injection 25mg/ml [CAMBRIDGE] |  |
| 18070 | fluorouracil | Antimetabolites | fluorouracil capsules 250mg |  |
| 39388 | fluorouracil | Antimetabolites | fluorouracil injection 1g/20ml |  |
| 36575 | fluorouracil | Antimetabolites | fluorouracil injection 50mg/ml |  |
| 41077 | gefitinib | Gefitinib | gefitinib tablets 250mg |  |
| 40780 | gemcitabine hydrochloride | Antimetabolites | gemcitabine powder for solution for infusion 1g/vial |  |
| 33418 | gemcitabine hydrochloride | Antimetabolites | gemcitabine powder for solution for infusion 200mg/vial |  |
| 6884 | hydroxycarbamide | Hydroxycarbamide | HYDREA capsules 500mg [SQUIBB] |  |
| 33330 | hydroxycarbamide | Hydroxycarbamide | HYDROXYCARBAMIDE capsules 500mg [MEDAC UK] |  |
| 6333 | hydroxycarbamide | Hydroxycarbamide | hydroxycarbamide capsules 500mg |  |
| 39548 | hydroxycarbamide | Hydroxycarbamide | hydroxycarbamide film coated tablets 1000mg |  |
| 38319 | hydroxycarbamide | Hydroxycarbamide | hydroxycarbamide oral solution 500mg/5ml |  |
| 3873 | hydroxycarbamide | Hydroxycarbamide | hydroxyurea capsules 500mg |  |
| 31339 | idarubicin hydrochloride | Anthracyclines and other cytotoxic antibiotics | idarubicin hydrochloride capsules 10mg |  |
| 31984 | idarubicin hydrochloride | Anthracyclines and other cytotoxic antibiotics | idarubicin hydrochloride capsules 5mg |  |
| 43168 | ifosfamide | Alkylating agents | ifosfamide injection 2g/vial |  |
| 21295 | imatinib mesilate | Imatinib | GLIVEC capsules 100mg [NOVARTIS] |  |
| 33823 | imatinib mesilate | Imatinib | GLIVEC tablets 100mg [NOVARTIS] |  |
| 28800 | imatinib mesilate | Imatinib | GLIVEC tablets 400mg [NOVARTIS] |  |
| 21286 | imatinib mesilate | Imatinib | imatinib capsules 100mg |  |
| 29229 | imatinib mesilate | Imatinib | imatinib tablets 100mg |  |
| 21318 | imatinib mesilate | Imatinib | imatinib tablets 400mg |  |
| 18460 | leflunomide | Drugs affecting the immune response (in rheumatic disease) | ARAVA tablets 100mg [AVENTIS] |  |
| 16522 | leflunomide | Drugs affecting the immune response (in rheumatic disease) | ARAVA tablets 10mg [AVENTIS] |  |
| 17642 | leflunomide | Drugs affecting the immune response (in rheumatic disease) | ARAVA tablets 20mg [AVENTIS] |  |
| 4970 | leflunomide | Drugs affecting the immune response (in rheumatic disease) | leflunomide tablets 100mg |  |
| 4971 | leflunomide | Drugs affecting the immune response (in rheumatic disease) | leflunomide tablets 10mg |  |
| 6934 | leflunomide | Drugs affecting the immune response (in rheumatic disease) | leflunomide tablets 20mg |  |
| 25848 | lomustine | Alkylating agents | CCNU capsules 10mg [LUNDBECK] |  |
| 8404 | lomustine | Alkylating agents | CCNU capsules 40mg [LUNDBECK] |  |
| 12067 | lomustine | Alkylating agents | lomustine capsules 40mg |  |
| 30014 | mannitol/sodium phosphate/plicamycin | Anthracyclines and other cytotoxic antibiotics | MITHRACIN injection 2.5mg/vial [PFIZER] |  |
| 26343 | melphalan | Alkylating agents | ALKERAN tablets 2mg [WELLCOME] |  |
| 23270 | melphalan | Alkylating agents | ALKERAN tablets 5mg [WELLCOME] |  |
| 16929 | melphalan | Alkylating agents | melphalan tablets 2mg |  |
| 12150 | melphalan | Alkylating agents | melphalan tablets 5mg |  |
| 26580 | melphalan hydrochloride | Alkylating agents | melphalan injection 100mg/vial |  |
| 37099 | melphalan hydrochloride | Alkylating agents | melphalan powder for solution for injection 50mg |  |
| 29675 | mercaptopurine | Antimetabolites/Immunosuppressants (in chronic bowel disorders) | PURI-NETHOL tablets 50mg [WELLCOME] |  |
| 19982 | mercaptopurine | Antimetabolites/Immunosuppressants (in chronic bowel disorders) | mercaptopurine capsules 10mg |  |
| 32972 | mercaptopurine | Antimetabolites/Immunosuppressants (in chronic bowel disorders) | mercaptopurine tablets 10mg |  |
| 3450 | mercaptopurine | Antimetabolites/Immunosuppressants (in chronic bowel disorders) | mercaptopurine tablets 50mg |  |
| 3986 | mesna | Urothelial toxicity | UROMITEXAN injection 100mg/ml [BAXTER ONC] |  |
| 30607 | mesna | Urothelial toxicity | UROMITEXAN tablets 400mg [BAXTER ONC] |  |
| 8926 | mesna | Urothelial toxicity | mesna injection 100mg/ml |  |
| 20841 | mesna | Urothelial toxicity | mesna tablets 400mg |  |
| 39017 | mesna | Urothelial toxicity | mesna tablets 600mg |  |
| 21753 | methotrexate | Antimetabolites/Immunosuppressants (in chronic bowel disorders)/Drugs affecting the immune response (in rheumatic disease)/Drugs affecting the immune response (for skin conditions) | MAXTREX tablets 10mg [PHARMACIA] |  |
| 13428 | methotrexate | Antimetabolites/Immunosuppressants (in chronic bowel disorders)/Drugs affecting the immune response (in rheumatic disease)/Drugs affecting the immune response (for skin conditions) | MAXTREX tablets 2.5mg [PHARMACIA] |  |
| 41585 | methotrexate | Antimetabolites/Immunosuppressants (in chronic bowel disorders)/Drugs affecting the immune response (in rheumatic disease)/Drugs affecting the immune response (for skin conditions) | METHOTREXATE SODIUM tablets 2.5mg [WYETH PHAR] |  |
| 34929 | methotrexate | Antimetabolites/Immunosuppressants (in chronic bowel disorders)/Drugs affecting the immune response (in rheumatic disease)/Drugs affecting the immune response (for skin conditions) | METHOTREXATE tablets 10mg [HOSPIRA] |  |
| 20951 | methotrexate | Antimetabolites/Immunosuppressants (in chronic bowel disorders)/Drugs affecting the immune response (in rheumatic disease)/Drugs affecting the immune response (for skin conditions) | METHOTREXATE tablets 2.5mg [GOLDSHIELD] |  |
| 32111 | methotrexate | Antimetabolites/Immunosuppressants (in chronic bowel disorders)/Drugs affecting the immune response (in rheumatic disease)/Drugs affecting the immune response (for skin conditions) | METHOTREXATE tablets 2.5mg [HOSPIRA] |  |
| 30780 | methotrexate | Antimetabolites/Immunosuppressants (in chronic bowel disorders)/Drugs affecting the immune response (in rheumatic disease)/Drugs affecting the immune response (for skin conditions) | METHOTREXATE tablets 2.5mg [PHARMACIA] |  |
| 40356 | methotrexate | Antimetabolites/Immunosuppressants (in chronic bowel disorders)/Drugs affecting the immune response (in rheumatic disease)/Drugs affecting the immune response (for skin conditions) | METOJECT injection 10mg/0.2ml [MEDAC UK] |  |
| 37117 | methotrexate | Antimetabolites/Immunosuppressants (in chronic bowel disorders)/Drugs affecting the immune response (in rheumatic disease)/Drugs affecting the immune response (for skin conditions) | METOJECT injection 10mg/1ml [MEDAC UK] |  |
| 40284 | methotrexate | Antimetabolites/Immunosuppressants (in chronic bowel disorders)/Drugs affecting the immune response (in rheumatic disease)/Drugs affecting the immune response (for skin conditions) | METOJECT injection 15mg/0.3ml [MEDAC UK] |  |
| 27400 | methotrexate | Antimetabolites/Immunosuppressants (in chronic bowel disorders)/Drugs affecting the immune response (in rheumatic disease)/Drugs affecting the immune response (for skin conditions) | METOJECT injection 15mg/1.5ml [MEDAC UK] |  |
| 40292 | methotrexate | Antimetabolites/Immunosuppressants (in chronic bowel disorders)/Drugs affecting the immune response (in rheumatic disease)/Drugs affecting the immune response (for skin conditions) | METOJECT injection 20mg/0.4ml [MEDAC UK] |  |
| 14348 | methotrexate | Antimetabolites/Immunosuppressants (in chronic bowel disorders)/Drugs affecting the immune response (in rheumatic disease)/Drugs affecting the immune response (for skin conditions) | METOJECT injection 20mg/2ml [MEDAC UK] |  |
| 40293 | methotrexate | Antimetabolites/Immunosuppressants (in chronic bowel disorders)/Drugs affecting the immune response (in rheumatic disease)/Drugs affecting the immune response (for skin conditions) | METOJECT injection 25mg/0.5ml [MEDAC UK] |  |
| 33601 | methotrexate | Antimetabolites/Immunosuppressants (in chronic bowel disorders)/Drugs affecting the immune response (in rheumatic disease)/Drugs affecting the immune response (for skin conditions) | METOJECT injection 25mg/2.5ml [MEDAC UK] |  |
| 40280 | methotrexate | Antimetabolites/Immunosuppressants (in chronic bowel disorders)/Drugs affecting the immune response (in rheumatic disease)/Drugs affecting the immune response (for skin conditions) | METOJECT injection 7.5mg/0.15ml [MEDAC UK] |  |
| 35865 | methotrexate | Antimetabolites/Immunosuppressants (in chronic bowel disorders)/Drugs affecting the immune response (in rheumatic disease)/Drugs affecting the immune response (for skin conditions) | METOJECT injection 7.5mg/0.75ml [MEDAC UK] |  |
| 40371 | methotrexate | Antimetabolites/Immunosuppressants (in chronic bowel disorders)/Drugs affecting the immune response (in rheumatic disease)/Drugs affecting the immune response (for skin conditions) | methotrexate injection 10mg/0.2ml |  |
| 32865 | methotrexate | Antimetabolites/Immunosuppressants (in chronic bowel disorders)/Drugs affecting the immune response (in rheumatic disease)/Drugs affecting the immune response (for skin conditions) | methotrexate injection 10mg/1ml |  |
| 40281 | methotrexate | Antimetabolites/Immunosuppressants (in chronic bowel disorders)/Drugs affecting the immune response (in rheumatic disease)/Drugs affecting the immune response (for skin conditions) | methotrexate injection 15mg/0.3ml |  |
| 27404 | methotrexate | Antimetabolites/Immunosuppressants (in chronic bowel disorders)/Drugs affecting the immune response (in rheumatic disease)/Drugs affecting the immune response (for skin conditions) | methotrexate injection 15mg/1.5ml |  |
| 40273 | methotrexate | Antimetabolites/Immunosuppressants (in chronic bowel disorders)/Drugs affecting the immune response (in rheumatic disease)/Drugs affecting the immune response (for skin conditions) | methotrexate injection 20mg/0.4ml |  |
| 45165 | methotrexate | Antimetabolites/Immunosuppressants (in chronic bowel disorders)/Drugs affecting the immune response (in rheumatic disease)/Drugs affecting the immune response (for skin conditions) | methotrexate injection 20mg/1ml |  |
| 26064 | methotrexate | Antimetabolites/Immunosuppressants (in chronic bowel disorders)/Drugs affecting the immune response (in rheumatic disease)/Drugs affecting the immune response (for skin conditions) | methotrexate injection 20mg/2ml |  |
| 40328 | methotrexate | Antimetabolites/Immunosuppressants (in chronic bowel disorders)/Drugs affecting the immune response (in rheumatic disease)/Drugs affecting the immune response (for skin conditions) | methotrexate injection 25mg/0.5ml |  |
| 45558 | methotrexate | Antimetabolites/Immunosuppressants (in chronic bowel disorders)/Drugs affecting the immune response (in rheumatic disease)/Drugs affecting the immune response (for skin conditions) | methotrexate injection 25mg/1.25ml |  |
| 24634 | methotrexate | Antimetabolites/Immunosuppressants (in chronic bowel disorders)/Drugs affecting the immune response (in rheumatic disease)/Drugs affecting the immune response (for skin conditions) | methotrexate injection 25mg/2.5ml |  |
| 44908 | methotrexate | Antimetabolites/Immunosuppressants (in chronic bowel disorders)/Drugs affecting the immune response (in rheumatic disease)/Drugs affecting the immune response (for skin conditions) | methotrexate injection 30mg/0.6ml |  |
| 40301 | methotrexate | Antimetabolites/Immunosuppressants (in chronic bowel disorders)/Drugs affecting the immune response (in rheumatic disease)/Drugs affecting the immune response (for skin conditions) | methotrexate injection 7.5mg/0.15ml |  |
| 35402 | methotrexate | Antimetabolites/Immunosuppressants (in chronic bowel disorders)/Drugs affecting the immune response (in rheumatic disease)/Drugs affecting the immune response (for skin conditions) | methotrexate injection 7.5mg/0.75ml |  |
| 36800 | methotrexate | Antimetabolites/Immunosuppressants (in chronic bowel disorders)/Drugs affecting the immune response (in rheumatic disease)/Drugs affecting the immune response (for skin conditions) | methotrexate oral solution 10mg/5ml |  |
| 36849 | methotrexate | Antimetabolites/Immunosuppressants (in chronic bowel disorders)/Drugs affecting the immune response (in rheumatic disease)/Drugs affecting the immune response (for skin conditions) | methotrexate oral suspension 10mg/5ml |  |
| 28041 | methotrexate | Antimetabolites/Immunosuppressants (in chronic bowel disorders)/Drugs affecting the immune response (in rheumatic disease)/Drugs affecting the immune response (for skin conditions) | methotrexate oral suspension 12.5mg/5ml |  |
| 35752 | methotrexate | Antimetabolites/Immunosuppressants (in chronic bowel disorders)/Drugs affecting the immune response (in rheumatic disease)/Drugs affecting the immune response (for skin conditions) | methotrexate oral suspension 7.5mg/5ml |  |
| 18424 | methotrexate | Antimetabolites/Immunosuppressants (in chronic bowel disorders)/Drugs affecting the immune response (in rheumatic disease)/Drugs affecting the immune response (for skin conditions) | methotrexate sodium tablets 2.5mg |  |
| 17035 | methotrexate | Antimetabolites/Immunosuppressants (in chronic bowel disorders)/Drugs affecting the immune response (in rheumatic disease)/Drugs affecting the immune response (for skin conditions) | methotrexate suspension 2.5mg/5ml |  |
| 877 | methotrexate | Antimetabolites/Immunosuppressants (in chronic bowel disorders)/Drugs affecting the immune response (in rheumatic disease)/Drugs affecting the immune response (for skin conditions) | methotrexate tablets 10mg |  |
| 823 | methotrexate | Antimetabolites/Immunosuppressants (in chronic bowel disorders)/Drugs affecting the immune response (in rheumatic disease)/Drugs affecting the immune response (for skin conditions) | methotrexate tablets 2.5mg |  |
| 27342 | methotrexate sodium | Antimetabolites/Immunosuppressants (in chronic bowel disorders)/Drugs affecting the immune response (in rheumatic disease)/Drugs affecting the immune response (for skin conditions) | MAXTREX injection 2.5mg/ml [PHARMACIA] |  |
| 34258 | methotrexate sodium | Antimetabolites/Immunosuppressants (in chronic bowel disorders)/Drugs affecting the immune response (in rheumatic disease)/Drugs affecting the immune response (for skin conditions) | METHOTREXATE injection 20mg/0.8ml [CENT HOME] |  |
| 36167 | methotrexate sodium | Antimetabolites/Immunosuppressants (in chronic bowel disorders)/Drugs affecting the immune response (in rheumatic disease)/Drugs affecting the immune response (for skin conditions) | methotrexate injection 1000mg/10ml |  |
| 12816 | methotrexate sodium | Antimetabolites/Immunosuppressants (in chronic bowel disorders)/Drugs affecting the immune response (in rheumatic disease)/Drugs affecting the immune response (for skin conditions) | methotrexate injection 100mg/ml |  |
| 7337 | methotrexate sodium | Antimetabolites/Immunosuppressants (in chronic bowel disorders)/Drugs affecting the immune response (in rheumatic disease)/Drugs affecting the immune response (for skin conditions) | methotrexate injection 10mg/0.4ml |  |
| 7336 | methotrexate sodium | Antimetabolites/Immunosuppressants (in chronic bowel disorders)/Drugs affecting the immune response (in rheumatic disease)/Drugs affecting the immune response (for skin conditions) | methotrexate injection 12.5mg/0.5ml |  |
| 16540 | methotrexate sodium | Antimetabolites/Immunosuppressants (in chronic bowel disorders)/Drugs affecting the immune response (in rheumatic disease)/Drugs affecting the immune response (for skin conditions) | methotrexate injection 15mg/0.6ml |  |
| 18890 | methotrexate sodium | Antimetabolites/Immunosuppressants (in chronic bowel disorders)/Drugs affecting the immune response (in rheumatic disease)/Drugs affecting the immune response (for skin conditions) | methotrexate injection 17.5mg/0.7ml |  |
| 14347 | methotrexate sodium | Antimetabolites/Immunosuppressants (in chronic bowel disorders)/Drugs affecting the immune response (in rheumatic disease)/Drugs affecting the immune response (for skin conditions) | methotrexate injection 20mg/0.8ml |  |
| 17672 | methotrexate sodium | Antimetabolites/Immunosuppressants (in chronic bowel disorders)/Drugs affecting the immune response (in rheumatic disease)/Drugs affecting the immune response (for skin conditions) | methotrexate injection 22.5mg/0.9ml |  |
| 16519 | methotrexate sodium | Antimetabolites/Immunosuppressants (in chronic bowel disorders)/Drugs affecting the immune response (in rheumatic disease)/Drugs affecting the immune response (for skin conditions) | methotrexate injection 25mg/1ml |  |
| 8583 | methotrexate sodium | Antimetabolites/Immunosuppressants (in chronic bowel disorders)/Drugs affecting the immune response (in rheumatic disease)/Drugs affecting the immune response (for skin conditions) | methotrexate injection 25mg/ml |  |
| 27642 | methotrexate sodium | Antimetabolites/Immunosuppressants (in chronic bowel disorders)/Drugs affecting the immune response (in rheumatic disease)/Drugs affecting the immune response (for skin conditions) | methotrexate injection 27.5mg/1.1ml |  |
| 30703 | methotrexate sodium | Antimetabolites/Immunosuppressants (in chronic bowel disorders)/Drugs affecting the immune response (in rheumatic disease)/Drugs affecting the immune response (for skin conditions) | methotrexate injection 30mg/1.2ml |  |
| 41086 | methotrexate sodium | Antimetabolites/Immunosuppressants (in chronic bowel disorders)/Drugs affecting the immune response (in rheumatic disease)/Drugs affecting the immune response (for skin conditions) | methotrexate injection 5000mg/50ml |  |
| 32229 | methotrexate sodium | Antimetabolites/Immunosuppressants (in chronic bowel disorders)/Drugs affecting the immune response (in rheumatic disease)/Drugs affecting the immune response (for skin conditions) | methotrexate injection 500mg/20ml |  |
| 24783 | methotrexate sodium | Antimetabolites/Immunosuppressants (in chronic bowel disorders)/Drugs affecting the immune response (in rheumatic disease)/Drugs affecting the immune response (for skin conditions) | methotrexate injection 50mg/2ml |  |
| 8327 | methotrexate sodium | Antimetabolites/Immunosuppressants (in chronic bowel disorders)/Drugs affecting the immune response (in rheumatic disease)/Drugs affecting the immune response (for skin conditions) | methotrexate injection 50mg/3ml |  |
| 30932 | methotrexate sodium | Antimetabolites/Immunosuppressants (in chronic bowel disorders)/Drugs affecting the immune response (in rheumatic disease)/Drugs affecting the immune response (for skin conditions) | methotrexate injection 5mg/0.2ml |  |
| 9528 | methotrexate sodium | Antimetabolites/Immunosuppressants (in chronic bowel disorders)/Drugs affecting the immune response (in rheumatic disease)/Drugs affecting the immune response (for skin conditions) | methotrexate injection 5mg/2ml |  |
| 16570 | methotrexate sodium | Antimetabolites/Immunosuppressants (in chronic bowel disorders)/Drugs affecting the immune response (in rheumatic disease)/Drugs affecting the immune response (for skin conditions) | methotrexate injection 7.5mg/0.3ml |  |
| 14748 | methotrexate sodium | Antimetabolites/Immunosuppressants (in chronic bowel disorders)/Drugs affecting the immune response (in rheumatic disease)/Drugs affecting the immune response (for skin conditions) | methotrexate sodium injection 25mg/ml |  |
| 37396 | mitobronitol | Alkylating agents | MYELOBROMOL tablets 125mg [DURBIN] |  |
| 44478 | mitotane | Mitotane | LYSODREN tablets 500mg [LAB HRA] |  |
| 35826 | mitotane | Mitotane | mitotane tablets 500mg |  |
| 15405 | mitoxantrone hydrochloride | Anthracyclines and other cytotoxic antibiotics | NOVANTRONE concentrate for solution for infusion 2mg/ml [WYETH PHAR] |  |
| 39366 | mitoxantrone hydrochloride | Anthracyclines and other cytotoxic antibiotics | mitoxantrone concentrate for solution for infusion 10mg/5ml |  |
| 41267 | mitoxantrone hydrochloride | Anthracyclines and other cytotoxic antibiotics | mitoxantrone concentrate for solution for infusion 20mg/10ml |  |
| 33174 | mitoxantrone hydrochloride | Anthracyclines and other cytotoxic antibiotics | mitoxantrone concentrate for solution for infusion 2mg/ml |  |
| 39895 | oxaliplatin | Platinum Compounds | oxaliplatin concentrate for solution for infusion 100mg/20ml |  |
| 36714 | oxaliplatin | Platinum Compounds | oxaliplatin powder for concentrate for solution for infusion 100mg |  |
| 27293 | oxaliplatin | Platinum Compounds | oxaliplatin powder for concentrate for solution for infusion 50mg |  |
| 35384 | paclitaxel | Taxanes | TAXOL concentrate for solution for infusion 30mg/5ml [BMS] |  |
| 16173 | paclitaxel | Taxanes | TAXOL concentrate for solution for infusion 6mg/ml [BMS] |  |
| 39919 | paclitaxel | Taxanes | paclitaxel albumin bound powder for suspension for infusion 100mg |  |
| 35854 | paclitaxel | Taxanes | paclitaxel concentrate for solution for infusion 30mg/5ml |  |
| 14381 | paclitaxel | Taxanes | paclitaxel concentrate for solution for infusion 6mg/ml |  |
| 45122 | panitumumab | Panitumumab | panitumumab concentrate for solution for infusion 100mg/5ml |  |
| 37272 | pemetrexed disodium | Antimetabolites | pemetrexed powder for concentrate for solution for infusion 500mg |  |
| 3327 | penicillamine | Heavy metal poisoning/Wilson's disease/Penicillamine | DISTAMINE tablets 125mg [ALLIANCE] |  |
| 8904 | penicillamine | Heavy metal poisoning/Wilson's disease/Penicillamine | DISTAMINE tablets 250mg [ALLIANCE] |  |
| 11959 | penicillamine | Heavy metal poisoning/Wilson's disease/Penicillamine | DISTAMINE tablets 50mg [ALLIANCE] |  |
| 29721 | penicillamine | Heavy metal poisoning/Wilson's disease/Penicillamine | PENDRAMINE tablets 125mg [VIATRIS] |  |
| 20255 | penicillamine | Heavy metal poisoning/Wilson's disease/Penicillamine | PENDRAMINE tablets 250mg [VIATRIS] |  |
| 31216 | penicillamine | Heavy metal poisoning/Wilson's disease/Penicillamine | PENICILLAMINE tablets 125mg [ACTAVIS] |  |
| 40170 | penicillamine | Heavy metal poisoning/Wilson's disease/Penicillamine | PENICILLAMINE tablets 125mg [GEN (UK)] |  |
| 31120 | penicillamine | Heavy metal poisoning/Wilson's disease/Penicillamine | PENICILLAMINE tablets 125mg [IVAX] |  |
| 30925 | penicillamine | Heavy metal poisoning/Wilson's disease/Penicillamine | PENICILLAMINE tablets 250mg [ACTAVIS] |  |
| 34684 | penicillamine | Heavy metal poisoning/Wilson's disease/Penicillamine | PENICILLAMINE tablets 250mg [GEN (UK)] |  |
| 31217 | penicillamine | Heavy metal poisoning/Wilson's disease/Penicillamine | PENICILLAMINE tablets 250mg [HILLCROSS] |  |
| 643 | penicillamine | Heavy metal poisoning/Wilson's disease/Penicillamine | penicillamine tablets 125mg |  |
| 604 | penicillamine | Heavy metal poisoning/Wilson's disease/Penicillamine | penicillamine tablets 250mg |  |
| 267 | penicillamine | Heavy metal poisoning/Wilson's disease/Penicillamine | penicillamine tablets 50mg |  |
| 28605 | procarbazine hydrochloride | Procarbazine | NATULAN capsules 50mg [CAMBRIDGE] |  |
| 17186 | procarbazine hydrochloride | Procarbazine | procarbazine capsules 50mg |  |
| 44222 | raltitrexed | Antimetabolites | raltitrexed powder for concentrate for solution for infusion 2mg |  |
| 12066 | razoxane | Razoxane | RAZOXIN tablets 125mg [CAMBRIDGE] |  |
| 22640 | razoxane | Razoxane | razoxane tablets 125mg |  |
| 283 | sodium aurothiomalate | Gold salts | MYOCRISIN injection 10mg/0.5ml [SANOFI/AVE] |  |
| 3329 | sodium aurothiomalate | Gold salts | MYOCRISIN injection 20mg/0.5ml [SANOFI/AVE] |  |
| 3267 | sodium aurothiomalate | Gold salts | MYOCRISIN injection 50mg/0.5ml [SANOFI/AVE] |  |
| 10842 | sodium aurothiomalate | Gold salts | sodium aurothiomalate injection 10mg/0.5ml |  |
| 16606 | sodium aurothiomalate | Gold salts | sodium aurothiomalate injection 20mg/0.5ml |  |
| 4470 | sodium aurothiomalate | Gold salts | sodium aurothiomalate injection 50mg/0.5ml |  |
| 42362 | sorafenib tosilate | Sorafenib | NEXAVAR tablets 200mg [BAYER] |  |
| 35685 | sorafenib tosilate | Sorafenib | sorafenib tablets 200mg |  |
| 39795 | sunitinib malate | Sunitinib | SUTENT capsules 12.5mg [PFIZER] |  |
| 42993 | sunitinib malate | Sunitinib | SUTENT capsules 25mg [PFIZER] |  |
| 45450 | sunitinib malate | Sunitinib | SUTENT capsules 50mg [PFIZER] |  |
| 38871 | sunitinib malate | Sunitinib | sunitinib malate capsules 12.5mg |  |
| 44063 | sunitinib malate | Sunitinib | sunitinib malate capsules 25mg |  |
| 40580 | sunitinib malate | Sunitinib | sunitinib malate capsules 50mg |  |
| 33519 | tegafur/uracil | Antimetabolites | UFTORAL capsules 224mg + 100mg [MERCK SER] |  |
| 33520 | tegafur/uracil | Antimetabolites | uracil with tegafur capsules 224mg + 100mg |  |
| 35226 | temozolomide | Dacarbazine and temozolomide | TEMODAL capsules 20mg [SCHERING-P] |  |
| 29700 | temozolomide | Dacarbazine and temozolomide | TEMODAL capsules 250mg [SCHERING-P] |  |
| 33803 | temozolomide | Dacarbazine and temozolomide | TEMODAL capsules 5mg [SCHERING-P] |  |
| 21249 | temozolomide | Dacarbazine and temozolomide | temozolomide capsules 100mg |  |
| 42372 | temozolomide | Dacarbazine and temozolomide | temozolomide capsules 140mg |  |
| 32490 | temozolomide | Dacarbazine and temozolomide | temozolomide capsules 20mg |  |
| 21250 | temozolomide | Dacarbazine and temozolomide | temozolomide capsules 250mg |  |
| 27922 | temozolomide | Dacarbazine and temozolomide | temozolomide capsules 5mg |  |
| 40453 | temsirolimus | Temsirolimus | TORISEL concentrate for solution for infusion 30mg/1.2ml [WYETH PHAR] |  |
| 26502 | tioguanine | Antimetabolites | LANVIS tablets 40mg [WELLCOME] |  |
| 20094 | tioguanine | Antimetabolites | tioguanine tablets 40mg |  |
| 33227 | topotecan hydrochloride | Topoisomerase I Inhibitors | HYCAMTIN powder for concentrate for solution for infusion 4mg [GLAXSK PHA] |  |
| 41960 | topotecan hydrochloride | Topoisomerase I Inhibitors | topotecan powder for concentrate for solution for infusion 1mg |  |
| 27292 | trastuzumab | Trastuzumab | HERCEPTIN powder for concentrate for solution for infusion 150mg [ROCHE] |  |
| 31076 | trastuzumab | Trastuzumab | trastuzumab powder for concentrate for solution for infusion 150mg |  |
| 23871 | treosulfan | Alkylating agents | TREOSULFAN capsules 250mg [FARILLON] |  |
| 24448 | treosulfan | Alkylating agents | treosulfan capsules 250mg |  |
| 28443 | tretinoin | Tretinoin | VESANOID capsules 10mg [ROCHE] |  |
| 27752 | tretinoin | Tretinoin | tretinoin capsules 10mg |  |
| 31223 | vinblastine sulphate | Vinca alkaloids and etoposide | vinblastine sulphate injection 10mg/vial |  |
| 27224 | vincristine sulphate | Vinca alkaloids and etoposide | ONCOVIN injection 2mg [LILLY] |  |
| 18914 | vincristine sulphate | Vinca alkaloids and etoposide | vincristine sulphate injection 1mg |  |
| 40649 | vincristine sulphate | Vinca alkaloids and etoposide | vincristine sulphate injection 1mg/1ml |  |
| 26316 | vincristine sulphate | Vinca alkaloids and etoposide | vincristine sulphate injection 2mg |  |
| 40659 | vincristine sulphate | Vinca alkaloids and etoposide | vincristine sulphate injection 5mg |  |
| 42684 | vinorelbine | Vinca alkaloids and etoposide | vinorelbine concentrate for solution for infusion 10mg/1ml |  |
| 33171 | vinorelbine | Vinca alkaloids and etoposide | vinorelbine injection solution 10mg/ml |  |
| 32774 | vinorelbine tartrate | Vinca alkaloids and etoposide | vinorelbine capsules 20mg |  |

| outcome definition 94: Any broad spectrum penicillin macrolide tetracycline |
| --- |
| n= 589 prodcodes used |

| **prodcode** | **drugsubstance** | **bnfchapter** | **productname** |  |
| --- | --- | --- | --- | --- |
| 34238 | amoxicillin sodium | Broad-spectrum penicillins | AMOXICILLIN injection 1g/vial [CP PHARM] |  |
| 33840 | amoxicillin sodium | Broad-spectrum penicillins | AMOXICILLIN injection 500mg/vial [CP PHARM] |  |
| 17099 | amoxicillin sodium | Broad-spectrum penicillins | AMOXIL injection 1g/vial [SMITHKLINE] |  |
| 10771 | amoxicillin sodium | Broad-spectrum penicillins | AMOXIL injection 250mg/vial [SMITHKLINE] |  |
| 24819 | amoxicillin sodium | Broad-spectrum penicillins | AMOXIL injection 500mg/vial [SMITHKLINE] |  |
| 17509 | amoxicillin sodium | Broad-spectrum penicillins | amoxicillin injection 1g |  |
| 598 | amoxicillin sodium | Broad-spectrum penicillins | amoxicillin injection 250mg |  |
| 1746 | amoxicillin sodium | Broad-spectrum penicillins | amoxicillin injection 500mg |  |
| 244 | amoxicillin sodium/potassium clavulanate | Broad-spectrum penicillins | AUGMENTIN injection 1.2g/vial [BEECHAM] |  |
| 17852 | amoxicillin sodium/potassium clavulanate | Broad-spectrum penicillins | AUGMENTIN injection 600mg/vial [BEECHAM] |  |
| 29474 | amoxicillin sodium/potassium clavulanate | Broad-spectrum penicillins | amoxicillin with clavulanic acid injection 1g + 200mg/vial |  |
| 28592 | amoxicillin sodium/potassium clavulanate | Broad-spectrum penicillins | amoxicillin with clavulanic acid injection 500mg + 100mg/vial |  |
| 24005 | amoxicillin sodium/potassium clavulanate | Broad-spectrum penicillins | co-amoxiclav (amoxicillin and clavulanic acid) injection 1000mg+200mg |  |
| 577 | amoxicillin sodium/potassium clavulanate | Broad-spectrum penicillins | co-amoxiclav (amoxicillin and clavulanic acid) injection 500mg+100mg |  |
| 21799 | amoxicillin trihydrate | Broad-spectrum penicillins | ALMODAN capsules 250mg [BERK] |  |
| 21827 | amoxicillin trihydrate | Broad-spectrum penicillins | ALMODAN capsules 500mg [BERK] |  |
| 22016 | amoxicillin trihydrate | Broad-spectrum penicillins | ALMODAN sugar free syrup 125mg/5ml [BERK] |  |
| 21963 | amoxicillin trihydrate | Broad-spectrum penicillins | ALMODAN sugar free syrup 250mg/5ml [BERK] |  |
| 17282 | amoxicillin trihydrate | Broad-spectrum penicillins | ALMODAN syrup 125mg/5ml [BERK] |  |
| 21845 | amoxicillin trihydrate | Broad-spectrum penicillins | ALMODAN syrup 250mg/5ml [BERK] |  |
| 11613 | amoxicillin trihydrate | Broad-spectrum penicillins | AMIX capsules 250mg [ASHBOURNE] |  |
| 18786 | amoxicillin trihydrate | Broad-spectrum penicillins | AMIX capsules 500mg [ASHBOURNE] |  |
| 11634 | amoxicillin trihydrate | Broad-spectrum penicillins | AMIX sugar-free suspension 125mg/5ml [ASHBOURNE] |  |
| 21844 | amoxicillin trihydrate | Broad-spectrum penicillins | AMIX sugar-free suspension 250mg/5ml [ASHBOURNE] |  |
| 30498 | amoxicillin trihydrate | Broad-spectrum penicillins | AMOPEN capsules 250mg [YORKSHIRE] |  |
| 17711 | amoxicillin trihydrate | Broad-spectrum penicillins | AMOPEN capsules 500mg [YORKSHIRE] |  |
| 29697 | amoxicillin trihydrate | Broad-spectrum penicillins | AMOPEN suspension 125mg/5ml [YORKSHIRE] |  |
| 31423 | amoxicillin trihydrate | Broad-spectrum penicillins | AMOPEN suspension 250mg/5ml [YORKSHIRE] |  |
| 9243 | amoxicillin trihydrate | Broad-spectrum penicillins | AMORAM capsules 250mg [EASTERN] |  |
| 22415 | amoxicillin trihydrate | Broad-spectrum penicillins | AMORAM capsules 500mg [EASTERN] |  |
| 12378 | amoxicillin trihydrate | Broad-spectrum penicillins | AMORAM sugar-free suspension 125mg/5ml [EASTERN] |  |
| 22438 | amoxicillin trihydrate | Broad-spectrum penicillins | AMORAM sugar-free suspension 250mg/5ml [EASTERN] |  |
| 33343 | amoxicillin trihydrate | Broad-spectrum penicillins | AMOXICILLIN capsules 250mg [ACTAVIS] |  |
| 31661 | amoxicillin trihydrate | Broad-spectrum penicillins | AMOXICILLIN capsules 250mg [CO-PHARMA] |  |
| 42809 | amoxicillin trihydrate | Broad-spectrum penicillins | AMOXICILLIN capsules 250mg [CP PHARM] |  |
| 28882 | amoxicillin trihydrate | Broad-spectrum penicillins | AMOXICILLIN capsules 250mg [CROSS-PHAR] |  |
| 34435 | amoxicillin trihydrate | Broad-spectrum penicillins | AMOXICILLIN capsules 250mg [DDSA] |  |
| 30745 | amoxicillin trihydrate | Broad-spectrum penicillins | AMOXICILLIN capsules 250mg [GEN (UK)] |  |
| 25484 | amoxicillin trihydrate | Broad-spectrum penicillins | AMOXICILLIN capsules 250mg [HILLCROSS] |  |
| 34042 | amoxicillin trihydrate | Broad-spectrum penicillins | AMOXICILLIN capsules 250mg [IVAX] |  |
| 30528 | amoxicillin trihydrate | Broad-spectrum penicillins | AMOXICILLIN capsules 250mg [KENT] |  |
| 33222 | amoxicillin trihydrate | Broad-spectrum penicillins | AMOXICILLIN capsules 250mg [LAGAP] |  |
| 32872 | amoxicillin trihydrate | Broad-spectrum penicillins | AMOXICILLIN capsules 250mg [MEPRA] |  |
| 34714 | amoxicillin trihydrate | Broad-spectrum penicillins | AMOXICILLIN capsules 250mg [NEOLAB] |  |
| 30743 | amoxicillin trihydrate | Broad-spectrum penicillins | AMOXICILLIN capsules 250mg [RANBAXY] |  |
| 45267 | amoxicillin trihydrate | Broad-spectrum penicillins | AMOXICILLIN capsules 250mg [REGENT] |  |
| 23967 | amoxicillin trihydrate | Broad-spectrum penicillins | AMOXICILLIN capsules 250mg [TEVA] |  |
| 26157 | amoxicillin trihydrate | Broad-spectrum penicillins | AMOXICILLIN capsules 500mg [ACTAVIS] |  |
| 38684 | amoxicillin trihydrate | Broad-spectrum penicillins | AMOXICILLIN capsules 500mg [CP PHARM] |  |
| 35570 | amoxicillin trihydrate | Broad-spectrum penicillins | AMOXICILLIN capsules 500mg [CROSS-PHAR] |  |
| 34885 | amoxicillin trihydrate | Broad-spectrum penicillins | AMOXICILLIN capsules 500mg [DDSA] |  |
| 23740 | amoxicillin trihydrate | Broad-spectrum penicillins | AMOXICILLIN capsules 500mg [GEN (UK)] |  |
| 33692 | amoxicillin trihydrate | Broad-spectrum penicillins | AMOXICILLIN capsules 500mg [HILLCROSS] |  |
| 29463 | amoxicillin trihydrate | Broad-spectrum penicillins | AMOXICILLIN capsules 500mg [IVAX] |  |
| 33706 | amoxicillin trihydrate | Broad-spectrum penicillins | AMOXICILLIN capsules 500mg [KENT] |  |
| 44854 | amoxicillin trihydrate | Broad-spectrum penicillins | AMOXICILLIN capsules 500mg [LAGAP] |  |
| 34912 | amoxicillin trihydrate | Broad-spectrum penicillins | AMOXICILLIN capsules 500mg [NEOLAB] |  |
| 34852 | amoxicillin trihydrate | Broad-spectrum penicillins | AMOXICILLIN capsules 500mg [RANBAXY] |  |
| 31801 | amoxicillin trihydrate | Broad-spectrum penicillins | AMOXICILLIN capsules 500mg [SANDOZ] |  |
| 34001 | amoxicillin trihydrate | Broad-spectrum penicillins | AMOXICILLIN capsules 500mg [TEVA] |  |
| 42822 | amoxicillin trihydrate | Broad-spectrum penicillins | AMOXICILLIN mixture 125mg/5ml [CELLTECH] |  |
| 28872 | amoxicillin trihydrate | Broad-spectrum penicillins | AMOXICILLIN mixture 125mg/5ml [CROSS-PHAR] |  |
| 42815 | amoxicillin trihydrate | Broad-spectrum penicillins | AMOXICILLIN mixture 250mg/5ml [CELLTECH] |  |
| 33570 | amoxicillin trihydrate | Broad-spectrum penicillins | AMOXICILLIN mixture 250mg/5ml [CROSS-PHAR] |  |
| 40238 | amoxicillin trihydrate | Broad-spectrum penicillins | AMOXICILLIN mixture 250mg/5ml [MEPRA] |  |
| 42545 | amoxicillin trihydrate | Broad-spectrum penicillins | AMOXICILLIN oral suspension 125mg/5ml [ALMUS] |  |
| 41090 | amoxicillin trihydrate | Broad-spectrum penicillins | AMOXICILLIN oral suspension 250mg/5ml [ALMUS] |  |
| 34679 | amoxicillin trihydrate | Broad-spectrum penicillins | AMOXICILLIN sugar free oral suspension 125mg/5ml [ACTAVIS] |  |
| 33696 | amoxicillin trihydrate | Broad-spectrum penicillins | AMOXICILLIN sugar free oral suspension 125mg/5ml [HILLCROSS] |  |
| 34384 | amoxicillin trihydrate | Broad-spectrum penicillins | AMOXICILLIN sugar free oral suspension 125mg/5ml [KENT] |  |
| 34638 | amoxicillin trihydrate | Broad-spectrum penicillins | AMOXICILLIN sugar free oral suspension 125mg/5ml [TEVA] |  |
| 40243 | amoxicillin trihydrate | Broad-spectrum penicillins | AMOXICILLIN sugar free oral suspension 250mg/5ml [ACTAVIS] |  |
| 34232 | amoxicillin trihydrate | Broad-spectrum penicillins | AMOXICILLIN sugar free oral suspension 250mg/5ml [HILLCROSS] |  |
| 34855 | amoxicillin trihydrate | Broad-spectrum penicillins | AMOXICILLIN sugar free oral suspension 250mg/5ml [KENT] |  |
| 34775 | amoxicillin trihydrate | Broad-spectrum penicillins | AMOXICILLIN sugar free oral suspension 250mg/5ml [TEVA] |  |
| 41835 | amoxicillin trihydrate | Broad-spectrum penicillins | AMOXICILLIN sugar free powder 125mg [IVAX] |  |
| 41734 | amoxicillin trihydrate | Broad-spectrum penicillins | AMOXICILLIN sugar free powder 3g [ACTAVIS] |  |
| 33383 | amoxicillin trihydrate | Broad-spectrum penicillins | AMOXICILLIN sugar free powder 3g [HILLCROSS] |  |
| 28130 | amoxicillin trihydrate | Broad-spectrum penicillins | AMOXICILLIN sugar free powder 3g [IVAX] |  |
| 40168 | amoxicillin trihydrate | Broad-spectrum penicillins | AMOXICILLIN sugar free powder 3g [KENT] |  |
| 29858 | amoxicillin trihydrate | Broad-spectrum penicillins | AMOXICILLIN sugar free syrup 125mg/5ml [LAGAP] |  |
| 36054 | amoxicillin trihydrate | Broad-spectrum penicillins | AMOXICILLIN sugar-free suspension 125mg/5ml [ALMUS] |  |
| 31014 | amoxicillin trihydrate | Broad-spectrum penicillins | AMOXICILLIN sugar-free suspension 125mg/5ml [GEN (UK)] |  |
| 24150 | amoxicillin trihydrate | Broad-spectrum penicillins | AMOXICILLIN sugar-free suspension 125mg/5ml [IVAX] |  |
| 43229 | amoxicillin trihydrate | Broad-spectrum penicillins | AMOXICILLIN sugar-free suspension 125mg/5ml [SANDOZ] |  |
| 42732 | amoxicillin trihydrate | Broad-spectrum penicillins | AMOXICILLIN sugar-free suspension 250mg/5ml [ALMUS] |  |
| 31535 | amoxicillin trihydrate | Broad-spectrum penicillins | AMOXICILLIN sugar-free suspension 250mg/5ml [GEN (UK)] |  |
| 33699 | amoxicillin trihydrate | Broad-spectrum penicillins | AMOXICILLIN sugar-free suspension 250mg/5ml [IVAX] |  |
| 37755 | amoxicillin trihydrate | Broad-spectrum penicillins | AMOXICILLIN sugar-free suspension 250mg/5ml [SANDOZ] |  |
| 32622 | amoxicillin trihydrate | Broad-spectrum penicillins | AMOXICILLIN suspension 125mg/5ml [GEN (UK)] |  |
| 23238 | amoxicillin trihydrate | Broad-spectrum penicillins | AMOXICILLIN suspension 125mg/5ml [IVAX] |  |
| 28875 | amoxicillin trihydrate | Broad-spectrum penicillins | AMOXICILLIN suspension 125mg/5ml [RANBAXY] |  |
| 33689 | amoxicillin trihydrate | Broad-spectrum penicillins | AMOXICILLIN suspension 250mg/5ml [GEN (UK)] |  |
| 32640 | amoxicillin trihydrate | Broad-spectrum penicillins | AMOXICILLIN suspension 250mg/5ml [IVAX] |  |
| 34857 | amoxicillin trihydrate | Broad-spectrum penicillins | AMOXICILLIN syrup 125mg/5ml [ACTAVIS] |  |
| 41818 | amoxicillin trihydrate | Broad-spectrum penicillins | AMOXICILLIN syrup 125mg/5ml [BERK] |  |
| 42240 | amoxicillin trihydrate | Broad-spectrum penicillins | AMOXICILLIN syrup 125mg/5ml [CO-PHARMA] |  |
| 33690 | amoxicillin trihydrate | Broad-spectrum penicillins | AMOXICILLIN syrup 125mg/5ml [HILLCROSS] |  |
| 29337 | amoxicillin trihydrate | Broad-spectrum penicillins | AMOXICILLIN syrup 125mg/5ml [NEOLAB] |  |
| 28870 | amoxicillin trihydrate | Broad-spectrum penicillins | AMOXICILLIN syrup 125mg/5ml [TEVA] |  |
| 34760 | amoxicillin trihydrate | Broad-spectrum penicillins | AMOXICILLIN syrup 250mg/5ml [ACTAVIS] |  |
| 33165 | amoxicillin trihydrate | Broad-spectrum penicillins | AMOXICILLIN syrup 250mg/5ml [HILLCROSS] |  |
| 45317 | amoxicillin trihydrate | Broad-spectrum penicillins | AMOXICILLIN syrup 250mg/5ml [NEOLAB] |  |
| 27725 | amoxicillin trihydrate | Broad-spectrum penicillins | AMOXICILLIN syrup 250mg/5ml [TEVA] |  |
| 4154 | amoxicillin trihydrate | Broad-spectrum penicillins | AMOXIL FIZTAB tablets 125mg [BENCARD] |  |
| 1637 | amoxicillin trihydrate | Broad-spectrum penicillins | AMOXIL FIZTAB tablets 250mg [BENCARD] |  |
| 7737 | amoxicillin trihydrate | Broad-spectrum penicillins | AMOXIL FIZTAB tablets 500mg [BENCARD] |  |
| 2171 | amoxicillin trihydrate | Broad-spectrum penicillins | AMOXIL PAEDIATRIC suspension 125mg/1.25ml [SMITHKLINE] |  |
| 133 | amoxicillin trihydrate | Broad-spectrum penicillins | AMOXIL capsules 250mg [SMITHKLINE] |  |
| 847 | amoxicillin trihydrate | Broad-spectrum penicillins | AMOXIL capsules 500mg [SMITHKLINE] |  |
| 15148 | amoxicillin trihydrate | Broad-spectrum penicillins | AMOXIL dispersible tablet 500mg [SMITHKLINE] |  |
| 4010 | amoxicillin trihydrate | Broad-spectrum penicillins | AMOXIL sachets 750mg [SMITHKLINE] |  |
| 2174 | amoxicillin trihydrate | Broad-spectrum penicillins | AMOXIL sucrose free sachets 3g [SMITHKLINE] |  |
| 2153 | amoxicillin trihydrate | Broad-spectrum penicillins | AMOXIL sugar free oral suspension 125mg/5ml [SMITHKLINE] |  |
| 1812 | amoxicillin trihydrate | Broad-spectrum penicillins | AMOXIL sugar free oral suspension 250mg/5ml [SMITHKLINE] |  |
| 3669 | amoxicillin trihydrate | Broad-spectrum penicillins | AMOXYMED capsules 250mg [MEDIPHARMA] |  |
| 31286 | amoxicillin trihydrate | Broad-spectrum penicillins | AMOXYMED syrup 125mg/5ml [MEDIPHARMA] |  |
| 27714 | amoxicillin trihydrate | Broad-spectrum penicillins | AMRIT capsules 250mg [BHR] |  |
| 33112 | amoxicillin trihydrate | Broad-spectrum penicillins | AMRIT capsules 500mg [BHR] |  |
| 33109 | amoxicillin trihydrate | Broad-spectrum penicillins | AMRIT suspension 125mg/5ml [BHR] |  |
| 33110 | amoxicillin trihydrate | Broad-spectrum penicillins | AMRIT suspension 250mg/5ml [BHR] |  |
| 18930 | amoxicillin trihydrate | Broad-spectrum penicillins | FLEMOXIN soluble tablet 375mg [P & B] |  |
| 24396 | amoxicillin trihydrate | Broad-spectrum penicillins | FLEMOXIN soluble tablet 750mg [P & B] |  |
| 14371 | amoxicillin trihydrate | Broad-spectrum penicillins | GALENAMOX capsules 250mg [GALEN] |  |
| 14396 | amoxicillin trihydrate | Broad-spectrum penicillins | GALENAMOX capsules 500mg [GALEN] |  |
| 14386 | amoxicillin trihydrate | Broad-spectrum penicillins | GALENAMOX sugar free oral suspension 125mg/5ml [GALEN] |  |
| 14407 | amoxicillin trihydrate | Broad-spectrum penicillins | GALENAMOX sugar free syrup 250mg/5ml [GALEN] |  |
| 24203 | amoxicillin trihydrate | Broad-spectrum penicillins | RESPILLIN capsules 250mg [OPD] |  |
| 24200 | amoxicillin trihydrate | Broad-spectrum penicillins | RESPILLIN capsules 500mg [OPD] |  |
| 22017 | amoxicillin trihydrate | Broad-spectrum penicillins | RESPILLIN sugar free syrup 125mg/5ml [OPD] |  |
| 22015 | amoxicillin trihydrate | Broad-spectrum penicillins | RESPILLIN syrup 125mg/5ml [OPD] |  |
| 21829 | amoxicillin trihydrate | Broad-spectrum penicillins | ZOXYCIL capsules 250mg [TRINITY] |  |
| 26262 | amoxicillin trihydrate | Broad-spectrum penicillins | ZOXYCIL capsules 500mg [TRINITY] |  |
| 9 | amoxicillin trihydrate | Broad-spectrum penicillins | amoxicillin capsules 250mg |  |
| 48 | amoxicillin trihydrate | Broad-spectrum penicillins | amoxicillin capsules 500mg |  |
| 1722 | amoxicillin trihydrate | Broad-spectrum penicillins | amoxicillin dispersible tablet 500mg |  |
| 485 | amoxicillin trihydrate | Broad-spectrum penicillins | amoxicillin paediatric suspension 125mg/1.25ml |  |
| 17746 | amoxicillin trihydrate | Broad-spectrum penicillins | amoxicillin soluble tablet 375mg |  |
| 4582 | amoxicillin trihydrate | Broad-spectrum penicillins | amoxicillin soluble tablet 750mg |  |
| 3742 | amoxicillin trihydrate | Broad-spectrum penicillins | amoxicillin sugar free chewable tablets 125mg |  |
| 870 | amoxicillin trihydrate | Broad-spectrum penicillins | amoxicillin sugar free chewable tablets 250mg |  |
| 2281 | amoxicillin trihydrate | Broad-spectrum penicillins | amoxicillin sugar free chewable tablets 500mg |  |
| 503 | amoxicillin trihydrate | Broad-spectrum penicillins | amoxicillin sugar free oral suspension 125mg/5ml |  |
| 585 | amoxicillin trihydrate | Broad-spectrum penicillins | amoxicillin sugar free oral suspension 250mg/5ml |  |
| 13848 | amoxicillin trihydrate | Broad-spectrum penicillins | amoxicillin sugar free powder 125mg |  |
| 1140 | amoxicillin trihydrate | Broad-spectrum penicillins | amoxicillin sugar free powder 3g |  |
| 9343 | amoxicillin trihydrate | Broad-spectrum penicillins | amoxicillin sugar free powder 750mg |  |
| 62 | amoxicillin trihydrate | Broad-spectrum penicillins | amoxicillin syrup 125mg/5ml |  |
| 427 | amoxicillin trihydrate | Broad-spectrum penicillins | amoxicillin syrup 250mg/5ml |  |
| 3979 | amoxicillin trihydrate/clarithromycin/lansoprazole | Broad-spectrum penicillins/Macrolides/Proton pump inhibitors | HELICLEAR triple pack 500mg + 500mg + 30mg [WYETH PHAR] |  |
| 5662 | amoxicillin trihydrate/clarithromycin/lansoprazole | Broad-spectrum penicillins/Macrolides/Proton pump inhibitors | amoxicillin with clarithromycin and lansoprazole triple pack 500mg + 500mg + 30mg |  |
| 11433 | amoxicillin trihydrate/clarithromycin/lansoprazole | Broad-spectrum penicillins/Macrolides/Proton pump inhibitors | clarithromycin with lansoprazole and amoxicillin triple pack 500mg + 30mg + 500mg |  |
| 15290 | amoxicillin trihydrate/clarithromycin/lansoprazole | Broad-spectrum penicillins/Macrolides/Proton pump inhibitors | lansoprazole with amoxicillin and clarithromycin triple pack 30mg + 500mg + 500mg |  |
| 22029 | amoxicillin trihydrate/potassium clavulanate | Broad-spectrum penicillins | AMICLAV tablets 250mg+125mg [ASHBOURNE] |  |
| 5341 | amoxicillin trihydrate/potassium clavulanate | Broad-spectrum penicillins | AUGMENTIN -DUO sugar-free suspension 400mg + 57mg/5ml [BEECHAM] |  |
| 2507 | amoxicillin trihydrate/potassium clavulanate | Broad-spectrum penicillins | AUGMENTIN dispersible tablet 375mg [BEECHAM] |  |
| 415 | amoxicillin trihydrate/potassium clavulanate | Broad-spectrum penicillins | AUGMENTIN sugar-free suspension 125mg + 31mg/5ml [BEECHAM] |  |
| 569 | amoxicillin trihydrate/potassium clavulanate | Broad-spectrum penicillins | AUGMENTIN sugar-free suspension 250mg + 62mg/5ml [BEECHAM] |  |
| 399 | amoxicillin trihydrate/potassium clavulanate | Broad-spectrum penicillins | AUGMENTIN tablets 375mg [BEECHAM] |  |
| 509 | amoxicillin trihydrate/potassium clavulanate | Broad-spectrum penicillins | AUGMENTIN tablets 625mg [BEECHAM] |  |
| 43548 | amoxicillin trihydrate/potassium clavulanate | Broad-spectrum penicillins | CO-AMOXICLAV sugar-free suspension 125mg + 31mg/5ml [HILLCROSS] |  |
| 28874 | amoxicillin trihydrate/potassium clavulanate | Broad-spectrum penicillins | CO-AMOXICLAV sugar-free suspension 125mg + 31mg/5ml [IVAX] |  |
| 34680 | amoxicillin trihydrate/potassium clavulanate | Broad-spectrum penicillins | CO-AMOXICLAV sugar-free suspension 125mg + 31mg/5ml [RANBAXY] |  |
| 34972 | amoxicillin trihydrate/potassium clavulanate | Broad-spectrum penicillins | CO-AMOXICLAV sugar-free suspension 125mg + 31mg/5ml [SANDOZ] |  |
| 42227 | amoxicillin trihydrate/potassium clavulanate | Broad-spectrum penicillins | CO-AMOXICLAV sugar-free suspension 250mg + 62mg/5ml [HILLCROSS] |  |
| 37304 | amoxicillin trihydrate/potassium clavulanate | Broad-spectrum penicillins | CO-AMOXICLAV sugar-free suspension 250mg + 62mg/5ml [IVAX] |  |
| 40320 | amoxicillin trihydrate/potassium clavulanate | Broad-spectrum penicillins | CO-AMOXICLAV sugar-free suspension 250mg + 62mg/5ml [RANBAXY] |  |
| 34234 | amoxicillin trihydrate/potassium clavulanate | Broad-spectrum penicillins | CO-AMOXICLAV sugar-free suspension 250mg + 62mg/5ml [TEVA] |  |
| 19209 | amoxicillin trihydrate/potassium clavulanate | Broad-spectrum penicillins | CO-AMOXICLAV tablets 250mg+125mg [ACTAVIS] |  |
| 34297 | amoxicillin trihydrate/potassium clavulanate | Broad-spectrum penicillins | CO-AMOXICLAV tablets 250mg+125mg [GEN (UK)] |  |
| 30786 | amoxicillin trihydrate/potassium clavulanate | Broad-spectrum penicillins | CO-AMOXICLAV tablets 250mg+125mg [HILLCROSS] |  |
| 28871 | amoxicillin trihydrate/potassium clavulanate | Broad-spectrum penicillins | CO-AMOXICLAV tablets 250mg+125mg [IVAX] |  |
| 33693 | amoxicillin trihydrate/potassium clavulanate | Broad-spectrum penicillins | CO-AMOXICLAV tablets 250mg+125mg [KENT] |  |
| 30783 | amoxicillin trihydrate/potassium clavulanate | Broad-spectrum penicillins | CO-AMOXICLAV tablets 250mg+125mg [RANBAXY] |  |
| 19414 | amoxicillin trihydrate/potassium clavulanate | Broad-spectrum penicillins | CO-AMOXICLAV tablets 250mg+125mg [SANDOZ] |  |
| 34734 | amoxicillin trihydrate/potassium clavulanate | Broad-spectrum penicillins | CO-AMOXICLAV tablets 250mg+125mg [TEVA] |  |
| 30705 | amoxicillin trihydrate/potassium clavulanate | Broad-spectrum penicillins | CO-AMOXICLAV tablets 500mg+125mg [GEN (UK)] |  |
| 33701 | amoxicillin trihydrate/potassium clavulanate | Broad-spectrum penicillins | CO-AMOXICLAV tablets 500mg+125mg [HILLCROSS] |  |
| 29356 | amoxicillin trihydrate/potassium clavulanate | Broad-spectrum penicillins | CO-AMOXICLAV tablets 500mg+125mg [IVAX] |  |
| 40148 | amoxicillin trihydrate/potassium clavulanate | Broad-spectrum penicillins | CO-AMOXICLAV tablets 500mg+125mg [KENT] |  |
| 34493 | amoxicillin trihydrate/potassium clavulanate | Broad-spectrum penicillins | CO-AMOXICLAV tablets 500mg+125mg [RANBAXY] |  |
| 32910 | amoxicillin trihydrate/potassium clavulanate | Broad-spectrum penicillins | CO-AMOXICLAV tablets 500mg+125mg [SANDOZ] |  |
| 29353 | amoxicillin trihydrate/potassium clavulanate | Broad-spectrum penicillins | CO-AMOXICLAV tablets 500mg+125mg [TEVA] |  |
| 44154 | amoxicillin trihydrate/potassium clavulanate | Broad-spectrum penicillins | CO-AMOXICLAV tablets 500mg+125mg [WINTHROP] |  |
| 27681 | amoxicillin trihydrate/potassium clavulanate | Broad-spectrum penicillins | RANCLAV sugar-free suspension 125mg + 31mg/5ml [RANBAXY] |  |
| 25370 | amoxicillin trihydrate/potassium clavulanate | Broad-spectrum penicillins | RANCLAV tablets 375mg [RANBAXY] |  |
| 439 | amoxicillin trihydrate/potassium clavulanate | Broad-spectrum penicillins | amoxicillin with clavulanic acid dispersible tablet |  |
| 13285 | amoxicillin trihydrate/potassium clavulanate | Broad-spectrum penicillins | amoxicillin with clavulanic acid oral suspension 125mg + 31mg/5ml |  |
| 13262 | amoxicillin trihydrate/potassium clavulanate | Broad-spectrum penicillins | amoxicillin with clavulanic acid oral suspension 250mg + 62mg/5ml |  |
| 15192 | amoxicillin trihydrate/potassium clavulanate | Broad-spectrum penicillins | amoxicillin with clavulanic acid sugar-free suspension 400mg + 57mg/5ml |  |
| 8906 | amoxicillin trihydrate/potassium clavulanate | Broad-spectrum penicillins | amoxicillin with clavulanic acid suspension 125mg + 31mg/5ml |  |
| 7636 | amoxicillin trihydrate/potassium clavulanate | Broad-spectrum penicillins | amoxicillin with clavulanic acid suspension 250mg + 62mg/5ml |  |
| 1391 | amoxicillin trihydrate/potassium clavulanate | Broad-spectrum penicillins | amoxicillin with clavulanic acid tablets 250mg + 125mg |  |
| 13216 | amoxicillin trihydrate/potassium clavulanate | Broad-spectrum penicillins | amoxicillin with clavulanic acid tablets 500mg+125mg |  |
| 24093 | amoxicillin trihydrate/potassium clavulanate | Broad-spectrum penicillins | clavulanic acid with amoxicillin dispersible tablet |  |
| 24006 | amoxicillin trihydrate/potassium clavulanate | Broad-spectrum penicillins | clavulanic acid with amoxicillin oral suspension 31mg + 125mg/5ml |  |
| 42485 | amoxicillin trihydrate/potassium clavulanate | Broad-spectrum penicillins | clavulanic acid with amoxicillin oral suspension 62mg + 250mg/5ml |  |
| 21775 | amoxicillin trihydrate/potassium clavulanate | Broad-spectrum penicillins | clavulanic acid with amoxicillin sugar-free suspension 31mg + 125mg/5ml |  |
| 20432 | amoxicillin trihydrate/potassium clavulanate | Broad-spectrum penicillins | clavulanic acid with amoxicillin sugar-free suspension 57mg + 400mg/5ml |  |
| 16612 | amoxicillin trihydrate/potassium clavulanate | Broad-spectrum penicillins | clavulanic acid with amoxicillin sugar-free suspension 62mg + 250mg/5ml |  |
| 9925 | amoxicillin trihydrate/potassium clavulanate | Broad-spectrum penicillins | clavulanic acid with amoxicillin tablets 125mg + 250mg |  |
| 13239 | amoxicillin trihydrate/potassium clavulanate | Broad-spectrum penicillins | clavulanic acid with amoxicillin tablets 125mg+500mg |  |
| 829 | amoxicillin trihydrate/potassium clavulanate | Broad-spectrum penicillins | co-amoxiclav (amoxicillin and clavulanic acid) dispersible tablet 250mg+125mg |  |
| 10200 | amoxicillin trihydrate/potassium clavulanate | Broad-spectrum penicillins | co-amoxiclav (amoxicillin and clavulanic acid) oral suspension 125mg + 31mg/5ml |  |
| 7364 | amoxicillin trihydrate/potassium clavulanate | Broad-spectrum penicillins | co-amoxiclav (amoxicillin and clavulanic acid) oral suspension 250mg + 62mg/5ml |  |
| 1638 | amoxicillin trihydrate/potassium clavulanate | Broad-spectrum penicillins | co-amoxiclav (amoxicillin and clavulanic acid) sugar-free suspension 125mg + 31mg/5ml |  |
| 524 | amoxicillin trihydrate/potassium clavulanate | Broad-spectrum penicillins | co-amoxiclav (amoxicillin and clavulanic acid) sugar-free suspension 250mg + 62mg/5ml |  |
| 6687 | amoxicillin trihydrate/potassium clavulanate | Broad-spectrum penicillins | co-amoxiclav (amoxicillin and clavulanic acid) sugar-free suspension 400mg + 57mg/5ml |  |
| 545 | amoxicillin trihydrate/potassium clavulanate | Broad-spectrum penicillins | co-amoxiclav (amoxicillin and clavulanic acid) tablets 250mg+125mg |  |
| 641 | amoxicillin trihydrate/potassium clavulanate | Broad-spectrum penicillins | co-amoxiclav (amoxicillin and clavulanic acid) tablets 500mg+125mg |  |
| 17222 | amphotericin/tetracycline | Tetracyclines | MYSTECLIN syrup [BMS] |  |
| 21926 | ampicillin sodium | Broad-spectrum penicillins | AMFIPEN injection 500mg/vial [YAMANOUCHI] |  |
| 30630 | ampicillin sodium | Broad-spectrum penicillins | PENBRITIN injection 250mg/vial [BEECHAM] |  |
| 7531 | ampicillin sodium | Broad-spectrum penicillins | PENBRITIN injection 500mg/vial [BEECHAM] |  |
| 31473 | ampicillin sodium | Broad-spectrum penicillins | VIDOPEN injection 250mg/vial [BERK] |  |
| 10685 | ampicillin sodium | Broad-spectrum penicillins | ampicillin injection 250mg |  |
| 14485 | ampicillin sodium | Broad-spectrum penicillins | ampicillin injection 500mg |  |
| 12083 | ampicillin sodium/cloxacillin sodium | Penicillinase-resistant penicillins/Broad-spectrum penicillins | AMPICLOX NEONATAL sugar-free suspension 90mg/0.6ml [BEECHAM] |  |
| 10755 | ampicillin sodium/cloxacillin sodium | Penicillinase-resistant penicillins/Broad-spectrum penicillins | ampicillin with cloxacillin sugar-free suspension 60mg + 30mg/0.6ml |  |
| 32148 | ampicillin trihydrate | Broad-spectrum penicillins | AMFIPEN FORTE syrup 250mg/5ml [YAMANOUCHI] |  |
| 20531 | ampicillin trihydrate | Broad-spectrum penicillins | AMFIPEN capsules 250mg [YAMANOUCHI] |  |
| 26356 | ampicillin trihydrate | Broad-spectrum penicillins | AMFIPEN capsules 500mg [YAMANOUCHI] |  |
| 32347 | ampicillin trihydrate | Broad-spectrum penicillins | AMFIPEN syrup 125mg/5ml [YAMANOUCHI] |  |
| 41646 | ampicillin trihydrate | Broad-spectrum penicillins | AMPICILLIN capsules 250mg [BERK] |  |
| 34228 | ampicillin trihydrate | Broad-spectrum penicillins | AMPICILLIN capsules 250mg [HILLCROSS] |  |
| 41647 | ampicillin trihydrate | Broad-spectrum penicillins | AMPICILLIN capsules 500mg [ACTAVIS] |  |
| 26174 | ampicillin trihydrate | Broad-spectrum penicillins | AMPICILLIN capsules 500mg [HILLCROSS] |  |
| 41744 | ampicillin trihydrate | Broad-spectrum penicillins | AMPICILLIN suspension 125mg/5ml [HILLCROSS] |  |
| 31156 | ampicillin trihydrate | Broad-spectrum penicillins | AMPITRIN capsules 250mg [OPD] |  |
| 31154 | ampicillin trihydrate | Broad-spectrum penicillins | AMPITRIN capsules 500mg [OPD] |  |
| 32760 | ampicillin trihydrate | Broad-spectrum penicillins | AMPITRIN suspension 125mg/5ml [OPD] |  |
| 38091 | ampicillin trihydrate | Broad-spectrum penicillins | PENBRITIN FORTE syrup 250mg/5ml [CHEMIDEX] |  |
| 18934 | ampicillin trihydrate | Broad-spectrum penicillins | PENBRITIN PAEDIATRIC suspension 125mg/1.25ml [BEECHAM] |  |
| 204 | ampicillin trihydrate | Broad-spectrum penicillins | PENBRITIN capsules 250mg [BEECHAM] |  |
| 24483 | ampicillin trihydrate | Broad-spectrum penicillins | PENBRITIN capsules 250mg [CHEMIDEX] |  |
| 15039 | ampicillin trihydrate | Broad-spectrum penicillins | PENBRITIN capsules 500mg [BEECHAM] |  |
| 31281 | ampicillin trihydrate | Broad-spectrum penicillins | PENBRITIN capsules 500mg [CHEMIDEX] |  |
| 10603 | ampicillin trihydrate | Broad-spectrum penicillins | PENBRITIN syrup 125mg/5ml [BEECHAM] |  |
| 37485 | ampicillin trihydrate | Broad-spectrum penicillins | PENBRITIN syrup 125mg/5ml [CHEMIDEX] |  |
| 4318 | ampicillin trihydrate | Broad-spectrum penicillins | PENBRITIN syrup 250mg/5ml [BEECHAM] |  |
| 21801 | ampicillin trihydrate | Broad-spectrum penicillins | VIDOPEN capsules 250mg [BERK] |  |
| 21967 | ampicillin trihydrate | Broad-spectrum penicillins | VIDOPEN capsules 500mg [BERK] |  |
| 23186 | ampicillin trihydrate | Broad-spectrum penicillins | VIDOPEN syrup 125mg/5ml [BERK] |  |
| 31471 | ampicillin trihydrate | Broad-spectrum penicillins | VIDOPEN syrup 250mg/5ml [BERK] |  |
| 115 | ampicillin trihydrate | Broad-spectrum penicillins | ampicillin capsules 250mg |  |
| 926 | ampicillin trihydrate | Broad-spectrum penicillins | ampicillin capsules 500mg |  |
| 8209 | ampicillin trihydrate | Broad-spectrum penicillins | ampicillin paediatric suspension 125mg/1.25ml |  |
| 900 | ampicillin trihydrate | Broad-spectrum penicillins | ampicillin sugar-free suspension 125mg/5ml |  |
| 16167 | ampicillin trihydrate | Broad-spectrum penicillins | ampicillin sugar-free suspension 250mg/5ml |  |
| 857 | ampicillin trihydrate | Broad-spectrum penicillins | ampicillin suspension 125mg/5ml |  |
| 106 | ampicillin trihydrate | Broad-spectrum penicillins | ampicillin suspension 250mg/5ml |  |
| 2874 | ampicillin trihydrate/flucloxacillin magnesium | Penicillinase-resistant penicillins/Broad-spectrum penicillins | MAGNAPEN syrup 250mg/5ml [CP PHARM] |  |
| 10369 | ampicillin trihydrate/flucloxacillin magnesium | Penicillinase-resistant penicillins/Broad-spectrum penicillins | ampicillin with flucloxacillin syrup 125mg+125mg |  |
| 9473 | ampicillin trihydrate/flucloxacillin magnesium | Penicillinase-resistant penicillins/Broad-spectrum penicillins | co-fluampicil (flucloxacillin and ampicillin) syrup 125mg + 125mg/5ml |  |
| 951 | ampicillin trihydrate/flucloxacillin magnesium | Penicillinase-resistant penicillins/Broad-spectrum penicillins | flucloxacillin with ampicillin syrup 125mg+125mg |  |
| 34380 | ampicillin trihydrate/flucloxacillin sodium | Penicillinase-resistant penicillins/Broad-spectrum penicillins | CO-FLUAMPICIL capsules 250mg+250mg [ACTAVIS] |  |
| 34358 | ampicillin trihydrate/flucloxacillin sodium | Penicillinase-resistant penicillins/Broad-spectrum penicillins | CO-FLUAMPICIL capsules 250mg+250mg [GEN (UK)] |  |
| 19648 | ampicillin trihydrate/flucloxacillin sodium | Penicillinase-resistant penicillins/Broad-spectrum penicillins | CO-FLUAMPICIL capsules 250mg+250mg [HILLCROSS] |  |
| 30764 | ampicillin trihydrate/flucloxacillin sodium | Penicillinase-resistant penicillins/Broad-spectrum penicillins | CO-FLUAMPICIL capsules 250mg+250mg [IVAX] |  |
| 41415 | ampicillin trihydrate/flucloxacillin sodium | Penicillinase-resistant penicillins/Broad-spectrum penicillins | CO-FLUAMPICIL capsules 250mg+250mg [KENT] |  |
| 25570 | ampicillin trihydrate/flucloxacillin sodium | Penicillinase-resistant penicillins/Broad-spectrum penicillins | CO-FLUAMPICIL capsules 250mg+250mg [SANDOZ] |  |
| 23485 | ampicillin trihydrate/flucloxacillin sodium | Penicillinase-resistant penicillins/Broad-spectrum penicillins | FLU-AMP capsules 500mg [GEN (UK)] |  |
| 308 | ampicillin trihydrate/flucloxacillin sodium | Penicillinase-resistant penicillins/Broad-spectrum penicillins | MAGNAPEN capsules 500mg [CP PHARM] |  |
| 13438 | ampicillin trihydrate/flucloxacillin sodium | Penicillinase-resistant penicillins/Broad-spectrum penicillins | MAGNAPEN injection 1g/vial [CP PHARM] |  |
| 13531 | ampicillin trihydrate/flucloxacillin sodium | Penicillinase-resistant penicillins/Broad-spectrum penicillins | MAGNAPEN injection 500mg/vial [CP PHARM] |  |
| 1450 | ampicillin trihydrate/flucloxacillin sodium | Penicillinase-resistant penicillins/Broad-spectrum penicillins | ampicillin with flucloxacillin capsules 250mg+250mg |  |
| 26510 | ampicillin trihydrate/flucloxacillin sodium | Penicillinase-resistant penicillins/Broad-spectrum penicillins | ampicillin with flucloxacillin injection 250mg+250mg |  |
| 24847 | ampicillin trihydrate/flucloxacillin sodium | Penicillinase-resistant penicillins/Broad-spectrum penicillins | ampicillin with flucloxacillin injection 500mg+500mg |  |
| 5454 | ampicillin trihydrate/flucloxacillin sodium | Penicillinase-resistant penicillins/Broad-spectrum penicillins | co-fluampicil (flucloxacillin and ampicillin) capsules 250mg+250mg |  |
| 26329 | ampicillin trihydrate/flucloxacillin sodium | Penicillinase-resistant penicillins/Broad-spectrum penicillins | co-fluampicil (flucloxacillin and ampicillin) injection 250mg+250mg |  |
| 45237 | ampicillin trihydrate/flucloxacillin sodium | Penicillinase-resistant penicillins/Broad-spectrum penicillins | co-fluampicil (flucloxacillin and ampicillin) injection 500mg+500mg |  |
| 9242 | ampicillin trihydrate/flucloxacillin sodium | Penicillinase-resistant penicillins/Broad-spectrum penicillins | flucloxacillin with ampicillin capsules 250mg+250mg |  |
| 20869 | ampicillin trihydrate/flucloxacillin sodium | Penicillinase-resistant penicillins/Broad-spectrum penicillins | flucloxacillin with ampicillin injection 250mg+250mg |  |
| 10538 | ampicillin/cloxacillin | Broad-spectrum penicillins | AMPICLOX NEONATAL injection 75mg/vial [BEECHAM] |  |
| 12382 | ampicillin/cloxacillin | Broad-spectrum penicillins | AMPICLOX capsules 500mg [BEECHAM] |  |
| 10795 | ampicillin/cloxacillin | Broad-spectrum penicillins | AMPICLOX syrup 250mg/5ml [BEECHAM] |  |
| 14484 | ampicillin/cloxacillin | Broad-spectrum penicillins | ampicillin with cloxacillin capsules 500mg |  |
| 28919 | ampicillin/cloxacillin | Broad-spectrum penicillins | ampicillin with cloxacillin injection 250mg + 250mg/vial |  |
| 28701 | ampicillin/cloxacillin | Broad-spectrum penicillins | ampicillin with cloxacillin injection 50mg + 25mg/vial |  |
| 40218 | azithromycin dihydrate | Macrolides | AZITHROMYCIN tablets 500mg [TEVA] |  |
| 43400 | azithromycin dihydrate | Macrolides | CLAMELLE tablets 500mg [ACTAVIS] |  |
| 4165 | azithromycin dihydrate | Macrolides | ZITHROMAX capsules 250mg [PFIZER] |  |
| 14514 | azithromycin dihydrate | Macrolides | ZITHROMAX suspension 40mg/ml [PFIZER] |  |
| 5335 | azithromycin dihydrate | Macrolides | ZITHROMAX tablets 500mg [PFIZER] |  |
| 5116 | azithromycin dihydrate | Macrolides | azithromycin capsules 250mg |  |
| 5057 | azithromycin dihydrate | Macrolides | azithromycin suspension 40mg/ml |  |
| 33888 | azithromycin dihydrate | Macrolides | azithromycin tablets 250mg |  |
| 743 | azithromycin dihydrate | Macrolides | azithromycin tablets 500mg |  |
| 12489 | bacampicillin hydrochloride | Broad-spectrum penicillins | AMBAXIN tablets 400mg [PHARMACIA] |  |
| 21345 | bacampicillin hydrochloride | Broad-spectrum penicillins | bacampicillin hydrochloride tablets 400mg |  |
| 2127 | chlortetracycline hydrochloride | Tetracyclines | AUREOMYCIN capsules 250mg [WYETH PHAR] |  |
| 7881 | chlortetracycline hydrochloride | Tetracyclines | chlortetracycline capsules 250mg |  |
| 13327 | chlortetracycline hydrochloride/demeclocycline hydrochloride/tetracycline hydrochloride | Tetracyclines/Oral antibacterials for acne | DETECLO tablets 300mg [GOLDSHIELD] |  |
| 2256 | chlortetracycline hydrochloride/demeclocycline hydrochloride/tetracycline hydrochloride | Tetracyclines/Oral antibacterials for acne | DETECLO tablets 300mg [WYETH PHAR] |  |
| 738 | chlortetracycline hydrochloride/demeclocycline hydrochloride/tetracycline hydrochloride | Tetracyclines/Oral antibacterials for acne | chlortetracycline with demeclocycline and tetracycline tablets |  |
| 24643 | chlortetracycline hydrochloride/demeclocycline hydrochloride/tetracycline hydrochloride | Tetracyclines/Oral antibacterials for acne | demeclocycline with chlortetracycline and tetracycline tablets |  |
| 15355 | chlortetracycline hydrochloride/demeclocycline hydrochloride/tetracycline hydrochloride | Tetracyclines/Oral antibacterials for acne | tetracycline with chlortetracycline and demeclocycline tablets |  |
| 12016 | chymotrypsin/tetracycline hydrochloride/trypsin | Tetracyclines | CHYMOCYCLAR capsules [RORER] |  |
| 18648 | ciclacillin | Broad-spectrum penicillins | CALTHOR tablets 250mg [WYETH PHAR] |  |
| 29545 | ciclacillin | Broad-spectrum penicillins | CALTHOR tablets 500mg [WYETH PHAR] |  |
| 45591 | clarithromycin | Macrolides | CLARIE XL modified release tablet 500mg [TEVA] |  |
| 41453 | clarithromycin | Macrolides | CLARITHROMYCIN suspension 125mg/5ml [RANBAXY] |  |
| 34811 | clarithromycin | Macrolides | CLARITHROMYCIN suspension 250mg/5ml [RANBAXY] |  |
| 34394 | clarithromycin | Macrolides | CLARITHROMYCIN tablets 250mg [GEN (UK)] |  |
| 34650 | clarithromycin | Macrolides | CLARITHROMYCIN tablets 250mg [HILLCROSS] |  |
| 34533 | clarithromycin | Macrolides | CLARITHROMYCIN tablets 250mg [TEVA] |  |
| 34608 | clarithromycin | Macrolides | CLARITHROMYCIN tablets 500mg [GEN (UK)] |  |
| 38163 | clarithromycin | Macrolides | CLARITHROMYCIN tablets 500mg [HILLCROSS] |  |
| 40784 | clarithromycin | Macrolides | CLARITHROMYCIN tablets 500mg [SANDOZ] |  |
| 34974 | clarithromycin | Macrolides | CLARITHROMYCIN tablets 500mg [TEVA] |  |
| 28349 | clarithromycin | Macrolides | CLAROSIP granules for oral suspension 125mg/straw [GRUNENTHAL] |  |
| 31689 | clarithromycin | Macrolides | CLAROSIP granules for oral suspension 187.5mg/straw [GRUNENTHAL] |  |
| 31690 | clarithromycin | Macrolides | CLAROSIP granules for oral suspension 250mg/straw [GRUNENTHAL] |  |
| 28289 | clarithromycin | Macrolides | KLARICID IV powder for concentrate for solution for infusion 500mg/vial [ABBOTT] |  |
| 38997 | clarithromycin | Macrolides | KLARICID PAEDIATRIC oral suspension 125mg/5ml [ABBOTT] |  |
| 39010 | clarithromycin | Macrolides | KLARICID PAEDIATRIC oral suspension 250mg/5ml [ABBOTT] |  |
| 6121 | clarithromycin | Macrolides | KLARICID XL modified release tablet 500mg [ABBOTT] |  |
| 3736 | clarithromycin | Macrolides | KLARICID paediatric suspension 125mg/5ml [ABBOTT] |  |
| 9583 | clarithromycin | Macrolides | KLARICID paediatric suspension 250mg/5ml [ABBOTT] |  |
| 14816 | clarithromycin | Macrolides | KLARICID sachets 250mg [ABBOTT] |  |
| 2719 | clarithromycin | Macrolides | KLARICID tablets 250mg [ABBOTT] |  |
| 6623 | clarithromycin | Macrolides | KLARICID tablets 500mg [ABBOTT] |  |
| 10326 | clarithromycin | Macrolides | clarithromycin granules for oral suspension 125mg/straw |  |
| 26059 | clarithromycin | Macrolides | clarithromycin granules for oral suspension 187.5mg/straw |  |
| 17645 | clarithromycin | Macrolides | clarithromycin granules for oral suspension 250mg/straw |  |
| 765 | clarithromycin | Macrolides | clarithromycin granules for suspension 250mg/sachet |  |
| 6803 | clarithromycin | Macrolides | clarithromycin modified release tablet 500mg |  |
| 331 | clarithromycin | Macrolides | clarithromycin paediatric suspension 125mg/5ml |  |
| 5357 | clarithromycin | Macrolides | clarithromycin paediatric suspension 250mg/5ml |  |
| 13323 | clarithromycin | Macrolides | clarithromycin powder for concentrate for solution for infusion 500mg/vial |  |
| 537 | clarithromycin | Macrolides | clarithromycin tablets 250mg |  |
| 681 | clarithromycin | Macrolides | clarithromycin tablets 500mg |  |
| 19001 | clomocycline sodium | Tetracyclines | MEGACLOR capsules 170mg [PHARMAX] |  |
| 12504 | clomocycline sodium | Tetracyclines | clomocycline capsules 170mg |  |
| 16613 | demeclocycline hydrochloride | Tetracyclines/Posterior pituitary hormones and antagonists | LEDERMYCIN capsules 150mg [GOLDSHIELD] |  |
| 7439 | demeclocycline hydrochloride | Tetracyclines/Posterior pituitary hormones and antagonists | LEDERMYCIN capsules 150mg [WYETH PHAR] |  |
| 22076 | demeclocycline hydrochloride | Tetracyclines/Posterior pituitary hormones and antagonists | LEDERMYCIN tablets 300mg [WYETH PHAR] |  |
| 9131 | demeclocycline hydrochloride | Tetracyclines/Posterior pituitary hormones and antagonists | demeclocycline capsules 150mg |  |
| 8694 | demeclocycline hydrochloride | Tetracyclines/Posterior pituitary hormones and antagonists | demeclocycline tablets 300mg |  |
| 21860 | doxycycline hyclate | Tetracyclines/Antimalarials/Oral antibacterials for acne | CYCLODOX capsules 100mg [BERK] |  |
| 21878 | doxycycline hyclate | Tetracyclines/Antimalarials/Oral antibacterials for acne | DEMIX 100 capsules [ASHBOURNE] |  |
| 21828 | doxycycline hyclate | Tetracyclines/Antimalarials/Oral antibacterials for acne | DEMIX 50 capsules [ASHBOURNE] |  |
| 21038 | doxycycline hyclate | Tetracyclines/Antimalarials/Oral antibacterials for acne | DOXATET tablets 100mg [???] |  |
| 34300 | doxycycline hyclate | Tetracyclines/Antimalarials/Oral antibacterials for acne | DOXYCYCLINE capsules 100mg [ACTAVIS] |  |
| 32066 | doxycycline hyclate | Tetracyclines/Antimalarials/Oral antibacterials for acne | DOXYCYCLINE capsules 100mg [GEN (UK)] |  |
| 24149 | doxycycline hyclate | Tetracyclines/Antimalarials/Oral antibacterials for acne | DOXYCYCLINE capsules 100mg [HILLCROSS] |  |
| 41560 | doxycycline hyclate | Tetracyclines/Antimalarials/Oral antibacterials for acne | DOXYCYCLINE capsules 100mg [IVAX] |  |
| 24126 | doxycycline hyclate | Tetracyclines/Antimalarials/Oral antibacterials for acne | DOXYCYCLINE capsules 100mg [IVAX] |  |
| 33671 | doxycycline hyclate | Tetracyclines/Antimalarials/Oral antibacterials for acne | DOXYCYCLINE capsules 100mg [KENT] |  |
| 34594 | doxycycline hyclate | Tetracyclines/Antimalarials/Oral antibacterials for acne | DOXYCYCLINE capsules 100mg [NEOLAB] |  |
| 34423 | doxycycline hyclate | Tetracyclines/Antimalarials/Oral antibacterials for acne | DOXYCYCLINE capsules 100mg [PLIVA] |  |
| 41605 | doxycycline hyclate | Tetracyclines/Antimalarials/Oral antibacterials for acne | DOXYCYCLINE capsules 100mg [SANDOZ] |  |
| 30739 | doxycycline hyclate | Tetracyclines/Antimalarials/Oral antibacterials for acne | DOXYCYCLINE capsules 100mg [TEVA] |  |
| 34765 | doxycycline hyclate | Tetracyclines/Antimalarials/Oral antibacterials for acne | DOXYCYCLINE capsules 50mg [GEN (UK)] |  |
| 34175 | doxycycline hyclate | Tetracyclines/Antimalarials/Oral antibacterials for acne | DOXYCYCLINE capsules 50mg [HILLCROSS] |  |
| 40391 | doxycycline hyclate | Tetracyclines/Antimalarials/Oral antibacterials for acne | DOXYCYCLINE capsules 50mg [IVAX] |  |
| 32419 | doxycycline hyclate | Tetracyclines/Antimalarials/Oral antibacterials for acne | DOXYCYCLINE capsules 50mg [TEVA] |  |
| 26747 | doxycycline hyclate | Tetracyclines/Antimalarials/Oral antibacterials for acne | DOXYCYCLINE tablets 100mg [NEOLAB] |  |
| 23405 | doxycycline hyclate | Tetracyclines/Antimalarials/Oral antibacterials for acne | DOXYLAR capsules 100mg [SANDOZ] |  |
| 23432 | doxycycline hyclate | Tetracyclines/Antimalarials/Oral antibacterials for acne | DOXYLAR capsules 50mg [SANDOZ] |  |
| 15071 | doxycycline hyclate | Tetracyclines/Antimalarials/Oral antibacterials for acne | NORDOX capsules 100mg [SANKYO] |  |
| 2202 | doxycycline hyclate | Tetracyclines/Antimalarials/Oral antibacterials for acne | VIBRAMYCIN 50 capsules [PFIZER] |  |
| 9267 | doxycycline hyclate | Tetracyclines/Antimalarials/Oral antibacterials for acne | VIBRAMYCIN ACNE PACK capsules containing spherical coated microgranules 50mg [PFIZER] |  |
| 268 | doxycycline hyclate | Tetracyclines/Antimalarials/Oral antibacterials for acne | VIBRAMYCIN capsules 100mg [PFIZER] |  |
| 10454 | doxycycline hyclate | Tetracyclines/Antimalarials/Oral antibacterials for acne | VIBRAMYCIN syrup 50mg/5ml [PFIZER] |  |
| 26392 | doxycycline hyclate | Tetracyclines/Antimalarials/Oral antibacterials for acne | VIBROX capsules 100mg [KENT] |  |
| 1046 | doxycycline hyclate | Tetracyclines/Antimalarials/Oral antibacterials for acne | doxycycline (as hyclate) capsules 100mg |  |
| 264 | doxycycline hyclate | Tetracyclines/Antimalarials/Oral antibacterials for acne | doxycycline (as hyclate) capsules 50mg |  |
| 12987 | doxycycline hyclate | Tetracyclines/Antimalarials/Oral antibacterials for acne | doxycycline (as hyclate) capsules containing spherical coated microgranules 50mg |  |
| 23819 | doxycycline hyclate | Tetracyclines/Antimalarials/Oral antibacterials for acne | doxycycline (as hyclate) capsules containing spherical coated microgranules 50mg |  |
| 8724 | doxycycline hyclate | Tetracyclines/Antimalarials/Oral antibacterials for acne | doxycycline (as hyclate) syrup 50mg/5ml |  |
| 970 | doxycycline hyclate | Tetracyclines/Antimalarials/Oral antibacterials for acne | doxycycline (as hyclate) tablets 100mg |  |
| 14904 | doxycycline monohydrate | Tetracyclines/Antimalarials/Oral antibacterials for acne | VIBRAMYCIN D dispersible tablet 100mg [PFIZER] |  |
| 3152 | doxycycline monohydrate | Tetracyclines/Antimalarials/Oral antibacterials for acne | VIBRAMYCIN dispersible tablet 100mg [PFIZER] |  |
| 2884 | doxycycline monohydrate | Tetracyclines/Antimalarials/Oral antibacterials for acne | doxycycline (as hyclate) dispersible tablet 100mg |  |
| 6396 | doxycycline monohydrate | Tetracyclines/Antimalarials/Oral antibacterials for acne | doxycycline monohydrate dispersible tablet 100mg |  |
| 40980 | doxycycline monohydrate | Tetracyclines/Oral antibacterials for acne | EFRACEA modified release capsules 40mg [GALDERMA] |  |
| 40796 | doxycycline monohydrate | Tetracyclines/Oral antibacterials for acne | doxycycline monohydrate modified release capsules 40mg |  |
| 14511 | erythromycin | Macrolides | ERYMAX SPRINKLE capsules 125mg [ELANPHARMA] |  |
| 14429 | erythromycin | Macrolides | erythromycin sprinkle capsules 125mg |  |
| 4489 | erythromycin | Macrolides/Oral antibacterials for acne | ERYCEN tablets 250mg [BERK] |  |
| 23017 | erythromycin | Macrolides/Oral antibacterials for acne | ERYCEN tablets 500mg [BERK] |  |
| 10190 | erythromycin | Macrolides/Oral antibacterials for acne | ERYMAX capsules 250mg [CEPHALON] |  |
| 318 | erythromycin | Macrolides/Oral antibacterials for acne | ERYMAX capsules 250mg [ELANPHARMA] |  |
| 9148 | erythromycin | Macrolides/Oral antibacterials for acne | ERYTHROMID DS tablets 500mg [ABBOTT] |  |
| 3209 | erythromycin | Macrolides/Oral antibacterials for acne | ERYTHROMID tablets 250mg [ABBOTT] |  |
| 29344 | erythromycin | Macrolides/Oral antibacterials for acne | ERYTHROMYCIN EC tablets 250mg [ACTAVIS] |  |
| 33686 | erythromycin | Macrolides/Oral antibacterials for acne | ERYTHROMYCIN capsules 250mg [HILLCROSS] |  |
| 34512 | erythromycin | Macrolides/Oral antibacterials for acne | ERYTHROMYCIN capsules of enteric coated granules 250mg [PLIVA] |  |
| 34837 | erythromycin | Macrolides/Oral antibacterials for acne | ERYTHROMYCIN enteric coated tablets 250mg [CO-PHARMA] |  |
| 42296 | erythromycin | Macrolides/Oral antibacterials for acne | ERYTHROMYCIN enteric coated tablets 250mg [DR REDDY`S] |  |
| 34334 | erythromycin | Macrolides/Oral antibacterials for acne | ERYTHROMYCIN enteric coated tablets 250mg [GEN (UK)] |  |
| 34479 | erythromycin | Macrolides/Oral antibacterials for acne | ERYTHROMYCIN enteric coated tablets 250mg [SOVEREIGN] |  |
| 33703 | erythromycin | Macrolides/Oral antibacterials for acne | ERYTHROMYCIN tablets 250mg [ABBOTT] |  |
| 42661 | erythromycin | Macrolides/Oral antibacterials for acne | ERYTHROMYCIN tablets 250mg [ALMUS] |  |
| 34873 | erythromycin | Macrolides/Oral antibacterials for acne | ERYTHROMYCIN tablets 250mg [BERK] |  |
| 34189 | erythromycin | Macrolides/Oral antibacterials for acne | ERYTHROMYCIN tablets 250mg [CP PHARM] |  |
| 24127 | erythromycin | Macrolides/Oral antibacterials for acne | ERYTHROMYCIN tablets 250mg [HILLCROSS] |  |
| 24129 | erythromycin | Macrolides/Oral antibacterials for acne | ERYTHROMYCIN tablets 250mg [IVAX] |  |
| 31530 | erythromycin | Macrolides/Oral antibacterials for acne | ERYTHROMYCIN tablets 250mg [RANBAXY] |  |
| 33685 | erythromycin | Macrolides/Oral antibacterials for acne | ERYTHROMYCIN tablets 250mg [TEVA] |  |
| 34869 | erythromycin | Macrolides/Oral antibacterials for acne | ERYTHROMYCIN tablets 500mg [CP PHARM] |  |
| 41604 | erythromycin | Macrolides/Oral antibacterials for acne | ERYTHROMYCIN tablets 500mg [HILLCROSS] |  |
| 26365 | erythromycin | Macrolides/Oral antibacterials for acne | ERYTHROMYCIN tablets 500mg [IVAX] |  |
| 18643 | erythromycin | Macrolides/Oral antibacterials for acne | ILOSONE tablets 500mg [DISTA] |  |
| 23244 | erythromycin | Macrolides/Oral antibacterials for acne | ILOTYCIN tablets 250mg [LILLY] |  |
| 33304 | erythromycin | Macrolides/Oral antibacterials for acne | KERYMAX capsules of enteric coated granules 250mg [KENT] |  |
| 31428 | erythromycin | Macrolides/Oral antibacterials for acne | RETCIN tablets 250mg [DDSA] |  |
| 11611 | erythromycin | Macrolides/Oral antibacterials for acne | ROMMIX tablets 250mg [ASHBOURNE] |  |
| 25278 | erythromycin | Macrolides/Oral antibacterials for acne | ROMMIX tablets 500mg [ASHBOURNE] |  |
| 25280 | erythromycin | Macrolides/Oral antibacterials for acne | TILORYTH capsules 250mg [TILLOMED] |  |
| 103 | erythromycin | Macrolides/Oral antibacterials for acne | erythromycin capsules of enteric coated granules 250mg |  |
| 63 | erythromycin | Macrolides/Oral antibacterials for acne | erythromycin enteric coated tablets 250mg |  |
| 401 | erythromycin | Macrolides/Oral antibacterials for acne | erythromycin enteric coated tablets 500mg |  |
| 29154 | erythromycin estolate | Macrolides/Oral antibacterials for acne | ERYTHROMYCIN capsules 250mg [ACTAVIS] |  |
| 17207 | erythromycin estolate | Macrolides/Oral antibacterials for acne | ILOSONE capsules 250mg [DISTA] |  |
| 18682 | erythromycin estolate | Macrolides/Oral antibacterials for acne | ILOSONE suspension 125mg/5ml [DISTA] |  |
| 19330 | erythromycin estolate | Macrolides/Oral antibacterials for acne | ILOSONE suspension 250mg/5ml [DISTA] |  |
| 9903 | erythromycin estolate | Macrolides/Oral antibacterials for acne | erythromycin estolate capsules 250mg |  |
| 37796 | erythromycin estolate | Macrolides/Oral antibacterials for acne | erythromycin estolate suspension 125mg/5ml |  |
| 40073 | erythromycin estolate | Macrolides/Oral antibacterials for acne | erythromycin estolate suspension 250mg/5ml |  |
| 37694 | erythromycin estolate | Macrolides/Oral antibacterials for acne | erythromycin estolate tablets 500mg |  |
| 36544 | erythromycin ethylsuccinate | Macrolides | ARPIMYCIN sugar-free suspension 125mg/5ml [ROSEMONT] |  |
| 36514 | erythromycin ethylsuccinate | Macrolides | ARPIMYCIN sugar-free suspension 250mg/5ml [ROSEMONT] |  |
| 27495 | erythromycin ethylsuccinate | Macrolides | ARPIMYCIN suspension 125mg/5ml [ROSEMONT] |  |
| 24220 | erythromycin ethylsuccinate | Macrolides | ARPIMYCIN suspension 250mg/5ml [ROSEMONT] |  |
| 37022 | erythromycin ethylsuccinate | Macrolides | ARPIMYCIN suspension 500mg/5ml [ROSEMONT] |  |
| 9434 | erythromycin ethylsuccinate | Macrolides | ERYMIN sugar free oral suspension 250mg/5ml [ELANPHARMA] |  |
| 41389 | erythromycin ethylsuccinate | Macrolides | ERYTHODEN suspension 250mg/5ml [STEVENDEN] |  |
| 4153 | erythromycin ethylsuccinate | Macrolides | ERYTHROLAR suspension 250mg/5ml [LAGAP] |  |
| 42659 | erythromycin ethylsuccinate | Macrolides | ERYTHROMYCIN sugar-free suspension 125mg/5ml [ABBOTT] |  |
| 33695 | erythromycin ethylsuccinate | Macrolides | ERYTHROMYCIN sugar-free suspension 125mg/5ml [GEN (UK)] |  |
| 33697 | erythromycin ethylsuccinate | Macrolides | ERYTHROMYCIN sugar-free suspension 125mg/5ml [HILLCROSS] |  |
| 34795 | erythromycin ethylsuccinate | Macrolides | ERYTHROMYCIN sugar-free suspension 125mg/5ml [IVAX] |  |
| 33705 | erythromycin ethylsuccinate | Macrolides | ERYTHROMYCIN sugar-free suspension 125mg/5ml [TEVA] |  |
| 33694 | erythromycin ethylsuccinate | Macrolides | ERYTHROMYCIN sugar-free suspension 250mg/5ml [GEN (UK)] |  |
| 32898 | erythromycin ethylsuccinate | Macrolides | ERYTHROMYCIN sugar-free suspension 250mg/5ml [HILLCROSS] |  |
| 30177 | erythromycin ethylsuccinate | Macrolides | ERYTHROMYCIN sugar-free suspension 250mg/5ml [IVAX] |  |
| 34853 | erythromycin ethylsuccinate | Macrolides | ERYTHROMYCIN sugar-free suspension 250mg/5ml [TEVA] |  |
| 31514 | erythromycin ethylsuccinate | Macrolides | ERYTHROMYCIN sugar-free suspension 500mg/5ml [ABBOTT] |  |
| 27203 | erythromycin ethylsuccinate | Macrolides | ERYTHROMYCIN sugar-free suspension 500mg/5ml [TEVA] |  |
| 34231 | erythromycin ethylsuccinate | Macrolides | ERYTHROMYCIN suspension 125mg/5ml [BERK] |  |
| 13167 | erythromycin ethylsuccinate | Macrolides | ERYTHROMYCIN suspension 125mg/5ml [HILLCROSS] |  |
| 33248 | erythromycin ethylsuccinate | Macrolides | ERYTHROMYCIN suspension 125mg/5ml [IVAX] |  |
| 34779 | erythromycin ethylsuccinate | Macrolides | ERYTHROMYCIN suspension 125mg/5ml [SANDOZ] |  |
| 13120 | erythromycin ethylsuccinate | Macrolides | ERYTHROMYCIN suspension 250mg/5ml [HILLCROSS] |  |
| 41584 | erythromycin ethylsuccinate | Macrolides | ERYTHROMYCIN suspension 250mg/5ml [IVAX] |  |
| 32902 | erythromycin ethylsuccinate | Macrolides | ERYTHROMYCIN suspension 250mg/5ml [KENT] |  |
| 30980 | erythromycin ethylsuccinate | Macrolides | ERYTHROMYCIN suspension 500mg/5ml [KENT] |  |
| 4596 | erythromycin ethylsuccinate | Macrolides | ERYTHROPED A sachets 1g [ABBOTT] |  |
| 327 | erythromycin ethylsuccinate | Macrolides | ERYTHROPED A tablets 500mg [ABBOTT] |  |
| 39632 | erythromycin ethylsuccinate | Macrolides | ERYTHROPED A tablets 500mg [AMDIPHARM] |  |
| 4372 | erythromycin ethylsuccinate | Macrolides | ERYTHROPED FORTE sachets 500mg [ABBOTT] |  |
| 4610 | erythromycin ethylsuccinate | Macrolides | ERYTHROPED FORTE sugar-free suspension 500mg/5ml [ABBOTT] |  |
| 39642 | erythromycin ethylsuccinate | Macrolides | ERYTHROPED FORTE sugar-free suspension 500mg/5ml [AMDIPHARM] |  |
| 993 | erythromycin ethylsuccinate | Macrolides | ERYTHROPED FORTE suspension 500mg/5ml [ABBOTT] |  |
| 3042 | erythromycin ethylsuccinate | Macrolides | ERYTHROPED PI sachets 125mg [ABBOTT] |  |
| 825 | erythromycin ethylsuccinate | Macrolides | ERYTHROPED PI sugar-free suspension 125mg/5ml [ABBOTT] |  |
| 39623 | erythromycin ethylsuccinate | Macrolides | ERYTHROPED PI sugar-free suspension 125mg/5ml [AMDIPHARM] |  |
| 997 | erythromycin ethylsuccinate | Macrolides | ERYTHROPED PI suspension 125mg/5ml [ABBOTT] |  |
| 16747 | erythromycin ethylsuccinate | Macrolides | ERYTHROPED sachets 250mg [ABBOTT] |  |
| 3572 | erythromycin ethylsuccinate | Macrolides | ERYTHROPED sugar free powder 250mg [ABBOTT] |  |
| 532 | erythromycin ethylsuccinate | Macrolides | ERYTHROPED sugar-free suspension 250mg/5ml [ABBOTT] |  |
| 39669 | erythromycin ethylsuccinate | Macrolides | ERYTHROPED sugar-free suspension 250mg/5ml [AMDIPHARM] |  |
| 105 | erythromycin ethylsuccinate | Macrolides | ERYTHROPED suspension 250mg/5ml [ABBOTT] |  |
| 30520 | erythromycin ethylsuccinate | Macrolides | PRIMACINE suspension 125mg/5ml [PINEWOOD] |  |
| 39118 | erythromycin ethylsuccinate | Macrolides | PRIMACINE suspension 250mg/5ml [PINEWOOD] |  |
| 27504 | erythromycin ethylsuccinate | Macrolides | PRIMACINE suspension 500mg/5ml [PINEWOOD] |  |
| 21808 | erythromycin ethylsuccinate | Macrolides | ROMMIX sugar-free suspension 125mg/5ml [ASHBOURNE] |  |
| 25751 | erythromycin ethylsuccinate | Macrolides | erythromycin ethylsuccinate (coated) sugar free oral suspension 250mg/5ml |  |
| 30234 | erythromycin ethylsuccinate | Macrolides | erythromycin ethylsuccinate sachets 125mg |  |
| 12330 | erythromycin ethylsuccinate | Macrolides | erythromycin ethylsuccinate sachets 1g |  |
| 13635 | erythromycin ethylsuccinate | Macrolides | erythromycin ethylsuccinate sachets 250mg |  |
| 15713 | erythromycin ethylsuccinate | Macrolides | erythromycin ethylsuccinate sachets 500mg |  |
| 4672 | erythromycin ethylsuccinate | Macrolides | erythromycin ethylsuccinate sugar-free suspension 125mg/5ml |  |
| 2225 | erythromycin ethylsuccinate | Macrolides | erythromycin ethylsuccinate sugar-free suspension 250mg/5ml |  |
| 14171 | erythromycin ethylsuccinate | Macrolides | erythromycin ethylsuccinate sugar-free suspension 500mg/5ml |  |
| 2429 | erythromycin ethylsuccinate | Macrolides | erythromycin ethylsuccinate suspension 125mg/5ml |  |
| 2376 | erythromycin ethylsuccinate | Macrolides | erythromycin ethylsuccinate suspension 250mg/5ml |  |
| 2226 | erythromycin ethylsuccinate | Macrolides | erythromycin ethylsuccinate suspension 500mg/5ml |  |
| 733 | erythromycin ethylsuccinate | Macrolides | erythromycin ethylsuccinate tablets 500mg |  |
| 397 | erythromycin ethylsuccinate | Macrolides | erythromycin suspension 125mg/5ml |  |
| 553 | erythromycin ethylsuccinate | Macrolides | erythromycin suspension 250mg/5ml |  |
| 2326 | erythromycin ethylsuccinate | Macrolides | erythromycin suspension 500mg/5ml |  |
| 37681 | erythromycin lactobionate | Macrolides | ERYTHROCIN IV powder for concentrate for solution for infusion 1g/vial [AMDIPHARM] |  |
| 201 | erythromycin lactobionate | Macrolides | ERYTHROCIN injection 1g/vial [ABBOTT] |  |
| 26189 | erythromycin lactobionate | Macrolides | erythromycin lactobionate powder for concentrate for solution for infusion 1g |  |
| 1072 | erythromycin stearate | Macrolides | ERYTHROCIN 500 tablets 500mg [ABBOTT] |  |
| 39613 | erythromycin stearate | Macrolides | ERYTHROCIN 500 tablets 500mg [AMDIPHARM] |  |
| 480 | erythromycin stearate | Macrolides | ERYTHROCIN tablets 250mg [ABBOTT] |  |
| 39616 | erythromycin stearate | Macrolides | ERYTHROCIN tablets 250mg [AMDIPHARM] |  |
| 23954 | erythromycin stearate | Macrolides | ERYTHROLAR tablets 500mg [LAGAP] |  |
| 438 | erythromycin stearate | Macrolides | erythromycin stearate tablets 250mg |  |
| 2350 | erythromycin stearate | Macrolides | erythromycin stearate tablets 500mg |  |
| 4951 | lymecycline | Tetracyclines | TETRALYSAL 300 capsules [GALDERMA] |  |
| 20054 | lymecycline | Tetracyclines | TETRALYSAL capsules 408mg [PHARMACIA] |  |
| 453 | lymecycline | Tetracyclines | lymecycline capsules 408mg |  |
| 30696 | mecillinam | Broad-spectrum penicillins | mecillinam injection 400mg/vial |  |
| 6256 | metronidazole/clarithromycin/lansoprazole | Macrolides/Metronidazole and tinidazole/Proton pump inhibitors | HELIMET triple pack [WYETH PHAR] |  |
| 6497 | metronidazole/clarithromycin/lansoprazole | Macrolides/Metronidazole and tinidazole/Proton pump inhibitors | clarithromycin with metronidazole and lansoprazole triple pack 500mg + 400mg + 30mg |  |
| 28896 | mezlocillin | Broad-spectrum penicillins | mezlocillin injection 1g/vial |  |
| 28572 | mezlocillin | Broad-spectrum penicillins | mezlocillin injection 500mg/vial |  |
| 14984 | minocycline hydrochloride | Tetracyclines/Oral antibacterials for acne | ACNAMINO MR capsules 100mg [DEXCEL] |  |
| 18728 | minocycline hydrochloride | Tetracyclines/Oral antibacterials for acne | AKNEMIN capsules 100mg [ALMIRALL] |  |
| 18684 | minocycline hydrochloride | Tetracyclines/Oral antibacterials for acne | AKNEMIN capsules 50mg [ALMIRALL] |  |
| 21978 | minocycline hydrochloride | Tetracyclines/Oral antibacterials for acne | BLEMIX 100 tablets 100mg [ASHBOURNE] |  |
| 21865 | minocycline hydrochloride | Tetracyclines/Oral antibacterials for acne | BLEMIX 50 tablets 50mg [ASHBOURNE] |  |
| 24245 | minocycline hydrochloride | Tetracyclines/Oral antibacterials for acne | CYCLOMIN tablets 100mg [BERK] |  |
| 21837 | minocycline hydrochloride | Tetracyclines/Oral antibacterials for acne | CYCLOMIN tablets 50mg [BERK] |  |
| 1039 | minocycline hydrochloride | Tetracyclines/Oral antibacterials for acne | MINOCIN MR capsules 100mg [MEDA] |  |
| 3413 | minocycline hydrochloride | Tetracyclines/Oral antibacterials for acne | MINOCIN tablets 100mg [WYETH PHAR] |  |
| 164 | minocycline hydrochloride | Tetracyclines/Oral antibacterials for acne | MINOCIN tablets 50mg [WYETH PHAR] |  |
| 34077 | minocycline hydrochloride | Tetracyclines/Oral antibacterials for acne | MINOCYCLINE modified release capsules 100mg [HILLCROSS] |  |
| 40383 | minocycline hydrochloride | Tetracyclines/Oral antibacterials for acne | MINOCYCLINE tablets 100mg [ACTAVIS] |  |
| 34926 | minocycline hydrochloride | Tetracyclines/Oral antibacterials for acne | MINOCYCLINE tablets 100mg [HILLCROSS] |  |
| 43700 | minocycline hydrochloride | Tetracyclines/Oral antibacterials for acne | MINOCYCLINE tablets 50mg [LAGAP] |  |
| 18109 | minocycline hydrochloride | Tetracyclines/Oral antibacterials for acne | SEBOMIN modified release capsules 100mg [ACTAVIS] |  |
| 37440 | minocycline hydrochloride | Tetracyclines/Oral antibacterials for acne | SEBREN MR capsules 100mg [IVAX] |  |
| 9380 | minocycline hydrochloride | Tetracyclines/Oral antibacterials for acne | minocycline capsules 100mg |  |
| 429 | minocycline hydrochloride | Tetracyclines/Oral antibacterials for acne | minocycline capsules 50mg |  |
| 2578 | minocycline hydrochloride | Tetracyclines/Oral antibacterials for acne | minocycline modified release capsules 100mg |  |
| 1532 | minocycline hydrochloride | Tetracyclines/Oral antibacterials for acne | minocycline tablets 100mg |  |
| 2999 | minocycline hydrochloride | Tetracyclines/Oral antibacterials for acne | minocycline tablets 50mg |  |
| 29418 | minocycline hydrochloride | Tetracyclines/Oropharyngeal anti-infective drugs | DENTOMYCIN dental gel 2% w/v [WYETH PHAR] |  |
| 32588 | minocycline hydrochloride | Tetracyclines/Oropharyngeal anti-infective drugs | DENTOMYCIN periodontal gel 2% [H. SCHEIN] |  |
| 17705 | minocycline hydrochloride | Tetracyclines/Oropharyngeal anti-infective drugs | minocycline dental gel 2% |  |
| 1013 | nystatin/tetracycline | Tetracyclines/Antifungals | MYSTECLIN capsules [BMS] |  |
| 1828 | nystatin/tetracycline | Tetracyclines/Antifungals | MYSTECLIN tablets [BMS] |  |
| 25071 | nystatin/tetracycline | Tetracyclines/Antifungals | tetracycline with nystatin capsules |  |
| 2922 | nystatin/tetracycline | Tetracyclines/Antifungals | tetracycline with nystatin tablets 250mg + 250,000 units |  |
| 21802 | oxytetracycline dihydrate | Tetracyclines/Oral antibacterials for acne | BERKMYCEN tablets 250mg [BERK] |  |
| 12541 | oxytetracycline dihydrate | Tetracyclines/Oral antibacterials for acne | IMPERACIN tablets 250mg [ASTRAZENEC] |  |
| 9361 | oxytetracycline dihydrate | Tetracyclines/Oral antibacterials for acne | OXYMYCIN tablets 250mg [DDSA] |  |
| 34040 | oxytetracycline dihydrate | Tetracyclines/Oral antibacterials for acne | OXYTETRACYCLINE tablets 250mg [ACTAVIS] |  |
| 34141 | oxytetracycline dihydrate | Tetracyclines/Oral antibacterials for acne | OXYTETRACYCLINE tablets 250mg [APS] |  |
| 34888 | oxytetracycline dihydrate | Tetracyclines/Oral antibacterials for acne | OXYTETRACYCLINE tablets 250mg [CP PHARM] |  |
| 34044 | oxytetracycline dihydrate | Tetracyclines/Oral antibacterials for acne | OXYTETRACYCLINE tablets 250mg [HILLCROSS] |  |
| 34336 | oxytetracycline dihydrate | Tetracyclines/Oral antibacterials for acne | OXYTETRACYCLINE tablets 250mg [IVAX] |  |
| 40483 | oxytetracycline dihydrate | Tetracyclines/Oral antibacterials for acne | OXYTETRACYCLINE tablets 250mg [SANDOZ] |  |
| 17703 | oxytetracycline dihydrate | Tetracyclines/Oral antibacterials for acne | OXYTETRAMIX tablets 250mg [ASHBOURNE] |  |
| 7455 | oxytetracycline dihydrate | Tetracyclines/Oral antibacterials for acne | TERRAMYCIN capsules 250mg [PFIZER] |  |
| 17467 | oxytetracycline dihydrate | Tetracyclines/Oral antibacterials for acne | TERRAMYCIN tablets 250mg [PFIZER] |  |
| 132 | oxytetracycline dihydrate | Tetracyclines/Oral antibacterials for acne | oxytetracycline capsules 250mg |  |
| 9034 | oxytetracycline dihydrate | Tetracyclines/Oral antibacterials for acne | oxytetracycline syrup 125mg/5ml |  |
| 77 | oxytetracycline dihydrate | Tetracyclines/Oral antibacterials for acne | oxytetracycline tablets 250mg |  |
| 2377 | pivampicillin | Broad-spectrum penicillins | PONDOCILLIN sugar-free suspension 175mg/5ml [LEO] |  |
| 2246 | pivampicillin | Broad-spectrum penicillins | PONDOCILLIN tablets 500mg [LEO] |  |
| 17181 | pivampicillin | Broad-spectrum penicillins | pivampicillin sachets 175mg |  |
| 8614 | pivampicillin | Broad-spectrum penicillins | pivampicillin suspension 175mg/5ml |  |
| 7570 | pivampicillin | Broad-spectrum penicillins | pivampicillin tablets 500mg |  |
| 25832 | pivampicillin/pivmecillinam | Broad-spectrum penicillins | pivampicillin with pivmecillinam tablets 125mg + 100mg |  |
| 12540 | pivampicillin/pivmecillinam | Broad-spectrum penicillins | pivampicillin with pivmecillinam tablets 250mg + 200mg |  |
| 21029 | pivampicillin/pivmecillinam hydrochloride | Broad-spectrum penicillins | MIRAXID 450 tablets [RPR/FISONS] |  |
| 20516 | pivampicillin/pivmecillinam hydrochloride | Broad-spectrum penicillins | MIRAXID PAEDIATRIC suspension [RPR/FISONS] |  |
| 17161 | pivampicillin/pivmecillinam hydrochloride | Broad-spectrum penicillins | MIRAXID tablets [RPR/FISONS] |  |
| 8960 | pivampicillin/pivmecillinam hydrochloride | Broad-spectrum penicillins | PONDOCILLIN PLUS tablets [BURGESS] |  |
| 23758 | potassium clavulanate/ticarcillin sodium | Broad-spectrum penicillins/Antipseudomonal penicillins | TIMENTIN infusion 1.6g/vial [BEECHAM] |  |
| 24063 | potassium clavulanate/ticarcillin sodium | Broad-spectrum penicillins/Antipseudomonal penicillins | TIMENTIN powder for concentrate for solution for infusion 3.2g/vial [BEECHAM] |  |
| 33421 | potassium clavulanate/ticarcillin sodium | Broad-spectrum penicillins/Antipseudomonal penicillins | ticarcillin with clavulanic acid powder for concentrate for solution for infusion 3g + 200mg |  |
| 25900 | spiramycin | Macrolides | spiramycin tablets 500mg |  |
| 8680 | talampicillin hydrochloride | Broad-spectrum penicillins | TALPEN syrup 125mg/5ml [BEECHAM] |  |
| 11954 | talampicillin hydrochloride | Broad-spectrum penicillins | TALPEN tablets 250mg [BEECHAM] |  |
| 16589 | talampicillin hydrochloride | Broad-spectrum penicillins | talampicillin syrup 125mg/5ml |  |
| 20007 | talampicillin hydrochloride | Broad-spectrum penicillins | talampicillin tablets 250mg |  |
| 31379 | telithromycin | Macrolides | KETEK tablets 400mg [AVENTIS] |  |
| 22964 | telithromycin | Macrolides | telithromycin tablets 400mg |  |
| 28573 | tetracycline hydrochloride | Tetracyclines/Antimalarials | ACHROMYCIN IM injection 100mg/vial [WYETH PHAR] |  |
| 31476 | tetracycline hydrochloride | Tetracyclines/Antimalarials | ACHROMYCIN IV injection 250mg/vial [WYETH PHAR] |  |
| 31230 | tetracycline hydrochloride | Tetracyclines/Antimalarials | ACHROMYCIN IV injection 500mg/vial [WYETH PHAR] |  |
| 21366 | tetracycline hydrochloride | Tetracyclines/Antimalarials | tetracycline IV injection 250mg/vial |  |
| 25274 | tetracycline hydrochloride | Tetracyclines/Antimalarials | tetracycline IV injection 500mg/vial |  |
| 15407 | tetracycline hydrochloride | Tetracyclines/Oral antibacterials for acne | ACHROMYCIN V capsules 250mg [WYETH PHAR] |  |
| 4579 | tetracycline hydrochloride | Tetracyclines/Oral antibacterials for acne | ACHROMYCIN capsules 250mg [WYETH PHAR] |  |
| 18685 | tetracycline hydrochloride | Tetracyclines/Oral antibacterials for acne | ACHROMYCIN syrup 125mg/5ml [WYETH PHAR] |  |
| 15513 | tetracycline hydrochloride | Tetracyclines/Oral antibacterials for acne | ACHROMYCIN tablets 250mg [WYETH PHAR] |  |
| 17226 | tetracycline hydrochloride | Tetracyclines/Oral antibacterials for acne | ECONOMYCIN capsules 250mg [DDSA] |  |
| 26111 | tetracycline hydrochloride | Tetracyclines/Oral antibacterials for acne | ECONOMYCIN tablets 250mg [DDSA] |  |
| 19693 | tetracycline hydrochloride | Tetracyclines/Oral antibacterials for acne | SUSTAMYCIN capsules 250mg [BOEH MANN] |  |
| 9014 | tetracycline hydrochloride | Tetracyclines/Oral antibacterials for acne | TETRABID-ORGANON capsules 250mg [ORGANON] |  |
| 8219 | tetracycline hydrochloride | Tetracyclines/Oral antibacterials for acne | TETRACHEL capsules 250mg [BERK] |  |
| 3816 | tetracycline hydrochloride | Tetracyclines/Oral antibacterials for acne | TETRACHEL tablets 250mg [BERK] |  |
| 41547 | tetracycline hydrochloride | Tetracyclines/Oral antibacterials for acne | TETRACYCLINE capsules 250mg [BERK] |  |
| 41636 | tetracycline hydrochloride | Tetracyclines/Oral antibacterials for acne | TETRACYCLINE tablets 250mg [ACTAVIS] |  |
| 43538 | tetracycline hydrochloride | Tetracyclines/Oral antibacterials for acne | TETRACYCLINE tablets 250mg [HILLCROSS] |  |
| 45271 | tetracycline hydrochloride | Tetracyclines/Oral antibacterials for acne | TETRACYCLINE tablets 250mg [NUMARK] |  |
| 34011 | tetracycline hydrochloride | Tetracyclines/Oral antibacterials for acne | tetracycline capsules 250mg |  |
| 121 | tetracycline hydrochloride | Tetracyclines/Oral antibacterials for acne | tetracycline capsules 250mg |  |
| 8284 | tetracycline hydrochloride | Tetracyclines/Oral antibacterials for acne | tetracycline syrup 125mg/5ml |  |
| 386 | tetracycline hydrochloride | Tetracyclines/Oral antibacterials for acne | tetracycline tablets 250mg |  |
